# Anti-cancer effects of genistein supplementation and moderate-intensity exercise in high-fat diet-induced breast cancer via regulation of inflammation and adipose tissue metabolism in vivo and in vitro

**DOI:** 10.1186/s12906-025-04968-x

**Published:** 2025-07-02

**Authors:** Hyeji Kwon, Hyejin Han, Yeonsoo Oh, Yuri Kim, Jung-Hyun Kim

**Affiliations:** 1 Department of Nutritional Science and Food Management, Ewha Womans University, 52 Ewhayeodae-gil, Seodaemun-gu, Seoul, 03760 Republic of Korea; 2 Graduate Program in System Health Science and Engineering, Ewha Womans University, Seoul, 03760 Republic of Korea; 3 Department of Physical Education, Chung-Ang University, 84 Heukseok-ro, Dongjak-gu, Seoul, 06974 Republic of Korea

**Keywords:** Breast cancer, Genistein, Exercise, Myokine, Apoptosis, Macrophage, Adipose tissue wasting

## Abstract

**Background:**

Breast cancer represents a significant global health concern and is influenced by a range of environmental factors. Increased fat intake and physical inactivity contribute to elevated body fat levels and are strongly linked to breast cancer incidence. Genistein (GEN), isoflavone in soy-derived foods, demonstrates anti-estrogenic properties and anti-cancer effects by regulating various mechanisms such as apoptosis. Regular physical activity prevents the progression and development of cancer by releasing various myokine signaling molecules from the muscles. This study aimed to explore the potential anti-cancer effects of combining GEN supplementation with regular moderate-intensity exercise on breast cancer.

**Methods:**

Female BALB/c mice aged 5 weeks were divided into five groups and received GEN, moderate-intensity exercise, or a combination of both throughout the experiment. After 8 weeks of treatment, mammary tumor cells were inoculated into mammary fat pads. Anti-cancer effects of these treatments on apoptosis, macrophage polarization, and adipose tissue wasting mechanisms in breast tumors were analyzed. In addition, U937, a human monocytic leukemia cell line, was treated with phorbol-12-myristate-13-acetate and interleukin (IL)-4 to induce an M2 macrophage phenotype and analyzed markers for M2 polarization.

**Results:**

Moderate-intensity exercise alone or in conjunction with GEN proved effective in retarding tumor initiation and growth, leading to reduced tumor volume compared to GEN supplementation alone. The combined regimen enhanced the expression of apoptosis markers and augmented the proportion of M1 macrophages while diminishing M2 macrophages. In vitro, treatment with GEN and myokines suppressed markers of M2 macrophage polarization and expression of the JAK1/STAT6 signaling pathway. Furthermore, the study suggested that the combined intervention of GEN supplementation and moderate-intensity exercise prevented adipose tissue wasting by regulating adipogenesis, lipolysis, and systemic inflammation in subcutaneous fat.

**Conclusions:**

The potential anti-cancer effects of GEN supplementation and regular moderate-intensity exercise on breast cancer are mediated through the induction of apoptosis and inhibition of macrophage polarization. They also exert a protective effect on adipose tissue wasting.

**Supplementary Information:**

The online version contains supplementary material available at 10.1186/s12906-025-04968-x.

## Introduction

Breast cancer is a significant global health concern that is predominantly diagnosed and is regarded as the primary cause of cancer-related mortality in women [1]. The development and progression of breast cancer are associated with a multitude of risk factors, including genetic predisposition and environmental factors, such as diet, obesity, and physical inactivity [2]. In particular, environmental factors are the leading cause of most cases of breast cancer. Consequently, it is recommended that individuals avoid high-fat diets and increase their physical activity to reduce their risk of developing breast cancer.

It has been demonstrated that cancer cells interact with the tumor microenvironment, including stromal cells, during cancer progression and metastasis [3]. The tumor microenvironment plays an important role in tumor initiation, progression, and metastasis, and is increasingly recognized as a therapeutic target and biomarker for cancer patients. Macrophages are potent immune cells in the tumor microenvironment, helping tissue regeneration and development and protecting the host from external factors [4, 5]. Intratumoral macrophages are designated tumor-associated macrophages (TAMs) and are highly adaptable, capable of altering their phenotype in response to environmental cues, including M1 and M2 [3, 6]. Interferon γ (IFN-γ) or lipopolysaccharide (LPS)-stimulated M1 macrophages elevate pro-inflammatory cytokines, such as tumor necrosis factor (TNF)-α, Interleukin (IL)-6, and IL-12 and promote anti-cancer immune responses [7]. In contrast, M2 macrophages play a pivotal role in tumorigenesis by secreting anti-inflammatory cytokines and growth factors [5]. A high prevalence of M2 macrophages is observed in solid tumors, and elevated macrophage infiltration is linked to unfavorable clinical outcomes in diverse cancer types, including breast, lung, and gastric cancers [8]. Consequently, TAM targeting and M2 polarization inhibition are emerging as personalized and promising therapeutic strategies.

Most cancer patients exhibit multiple metabolic dysregulations, including protein catabolism and lipolysis, and an increase of resting energy expenditure [9, 10]. In particular, the loss of adipose tissue has a profoundly negative impact on cancer patients such as hyperlipidemia, insulin resistance, and complicating anti-tumor therapies [11]. Understanding the molecular mechanisms such as adipogenesis, lipolysis, and systemic inflammation, associated with this adipose tissue wasting in these cancer patients and identifying their preventive and inhibitory effects will be a valuable therapeutic strategy for cancer-related adipose tissue wasting.

Genistein (4’, 5, 7-trihydroxy isoflavone, GEN) is an isoflavone found primarily in soy-derived foods and is one of the most widely present phytoestrogens in the human diet [12, 13]. It has been demonstrated that GEN exerts antioxidant and anti-estrogenic effects [14, 15]. The structural and functional similarities between GEN and 17β-estradiol (E2) indicate that GEN supplementation exerts anti-estrogenic effects by inhibiting its binding to estrogen receptors, which may contribute to anti-hormone-related cancer activity [14]. Previous studies have demonstrated that GEN inhibits growth and invasion of malignant cells through the modulation of inflammation and angiogenesis, as well as tyrosine kinase activity at high concentrations [16–18]. Several studies have demonstrated that GEN reduces the proliferation of breast cancer cells through the induction of cell cycle arrest and the downregulation of DNA methylation in the promoter region of multiple tumor suppressor genes [19–22]. Additionally, GEN has been demonstrated to induce apoptosis of breast cancer cells by regulating Bcl-2, Bax, Akt, and NF-κB [12, 23–25]. Furthermore, previous research has demonstrated that GEN exerts a protective effect against breast cancer at an early stage of life, achieved by enhancing mammary gland differentiation [26]. GEN has been shown to decrease carcinogenesis, invasion of cancer cells, and angiogenesis through modulation of matrix metalloproteinases and vascular endothelial growth factor expressions [27–30]. Consequently, GEN may be a promising alternative anti-cancer agent that inhibits cancer progression.

Regular physical activity has been reported to enhance muscle strength, physical function, aerobic capacity, and quality of life in women with early-stage breast cancer [31]. Additionally, exercise training has been shown to prevent the development and progression of breast cancer through the modulation of several biological pathways, including systemic low-grade inflammation, metabolic hormones, sex hormones, and others [32]. During physical activity, myokines, a type of cytokine, are released as muscles relax and contract [33, 34]. Myokines regulate the immune system, are involved in cancer development, and exhibit anti-cancer effects [35]. Recent studies have demonstrated the potential efficacy of irisin (Iri) in breast cancer prevention and treatment by stimulating caspase activity to promote apoptosis, eliciting anti-inflammatory reactions, or improving tumor responsiveness to anti-cancer medications [36]. Oncostatin (OSM), a member of the interleukin-6 cytokine family, has been shown to inhibit proliferation [37] and promote cell detachment in T-47D human breast cancer cells [38].

Although the benefits of exercise and nutrition have been extensively studied individually, the combination of the two therapies has been reported to be more effective in preventing and treating cancer and improving the quality of life of cancer patients [39–43]. One study observed the combination effect of regular swimming exercise and genistein decreased inflammatory levels and increased anti-inflammatory levels stronger than individually [41]. Additionally, another investigation reported that combination of yoga training and vitamin D supplementation in breast cancer survivors resulted in improvement of cytokine profiles, such as anti-inflammatory index (IL-10/TNF-α) [43]. However, it is still unclear whether the combination treatment of GEN and exercise has additional anti-cancer effects on breast cancer related to tumor growth and activity, or surrounding body composition. Therefore, the present study aimed to investigate the effects of GEN and regular moderate-intensity exercise on anti-cancer effects in breast cancer by regulating cell apoptosis, macrophage polarization, and adipose tissue wasting.

## Methods

### Animals and experimental procedures

Five-week-old female BALB/c mice (Central Lab Animal Inc., Seoul, Korea) were housed in a controlled environment with specific conditions including temperature (22 ± 2 ℃), humidity (50 ± 5%), and a 12-hour light/dark cycle. The mice were provided with food (DooYeol Biotech, Seoul, Korea) and water *ad libitum*. The number of mice required in each group was calculated using G*Power software. We selected an ANOVA test with an independent sample study design. The effect size was set at 0.6, the significance level (α) at 0.05, and the statistical power (1-β) at 0.9. Based on these parameters, the required number of mice per group was determined to be 10. Mice were randomly assigned to five groups on body weight: (1) Normal control mice group given AIN-93G (NC; *n* = 10), (2) 4T1-induced breast cancer mice on a high-fat diet group (H, *n* = 10), (3) 4T1-induced breast cancer mice on a high-fat diet supplemented with 200 mg/kg body weight (b.w.) of GEN group (HG, *n* = 10), (4) 4T1-induced breast cancer mice on a high-fat diet with moderate-intensity exercise group (HE, *n* = 10), and (5) 4T1-induced breast cancer mice on a high-fat diet supplemented with 200 mg/kg b.w. of GEN and moderate-intensity exercise group (HGE, *n* = 10). Mice were randomly assigned to experimental groups using stratified randomization based on initial body weight. The mice were fed an AIN-93G diet (NC) or a 60% high-fat diet (HFD) during the experiment. AIN-93G is a purified rodent diet designed to provide balanced nutrition for laboratory animals and is commonly used as a standard control diet in dietary intervention studies. Following the acclimation period, mice in the HFD group were fed a high-fat diet (60% fat composition) for 13 weeks, administered ad libitum to ensure continuous consumption. The oil in the high-fat diet was changed from soybean oil to corn oil to prevent GEN contamination.

GEN supplementation and moderate-intensity exercise were employed to study their anti-cancer effects. GEN was administered orally at a dose of 200 mg/kg five times per week, suspended in corn oil. Moderate-intensity exercise training was conducted five times a week using a treadmill (Biokonvision, Gwacheon, Korea). The exercise regimen consisted of a 10-minute warm-up at a speed of 5 m per minute, followed by a 30-minute main activity at 13 m per minute, and a 10-minute cool-down at 5 m per minute. A breast cancer mouse model was established by injecting 1 × 10^4^ 4T1 cells suspended in phosphate-buffered saline (PBS) into the mammary fat pads 8 weeks after the experiment began. Food intake, body weights, and tumor volumes were monitored biweekly. Tumor delay was defined as the duration from tumor cell inoculation to the time when the tumor became palpable and exhibited measurable growth. Tumor volumes were determined by measuring each tumor with a digital caliper and calculating it using the formula: [length (mm) x width^2^ (mm^2^) x 0.5] [44]. All experimental procedures were conducted in accordance with institutional ethical guidelines, with humane endpoints strictly observed. For tissue collection, mice were anesthetized using isoflurane (3 mL applied to a cotton pad in a sealed chamber) until unresponsive to a toe pinch. Anesthesia was maintained through intermittent exposure to isoflurane administered via a corneal tube. While under deep anesthesia, mice were euthanized by exsanguination via cardiac puncture, followed by organ harvesting. Death was confirmed by the complete cessation of heartbeat, respiration, and reflexes. Tissues were then carefully dissected, weighed, rinsed, snap-frozen in liquid nitrogen, and stored at − 80 °C. All surgical procedures were performed in a controlled environment within a laboratory fume hood, with strict adherence to personal protective equipment (PPE) protocols to minimize isoflurane exposure. The in vivo procedures were approved by the Institutional Animal Care and Use Committee of Ewha Womans University (IACUC approval No. 22–043).

### Cell culture and reagents

The murine mammary cancer cell line, 4T1 and human monocytic leukemia cell line, U937, were obtained from the American Type Culture Collection (ATCC, Manassas, VA, USA) and maintained in Roswell Park Memorial Institute (RPMI) 1640 medium (Welgene, Daegu, Korea) supplemented with 10% fetal bovine serum (FBS; Hyclone, Logan, UT, USA) and 1% penicillin-streptomycin (100 U/mL and 100 µg/mL) (Invitrogen, Carlsbad, CA, USA). The cells were cultured at 37 °C in a humidified atmosphere containing 5% CO_2_ throughout the experiment. GEN was acquired from BOC Sciences (NY, USA) and solubilized in dimethyl sulfoxide (DMSO, Sigma Aldrich, St. Louis, MO, USA). Iri was procured from Cayman Chemical (Ann Arbor, Michigan, USA), and OSM was purchased from Sino Biological (Beijing, China) and dissolved in distilled water.

### Cell viability assay

2,5-dephenyl-2 H-tetrazolium bromide (MTT) assay was conducted to evaluate cell viability. The impact of GEN, Iri, and OSM on cell viability was investigated by seeding cells at a density of 2.0 × 10^4^ cells per well in 96-well plates and incubating them for 24 h. U937 cells were differentiated using 10 ng/mL phorbol 12-myristate 13-acetate (PMA) with or without GEN, Iri, and OSM for 48 h, followed by 30 ng/mL IL-4 in the presence or absence of GEN, Iri, and OSM for an additional 48 h. After 96 h of incubation of varying concentrations of GEN, Iri, and OSM, the medium was aspirated, and 200 µl of MTT solution (0.5 mg/mL, Sigma-Aldrich) was added to each well. The mixture was then incubated at 37℃ for three hours. Subsequently, formazan crystals were dissolved in each well by adding 100 µL of DMSO solution after removing the supernatant. Absorbance values of each well were determined at 560 nm using a microplate reader (Molecular Device, Sunnyvale, CA, USA) for analysis.

### RNA extraction and reverse transcriptase-polymerase chain reaction (RT-PCR) analysis

Total RNA was isolated from mouse tissues and cells by utilizing the TRIzol reagent (Invitrogen, CA, USA). Following assessment of concentration and integrity, complementary DNA (cDNA) was synthesized from 1 µg of each total RNA using a RevertAid First Strand cDNA Synthesis Kit (Thermo Fisher Scientific, Waltham, MA, USA). The PCR amplification process involves various thermal cycling conditions, commencing with an initial step at 94 ℃ for 5 min, followed by denaturation at 94 ℃ for 30 s. Subsequent annealing is performed at a specific temperature for each primer for 45 s, and extension is carried out at 72 ℃ for 2 min. The samples were then subjected to electrophoresis on a 1.5-2.0% agarose gel and visualized using a gel documentation system. Image J software (National Institutes of Health, Bethesda, MD, USA) was employed for quantifying band intensities, with each outcome normalized to the internal control, glyceraldehyde-3-phosphate dehydrogenase (Gapdh).

### Immunohistochemistry

Tumor tissues obtained in mice with breast cancer induced by 4T1 cell injection were harvested, fixed in 10% formaldehyde, and embedded in paraffin. Following this, the paraffin-embedded samples were sliced into 4 µm sections and affixed onto glass slides. The sections underwent deparaffinization and rehydration and were then exposed overnight to primary antibodies CD68 (Proteintech, Manchester, UK) and ARG1 (Abcam, Cambridge, England), following the manufacturer’s instructions. Subsequently, a secondary antibody was added and left to incubate at room temperature for one hour. The sections were then subjected to staining using 3,3’-diaminobenxidine tetrahydrochloride (DAB; DABKO, Ely, UK), followed by counterstaining with 1% hematoxylin and eosin for one minute. Finally, the sections were mounted using a Permount Mounting medium (Fisher Scientific, Pittsburgh, PA, USA).

### Western blot analysis

Mice breast tumor tissues and cells were rinsed with 1X cold PBS and lysed using PRO-PREP protein extraction solution (iNtRON Biotechnology, Seongnam, Korea) supplemented with a phosphatase inhibitor cocktail (Sigma-Aldrich). Following protein quantification, equal quantities of the samples were loaded onto SDS-PAGE. After electrophoresis, the proteins were transferred onto polyvinylidene difluoride (PVDF) membranes (Millipore, Billerica, MA, USA). The membranes were then blocked with 5% bovine serum albumin (BSA) or 5% skim milk in Tris-buffered saline containing Tween 20 (TBS-T) at ambient temperature for one hour. The blocked membranes were incubated overnight at 4℃ with diluted primary antibodies for detection. The primary antibodies were used in this study as follows: Bcl-2, Bax, Proliferating cell nuclear antigen (PCNA) (Santa Cruz Biotechnology, Santa Cruz, CA, USA), cleaved caspase-3, total-JAK1, phospho-JAK1, total-STAT6, phospho-STAT6 (Cell Signaling Technology, Danvers, MA, USA), Arg1 (Proteintech, Manchester, UK), CD163, CD68, and β-actin (Abcam, Cambridge, England). Following multiple washes with TBS-T, the membranes were exposed to anti-mouse or anti-rabbit secondary antibodies (Santa Cruz Biotechnology) at room temperature for one hour. β-actin was utilized as a loading control. The visualization of protein bands was achieved using an enhanced chemiluminescence solution (ECL; Animal Genetics Inc, Suwon, Korea) on film. The levels of protein expression were quantified using Image J software (National Institutes of Health, Bethesda, MD, USA).

### Statistical analysis

Data were represented as mean ± standard error of the mean (SEM). Statistical analyses were performed utilizing the GraphPad PRISM software (Version 3.02, GraphPad Software, Inc., San Diego, CA, USA). Group comparisons were analyzed using a one-way analysis of variance (ANOVA), followed by the Newman–Keuls post hoc test. The Newman–Keuls test was chosen as it allows for pairwise comparisons while balancing Type I error control and statistical power. Statistical significance was determined at a *P*-value below 0.05.

## Results

### Effects of GEN supplementation, moderate-intensity exercise, or their combination on the body and organ weight in high-fat diet-induced breast cancer mice

To determine the effect of GEN supplementation and moderate-intensity exercise on breast cancer, a high-fat diet-induced breast cancer mice model was established. There was no significant difference between the initial and final body weight in each group (Table 1). Although a high-fat diet significantly increased body weight before cancer cell injection (*P* < 0.05), the final body weight was not different among the groups. Although body weight was lost significantly in the H group compared to other groups (NC, HE, HGE; *P* < 0.001, HG; *P* < 0.05), GEN supplementation and moderate-intensity exercise suppressed breast cancer-induced body weight loss. The ratio of liver weight to body weight was found to be increased in the H (*P* < 0.01) and HG groups (*P* < 0.001), respectively, compared to the NC group. The spleen serves as an indirect marker of inflammation. The ratio of spleen weight to body weight was significantly increased by about 6.7 times, 5.3 times, and 2.7 times in the H, HG, and HE groups, respectively, compared to the NC group. These results indicate that GEN, moderate-intensity exercise, and their combination suppressed body weight loss and spleen enlargement. However, the combination did not show an additive or synergistic effect on body weight loss or organ weight compared to each treatment alone.

**Table 1:** Effects of GEN supplementation, moderate-intensity exercise, or their combination on body weight and organ weight

	NC	H	HG	HE	HGE
Body weight (BW, g)					
Initial BW	18.73 ± 0.31^a^	18.17 ± 0.34^a^	18.51 ± 0.31^a^	17.60 ± 0.37^a^	17.78 ± 0.31^a^
BW before TI	20.00 ± 0.37^a^	22.04 ± 0.45^b^	21.77 ± 0.50^ab^	21.62 ± 0.63^ab^	20.93 ± 0.46^ab^
Final BW	21.18 ± 0.43^a^	20.59 ± 0.44^a^	21.27 ± 0.47^a^	21.72 ± 0.52^a^	21.02 ± 0.45^a^
BW gain or loss (g) (BW before TI to Final BW)	1.19 ± 0.23^a^	-1.45 ± 0.36^b^	-0.42 ± 0.26^c^	0.10 ± 0.20^c^	0.41 ± 0.35^c^
Liver / BW ratio (%)	3.72 ± 0.43^a^	4.52 ± 0.40^b^	4.83 ± 0.66^b^	4.01 ± 0.68^a^	3.77 ± 0.40^a^
Spleen / BW ratio (%)	0.44 ± 0.05^a^	2.93 ± 0.46^b^	2.34 ± 0.56^c^	1.20 ± 0.95^d^	0.76 ± 0.52^ad^

All data were presented as mean ± standard error of the mean (SEM) and subjected to one-way analysis of variance (ANOVA) followed by the Newman-Keuls post hoc test. Statistical significance among groups was denoted by different alphabetical letters (*P* < 0.05). TI, tumor inoculation; GEN, genistein; NC, normal control; H, breast cancer-induced mice fed a high-fat diet; HG, breast cancer-induced mice fed a high-fat diet with GEN; HE, breast cancer-induced mice fed a high-fat diet with moderate-intensity exercise; HGE, breast cancer-induced mice fed a high-fat diet with GEN and moderate-intensity exercise

### Effects of GEN supplementation, moderate-intensity exercise, or their combination on the regulation of breast tumor growth and apoptosis markers in high-fat diet-induced breast cancer mice

4T1 cells (1 × 10^4^) were injected into the mammary fat pad of mice, resulting in tumor development. Tumor incidence was suppressed by exercise (HE, *P* < 0.05) and the combination of GEN and exercise (HGE, *P* < 0.05) group compared to the H group (Table 2).

**Table 2:** Effects of GEN supplementation, moderate-intensity exercise, or their combination on breast tumor incidence

Treatment group	Tumor incidence
Ha	10/10^a^
HG^a^	10/10^a^
HE^b^	4/10^b^
HGE^b^	3/10^b^

Comparisons among the groups were evaluated by Fisher’s exact test. Statistical significance among groups was denoted by different alphabetical letters (*P* < 0.05). GEN, genistein; H, breast cancer-induced mice fed a high-fat diet; HG, breast cancer-induced mice fed a high-fat diet with GEN; HE, breast cancer-induced mice fed a high-fat diet with moderate-intensity exercise; HGE, breast cancer-induced mice fed a high-fat diet with GEN and moderate-intensity exercise

**Fig. 1:**
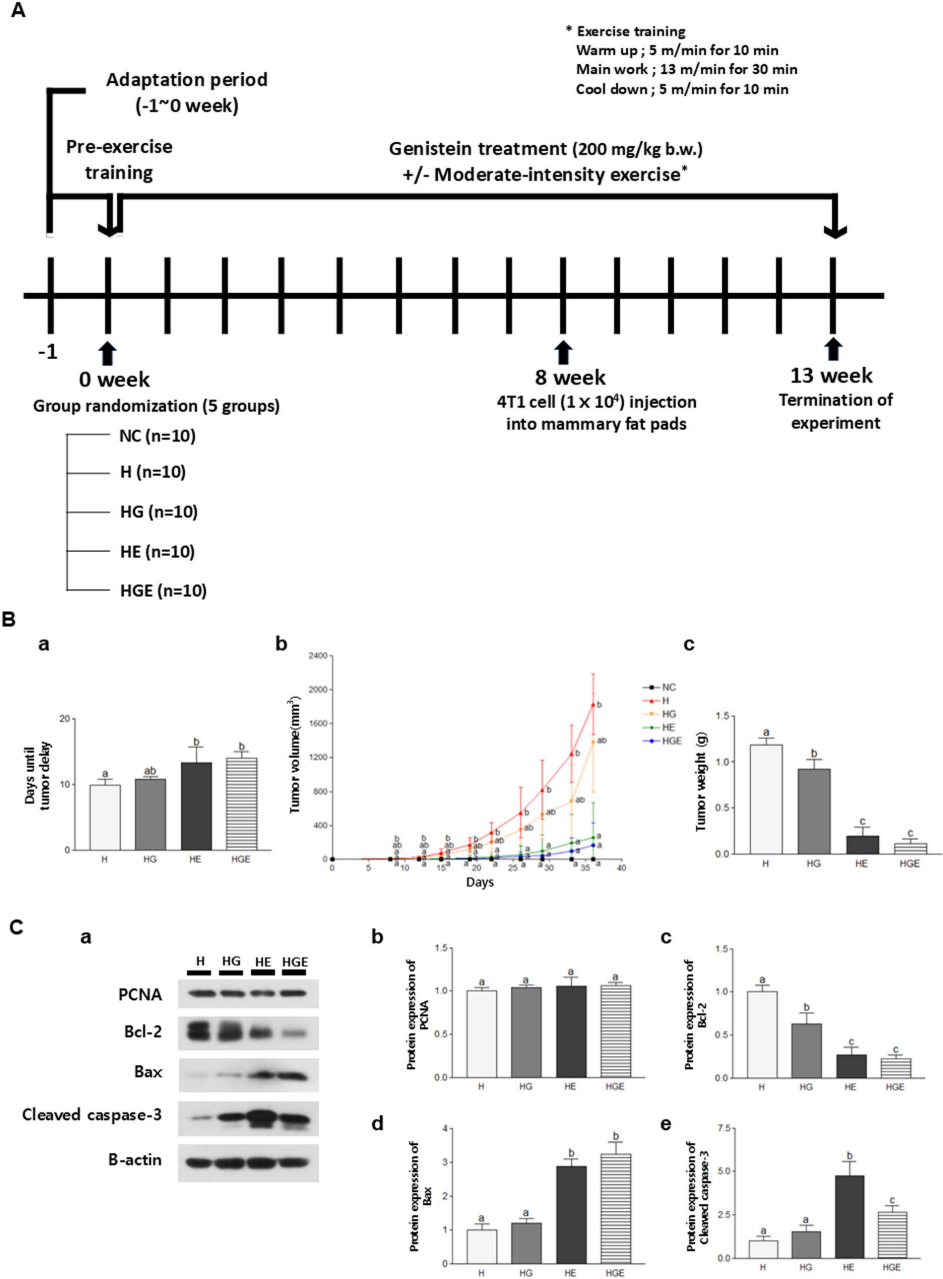
Study design and effects of GEN supplementation, moderate-intensity exercise, or their combination on the regulation of breast tumor growth and apoptosis markers in high-fat diet-induced breast cancer mice. (**A**) Study design. (**B**) (**a**) Tumor delay, (**b**) tumor volumes, and (**c**) tumor weight were measured. (**C**) (**a**) Representative blots for the protein expression of tumor cell proliferation and apoptosis markers, (**b**) PCNA, (**c**) Bcl-2, (**d**) Bax, and (**e**) cleaved caspase-3, were assessed by Western blot assay in breast tumor tissues. All data were presented as mean ± standard error of the mean (SEM) and subjected to one-way analysis of variance (ANOVA) followed by the Newman-Keuls post hoc test. Statistical significance among groups was denoted by different alphabetical letters (*P* < 0.05). GEN, genistein; H, breast cancer-induced mice fed a high-fat diet; HG, breast cancer-induced mice fed a high-fat diet with GEN; HE, breast cancer-induced mice fed a high-fat diet with moderate-intensity exercise; HGE, breast cancer-induced mice fed a high-fat diet with GEN and moderate-intensity exercise

To investigate the anti-breast cancer effects of GEN supplementation and regular moderate-intensity exercise, breast tumor growth and delay were analyzed (Fig. 1). The average tumor formation time in the H and HG groups showed approximately 9.9 and 10.8 days after tumor cell inoculation (Fig. 1Aa). In contrast, the HE and HGE groups demonstrated an average tumor formation time of roughly 13 and 14 days, respectively. The tumor volume was significantly reduced in the HE (*P* < 0.05) and HGE (*P* < 0.05) groups compared to the H group (Fig. 1Ab). The final tumor weight was significantly lower in all groups compared to the H group (Fig. 1Ac). The tumor weight was decreased by 22.3% (*P* < 0.05), 84.0% (*P* < 0.001), and 89.9% (*P* < 0.001) in the HG, HE, and HGE groups, respectively, compared to the H group.

Next, the effect of GEN supplementation and moderate-intensity exercise on breast tumor proliferation and apoptosis was investigated. The protein expression of PCNA, a marker for proliferation, did not show a statistically significant difference among the groups (Fig. 1Bb). The levels of Bcl-2, an anti-apoptotic protein, were notably downregulated in the HG, HE, and HGE groups compared to the H group by 37.5% (*P* < 0.01), 72.8% (*P* < 0.001), and 77.8% (*P* < 0.001), respectively (Fig. 1Bc). Conversely, the expression of Bax, a pro-apoptotic protein, was significantly upregulated in the HE (*P* < 0.001) and HGE (*P* < 0.001) groups compared to the H group (Fig. 1Bd). In addition, the levels of cleaved caspase-3, another pro-apoptotic protein, were significantly higher in the groups that engaged in moderate-intensity exercise compared to the H group (Fig. 1Be). These findings suggested that the combined approach of GEN supplementation and moderate-intensity exercise effectively enhanced breast tumor cell apoptosis. However, the combined treatment did not exhibit additional or synergistic effects compared to each treatment administered individually.

### Effects of GEN supplementation, moderate-intensity exercise, or their combination on TAM polarization in high-fat diet-induced breast cancer mice

**Fig. 2:**
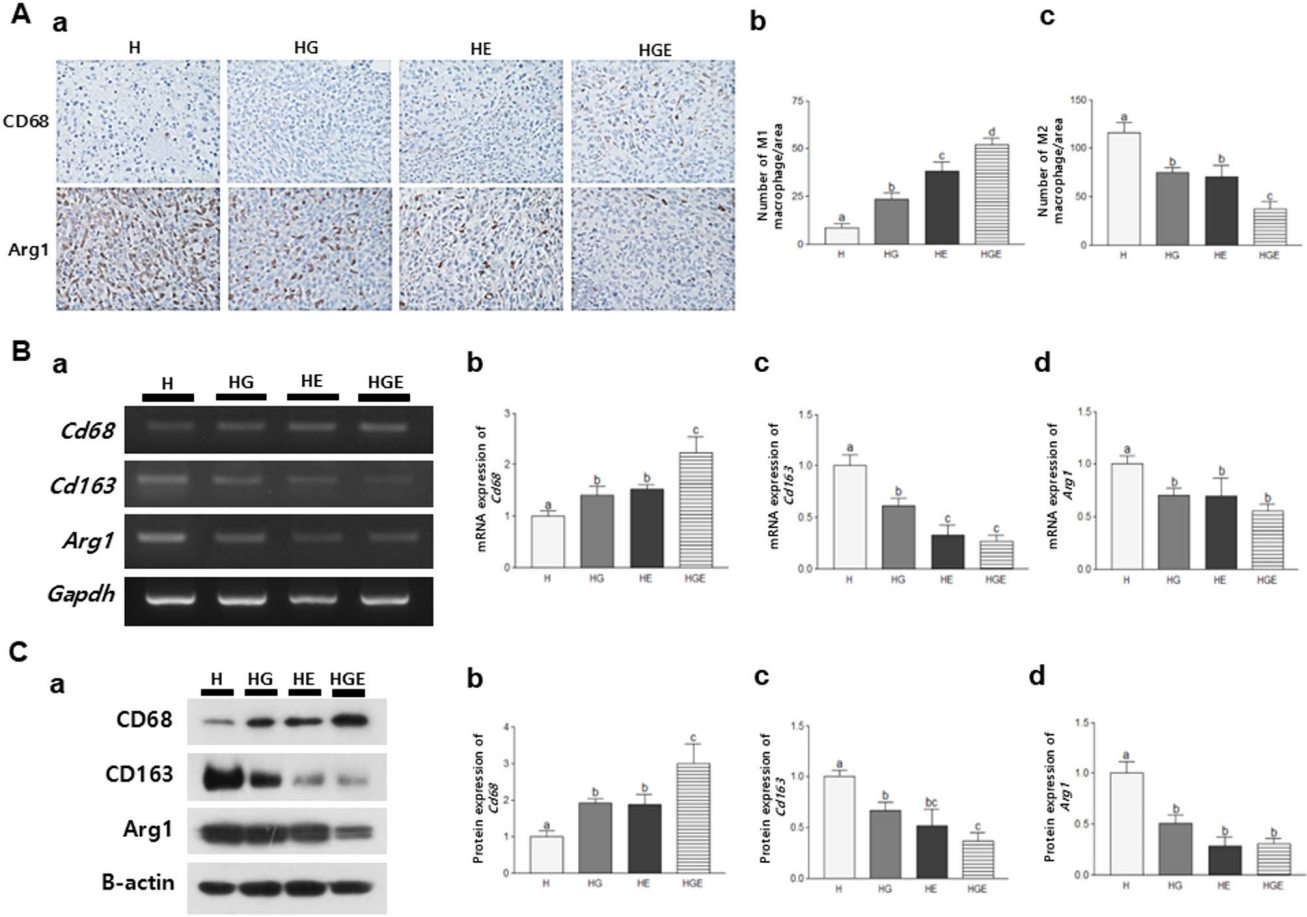
Effects of GEN supplementation, moderate-intensity exercise, or their combination on TAM polarization. (**A**) Immunohistological expression of CD68 and Arg-1 in breast tumor tissues of mice. (**a**) Representative pictures were shown at 400 × magnification. (**b**) The number of macrophages counted from the average of three immunostained photographs was shown. (**B**) (**a**) Representative pictures were shown. The mRNA expression levels of (**b**) *Cd68*, (**c**) *Cd163*, and (**d**) *Arg1* were assessed by RT-PCR. (**C**) (**a**) The protein expression levels of (**b**) CD68, (**c**) CD163, and (**d**) Arg1 were assessed by Western blot assay. All data were presented as mean ± standard error of the mean (SEM) and subjected to one-way analysis of variance (ANOVA) followed by the Newman-Keuls post hoc test. Statistical significance among groups was denoted by different alphabetical letters (*P* < 0.05). GEN, genistein; H, breast cancer-induced mice fed a high-fat diet; HG, breast cancer-induced mice fed a high-fat diet with GEN; HE, breast cancer-induced mice fed a high-fat diet with moderate-intensity exercise; HGE, breast cancer-induced mice fed a high-fat diet with GEN and moderate-intensity exercise; TAM, tumor-associated macrophage

Since suppressing M2 polarization presented potential anticancer effects, TAM polarization in breast tumor tissues by treating GEN and moderate-intensity exercise was determined (Fig. 2). An immunohistochemical analysis was conducted to assess the expression levels of CD68, a marker for the M1 phenotype, and Arg-1, a marker associated with the M2 phenotype. The results revealed a significant increase in the number of CD68-positive cells in the HG (*P* < 0.05), HE (*P* < 0.01), and HGE (*P* < 0.001) groups compared to the H group (Fig. 2Ab). In contrast, the number of Arg-1-positive cells was observed to be lower in the HG (*P* < 0.05), HE (*P* < 0.05), and HGE (*P* < 0.01) groups than in the H group (Fig. 2Ac). The combination group showed the most effectiveness compared to each treatment alone.

The mRNA expression levels of the M1 phenotype (*Cd68*) were significantly upregulated in all experimental groups compared with the H group (Fig. 2Bb). Notably, the HGE group exhibited the highest expression level, showing a substantial increase compared with the H group (*P* < 0.001). Conversely, all the treatments significantly suppressed the expression of M2-related genes, *Cd163* and *Arg1* when compared with the H group. Particularly, the HGE group demonstrated marked reductions of 73.32% (*P* < 0.01) and 44.95% (*P* < 0.05) for *Cd163* and *Arg1*, respectively (Fig. 2Bc and 2Bd). In addition, the protein expression levels of CD68 in all treatment groups were compared to the H group. The most significant upregulation by 198.5% (*P* < 0.001) was observed in the group that received the combination of GEN and exercise. Consistent with mRNA expression, the protein expression of CD163 and Arg1 were significantly reduced in the GEN and moderate-intensity exercise treatment groups compared with the H group. This reduction was most pronounced in the HGE group, with inhibition rates of 63.24% (*P* < 0.01) for CD163 and 69.70% (*P* < 0.01) for Arg1, respectively (Fig. 2Cc and 2Cd). Collectively, these data indicated that the concurrent administration of GEN supplementation and regular moderate-intensity exercise promotes the polarization of M1 macrophages while suppressing it in breast tumors.

### Effects of GEN, myokines, or their combination on M2 macrophage polarization and the JAK1/STAT6 pathway in U937 cells

**Fig. 3:**
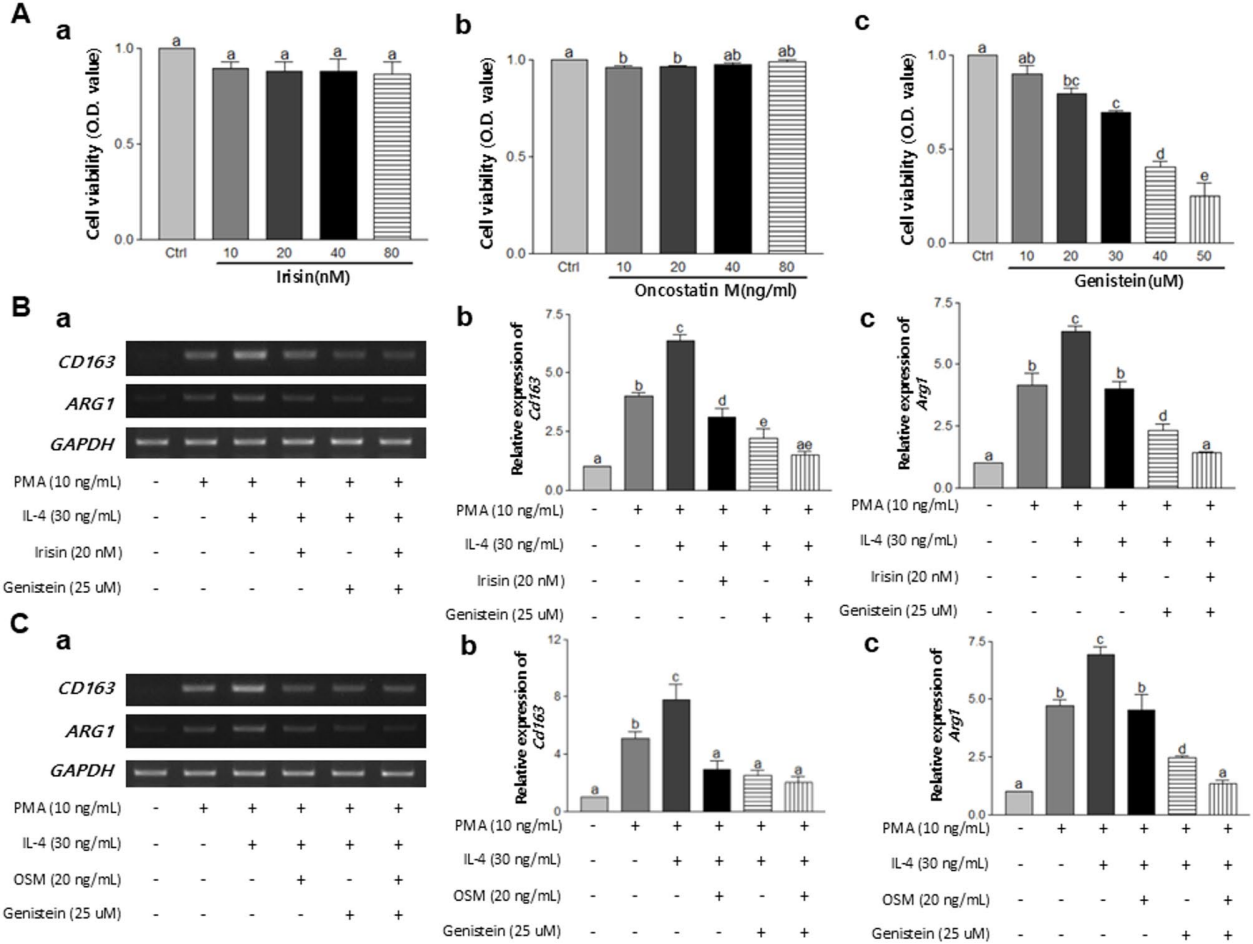
Effects of GEN, myokines, or their combination on M2 macrophage polarization in U937 cells. (**A**) U937 cells were applied with various concentrations of (**a**) Iri (10, 20, 40, and 80 nM), (**b**) OSM (10, 20, 40, and 80 ng/mL), and (**c**) Gen (10, 20, 30, 40, and 50 µM) for 96 h. (**B**) U937 cells were treated with GEN, Iri (20 nM), or their combination after PMA and IL-4 treatment. (**C**) U937 cells were treated with GEN, OSM (20 ng/mL), or their combination after PMA and IL-4 treatment. (**a**) Representative pictures were shown. mRNA gene expressions of (**b**) *CD163* and (**c**) *ARG1* were assessed by RT-PCR. All data are presented as the mean ± standard error of the mean (SEM) from at least three independent experiments. Statistical analysis was performed using one-way analysis of variance (ANOVA), followed by the Newman-Keuls post hoc test. Statistical significance among groups was denoted by different alphabetical letters (*p* < 0.05). Ctrl, Control; OSM, Oncostatin M; PMA, phorbol-12-myristate-13-acetate; IL-4, interleukin-4

To determine the effect of myokines, Iri or OSM combined with GEN on the suppression of M2 macrophage polarization, the U937 cell line was selected. It has shown that U937 cells resembled M2 polarized macrophages, as evidenced by the increased expression of M2 markers following PMA treatment with IL-4. Neither Iri nor OSM significantly affect cell viability (Fig. 3Aa and 3Ab). However, treatment with GEN resulted in a dose-dependent decrease in U937 cell viability (Fig. 3Ac). The treatment doses in the subsequent in vitro experiments were based on the respective median of each treatment.

After exposure to PMA, U937 cells displayed distinctive characteristics of macrophages and were polarized with IL-4 treatment. Upon treatment with myokine (Iri or Osm) and GEN, the mRNA levels of M2 macrophage phenotype markers, *Cd163* and *Arg1* were significantly downregulated compared with the IL-4-treated group (Fig. 3B and C). The combination treatment of irisin and GEN demonstrated the most effective inhibition of *Cd163* (*P* < 0.001) and *Arg1* (*P* < 0.001). Similarly, the combination treatment of OSM and GEN showed a significant inhibitory effect on *Cd163* (*P* < 0.001) and *Arg1* (*P* < 0.001).

**Fig. 4:**
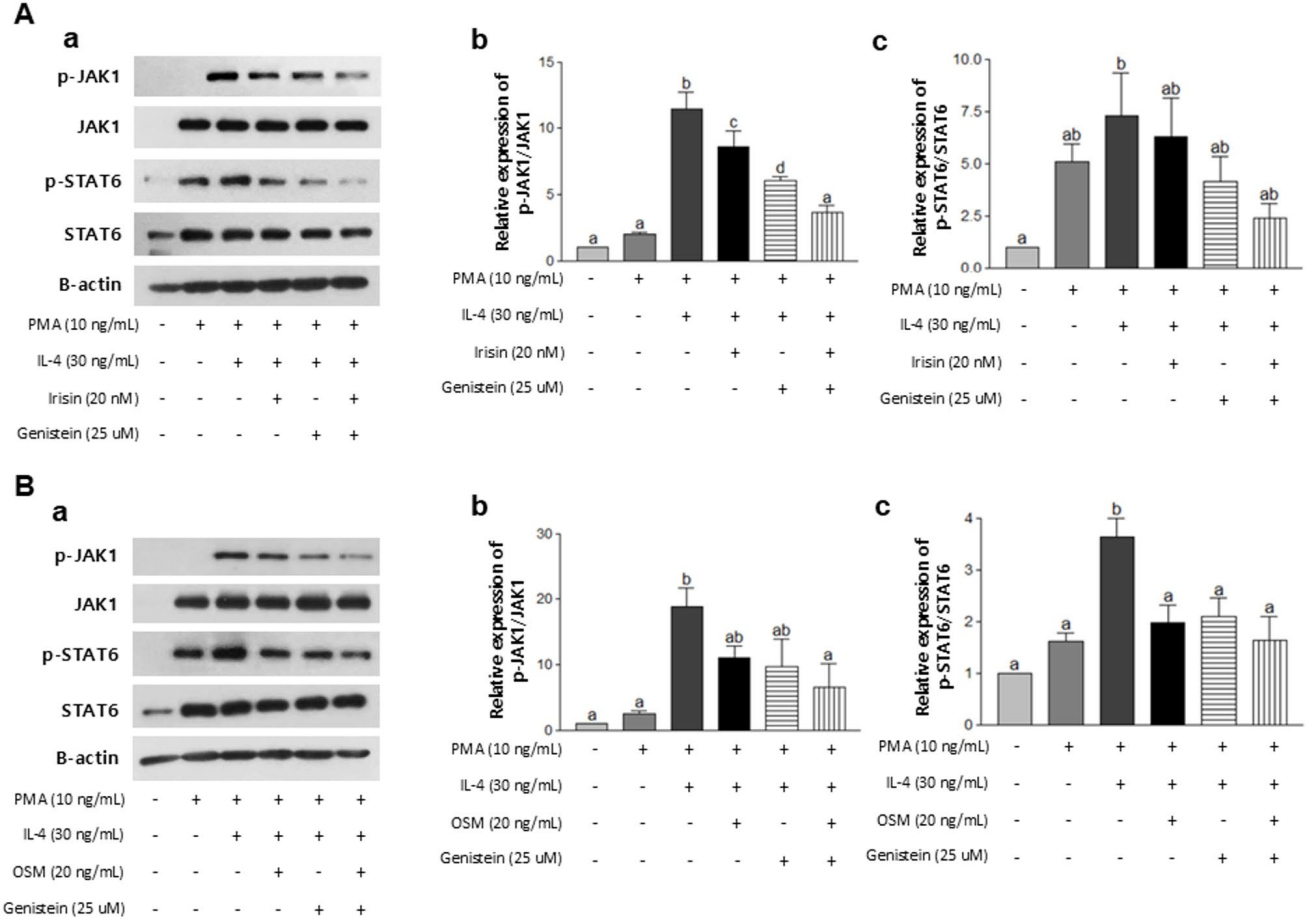
Effects of GEN, myokines, or their combination on the JAK1/STAT6 pathway in U937 cells. (**A**) U937 cells were treated with GEN, Iri (20 nM), or their combination after PMA and IL-4 treatment. (**B**) U937 cells were treated with GEN, OSM (20 ng/mL), or their combination after PMA and IL-4 treatment. (**a**) Representative pictures were shown. Protein expressions of (**b**) p-JAK1/JAK1 and (**c**) p-STAT6/STAT6 were assessed by Western blot assay. All data are presented as the mean ± standard error of the mean (SEM) from at least three independent experiments. Statistical analysis was performed using one-way analysis of variance (ANOVA), followed by the Newman-Keuls post hoc test. Statistical significance among groups was denoted by different alphabetical letters (*P* < 0.05). GEN, genistein; H, breast cancer-induced mice fed a high-fat diet; HG, breast cancer-induced mice fed a high-fat diet with GEN; HE, breast cancer-induced mice fed a high-fat diet with moderate-intensity exercise; HGE, breast cancer-induced mice fed a high-fat diet with GEN and moderate-intensity exercise

The activation of the JAK1/STAT6 pathway was found to be associated with tumor progression and M2 polarization. The expression levels of phosphorylation of JAK1 and STAT6 were significantly higher in the IL-4-treated group compared with the PMA-treated group (Fig. 4). Notably, this increase in expression was accompanied by a significant inhibition of phosphorylation of both JAK1 and STAT6 signaling after treatment with myokine (Iri or OSM), and GEN. Taken together, these findings suggested that irisin, OSM, and GEN can impede tumor progression and shift towards M2 macrophages by suppressing the JAK1/STAT6 pathway.

### Effects of GEN supplementation, moderate-intensity exercise, or their combination on adipose tissue weight in breast cancer-induced mice

The study evaluated various body fat changes induced by cancer (Table 3). The total adipose tissue weight per body weight was significantly decreased in the H group compared to the NC group after tumor development even though mice were fed a high-fat diet (*P* < 0.001). However, the adipose tissue weight was significantly restored in the HG (*P* < 0.001), HE (*P* < 0.001), and HGE (*P* < 0.001) groups compared to the H group. The study found that both the HE and HGE groups had significant fat recovery compared to the H group in terms of subcutaneous adipose tissue per body weight and visceral adipose tissue per body weight. Among the visceral adipose tissues, the weight of periovarian (*P* < 0.05 for both HE and HGE) and mesenteric adipose tissue (*P* < 0.05 for both HE and HGE) per body weight exhibited a significant fat recovery comparison to the H group. Additionally, the weight of perirenal adipose tissue per body weight was significantly recovered in the HGE group compared to the H group (*P* < 0.05). Taken together, these data suggest that interventions GEN supplementation, regular moderate-intensity exercise, or a combination of both were effective in inhibiting adipose tissue reduction induced by cancer.

**Table 3:** Effects of GEN supplementation, moderate-intensity exercise, or their combination on adipose tissue weight

	NC	H	HG	HE	HGE
Total adipose tissues (g)	0.59 ± 0.03^a^	0.39 ± 0.06^b^	0.56 ± 0.07^a^	0.65 ± 0.03^a^	0.61 ± 0.07^a^
Total adipose tissues / BW ratio (%)	2.77 ± 0.01^a^	1.87 ± 0.01^b^	2.93 ± 0.01^a^	3.00 ± 0.01^a^	3.19 ± 0.01^a^
Subcutaneous adipose tissues / BW ratio (%)	0.62 ± 0.01^a^	0.33 ± 0.01^b^	0.63 ± 0.01^a^	0.62 ± 0.01^a^	0.65 ± 0.0^a^
Visceral adipose tissues / BW ratio (%)	1.92 ± 0.01^a^	1.33 ± 0.01^b^	2.07 ± 0.01^a^	2.14 ± 0.01^a^	2.29 ± 0.01^a^
Periovarian adipose tissues / BW ratio (%)	0.23 ± 0.01^a^	0.10 ± 0.01^b^	0.25 ± 0.01^a^	0.25 ± 0.01^a^	0.27 ± 0.01^a^
Mesenteric adipose tissues / BW ratio (%)	1.36 ± 0.01^ab^	1.00 ± 0.01^a^	1.30 ± 0.01^ab^	1.54 ± 0.01^b^	1.57 ± 0.01^b^
Perirenal adipose tissues / BW ratio (%)	0.34 ± 0.01^ac^	0.24 ± 0.01^a^	0.53 ± 0.01^b^	0.36 ± 0.01^ac^	0.45 ± 0.01^bc^
Brown adipose tissues / BW ratio (%)	0.22 ± 0.01^a^	0.21 ± 0.01^a^	0.23 ± 0.01^a^	0.24 ± 0.01^a^	0.25 ± 0.01^a^

All data were presented as mean ± standard error of the mean (SEM) and subjected to one-way analysis of variance (ANOVA) followed by the Newman-Keuls post hoc test. Statistical significance among groups was denoted by different alphabetical letters (*P* < 0.05). GEN, genistein; NC, normal control; H, breast cancer-induced mice fed a high-fat diet; HG, breast cancer-induced mice fed a high-fat diet with GEN; HE, breast cancer-induced mice fed a high-fat diet with moderate-intensity exercise; HGE, breast cancer-induced mice fed a high-fat diet with GEN and moderate-intensity exercise

### Effects of GEN supplementation, moderate-intensity exercise, or their combination on adipogenesis, lipolysis, and pro-inflammatory cytokines in adipose tissue of breast cancer-induced mice

**Fig. 5:**
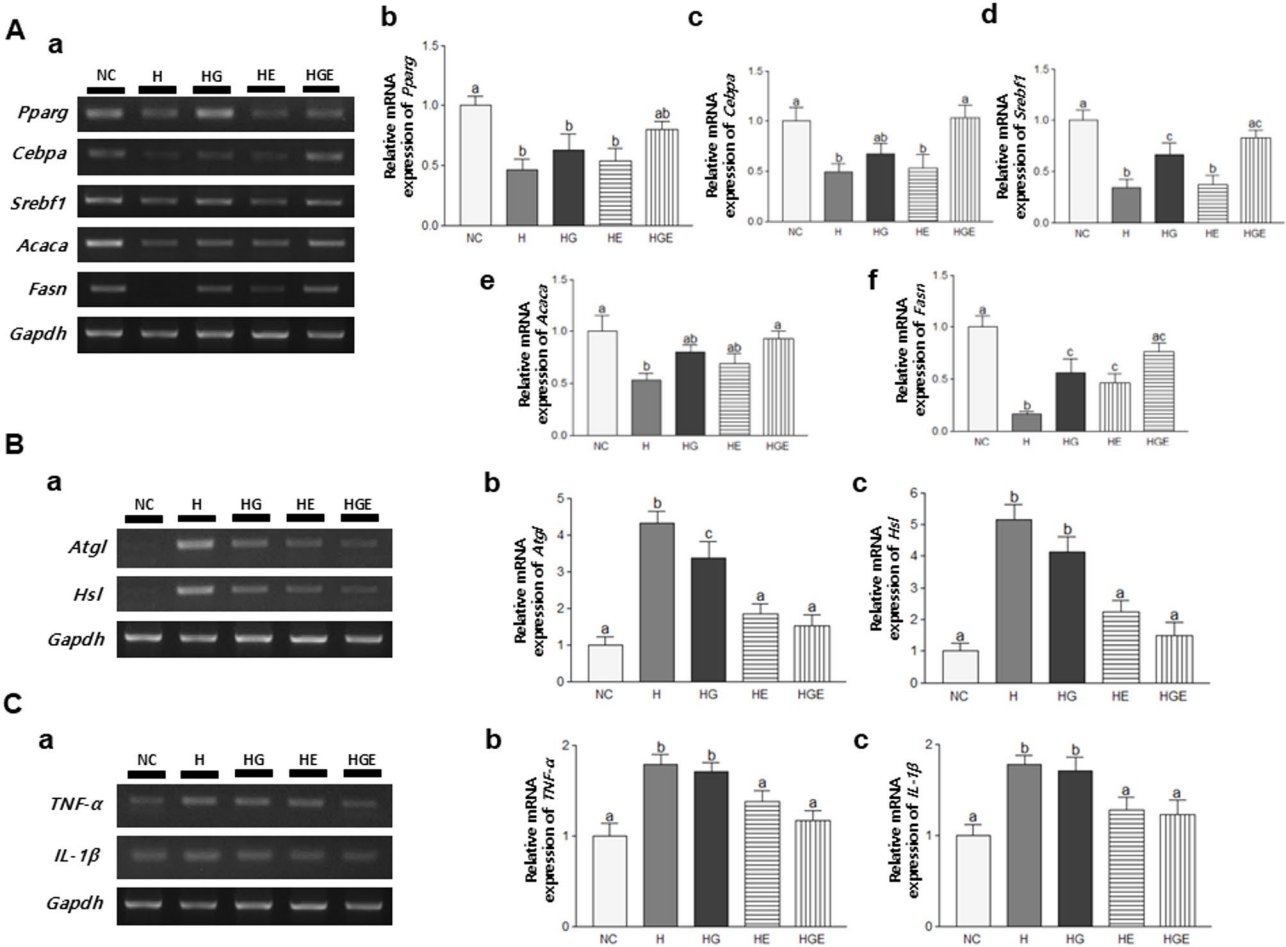
Effects of GEN supplementation, moderate-intensity exercise, or their combination on adipogenesis, lipolysis, and pro-inflammatory cytokines in adipose tissue of breast cancer-induced mice. (**A**) (**a**) Representative pictures were shown. Relative mRNA gene expressions of (**b**) *Pparg*, (**c**) *Cebpa*, (**d**) *Srebf1*, (**e**) *Acaca*, and (**f**) *Fasn* were assessed by RT-PCR. (**B**) (**a**) Representative pictures were shown. Relative mRNA gene expressions of (**b**) *Atgl* and (**c**) *Hsl* were assessed by RT-PCR. (**C**) (**a**) Representative pictures were shown. Relative mRNA gene expressions of (**b**) *TNF-α* and (**c**) *IL-1β* were assessed by RT-PCR. All data were presented as mean ± standard error of the mean (SEM) and subjected to one-way analysis of variance (ANOVA) followed by the Newman-Keuls post hoc test. Statistical significance among groups was denoted by different alphabetical letters (*P* < 0.05). GEN, genistein; H, breast cancer-induced mice fed a high-fat diet; HG, breast cancer-induced mice fed a high-fat diet with GEN; HE, breast cancer-induced mice fed a high-fat diet with moderate-intensity exercise; HGE, breast cancer-induced mice fed a high-fat diet with GEN and moderate-intensity exercise

The investigation aimed to elucidate the molecular mechanisms of adipose tissue recovery by administering GEN supplementation and moderate-intensity exercise. In this study, the mRNA expression levels of adipogenesis and lipogenesis-related markers, *Pparg*,* Cebpa*,* Srebf1*,* Acaca*, and *Fasn* in breast cancer subcutaneous adipose tissue were examined using RT-PCR (Fig. 5A). The analysis revealed a significant downregulated in the expression of genes related to adipogenesis and lipogenesis in the H group compared with the NC group (*P* < 0.05 for *Cebpa*; *P* < 0.01 for both *Pparg* and *Acaca*; *P* < 0.001 for both *Srebf1* and *Fasn*). However, all genes except *Pparg* exhibited a significant upregulation in the HGE group compared with the H group (*P* < 0.05 for both *Cebpa* and *Acaca*; *P* < 0.01 for *Srebf1*; *P* < 0.001 for *Fasn*). Consequently, the results suggested that GEN supplementation and moderate-intensity exercise might enhance adipogenesis and lipogenesis in the subcutaneous adipose tissue of 4T1 cell-induced orthotopic breast cancer mice.

To investigate observed increase in adipose tissue resulting from inhibiting fat breakdown by GEN supplementation and moderate-intensity exercise, mRNA expression levels of lipolysis-related genes, specifically *Atgl* and *Hsl* (Fig. 5B). The expression of *Atgl* and *Hsl* was significantly upregulated in the H group compared with the NC group (*P* < 0.001 for both *Atgl* and *Hsl*). However, the combination of GEN and moderate-intensity exercise suppressed genes involved in lipolysis, as evidenced by a significant downregulation of both *Atgl* and *Hsl* compared to the H group (*P* < 0.001 for both *Atgl* and *Hsl*). These findings suggested that the combination of GEN supplementation and regular moderate-intensity exercise may effectively inhibit lipolysis in subcutaneous adipose tissue, thereby preventing the loss of fat.

To understand whether GEN supplementation and moderate-intensity exercise affect inflammation, mRNA expression levels of pro-inflammatory cytokines in subcutaneous adipose tissue in this model (Fig. 5C). The expression of *TNF-α* and *IL-1β* was significantly upregulated in the H group compared to the NC group (*P* < 0.001 for *TNF-α*; *P* < 0.01 for *IL-1β*). In contrast, the expression of these genes was significantly decreased compared to the H group in both the HE group (*P* < 0.05 for both *TNF-α* and *IL-1β*) and the HGE group (*P* < 0.01 for *TNF-α*; *P* < 0.05 for *IL-1β*). These results suggested that the combination of GEN supplementation and regular moderate-intensity exercise can inhibit adipose tissue depletion by suppressing inflammation in the subcutaneous adipose tissue of 4T1 cell-induced orthotopic breast cancer mice.

## Discussion

The investigation revealed the protective impact of GEN supplementation, moderate-intensity exercise, or their combination on tumor progression, macrophage polarization, fat metabolism, and the depletion of adipose tissue using both 4T1 cell-induced orthotopic breast cancer mice and U937 human monocytic cells. The results from the in vivo mouse model demonstrated a significant reduction in both tumor growth and tumor weight following GEN supplementation and moderate-intensity exercise. Additionally, both GEN and exercise were found to modulate the polarization of macrophages, shifting the balance from pro-cancer M2-type to anti-cancer M1-type macrophages in vivo. Furthermore, the treatments exhibited protective effects against adipose tissue wasting. The underlying mechanisms responsible for the prevention of adipose tissue wasting involved the regulation of adipogenesis, lipolysis, and systemic inflammation.

The results of this study indicate that regular moderate-intensity exercise significantly suppressed tumor volume compared to the H group. Previous studies have demonstrated that consistent exercise significantly reduces serum estrogen levels, thereby decreasing the risk of postmenopausal breast cancer [45]. Moreover, physical activity has been shown to impede the progression of breast cancer by stimulating the Hippo signaling pathway, which functions as a tumor suppressor by releasing exercise-induced catecholamines [46]. Several studies have indicated that GEN supplementation was strongly associated with decreased tumor growth through the induction of apoptosis by downregulating the expression of miR155, which acted as a potent oncogene in breast cancer [47].

Previous research has explored how different levels of exercise intensity affect breast cancer progression. One study discovered that engaging in moderate-intensity exercise (10 m/min) five times weekly for an extended period before tumor inoculation had notable anti-cancer effects on both tumor growth and cell apoptosis when compared to high-intensity exercise (15 m/min with a 2.5° incline) [48]. However, the impact of exercise intensity on anti-cancer effectiveness has shown different results across studies. In an investigation, mice with 4T1 cell-induced breast cancer underwent low-intensity (6 m/min), moderate-intensity (10 m/min), and high-intensity (15 m/min) exercise for 20 consecutive days. High-intensity exercise showed the most significant effect on reducing tumor weight. And moderate-intensity exercise (10 m/min) played a critical role in the anti-cancer response by influencing apoptosis, macrophage polarization, and adipose tissue metabolism [49]. In addition, this study emphasized the significance of regular exercise consistent with previous research.

M2 macrophages play a crucial role in regulating various aspects of tumor progression, including growth, invasion, migration, and angiogenesis, thereby facilitating tumor advancement and metastasis [50]. Consequently, targeting the polarization of M2 macrophages could be a key strategy for cancer prevention. The current study revealed that a combination of GEN supplementation and moderate-intensity exercise increased the M1 macrophage marker CD68 while reducing M2 macrophage markers CD163 and Arg1 in breast cancer tissue. These findings align with prior research that has highlighted the involvement of GEN and exercise in tumor initiation, growth, and the cancer microenvironment [51, 52]. Cytokines like IL-4 or IL-13 activate JAK1, leading to STAT6 phosphorylation, which subsequently induces the transcription of M2 polarization markers such as CD163 and Arg1 [53]. Our results demonstrate that treatment with GEN and Iri or OSM attenuated JAK1 phosphorylation and STAT6 activation, thereby reducing the expression of M2 polarization markers compared to the IL-4 control. Given the crucial role of M2 macrophages in facilitating tumor growth and immune evasion, these findings suggest that the combination of GEN and moderate-intensity exercise may exert anti-cancer effects by reprogramming macrophage polarization toward a less tumor-promoting phenotype. Further studies are warranted to explore potential crosstalk between JAK/STAT and other signaling pathways, such as NF-κB or PI3K/Akt, to better understand its impact on the tumor immune environment.

The present study observed a significant inhibition of cancer-induced loss of subcutaneous and visceral adipose tissue mass by the combination of GEN and moderate-intensity exercise, suggesting that these treatments have the potential to prevent lipoatrophy caused by early cancer symptoms. Significant subcutaneous adipose tissue (SAT) alterations among various types of white adipose tissue (WAT) were observed. Previous research has indicated a strong correlation between cancer cachexia and SAT loss. In patients with gastric cancer cachexia, the reduction in SAT was more significant than visceral adipose tissue loss, and lower SAT levels were linked to unfavorable survival outcomes [54]. Moreover, changes in SAT were associated with modifications in adipose tissue-derived factors, suggesting their potential as biomarkers in individuals with cancer cachexia [55].

Previous studies have highlighted the significance of adipose tissue in cancer-related metabolic processes [56]. In this study, the combination of GEN supplementation and moderate-intensity exercise regulated adipose tissue mass and metabolism in 4T1-induced breast cancer mice. This result identified that the mechanisms underlying fat loss were associated with the reduction of adipogenesis and lipolysis as well as the increase of systemic inflammation in subcutaneous fat. It is widely acknowledged that the activation of AMPK within adipose tissue impedes the differentiation of WAT cells. AMPK acts by inhibiting and phosphorylating key targets involved in fatty acid synthesis, including PPARγ, C/EBPα, and SREBP-1 C [57]. SREBP-1 C, in particular, plays a pivotal role in regulating adipogenesis by modulating the transcription of various lipogenic enzymes, such as fatty acid synthase and acetyl-CoA carboxylase [57]. This finding suggested that the concurrent administration of GEN supplementation and regular moderate-intensity exercise may potentially facilitate the regeneration of adipose tissue by upregulating the expression of genes associated with both adipogenesis and lipogenesis. However, further research is warranted to comprehensively elucidate the molecular mechanisms and related signaling pathways, particularly those involving AMPK, which may be influenced by GEN and moderate-intensity exercise in the context of breast cancer.

Recent research has suggested a correlation between cancer cachexia symptoms and increased inflammation, which contributes to the depletion of WAT [56]. This association implies inflammatory cytokines as biomarkers for diagnosing cancer cachexia. We found that the mRNA expression levels of TNF-α and IL-1β were significantly downregulated in adipocytes in the animals treated with the combination of GEN and moderate-intensity exercise. TNF-α, a prominent pro-inflammatory cytokine, is known for its role in inhibiting the differentiation of adipose progenitor cells and suppressing the expression of adipogenic transcription factors such as PPARγ and C/EBPα [58]. Additionally, Inflammatory cytokines like TNF-α have been shown to activate lipolysis in metabolic pathways within adipose tissue [59, 60]. TNF-α also influences the weight of key organs, liver and spleen, which are involved in inflammation [61]. This study noted a significant increase in relative liver and spleen weight in breast cancer-induced mice compared to the normal control group, however, it was restored to control group levels by the combination of GEN and moderate-intensity exercise. The ratio of liver weight to body weight serves as a key indicator of hepatic metabolic stress and systemic inflammation, particularly in high-fat diet-induced conditions. An increase in this ratio may reflect hepatomegaly due to lipid accumulation or inflammation, providing insights into the metabolic impact of dietary and exercise interventions in our study. Hence, the results of this study suggested that GEN supplementation and moderate-intensity exercise might regulate metabolism associated with adipogenesis, lipolysis, and inflammation, thus mitigating excessive weight loss.

This study acknowledges a potential limitation that warrants consideration. The bioactivity of GEN may be influenced by various factors, including dosage, route of administration, and individual metabolic characteristics. Its bioavailability can also vary among individuals due to differences in metabolic capacity and gut microbiota composition, which may influence its overall efficacy. The menopausal status and expression of estrogen receptors in patients could significantly impact GEN’s bioactivity [22]. Despite extensive research into GEN’s anti-cancer properties as a tyrosine kinase inhibitor, DNA methyltransferase inhibitor, and endocrine disruptor, conflicting findings have been reported in animal models with post-menopause or following ovariectomy [22, 62, 63]. Therefore, potential side effects, such as its endocrine-disrupting properties and hepatic metabolism, should be considered when evaluating its therapeutic applicability.

The present study investigated the effects of GEN supplementation on the suppression of breast cancer, with a particular focus on the outcomes observed in combination with moderate-intensity exercise. Despite the observation of synergistic effects in vitro, these results were not replicated in vivo. This discrepancy may be attributable to several factors, including variations in the optimal dose of GEN and the extent of exercise-induced saturation. A recent study reported that a higher dose of GEN (400 mg/kg) significantly suppressed breast tumor growth in an obese animal model by altering the tumor microenvironment [64]. This finding suggests that higher doses of GEN may be more effective in models of obesity or high-fat diet-induced breast cancer. Furthermore, the present study demonstrated that moderate-intensity exercise reduced breast tumor weight by approximately 84% in comparison with the high-fat diet control. This finding suggests that the combination of GEN supplementation and exercise would be challenging to observe to be synergistically suppressing breast tumor growth. As myokine concentrations in plasma have been shown to be correlated with the intensity of exercise [65], it may be possible to apply low-intensity exercise to test for synergic effects. Consequently, to ascertain the synergistic effects of GEN and exercise, it is imperative to employ higher doses of GEN and/or lower exercise intensity. Despite the lack of a clear synergistic effect in some outcomes, the translational potential of combining GEN and exercise remains promising. These findings highlight the need for further research to optimize key parameters of the intervention, including GEN dose and exercise intensity.

Translational relevance is supported by a prospective cohort study in Japan, which linked isoflavone intake to a reduced risk of breast cancer [66]. However, clinical trials are required to establish precise dosing strategies and determine the most effective exercise timing for breast cancer patients. Further investigations are necessary to elucidate the molecular mechanisms underlying the interaction between GEN supplementation and moderate-intensity exercise in breast cancer prevention and treatment. Finally, U937-derived macrophages are widely used in macrophage polarization studies. However, they exhibit differences in cytokine secretion, surface marker expression and functional properties due to their tumor-derived cell line status when compared to primary macrophages. Nevertheless, U937 cells have been reported to stably express key polarization markers and inflammatory mediators [67] and were therefore used as a model of macrophage polarization in this study. Further validation using primary macrophages is required in future studies.

## Conclusions

The administration of GEN and regular moderate-intensity physical activity, either individually or in combination, demonstrates anti-cancer properties by regulating apoptosis, macrophage polarization, and adipose tissue metabolism both in vivo and in vitro. These findings suggest a promising therapeutic potential for breast cancer prevention and treatment. Additional clinical studies are required to determine optimal dosing, exercise regimens, and their translational applicability.

## Electronic supplementary material

Below is the link to the electronic supplementary material.

Supplementary Material 1

Supplementary Material 2

Supplementary Material 3

Supplementary Material 4

Supplementary Material 5

Supplementary Material 6

Supplementary Material 7

Supplementary Material 8

Supplementary Material 9

## Data Availability

Data is provided within supplementary information files.

## Abbreviations

ANOVA: Analysis of variance
BSA: Bovine serum albumin
DMSO: Dimethyl sulfoxide
GEN: Genistein
IL: Interleukin
Iri: Irisin
MTT: 2,5-dephenyl-2 H-tetrazolium bromide
OSM: Oncostatin M
PBS: Phosphate-buffered saline
PCNA: Proliferating cell nuclear antigen
PMA: Phorbol 12-myristate 13-acetate
PVDF: Polyvinylidene difluoride
RPMI: Roswell Park Memorial Institute
RT-PCR: Reverse transcriptase-polymerase chain reaction
SAT: Subcutaneous adipose tissue
TAMs: Tumor-associated macrophages
TBS-T: Tris-buffered saline containing Tween 20
TNF-α: Tumor necrosis factor- α
WAT: White adipose tissue
