# Supplementary material for: Anti-cancer effects of genistein supplementation and moderate-intensity exercise in high-fat diet-induced breast cancer via regulation of inflammation and adipose tissue metabolism in vivo and in vitro
Source: BMC Complement Med Ther. 2025 Jul 2;25:223. doi: 10.1186/s12906-025-04968-x (PMC12225189; doi:10.1186/s12906-025-04968-x)
Supplement: Supplementary file 3 — Supplementary Material 3 [file 12906_2025_4968_MOESM3_ESM.pptx]

## Slide 1
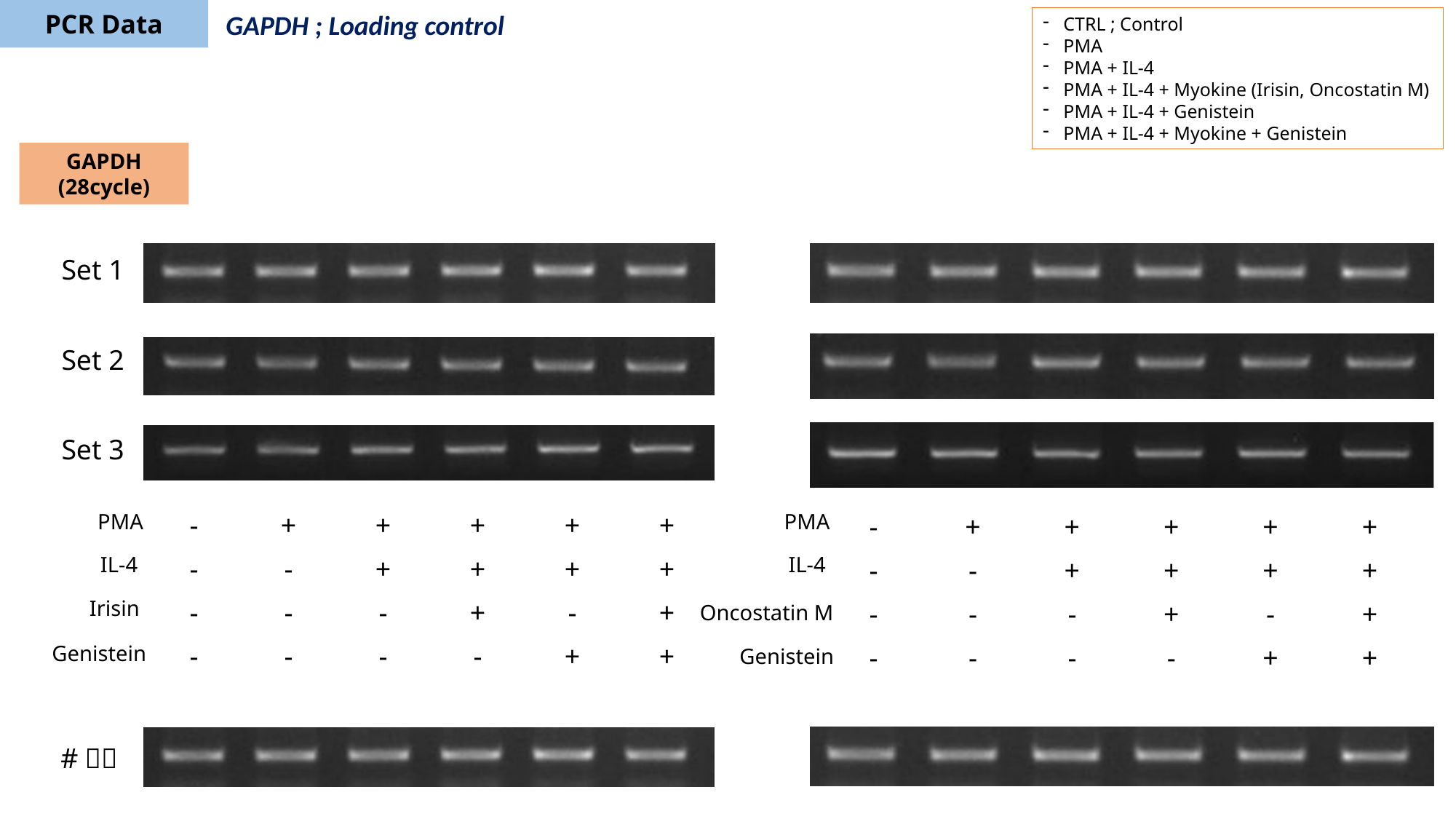

PCR Data
GAPDH ; Loading control
CTRL ; Control
PMA
PMA + IL-4
PMA + IL-4 + Myokine (Irisin, Oncostatin M)
PMA + IL-4 + Genistein
PMA + IL-4 + Myokine + Genistein
GAPDH
(28cycle)
Set 1
Set 2
Set 3
| - | + | + | + | + | + |
| --- | --- | --- | --- | --- | --- |
| - | - | + | + | + | + |
| - | - | - | + | - | + |
| - | - | - | - | + | + |
| - | + | + | + | + | + |
| --- | --- | --- | --- | --- | --- |
| - | - | + | + | + | + |
| - | - | - | + | - | + |
| - | - | - | - | + | + |
PMA
PMA
IL-4
IL-4
Irisin
Oncostatin M
Genistein
Genistein
#대표

## Slide 2
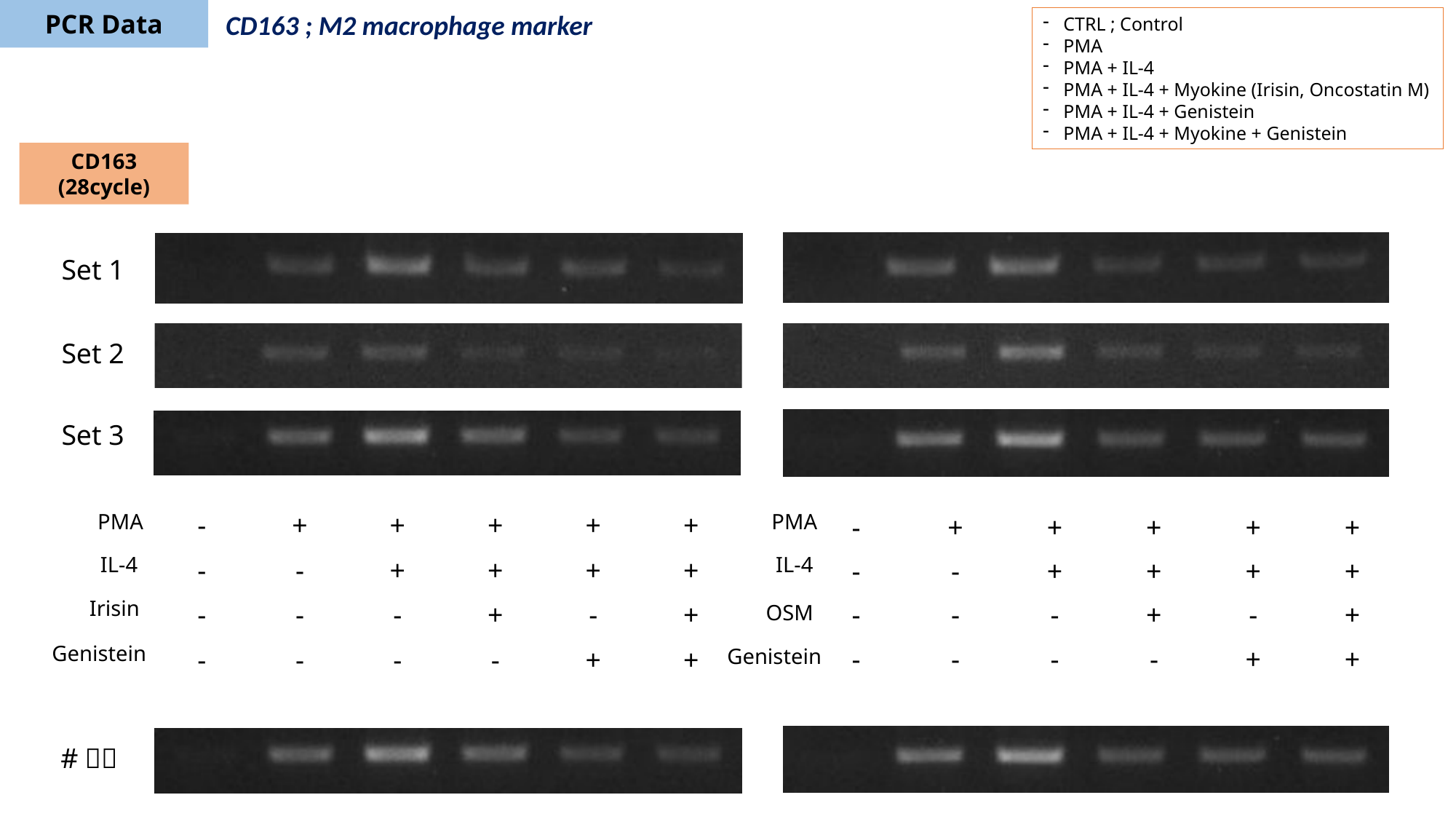

PCR Data
CD163 ; M2 macrophage marker
CTRL ; Control
PMA
PMA + IL-4
PMA + IL-4 + Myokine (Irisin, Oncostatin M)
PMA + IL-4 + Genistein
PMA + IL-4 + Myokine + Genistein
CD163
(28cycle)
Set 1
Set 2
Set 3
| - | + | + | + | + | + |
| --- | --- | --- | --- | --- | --- |
| - | - | + | + | + | + |
| - | - | - | + | - | + |
| - | - | - | - | + | + |
PMA
PMA
| - | + | + | + | + | + |
| --- | --- | --- | --- | --- | --- |
| - | - | + | + | + | + |
| - | - | - | + | - | + |
| - | - | - | - | + | + |
IL-4
IL-4
Irisin
OSM
Genistein
Genistein
#대표

## Slide 3
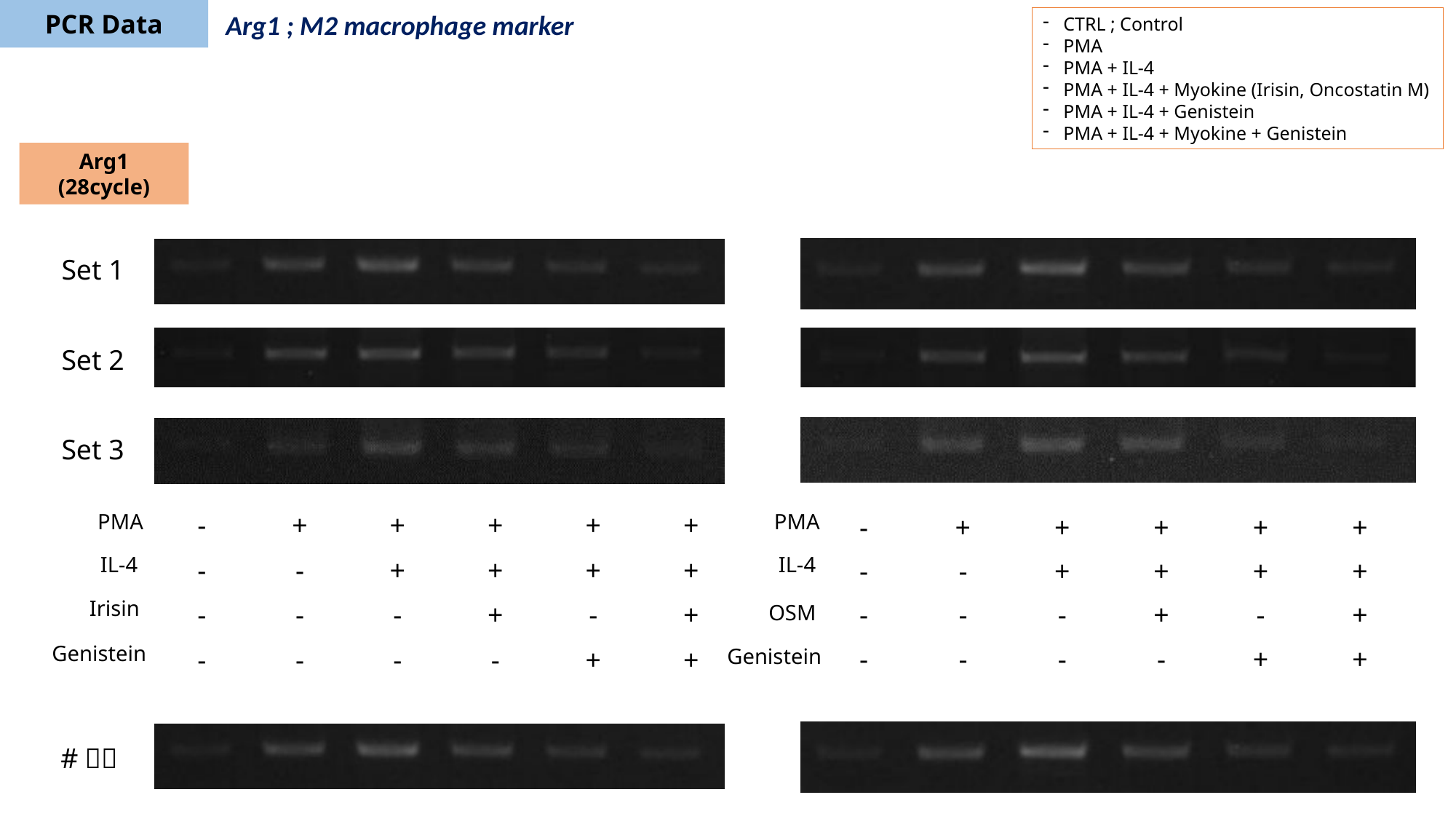

PCR Data
Arg1 ; M2 macrophage marker
CTRL ; Control
PMA
PMA + IL-4
PMA + IL-4 + Myokine (Irisin, Oncostatin M)
PMA + IL-4 + Genistein
PMA + IL-4 + Myokine + Genistein
Arg1
(28cycle)
Set 1
Set 2
Set 3
| - | + | + | + | + | + |
| --- | --- | --- | --- | --- | --- |
| - | - | + | + | + | + |
| - | - | - | + | - | + |
| - | - | - | - | + | + |
PMA
PMA
| - | + | + | + | + | + |
| --- | --- | --- | --- | --- | --- |
| - | - | + | + | + | + |
| - | - | - | + | - | + |
| - | - | - | - | + | + |
IL-4
IL-4
Irisin
OSM
Genistein
Genistein
#대표

## Slide 4
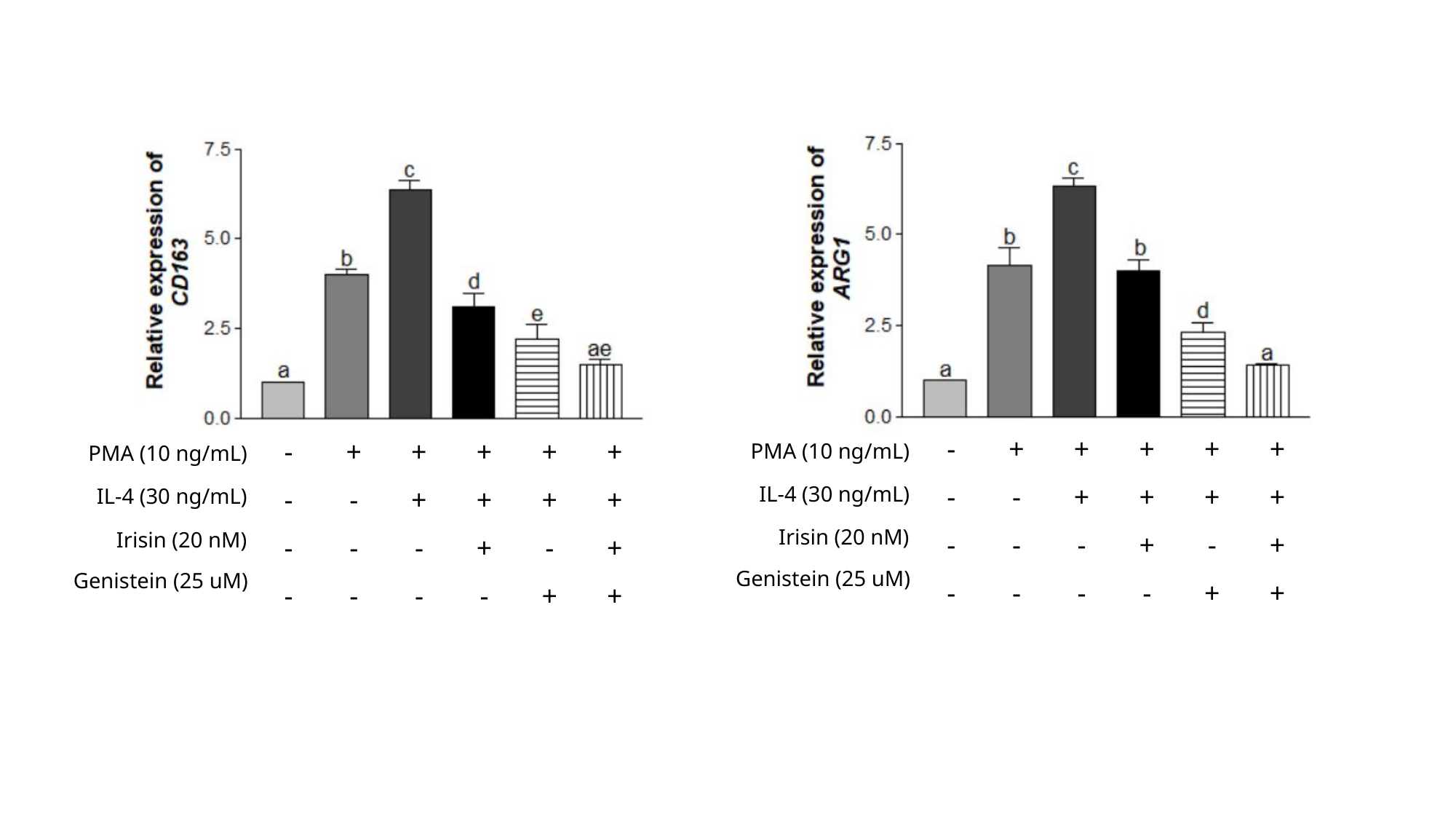

| - | + | + | + | + | + |
| --- | --- | --- | --- | --- | --- |
| - | - | + | + | + | + |
| - | - | - | + | - | + |
| - | - | - | - | + | + |
| - | + | + | + | + | + |
| --- | --- | --- | --- | --- | --- |
| - | - | + | + | + | + |
| - | - | - | + | - | + |
| - | - | - | - | + | + |
PMA (10 ng/mL)
PMA (10 ng/mL)
IL-4 (30 ng/mL)
IL-4 (30 ng/mL)
Irisin (20 nM)
Irisin (20 nM)
Genistein (25 uM)
Genistein (25 uM)

## Slide 5
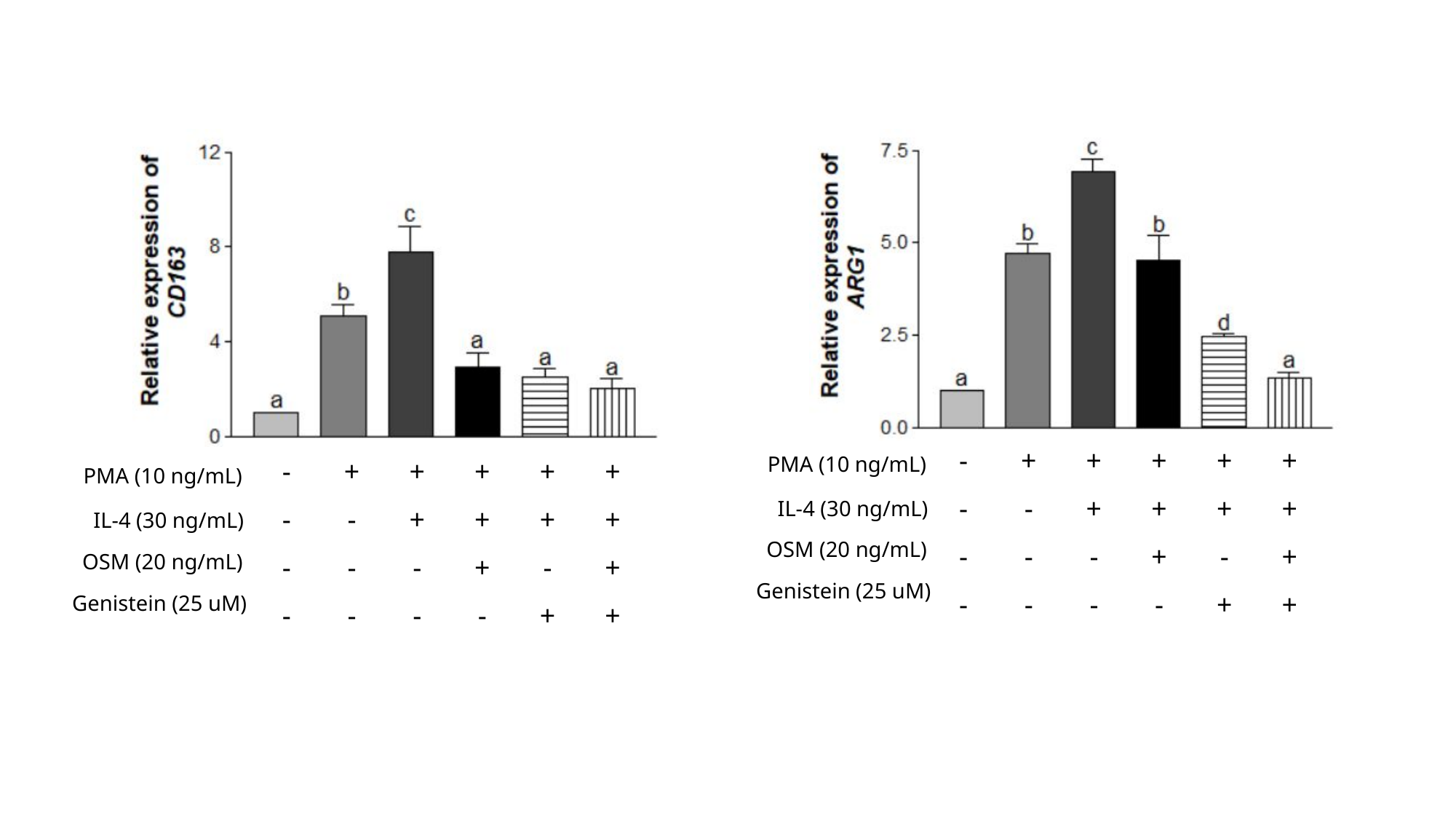

| - | + | + | + | + | + |
| --- | --- | --- | --- | --- | --- |
| - | - | + | + | + | + |
| - | - | - | + | - | + |
| - | - | - | - | + | + |
PMA (10 ng/mL)
| - | + | + | + | + | + |
| --- | --- | --- | --- | --- | --- |
| - | - | + | + | + | + |
| - | - | - | + | - | + |
| - | - | - | - | + | + |
PMA (10 ng/mL)
IL-4 (30 ng/mL)
IL-4 (30 ng/mL)
OSM (20 ng/mL)
OSM (20 ng/mL)
Genistein (25 uM)
Genistein (25 uM)

## Slide 6
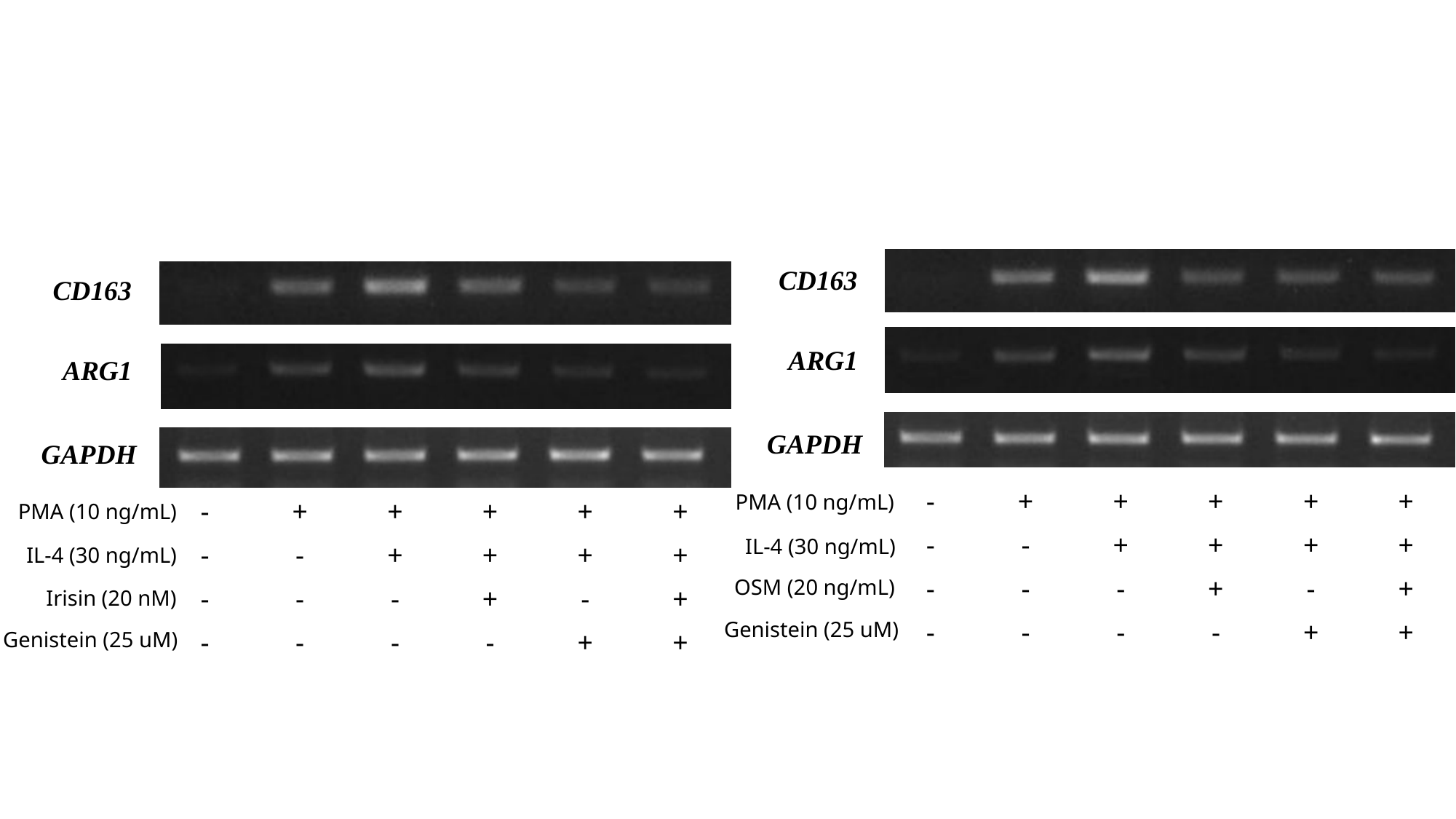

CD163
CD163
ARG1
ARG1
GAPDH
GAPDH
| - | + | + | + | + | + |
| --- | --- | --- | --- | --- | --- |
| - | - | + | + | + | + |
| - | - | - | + | - | + |
| - | - | - | - | + | + |
PMA (10 ng/mL)
| - | + | + | + | + | + |
| --- | --- | --- | --- | --- | --- |
| - | - | + | + | + | + |
| - | - | - | + | - | + |
| - | - | - | - | + | + |
PMA (10 ng/mL)
IL-4 (30 ng/mL)
IL-4 (30 ng/mL)
OSM (20 ng/mL)
Irisin (20 nM)
Genistein (25 uM)
Genistein (25 uM)
