# Supplementary material for: Anti-cancer effects of genistein supplementation and moderate-intensity exercise in high-fat diet-induced breast cancer via regulation of inflammation and adipose tissue metabolism in vivo and in vitro
Source: BMC Complement Med Ther. 2025 Jul 2;25:223. doi: 10.1186/s12906-025-04968-x (PMC12225189; doi:10.1186/s12906-025-04968-x)
Supplement: Supplementary file 4 — Supplementary Material 4 [file 12906_2025_4968_MOESM4_ESM.pptx]

## Slide 1
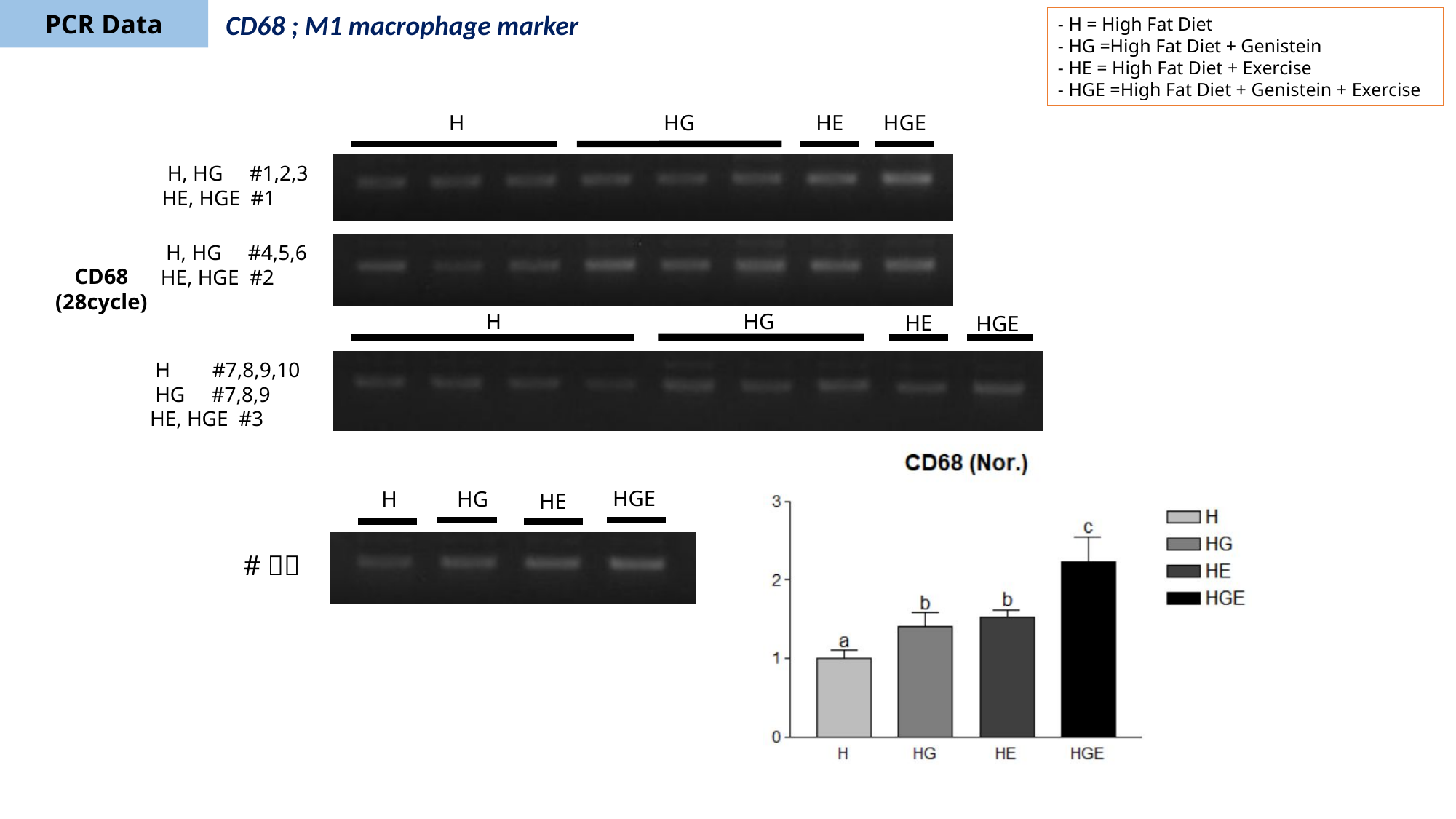

PCR Data
CD68 ; M1 macrophage marker
- H = High Fat Diet
- HG =High Fat Diet + Genistein
- HE = High Fat Diet + Exercise
- HGE =High Fat Diet + Genistein + Exercise
HGE
HE
HG
H
 H, HG #1,2,3
HE, HGE #1
 H, HG #4,5,6
HE, HGE #2
CD68
(28cycle)
H
HG
HE
HGE
 H #7,8,9,10
 HG #7,8,9
HE, HGE #3
HGE
H
HG
HE
#대표

## Slide 2
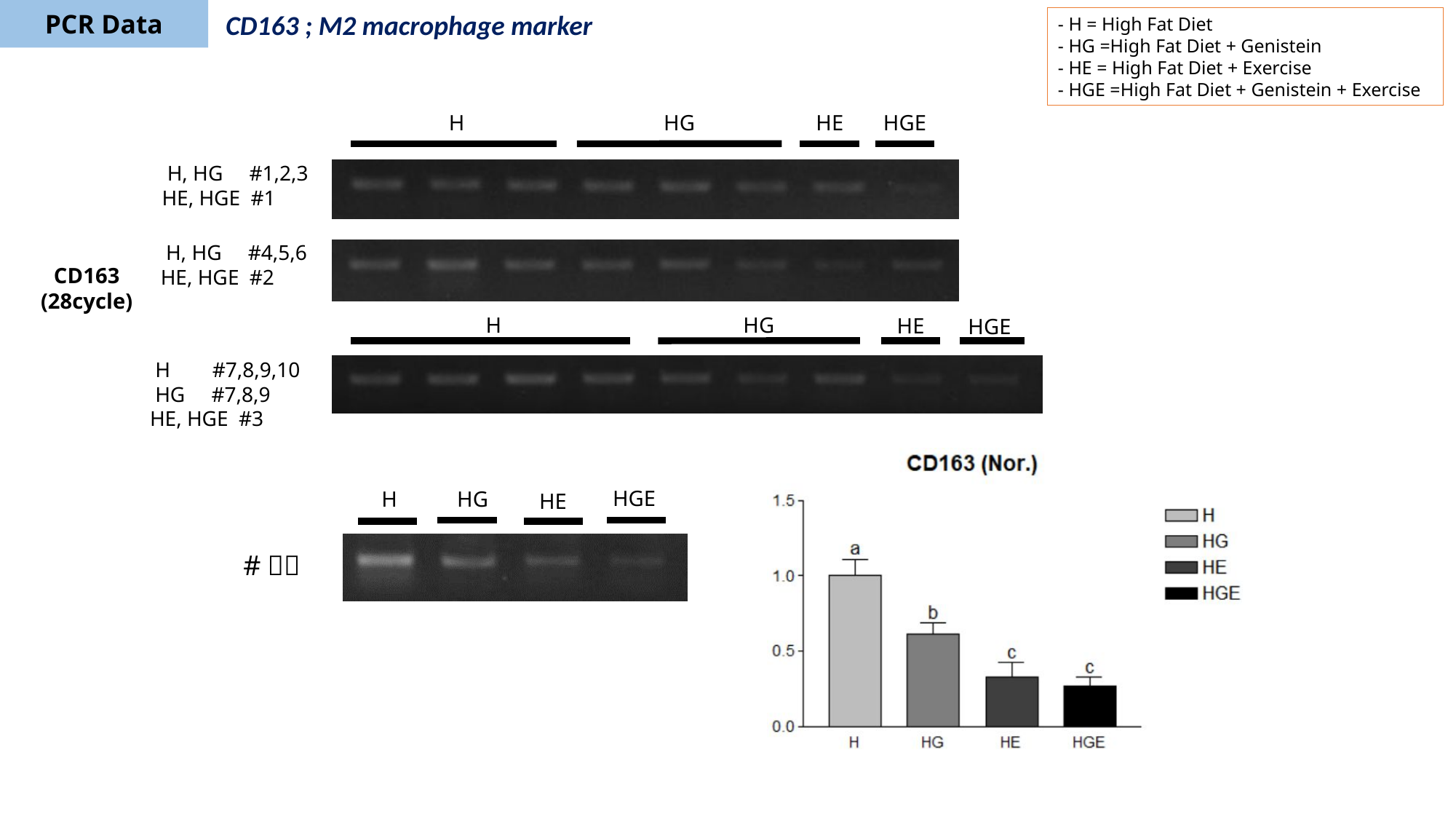

PCR Data
CD163 ; M2 macrophage marker
- H = High Fat Diet
- HG =High Fat Diet + Genistein
- HE = High Fat Diet + Exercise
- HGE =High Fat Diet + Genistein + Exercise
HGE
HE
HG
H
 H, HG #1,2,3
HE, HGE #1
 H, HG #4,5,6
HE, HGE #2
CD163
(28cycle)
H
HG
HE
HGE
 H #7,8,9,10
 HG #7,8,9
HE, HGE #3
HGE
H
HG
HE
#대표

## Slide 3
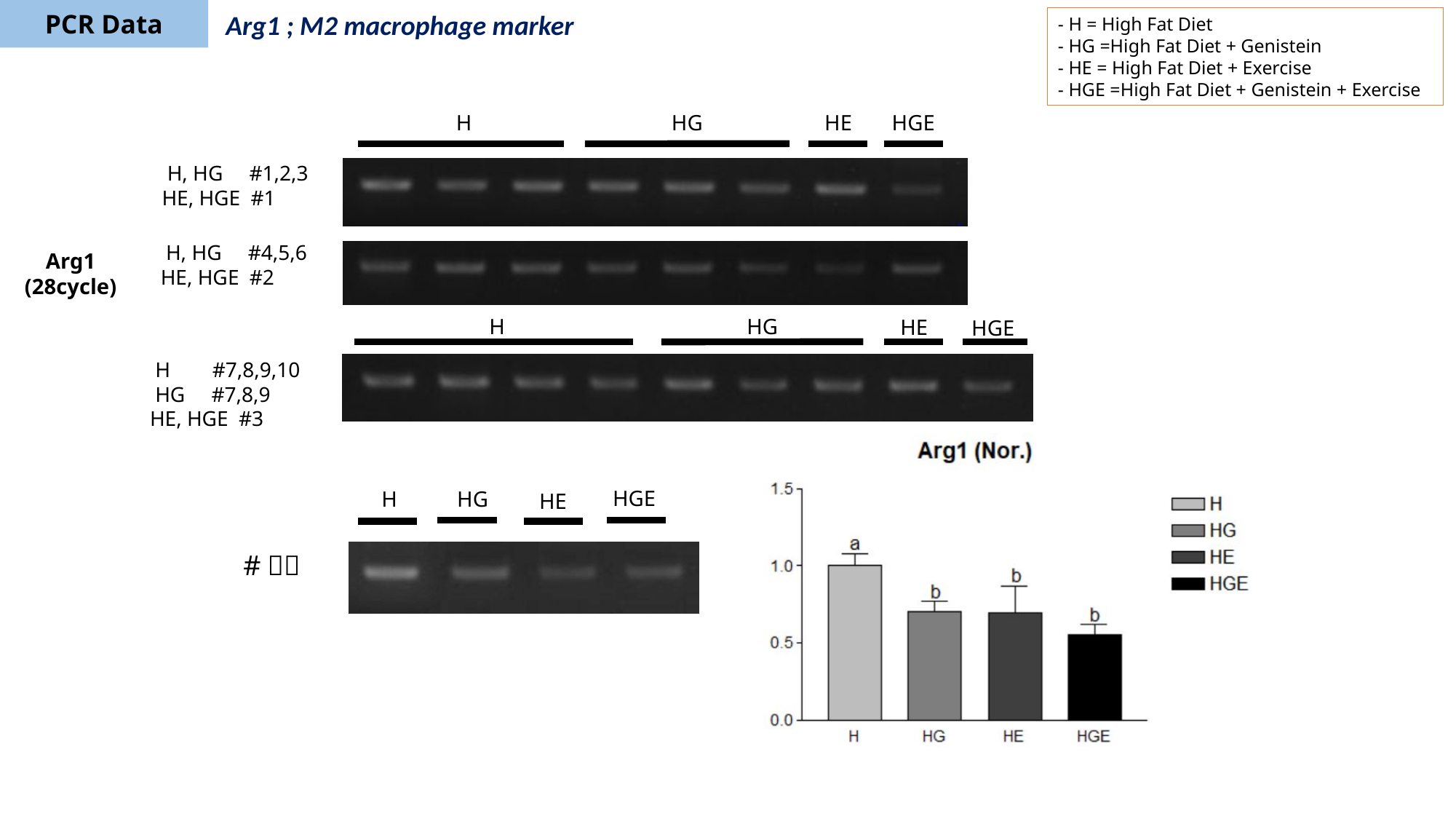

PCR Data
Arg1 ; M2 macrophage marker
- H = High Fat Diet
- HG =High Fat Diet + Genistein
- HE = High Fat Diet + Exercise
- HGE =High Fat Diet + Genistein + Exercise
HGE
HE
HG
H
 H, HG #1,2,3
HE, HGE #1
 H, HG #4,5,6
HE, HGE #2
Arg1
(28cycle)
H
HG
HE
HGE
 H #7,8,9,10
 HG #7,8,9
HE, HGE #3
HGE
H
HG
HE
#대표

## Slide 4
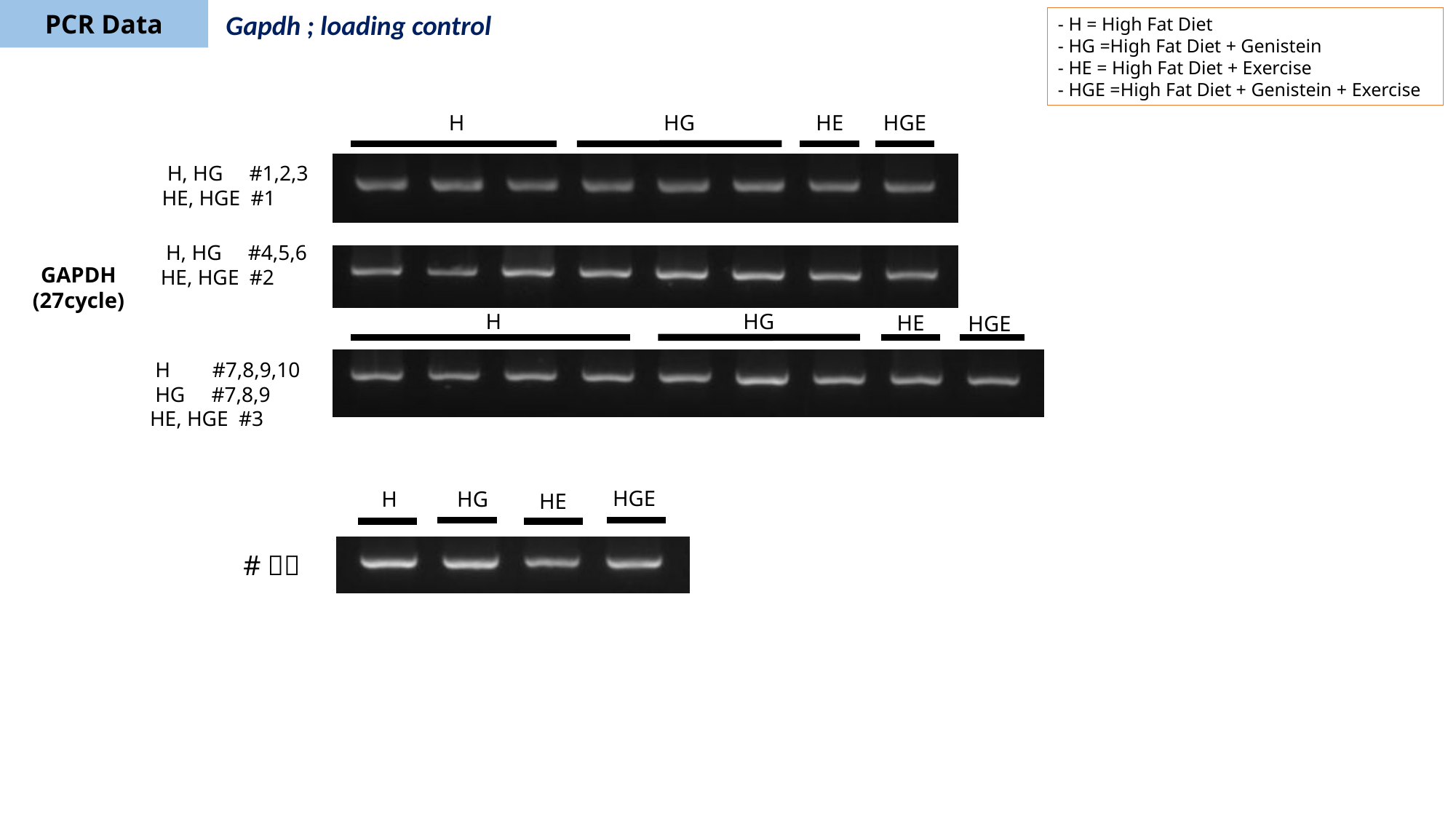

PCR Data
Gapdh ; loading control
- H = High Fat Diet
- HG =High Fat Diet + Genistein
- HE = High Fat Diet + Exercise
- HGE =High Fat Diet + Genistein + Exercise
HGE
HE
HG
H
 H, HG #1,2,3
HE, HGE #1
 H, HG #4,5,6
HE, HGE #2
GAPDH
(27cycle)
H
HG
HE
HGE
 H #7,8,9,10
 HG #7,8,9
HE, HGE #3
HGE
H
HG
HE
#대표
