# Supplementary material for: Anti-cancer effects of genistein supplementation and moderate-intensity exercise in high-fat diet-induced breast cancer via regulation of inflammation and adipose tissue metabolism in vivo and in vitro
Source: BMC Complement Med Ther. 2025 Jul 2;25:223. doi: 10.1186/s12906-025-04968-x (PMC12225189; doi:10.1186/s12906-025-04968-x)
Supplement: Supplementary file 5 — Supplementary Material 5 [file 12906_2025_4968_MOESM5_ESM.pptx]

## Slide 1
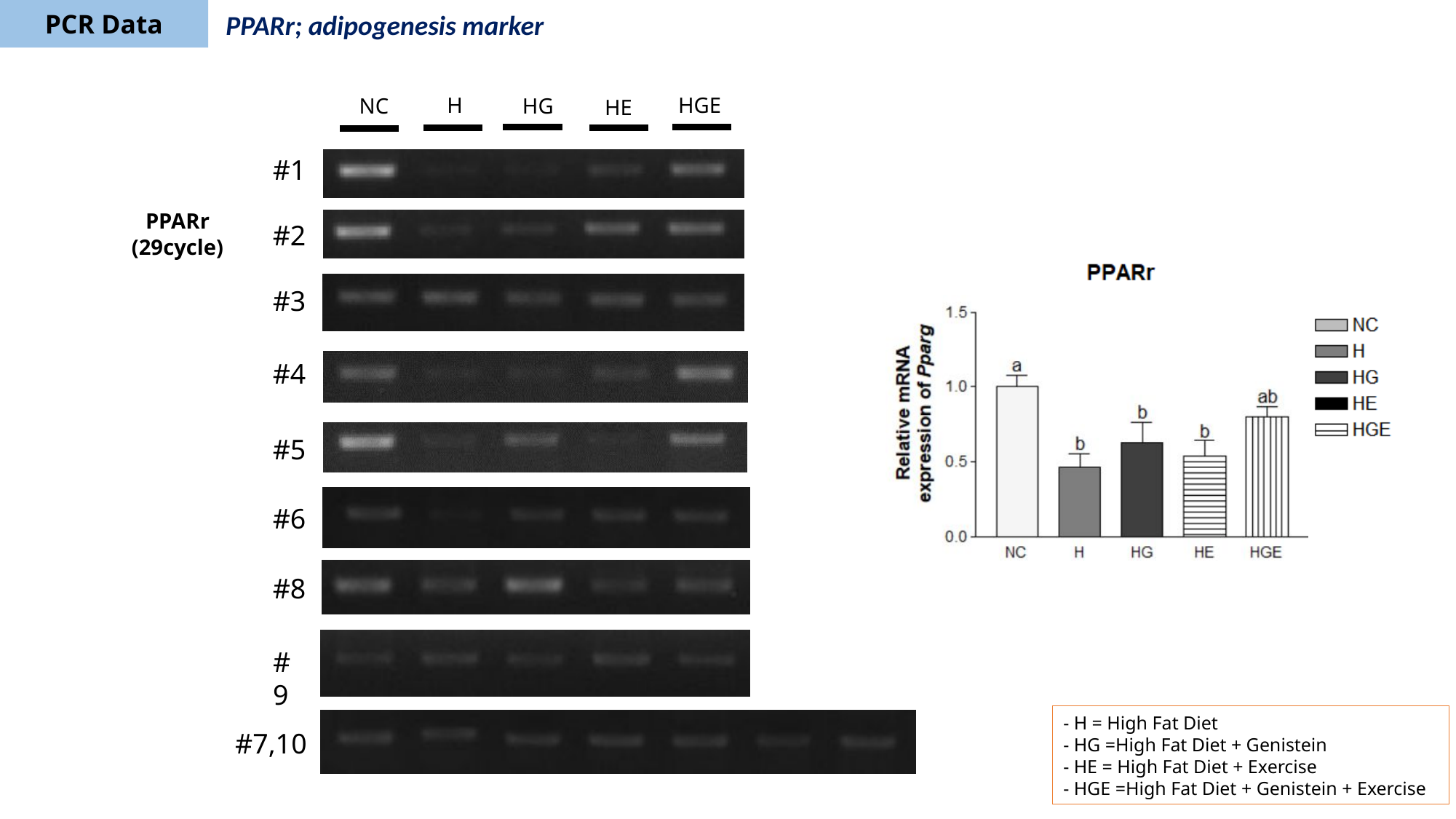

PCR Data
PPARr; adipogenesis marker
HGE
H
HG
NC
HE
#1
PPARr
(29cycle)
#2
#3
#4
#5
#6
#8
#9
- H = High Fat Diet
- HG =High Fat Diet + Genistein
- HE = High Fat Diet + Exercise
- HGE =High Fat Diet + Genistein + Exercise
#7,10

## Slide 2
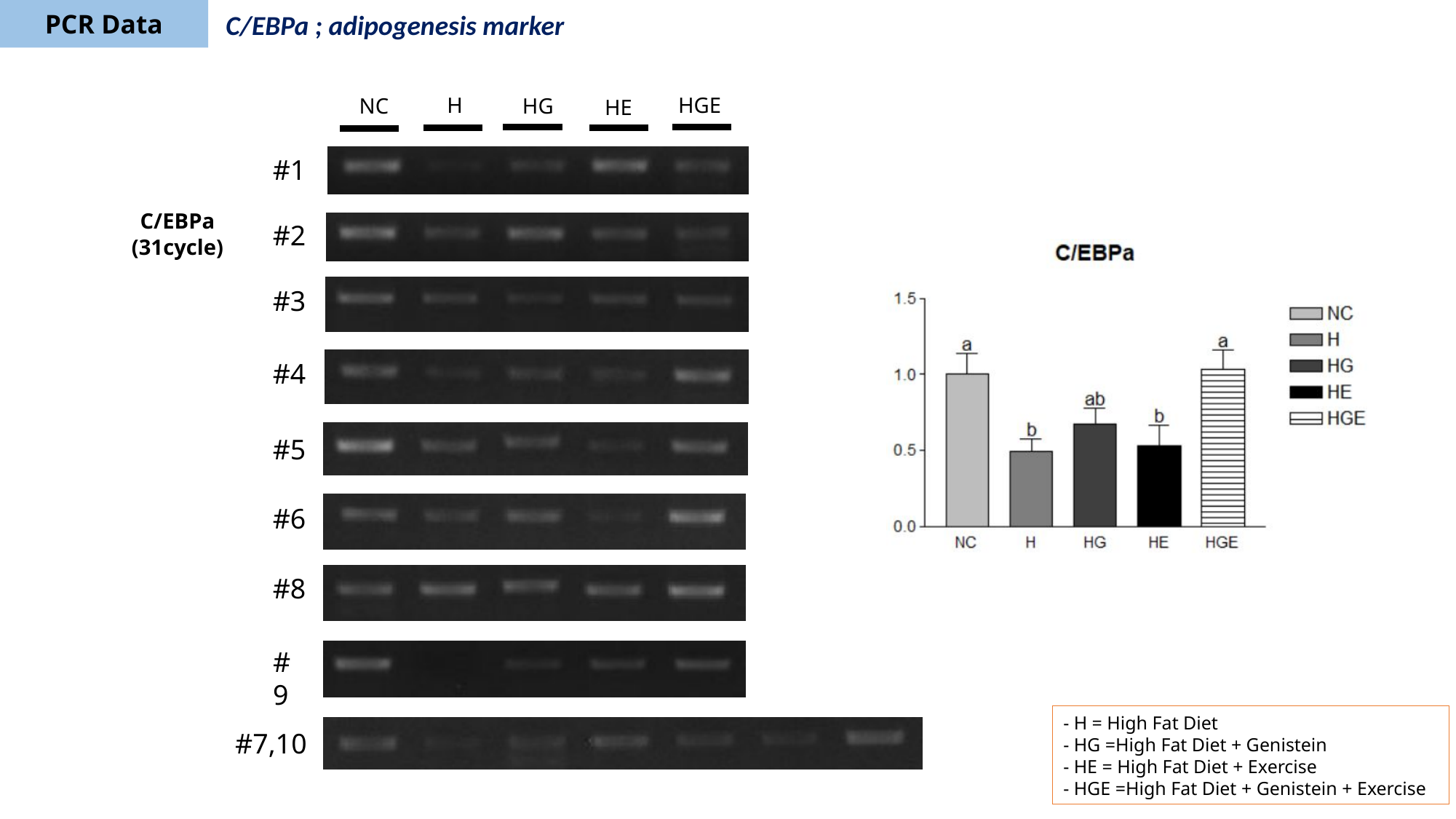

PCR Data
C/EBPa ; adipogenesis marker
HGE
H
HG
NC
HE
#1
C/EBPa
(31cycle)
#2
#3
#4
#5
#6
#8
#9
- H = High Fat Diet
- HG =High Fat Diet + Genistein
- HE = High Fat Diet + Exercise
- HGE =High Fat Diet + Genistein + Exercise
#7,10

## Slide 3
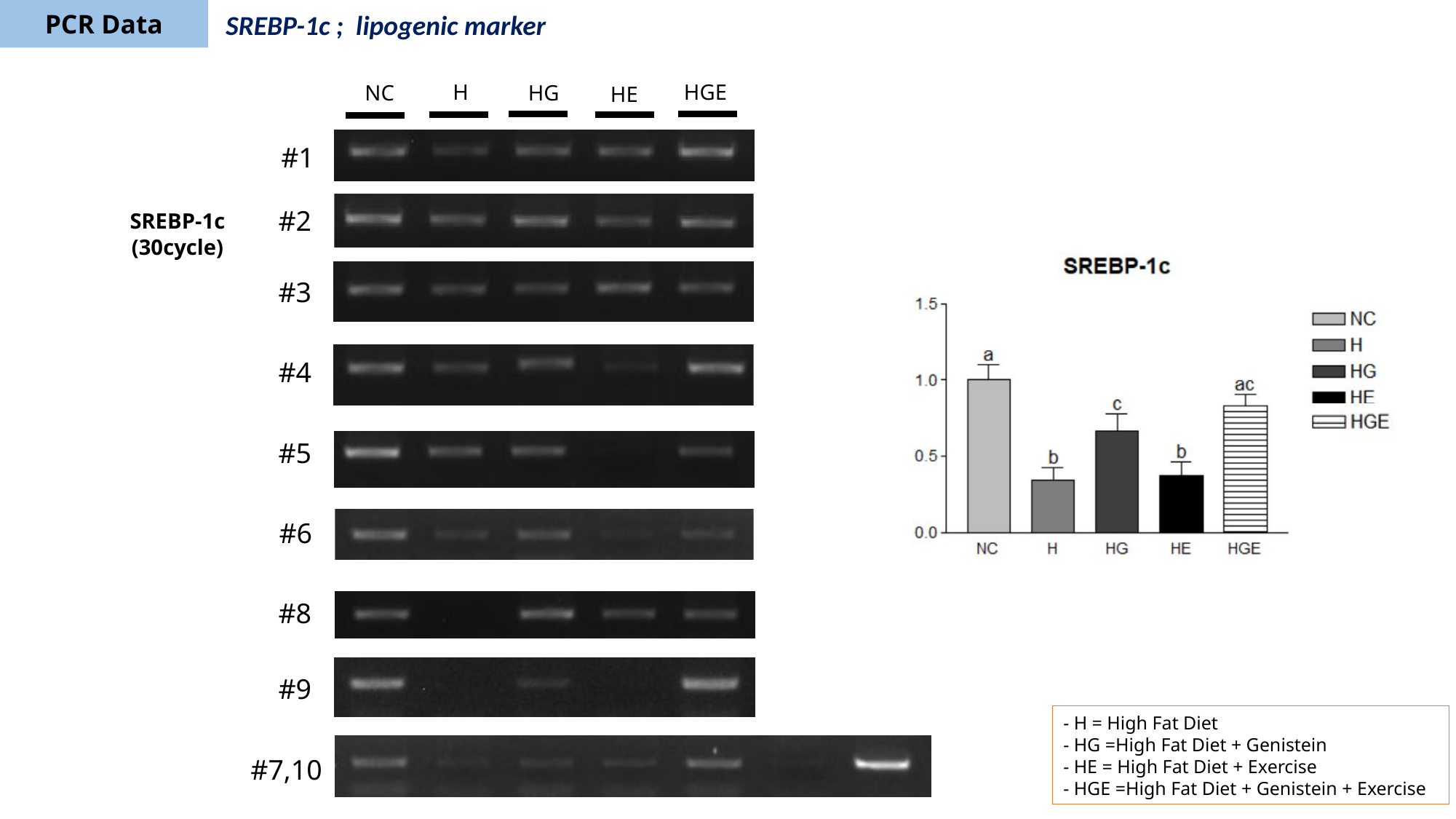

PCR Data
SREBP-1c ; lipogenic marker
HGE
H
HG
NC
HE
#1
#2
SREBP-1c
(30cycle)
#3
#4
#5
#6
#8
#9
- H = High Fat Diet
- HG =High Fat Diet + Genistein
- HE = High Fat Diet + Exercise
- HGE =High Fat Diet + Genistein + Exercise
#7,10

## Slide 4
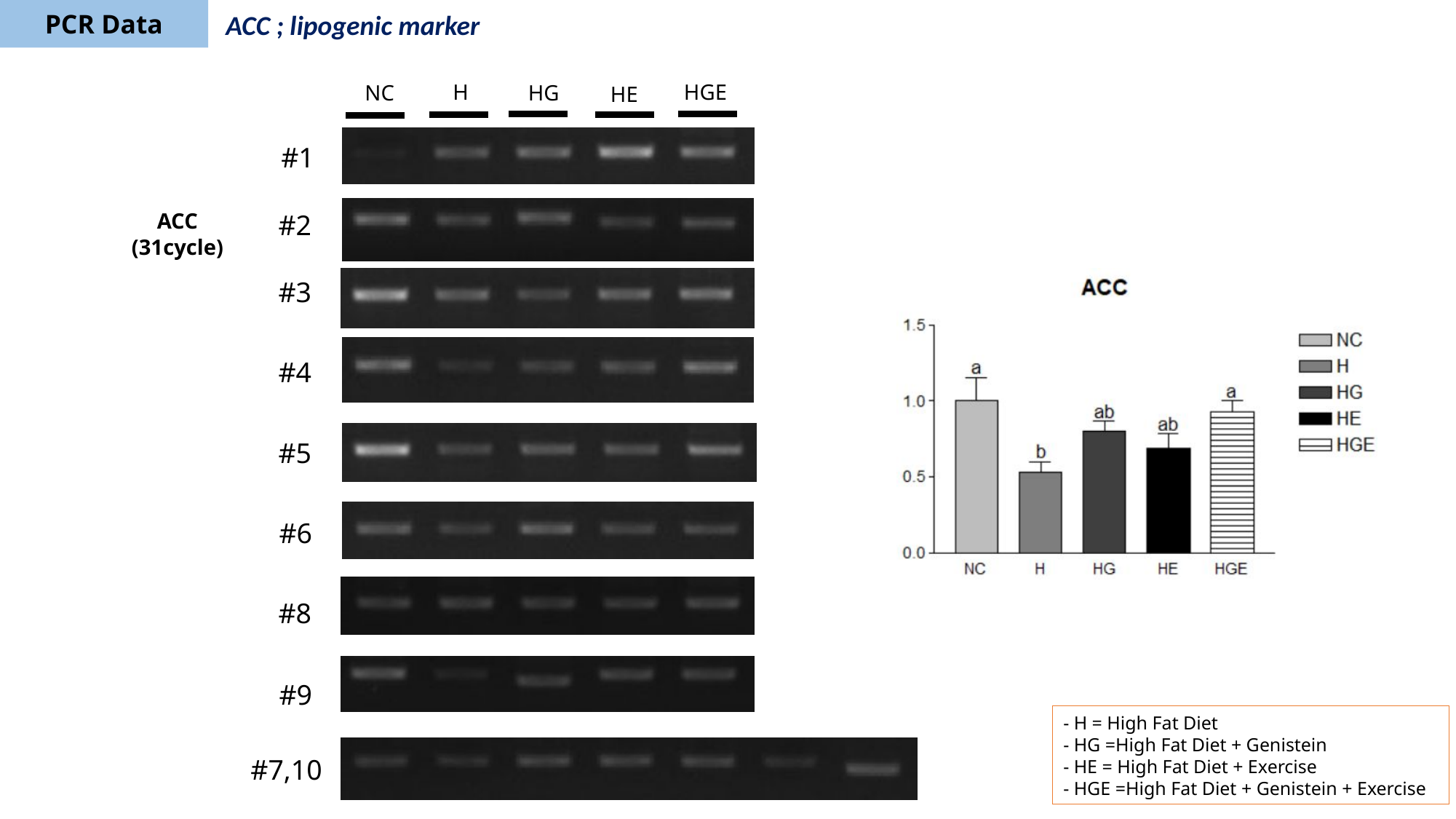

PCR Data
ACC ; lipogenic marker
HGE
H
HG
NC
HE
#1
ACC
(31cycle)
#2
#3
#4
#5
#6
#8
#9
- H = High Fat Diet
- HG =High Fat Diet + Genistein
- HE = High Fat Diet + Exercise
- HGE =High Fat Diet + Genistein + Exercise
#7,10

## Slide 5
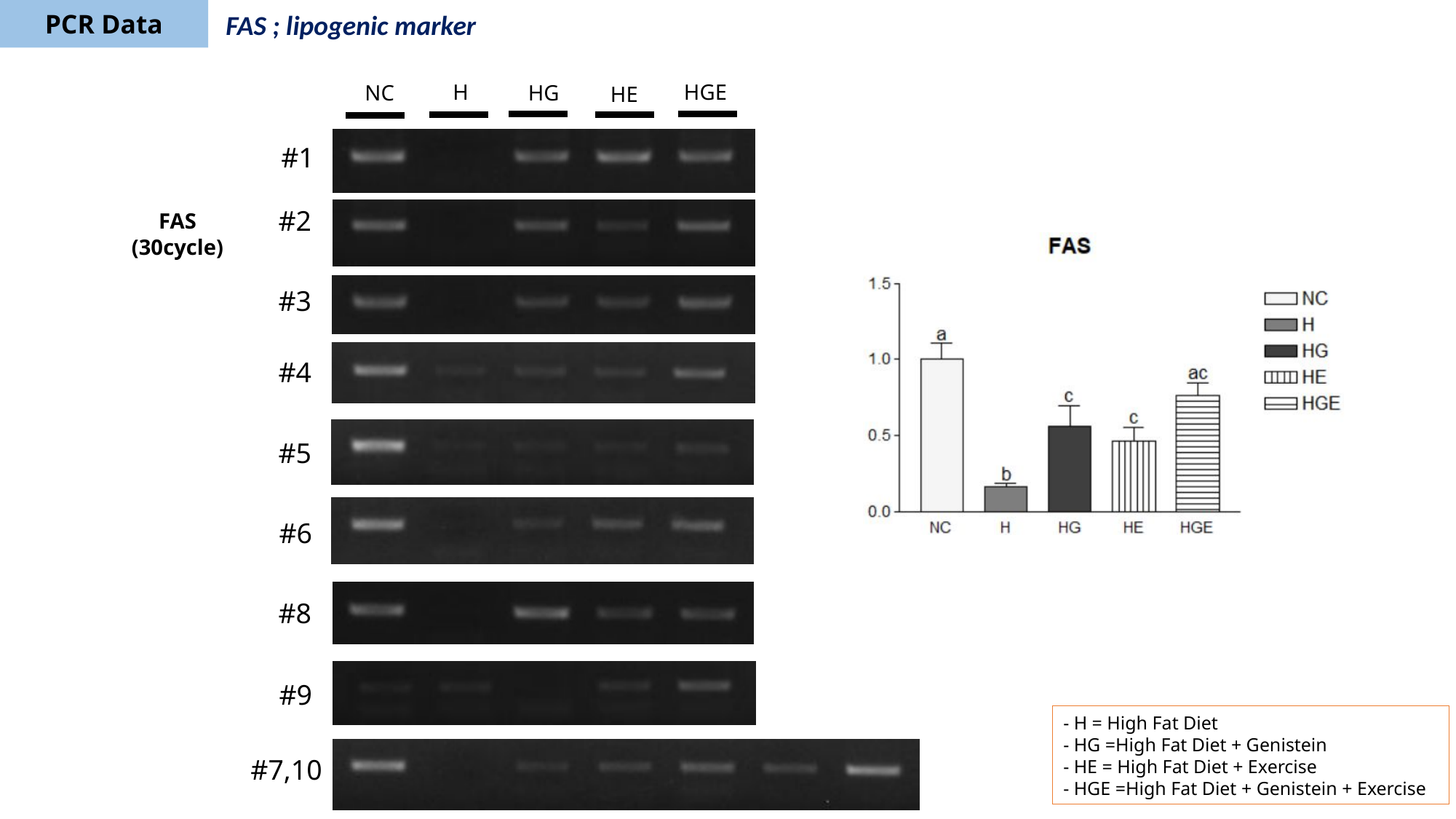

PCR Data
FAS ; lipogenic marker
HGE
H
HG
NC
HE
#1
#2
FAS
(30cycle)
#3
#4
#5
#6
#8
#9
- H = High Fat Diet
- HG =High Fat Diet + Genistein
- HE = High Fat Diet + Exercise
- HGE =High Fat Diet + Genistein + Exercise
#7,10

## Slide 6
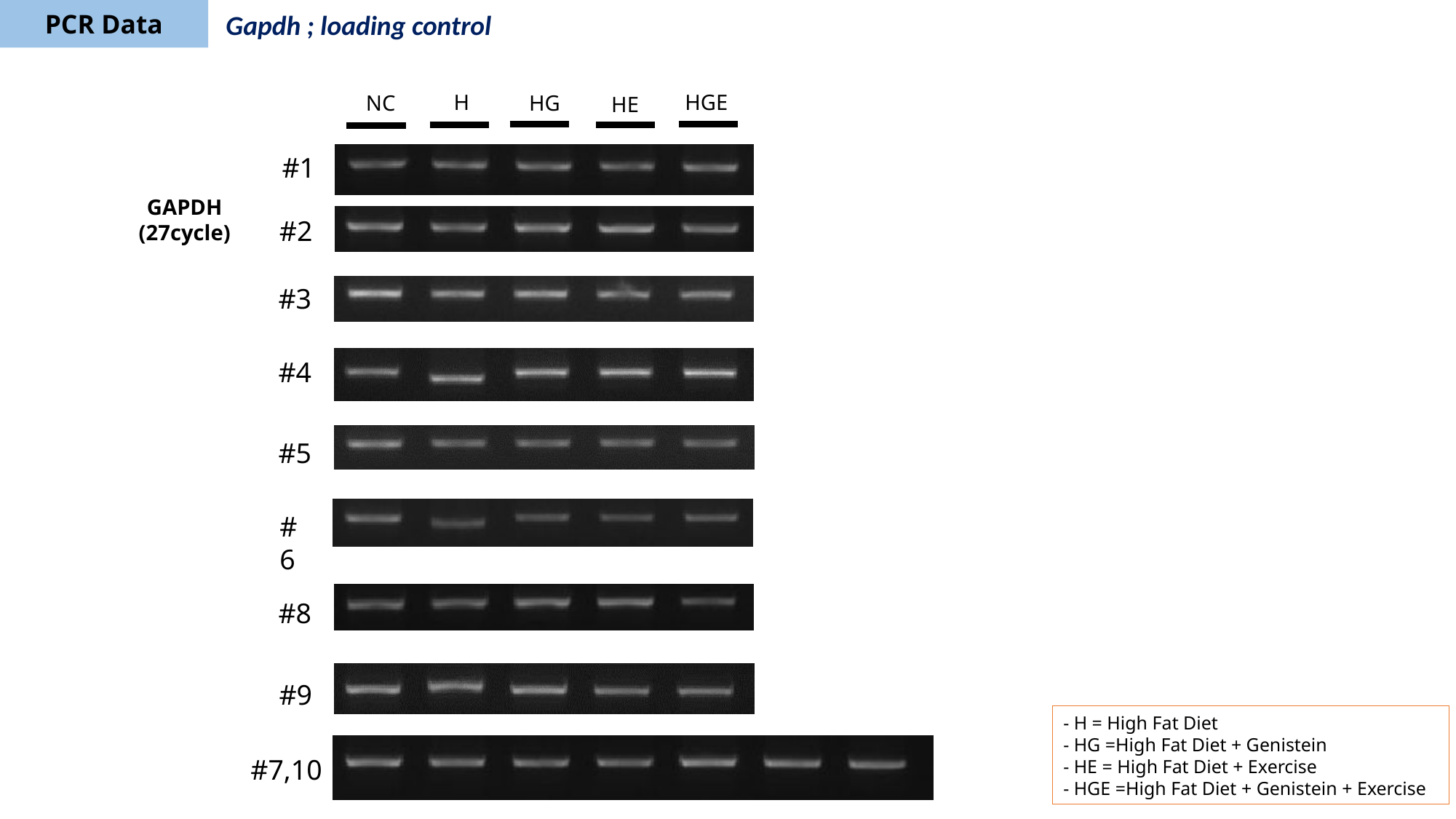

PCR Data
Gapdh ; loading control
HGE
H
HG
NC
HE
#1
GAPDH
(27cycle)
#2
#3
#4
#5
#6
#8
#9
- H = High Fat Diet
- HG =High Fat Diet + Genistein
- HE = High Fat Diet + Exercise
- HGE =High Fat Diet + Genistein + Exercise
#7,10
