# Supplementary material for: Anti-cancer effects of genistein supplementation and moderate-intensity exercise in high-fat diet-induced breast cancer via regulation of inflammation and adipose tissue metabolism in vivo and in vitro
Source: BMC Complement Med Ther. 2025 Jul 2;25:223. doi: 10.1186/s12906-025-04968-x (PMC12225189; doi:10.1186/s12906-025-04968-x)
Supplement: Supplementary file 6 — Supplementary Material 6 [file 12906_2025_4968_MOESM6_ESM.pptx]

## Slide 1
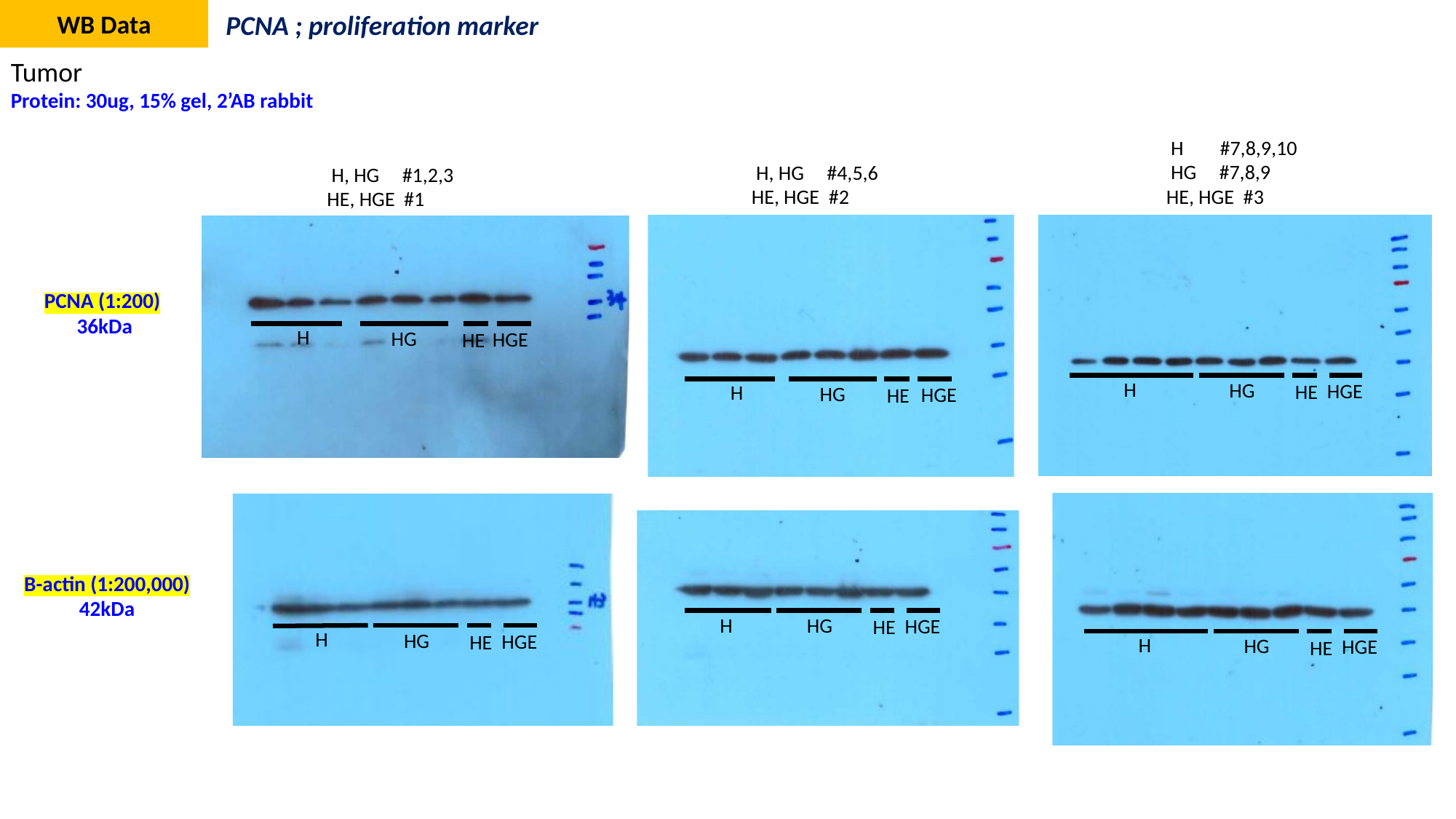

WB Data
PCNA ; proliferation marker
Tumor
Protein: 30ug, 15% gel, 2’AB rabbit
 H #7,8,9,10
 HG #7,8,9
HE, HGE #3
 H, HG #4,5,6
HE, HGE #2
 H, HG #1,2,3
HE, HGE #1
H
HG
HGE
HE
PCNA (1:200)
36kDa
H
HG
HGE
HE
H
HG
HGE
HE
B-actin (1:200,000) 42kDa
H
HG
HGE
HE
H
HG
HGE
HE
H
HG
HGE
HE

## Slide 2
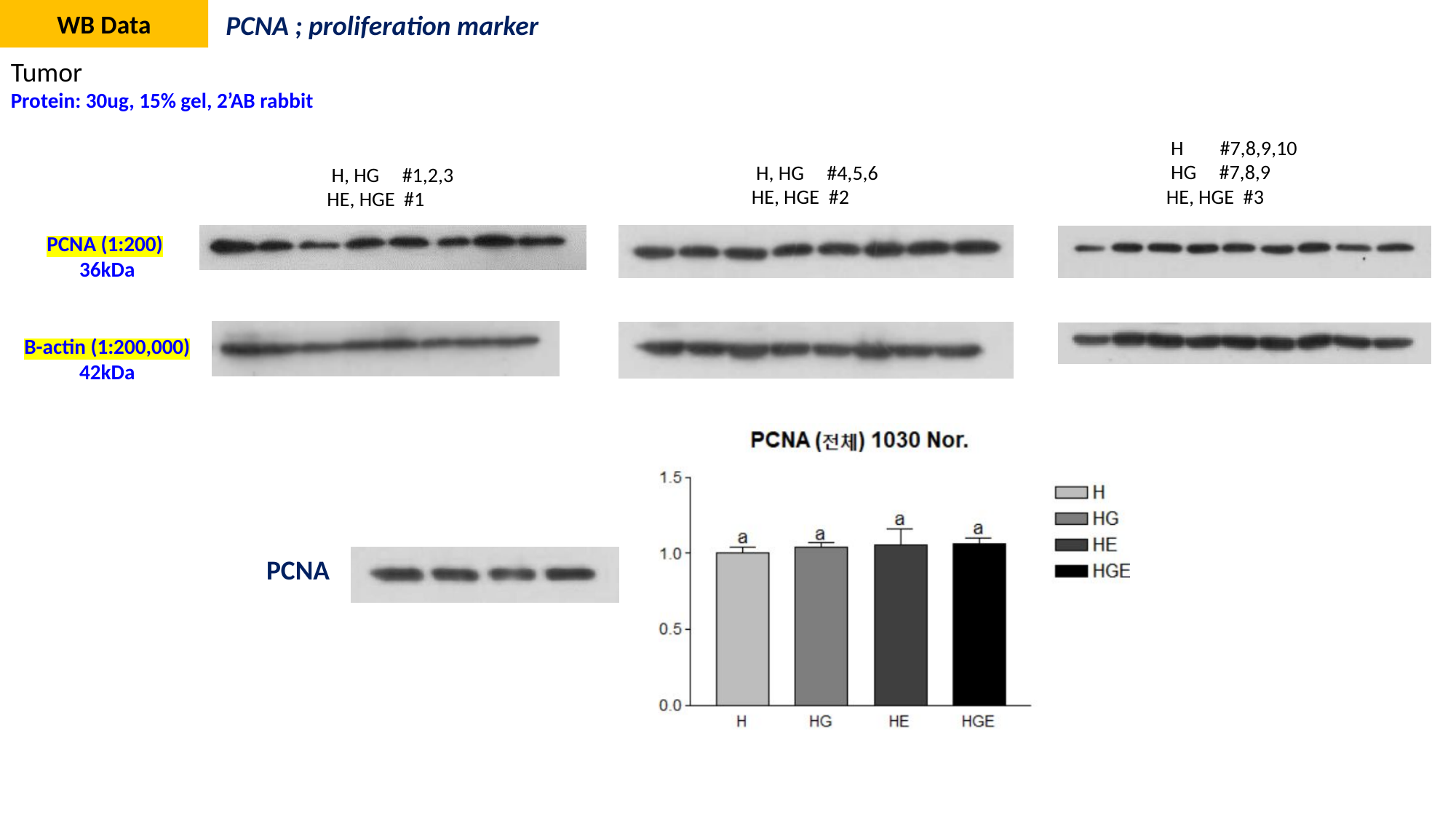

WB Data
PCNA ; proliferation marker
Tumor
Protein: 30ug, 15% gel, 2’AB rabbit
 H #7,8,9,10
 HG #7,8,9
HE, HGE #3
 H, HG #4,5,6
HE, HGE #2
 H, HG #1,2,3
HE, HGE #1
PCNA (1:200)
36kDa
B-actin (1:200,000) 42kDa
PCNA

## Slide 3
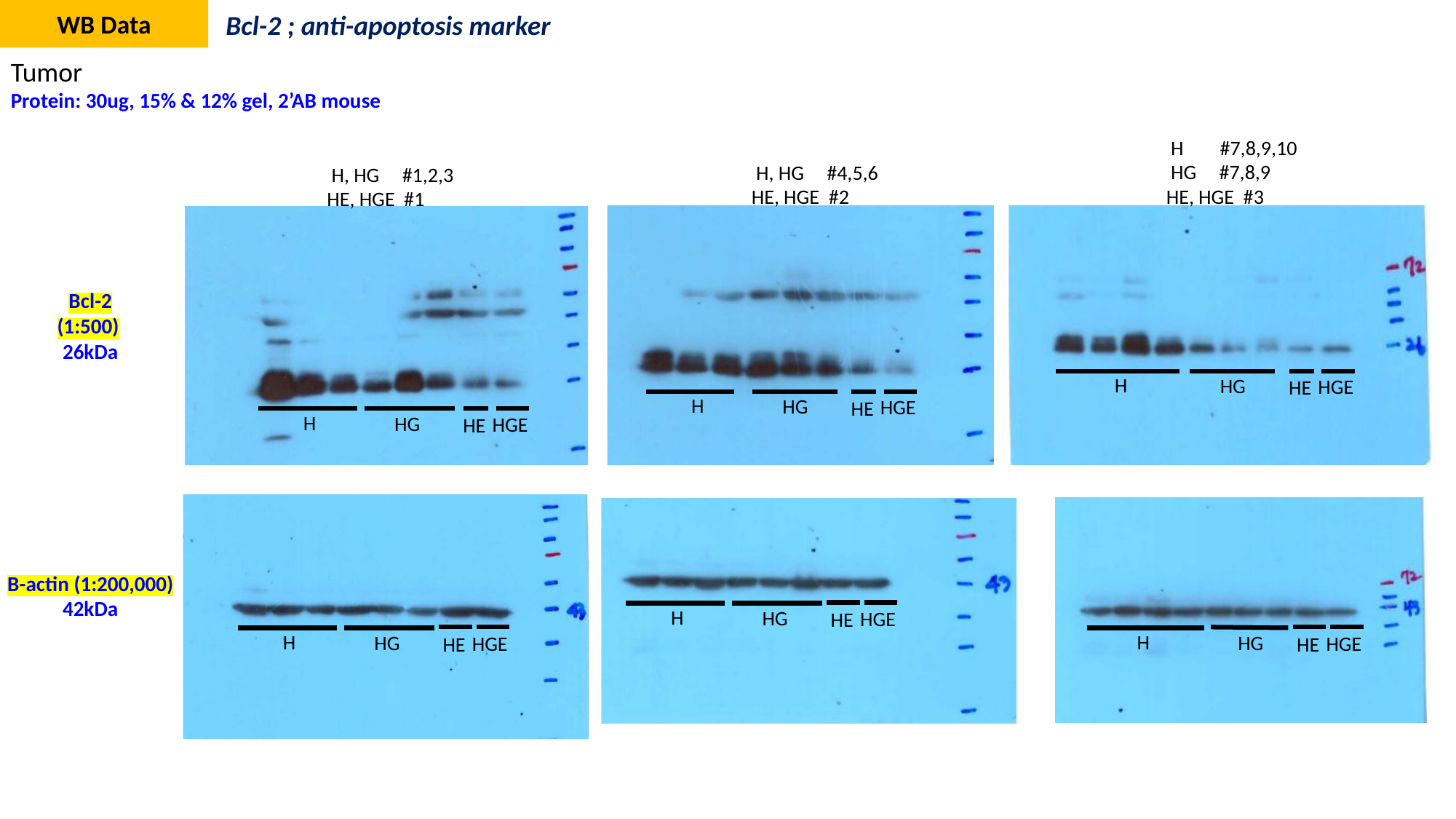

WB Data
Bcl-2 ; anti-apoptosis marker
Tumor
Protein: 30ug, 15% & 12% gel, 2’AB mouse
 H #7,8,9,10
 HG #7,8,9
HE, HGE #3
 H, HG #4,5,6
HE, HGE #2
 H, HG #1,2,3
HE, HGE #1
H
HG
HGE
HE
Bcl-2 (1:500)
26kDa
H
HG
HGE
HE
H
HG
HGE
HE
B-actin (1:200,000) 42kDa
H
HG
HGE
HE
H
HG
HGE
HE
H
HG
HGE
HE

## Slide 4
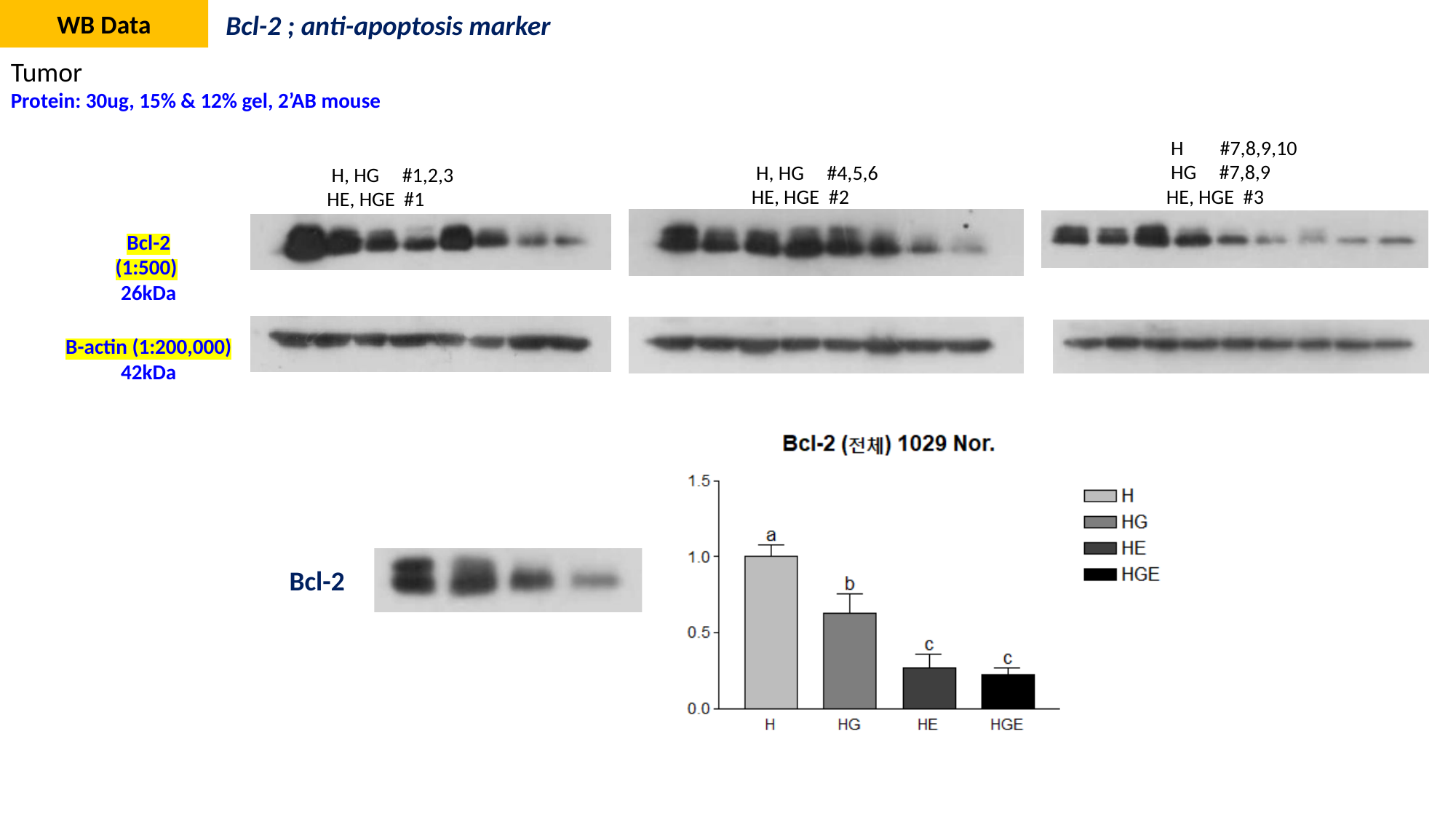

WB Data
Bcl-2 ; anti-apoptosis marker
Tumor
Protein: 30ug, 15% & 12% gel, 2’AB mouse
 H #7,8,9,10
 HG #7,8,9
HE, HGE #3
 H, HG #4,5,6
HE, HGE #2
 H, HG #1,2,3
HE, HGE #1
Bcl-2 (1:500)
26kDa
B-actin (1:200,000) 42kDa
Bcl-2

## Slide 5
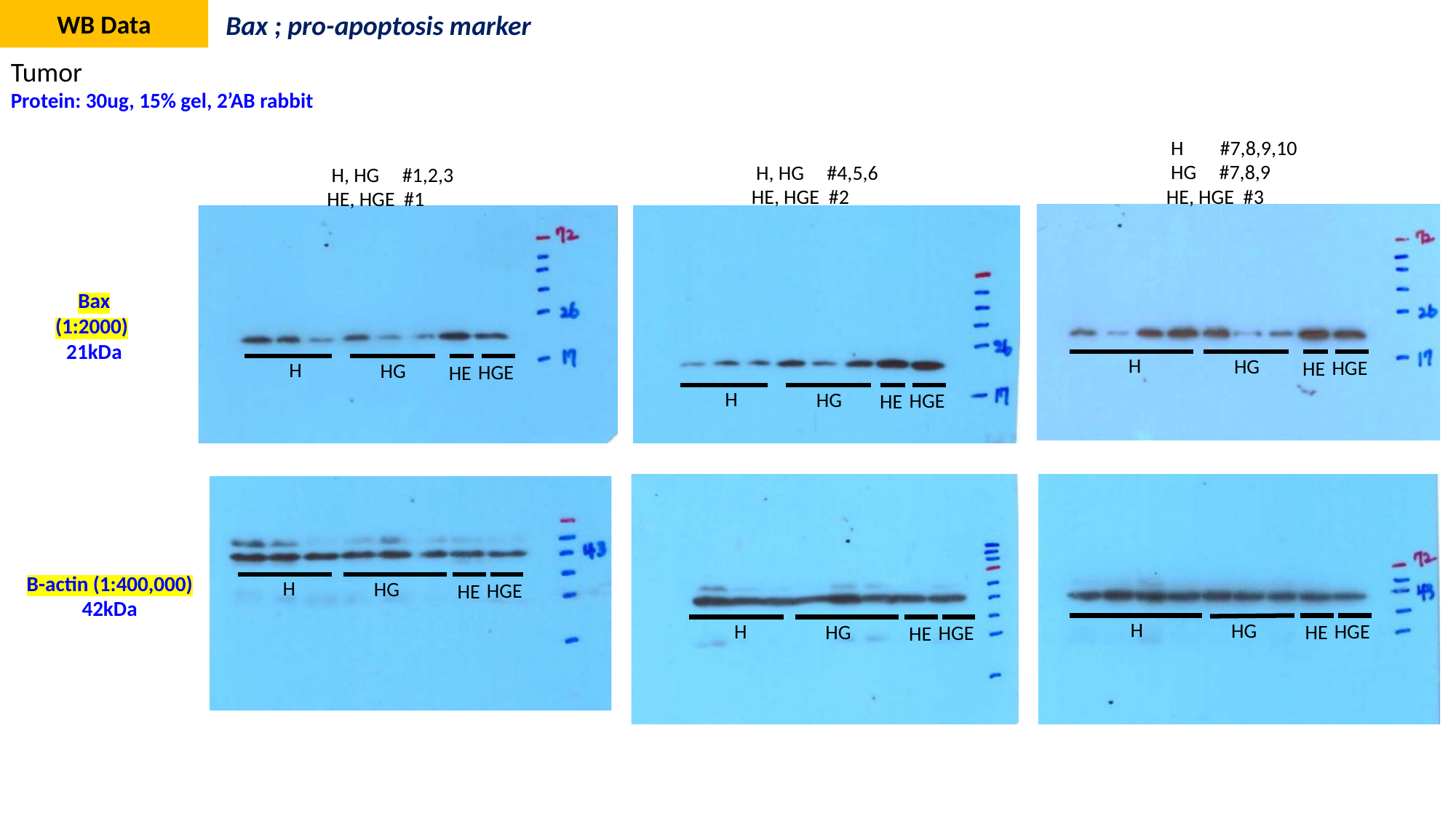

WB Data
Bax ; pro-apoptosis marker
Tumor
Protein: 30ug, 15% gel, 2’AB rabbit
 H #7,8,9,10
 HG #7,8,9
HE, HGE #3
 H, HG #4,5,6
HE, HGE #2
 H, HG #1,2,3
HE, HGE #1
H
HG
HGE
HE
H
HG
HGE
HE
H
HG
HGE
HE
Bax (1:2000)
21kDa
B-actin (1:400,000) 42kDa
H
HG
HGE
HE
H
HG
HGE
HE
H
HG
HGE
HE

## Slide 6
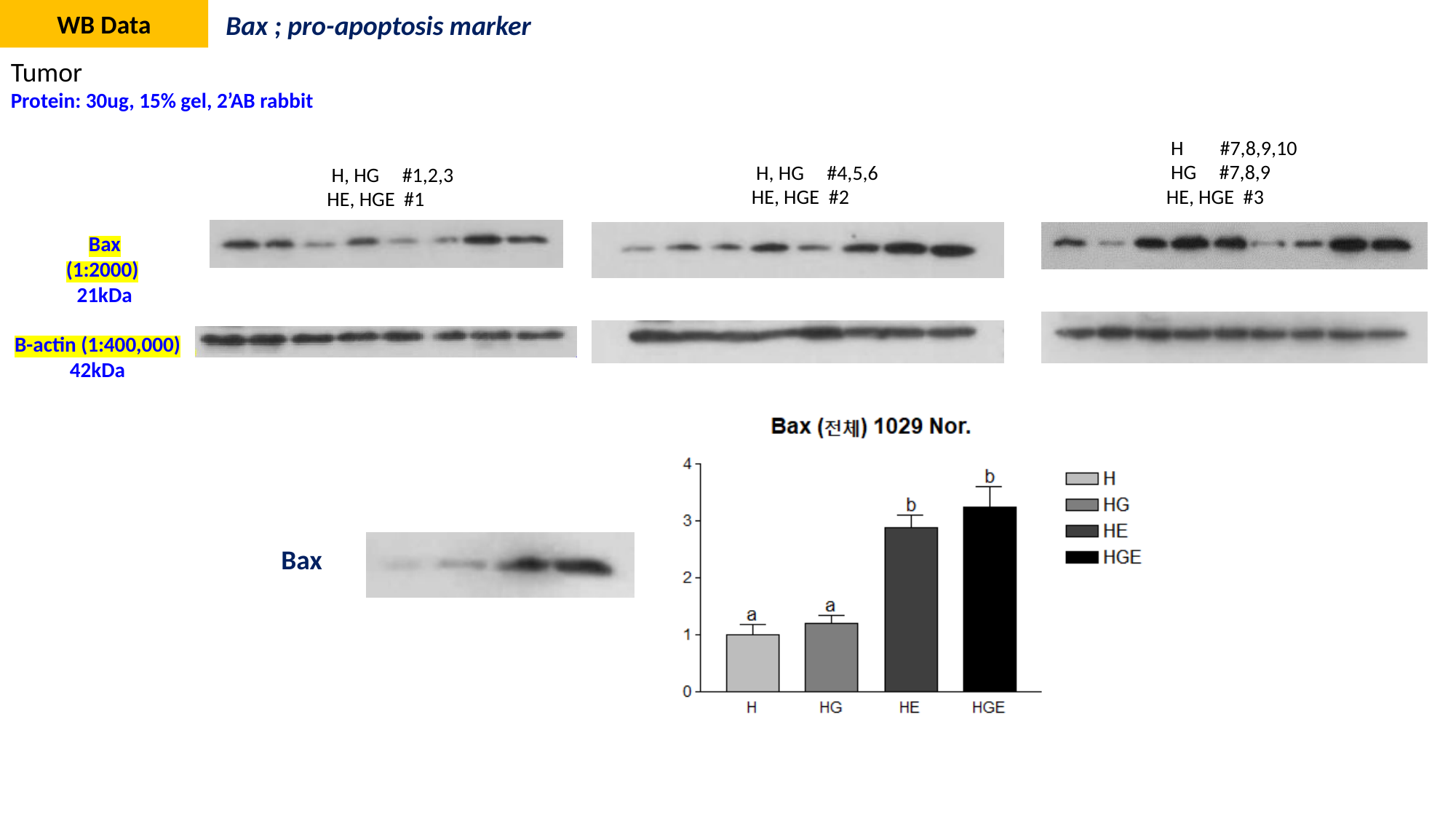

WB Data
Bax ; pro-apoptosis marker
Tumor
Protein: 30ug, 15% gel, 2’AB rabbit
 H #7,8,9,10
 HG #7,8,9
HE, HGE #3
 H, HG #4,5,6
HE, HGE #2
 H, HG #1,2,3
HE, HGE #1
Bax (1:2000)
21kDa
B-actin (1:400,000) 42kDa
Bax

## Slide 7
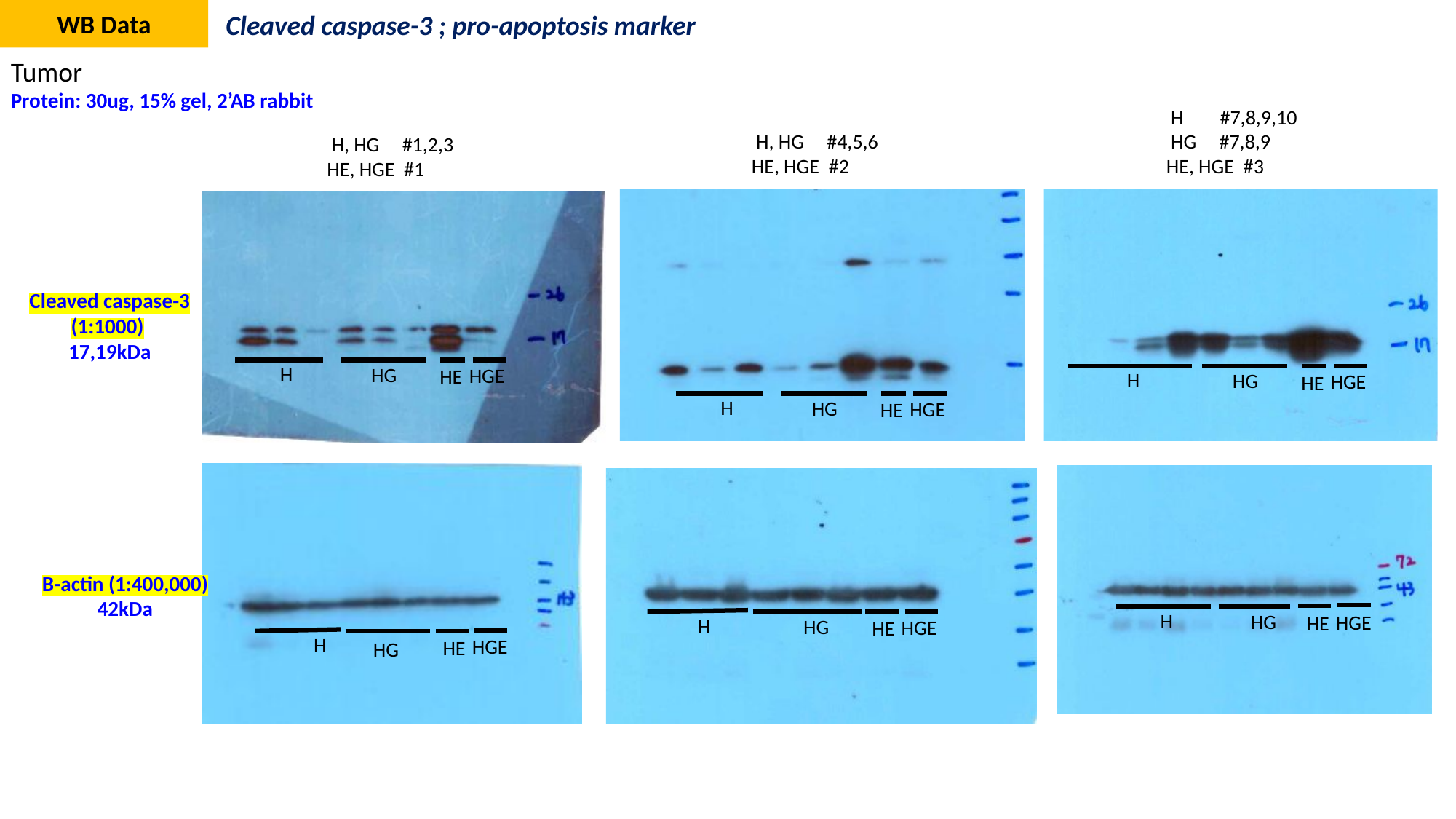

WB Data
Cleaved caspase-3 ; pro-apoptosis marker
Tumor
Protein: 30ug, 15% gel, 2’AB rabbit
 H #7,8,9,10
 HG #7,8,9
HE, HGE #3
 H, HG #4,5,6
HE, HGE #2
 H, HG #1,2,3
HE, HGE #1
H
HG
HGE
HE
H
HG
HGE
HE
H
HG
HGE
HE
Cleaved caspase-3 (1:1000)
17,19kDa
B-actin (1:400,000) 42kDa
H
HG
HGE
HE
H
HG
HGE
HE
H
HGE
HE
HG

## Slide 8
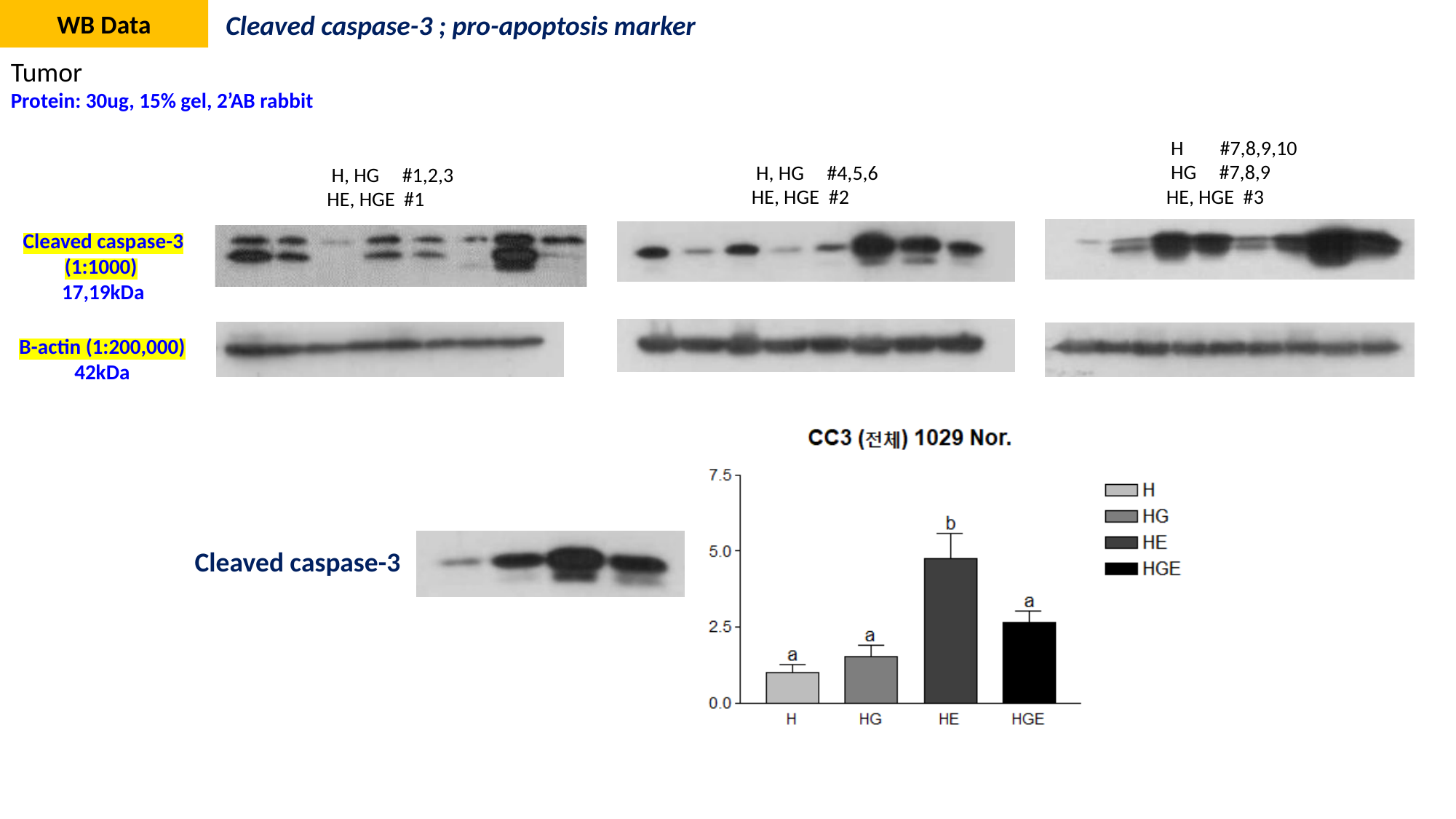

WB Data
Cleaved caspase-3 ; pro-apoptosis marker
Tumor
Protein: 30ug, 15% gel, 2’AB rabbit
 H #7,8,9,10
 HG #7,8,9
HE, HGE #3
 H, HG #4,5,6
HE, HGE #2
 H, HG #1,2,3
HE, HGE #1
Cleaved caspase-3 (1:1000)
17,19kDa
B-actin (1:200,000) 42kDa
Cleaved caspase-3

## Slide 9
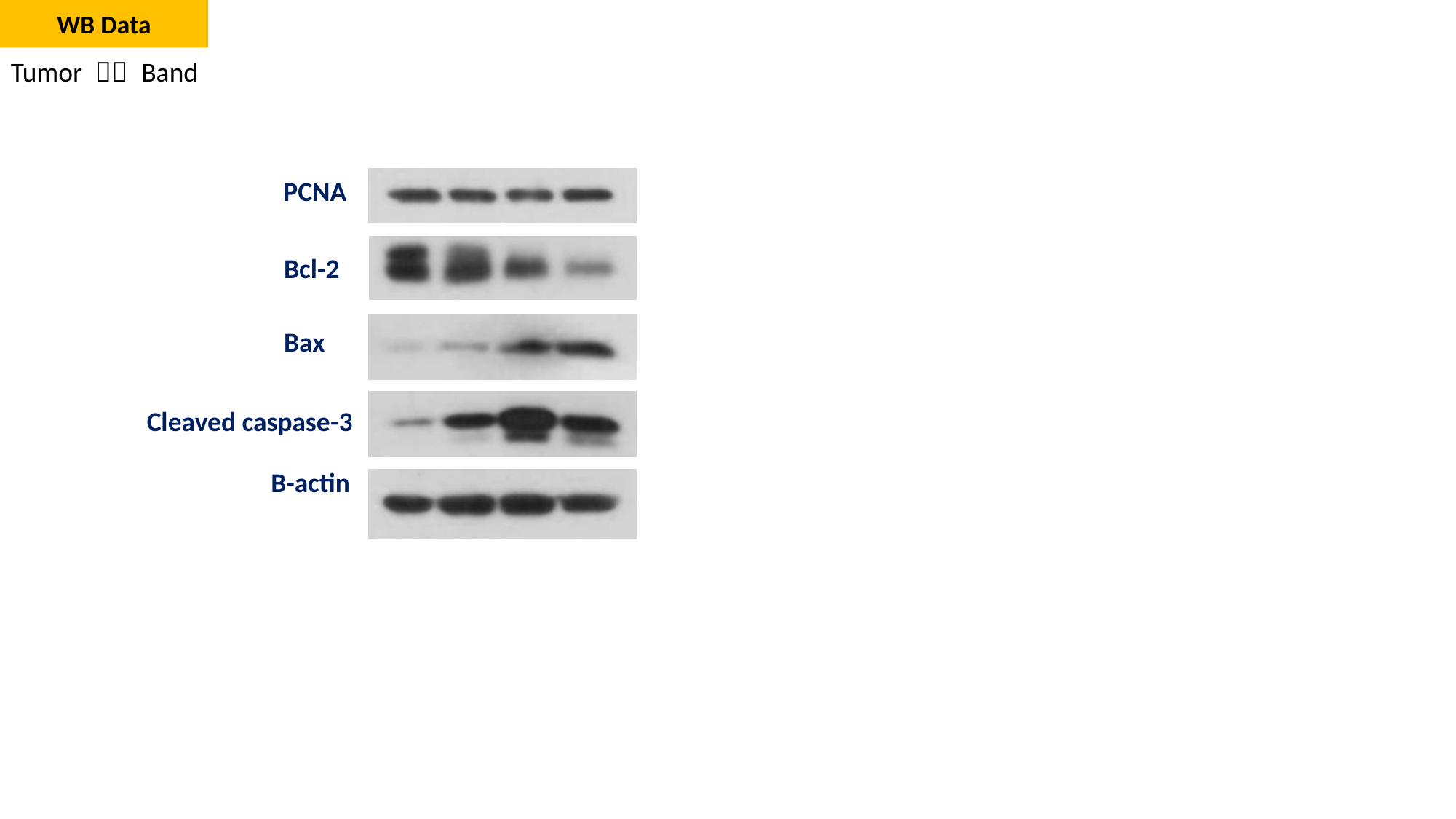

WB Data
Tumor 대표 Band
PCNA
Bcl-2
Bax
Cleaved caspase-3
B-actin
