# Supplementary material for: Anti-cancer effects of genistein supplementation and moderate-intensity exercise in high-fat diet-induced breast cancer via regulation of inflammation and adipose tissue metabolism in vivo and in vitro
Source: BMC Complement Med Ther. 2025 Jul 2;25:223. doi: 10.1186/s12906-025-04968-x (PMC12225189; doi:10.1186/s12906-025-04968-x)
Supplement: Supplementary file 7 — Supplementary Material 7 [file 12906_2025_4968_MOESM7_ESM.pptx]

## Slide 1
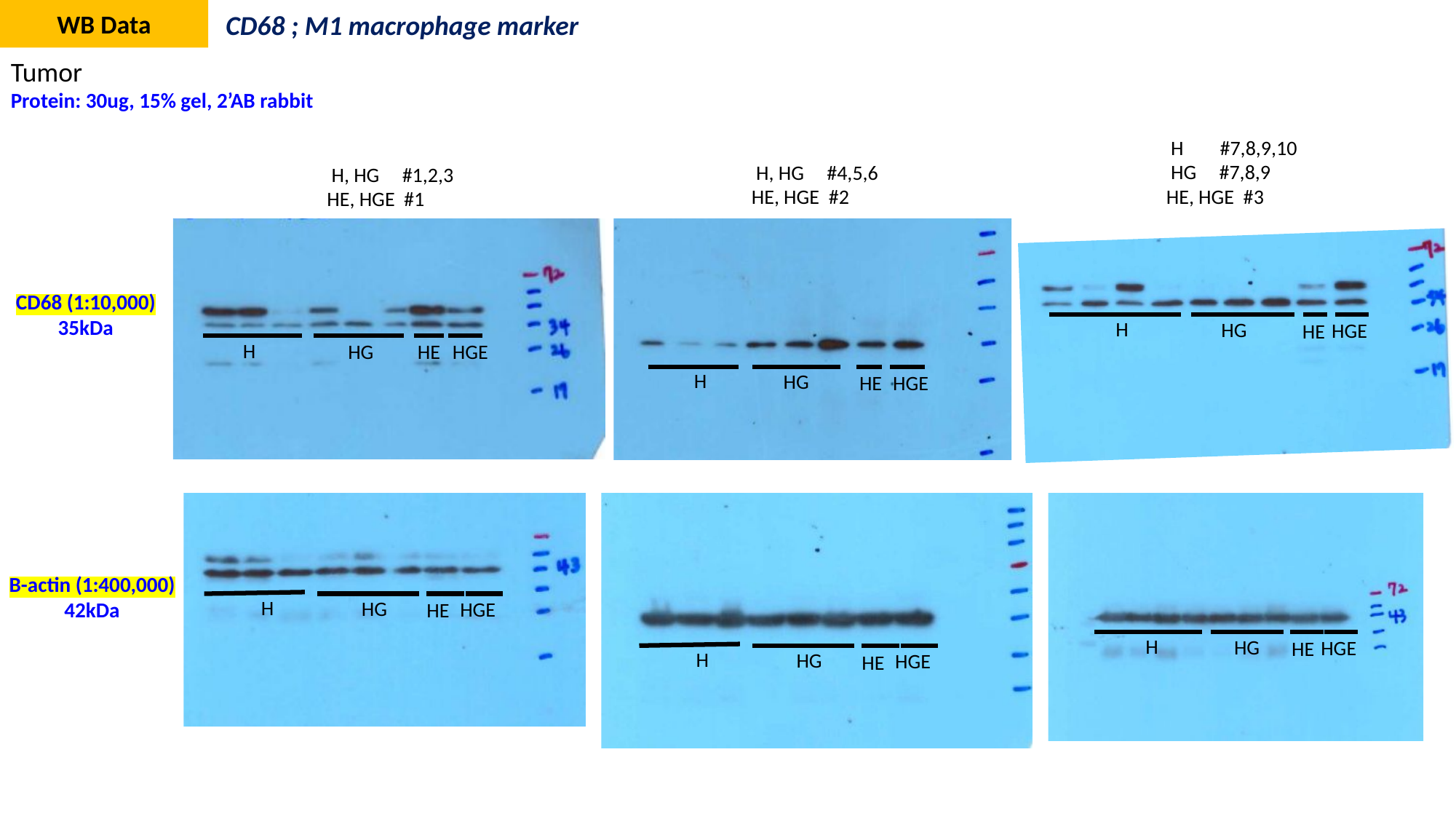

WB Data
CD68 ; M1 macrophage marker
Tumor
Protein: 30ug, 15% gel, 2’AB rabbit
 H #7,8,9,10
 HG #7,8,9
HE, HGE #3
 H, HG #4,5,6
HE, HGE #2
 H, HG #1,2,3
HE, HGE #1
H
HE
HGE
HG
H
HG
HGE
HE
H
HG
HGE
HE
CD68 (1:10,000)
35kDa
B-actin (1:400,000) 42kDa
H
HG
HGE
HE
H
HG
HGE
HE
H
HG
HGE
HE

## Slide 2
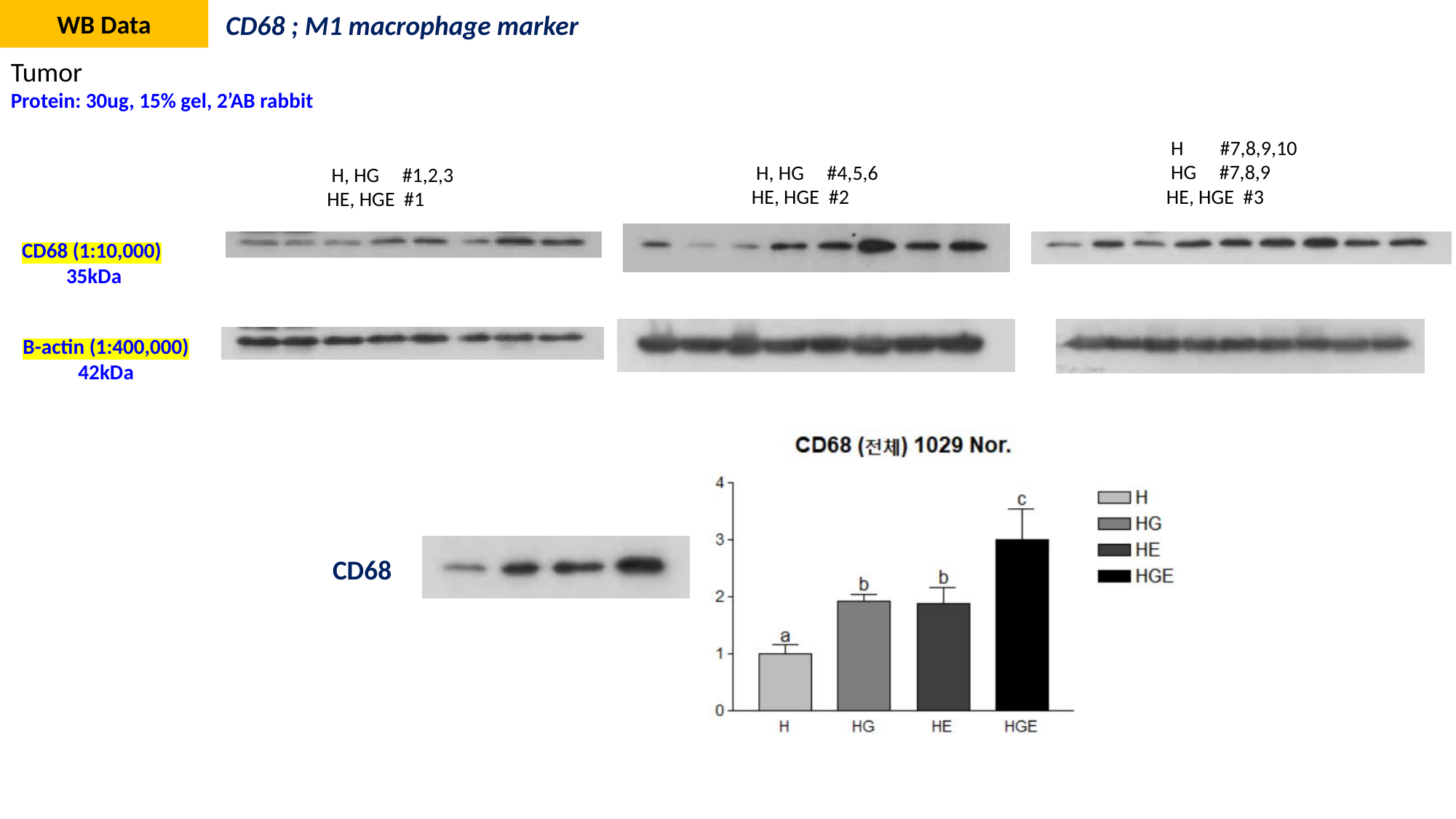

WB Data
CD68 ; M1 macrophage marker
Tumor
Protein: 30ug, 15% gel, 2’AB rabbit
 H #7,8,9,10
 HG #7,8,9
HE, HGE #3
 H, HG #4,5,6
HE, HGE #2
 H, HG #1,2,3
HE, HGE #1
CD68 (1:10,000)
35kDa
B-actin (1:400,000) 42kDa
CD68

## Slide 3
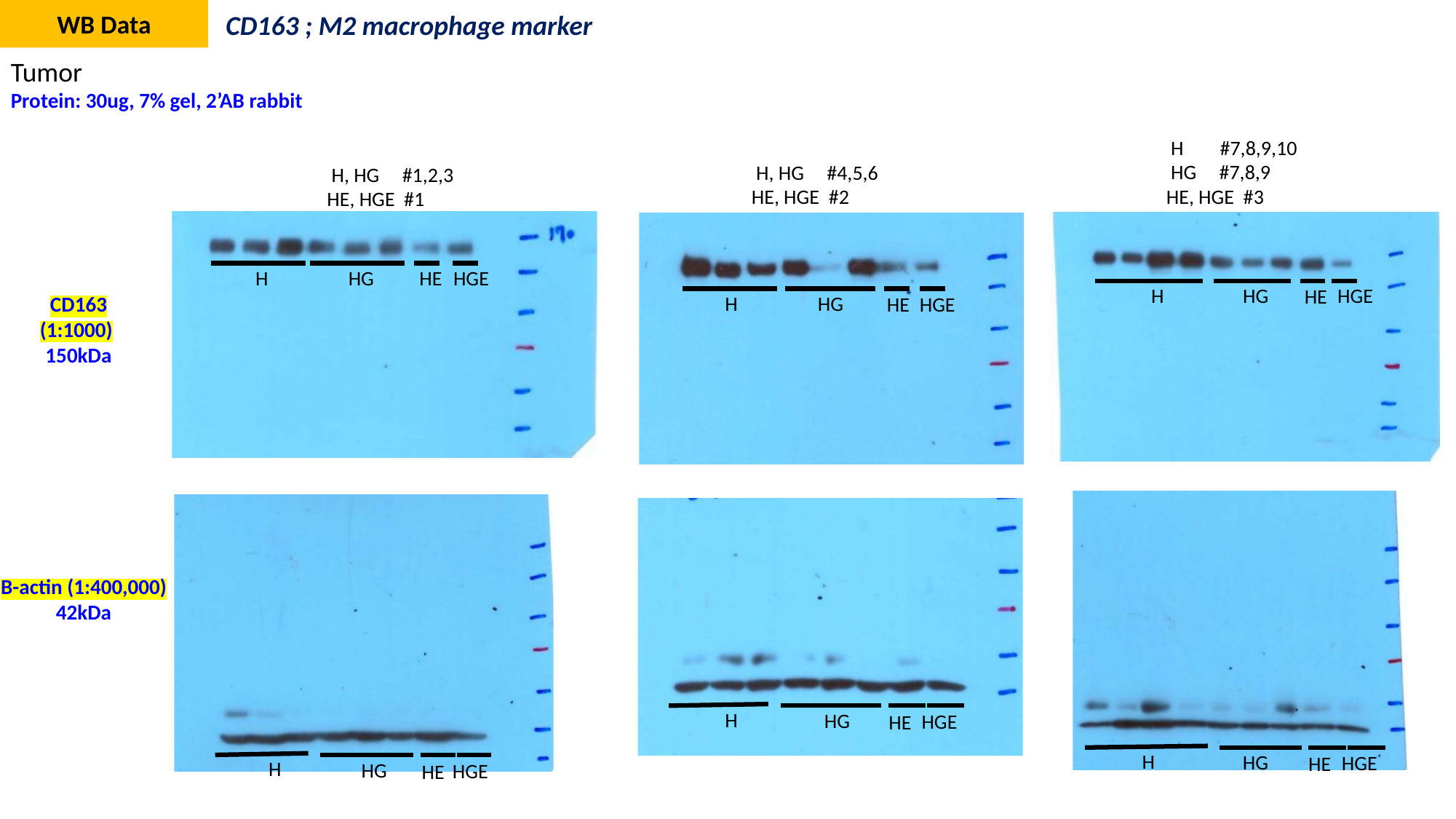

WB Data
CD163 ; M2 macrophage marker
Tumor
Protein: 30ug, 7% gel, 2’AB rabbit
 H #7,8,9,10
 HG #7,8,9
HE, HGE #3
 H, HG #4,5,6
HE, HGE #2
 H, HG #1,2,3
HE, HGE #1
H
HG
HE
HGE
H
HG
HGE
HE
CD163 (1:1000)
150kDa
H
HG
HE
HGE
B-actin (1:400,000) 42kDa
H
HG
HGE
HE
H
HG
HGE
HE
H
HG
HGE
HE

## Slide 4
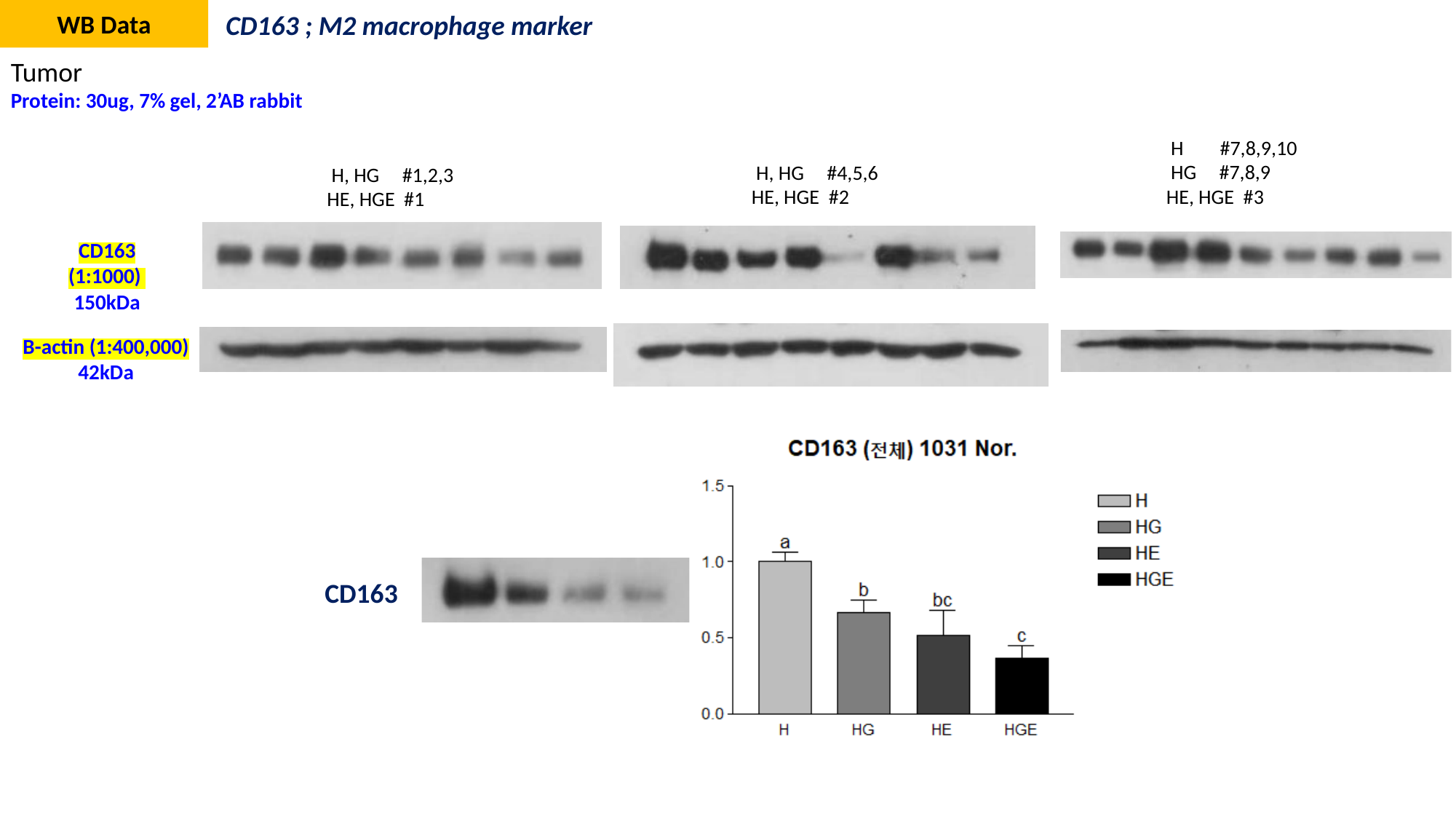

WB Data
CD163 ; M2 macrophage marker
Tumor
Protein: 30ug, 7% gel, 2’AB rabbit
 H #7,8,9,10
 HG #7,8,9
HE, HGE #3
 H, HG #4,5,6
HE, HGE #2
 H, HG #1,2,3
HE, HGE #1
CD163 (1:1000)
150kDa
B-actin (1:400,000) 42kDa
CD163

## Slide 5
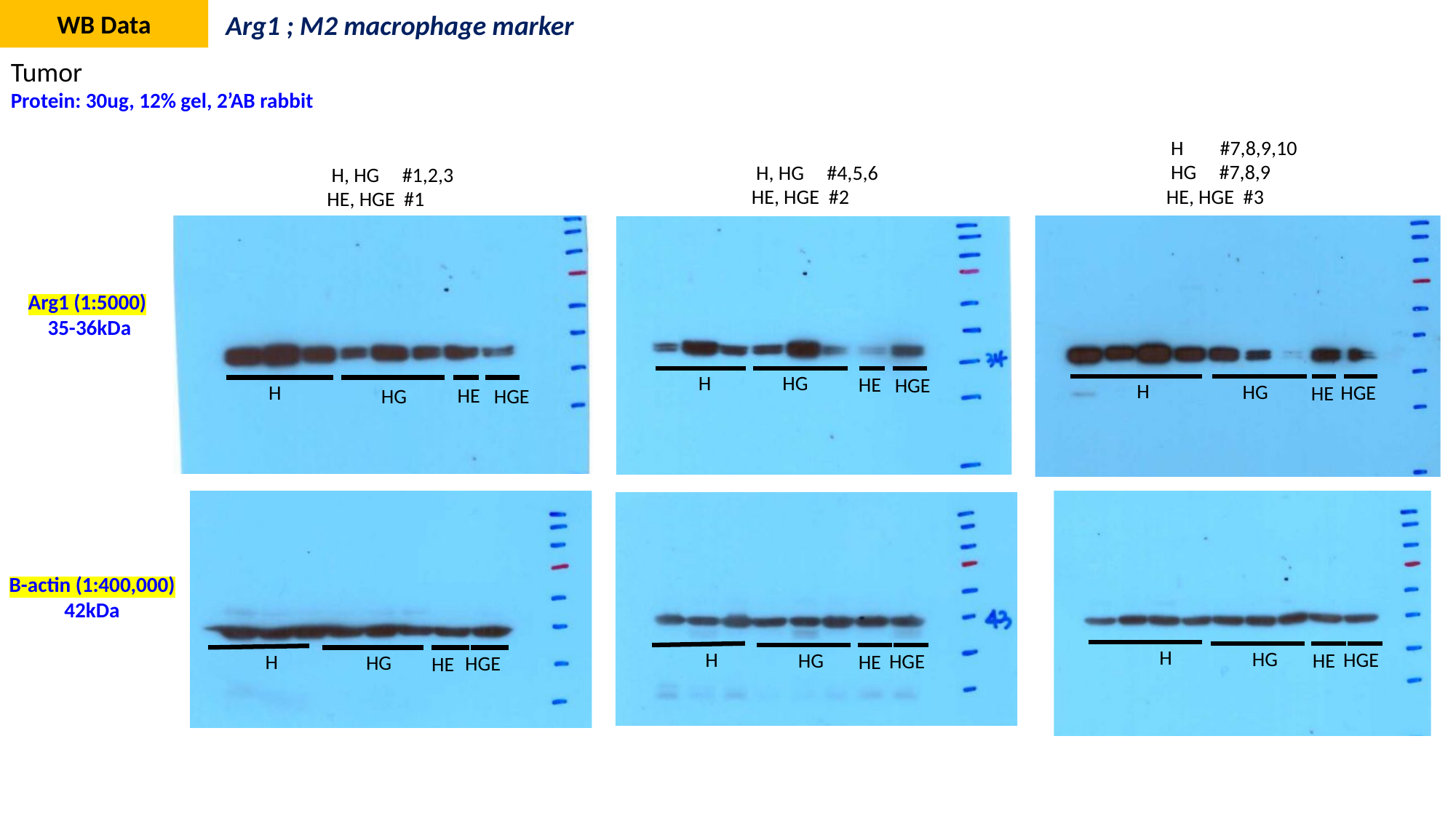

WB Data
Arg1 ; M2 macrophage marker
Tumor
Protein: 30ug, 12% gel, 2’AB rabbit
 H #7,8,9,10
 HG #7,8,9
HE, HGE #3
 H, HG #4,5,6
HE, HGE #2
 H, HG #1,2,3
HE, HGE #1
H
HE
HGE
HG
H
HG
HE
HGE
Arg1 (1:5000)
35-36kDa
H
HG
HGE
HE
B-actin (1:400,000) 42kDa
H
HG
HGE
HE
H
HG
HGE
HE
H
HG
HGE
HE

## Slide 6
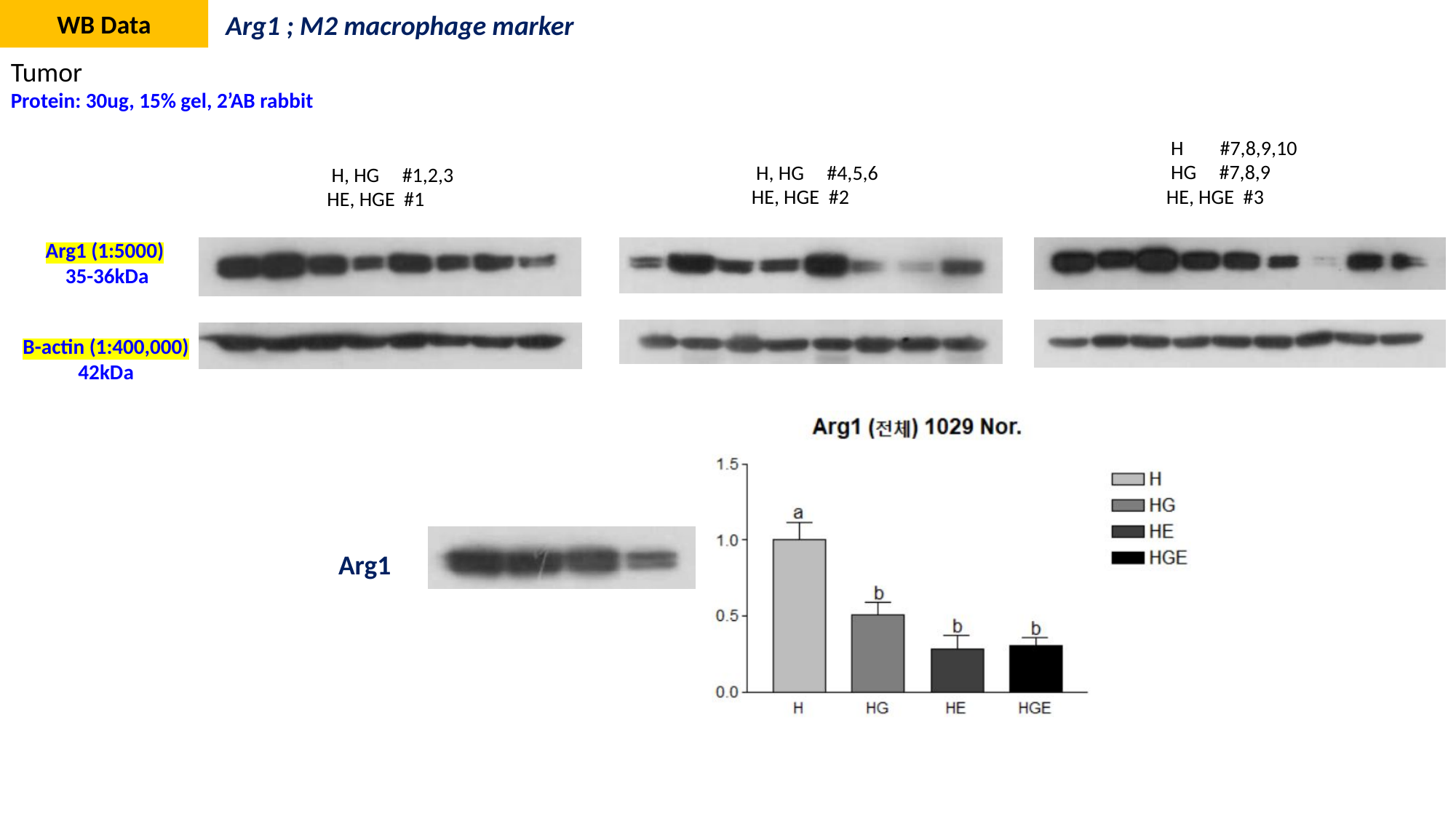

WB Data
Arg1 ; M2 macrophage marker
Tumor
Protein: 30ug, 15% gel, 2’AB rabbit
 H #7,8,9,10
 HG #7,8,9
HE, HGE #3
 H, HG #4,5,6
HE, HGE #2
 H, HG #1,2,3
HE, HGE #1
Arg1 (1:5000)
35-36kDa
B-actin (1:400,000) 42kDa
Arg1

## Slide 7
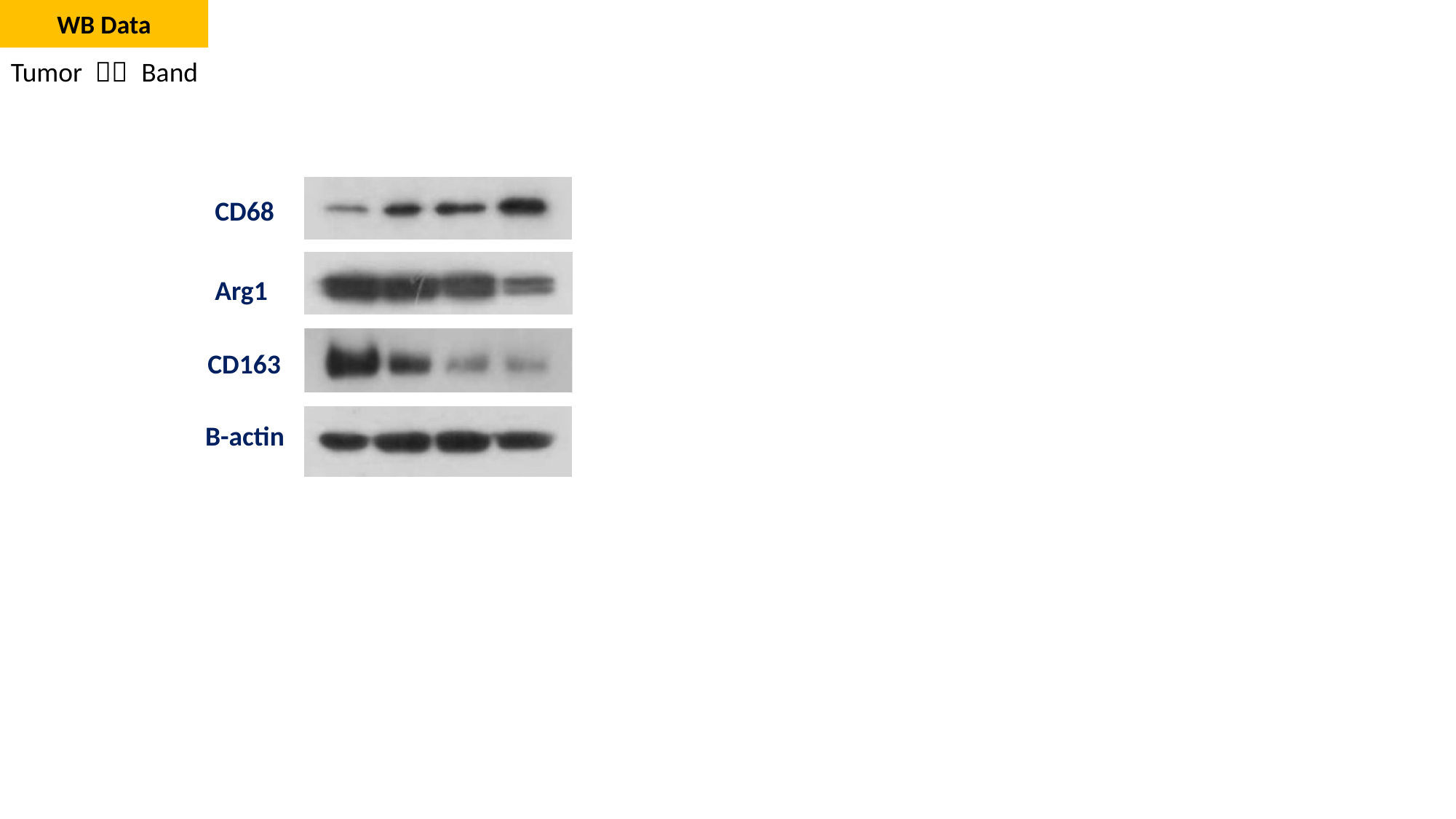

WB Data
Tumor 대표 Band
CD68
Arg1
CD163
B-actin
