# Supplementary material for: Anti-cancer effects of genistein supplementation and moderate-intensity exercise in high-fat diet-induced breast cancer via regulation of inflammation and adipose tissue metabolism in vivo and in vitro
Source: BMC Complement Med Ther. 2025 Jul 2;25:223. doi: 10.1186/s12906-025-04968-x (PMC12225189; doi:10.1186/s12906-025-04968-x)
Supplement: Supplementary file 8 — Supplementary Material 8 [file 12906_2025_4968_MOESM8_ESM.pptx]

## Slide 1
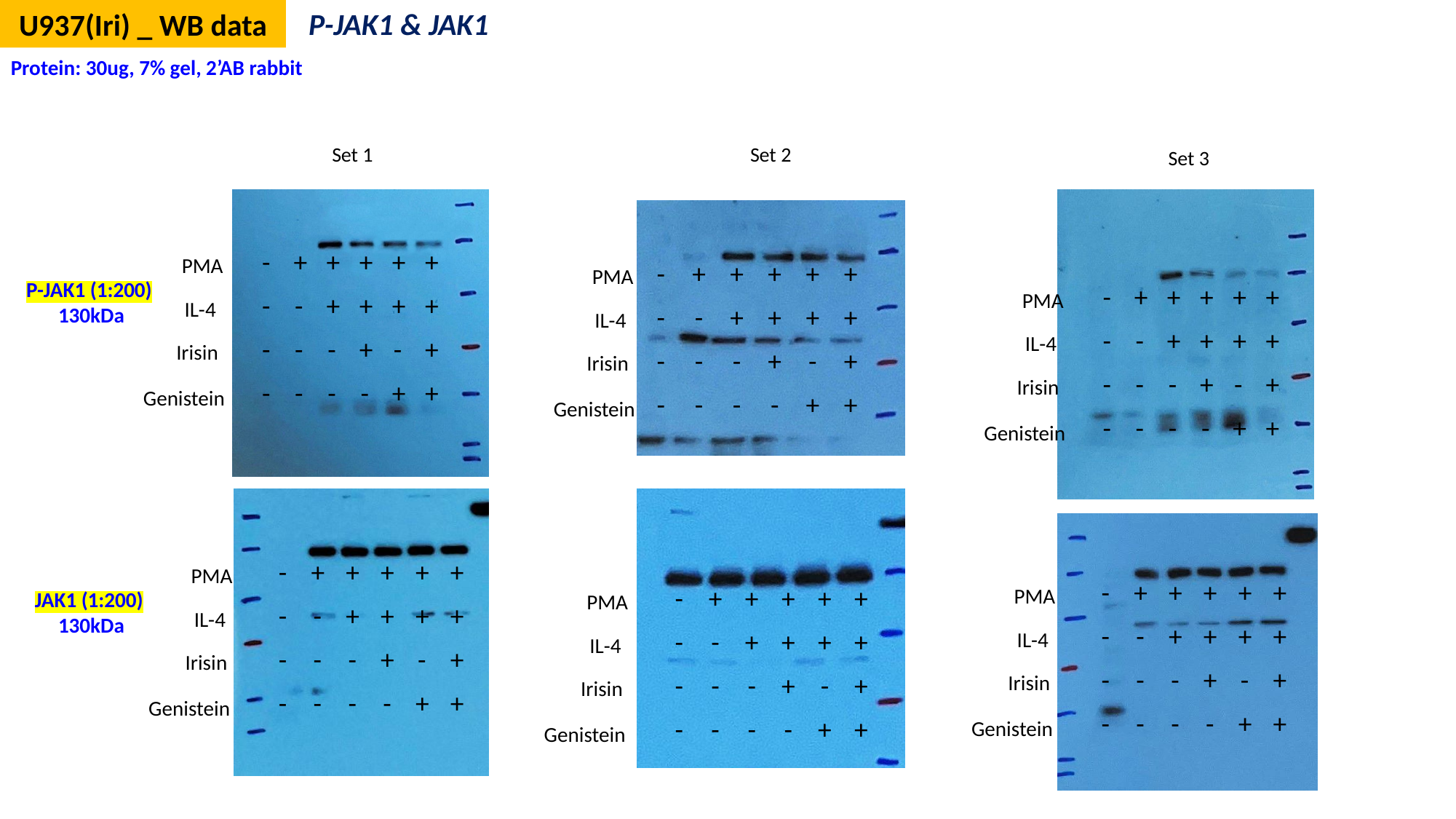

P-JAK1 & JAK1
U937(Iri) _ WB data
Protein: 30ug, 7% gel, 2’AB rabbit
Set 1
Set 2
Set 3
| - | + | + | + | + | + |
| --- | --- | --- | --- | --- | --- |
| - | - | + | + | + | + |
| - | - | - | + | - | + |
| - | - | - | - | + | + |
PMA
IL-4
Irisin
Genistein
PMA
IL-4
Irisin
Genistein
| - | + | + | + | + | + |
| --- | --- | --- | --- | --- | --- |
| - | - | + | + | + | + |
| - | - | - | + | - | + |
| - | - | - | - | + | + |
P-JAK1 (1:200)
130kDa
| - | + | + | + | + | + |
| --- | --- | --- | --- | --- | --- |
| - | - | + | + | + | + |
| - | - | - | + | - | + |
| - | - | - | - | + | + |
PMA
IL-4
Irisin
Genistein
| - | + | + | + | + | + |
| --- | --- | --- | --- | --- | --- |
| - | - | + | + | + | + |
| - | - | - | + | - | + |
| - | - | - | - | + | + |
PMA
IL-4
Irisin
Genistein
| - | + | + | + | + | + |
| --- | --- | --- | --- | --- | --- |
| - | - | + | + | + | + |
| - | - | - | + | - | + |
| - | - | - | - | + | + |
PMA
IL-4
Irisin
Genistein
JAK1 (1:200)
130kDa
| - | + | + | + | + | + |
| --- | --- | --- | --- | --- | --- |
| - | - | + | + | + | + |
| - | - | - | + | - | + |
| - | - | - | - | + | + |
PMA
IL-4
Irisin
Genistein

## Slide 2
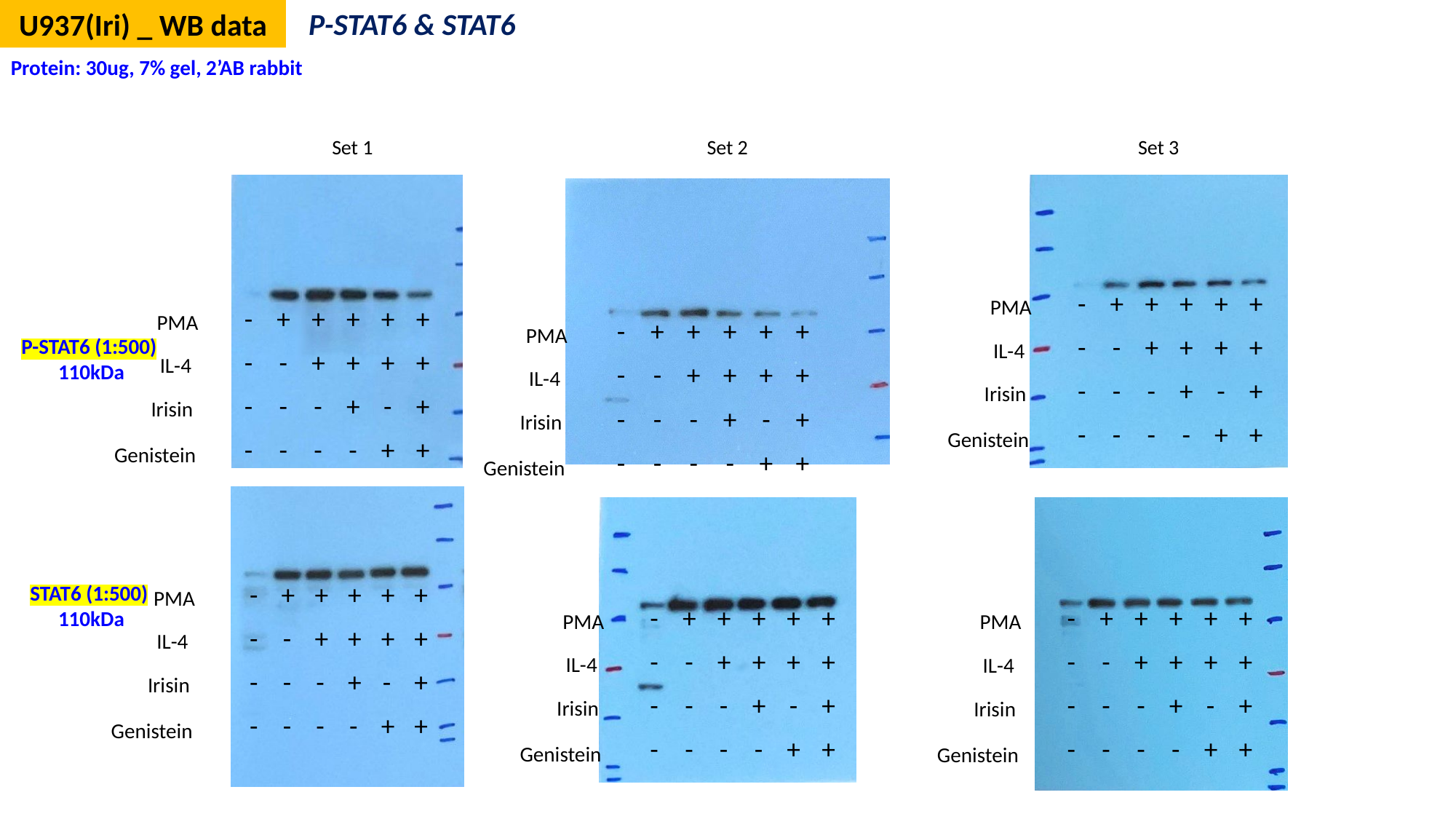

P-STAT6 & STAT6
U937(Iri) _ WB data
Protein: 30ug, 7% gel, 2’AB rabbit
Set 1
Set 2
Set 3
| - | + | + | + | + | + |
| --- | --- | --- | --- | --- | --- |
| - | - | + | + | + | + |
| - | - | - | + | - | + |
| - | - | - | - | + | + |
PMA
IL-4
Irisin
Genistein
| - | + | + | + | + | + |
| --- | --- | --- | --- | --- | --- |
| - | - | + | + | + | + |
| - | - | - | + | - | + |
| - | - | - | - | + | + |
PMA
IL-4
Irisin
Genistein
| - | + | + | + | + | + |
| --- | --- | --- | --- | --- | --- |
| - | - | + | + | + | + |
| - | - | - | + | - | + |
| - | - | - | - | + | + |
PMA
IL-4
Irisin
Genistein
P-STAT6 (1:500)
110kDa
STAT6 (1:500)
110kDa
| - | + | + | + | + | + |
| --- | --- | --- | --- | --- | --- |
| - | - | + | + | + | + |
| - | - | - | + | - | + |
| - | - | - | - | + | + |
PMA
IL-4
Irisin
Genistein
| - | + | + | + | + | + |
| --- | --- | --- | --- | --- | --- |
| - | - | + | + | + | + |
| - | - | - | + | - | + |
| - | - | - | - | + | + |
| - | + | + | + | + | + |
| --- | --- | --- | --- | --- | --- |
| - | - | + | + | + | + |
| - | - | - | + | - | + |
| - | - | - | - | + | + |
PMA
IL-4
Irisin
Genistein
PMA
IL-4
Irisin
Genistein

## Slide 3
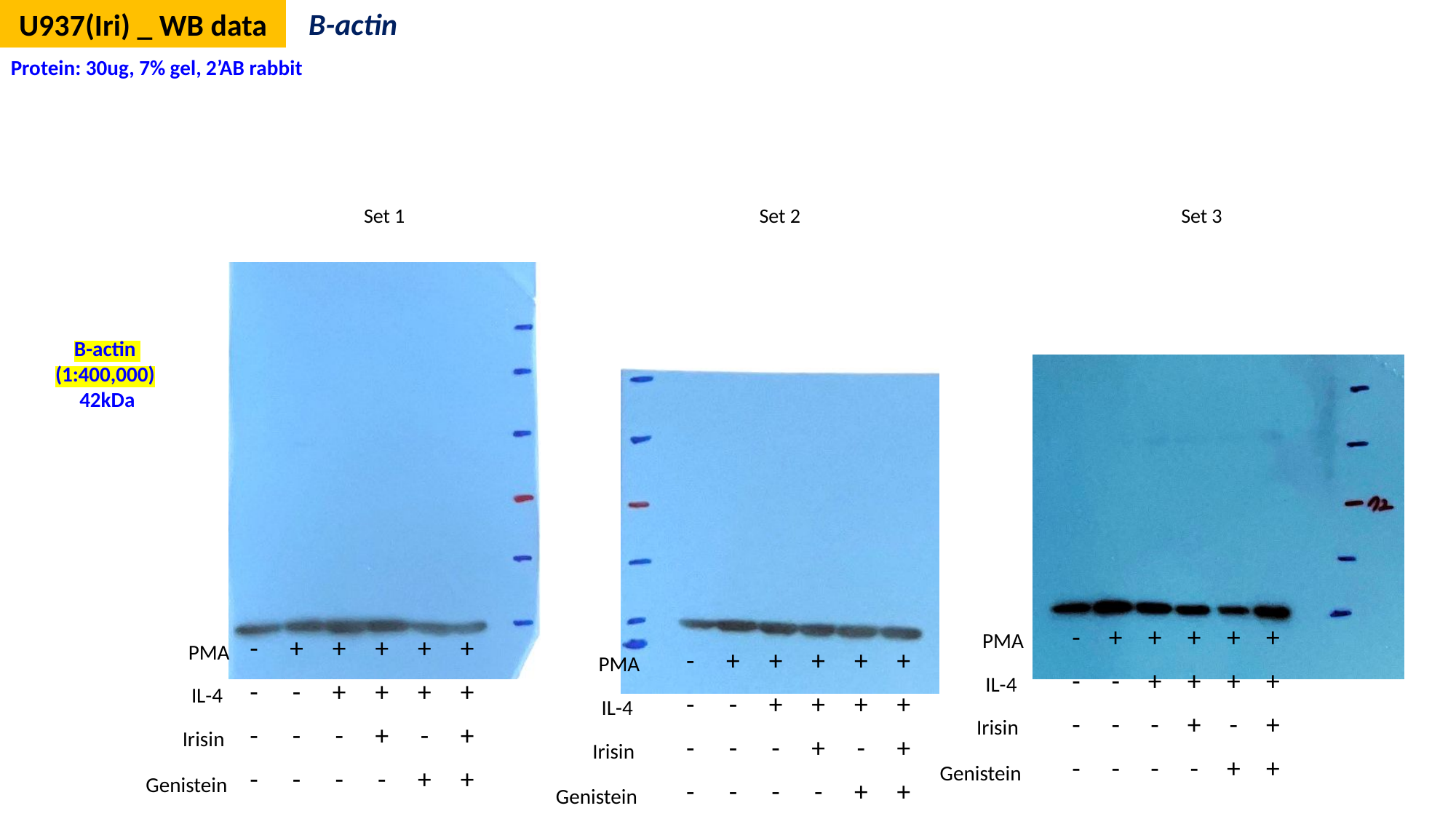

B-actin
U937(Iri) _ WB data
Protein: 30ug, 7% gel, 2’AB rabbit
Set 1
Set 2
Set 3
B-actin
(1:400,000)
42kDa
| - | + | + | + | + | + |
| --- | --- | --- | --- | --- | --- |
| - | - | + | + | + | + |
| - | - | - | + | - | + |
| - | - | - | - | + | + |
PMA
IL-4
Irisin
Genistein
| - | + | + | + | + | + |
| --- | --- | --- | --- | --- | --- |
| - | - | + | + | + | + |
| - | - | - | + | - | + |
| - | - | - | - | + | + |
PMA
IL-4
Irisin
Genistein
| - | + | + | + | + | + |
| --- | --- | --- | --- | --- | --- |
| - | - | + | + | + | + |
| - | - | - | + | - | + |
| - | - | - | - | + | + |
PMA
IL-4
Irisin
Genistein

## Slide 4
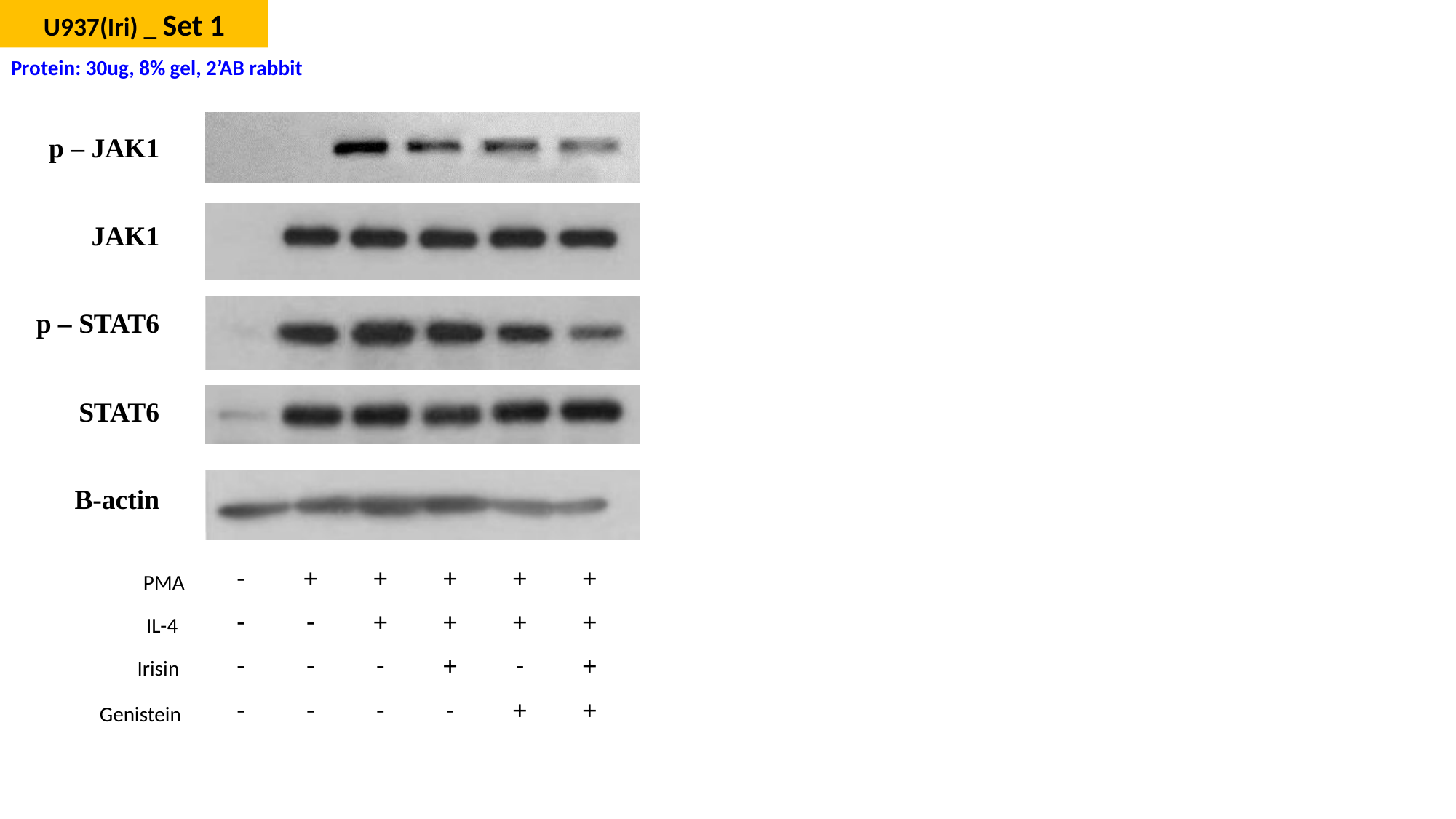

U937(Iri) _ Set 1
Protein: 30ug, 8% gel, 2’AB rabbit
p – JAK1
JAK1
p – STAT6
STAT6
B-actin
| - | + | + | + | + | + |
| --- | --- | --- | --- | --- | --- |
| - | - | + | + | + | + |
| - | - | - | + | - | + |
| - | - | - | - | + | + |
PMA
IL-4
Irisin
Genistein

## Slide 5
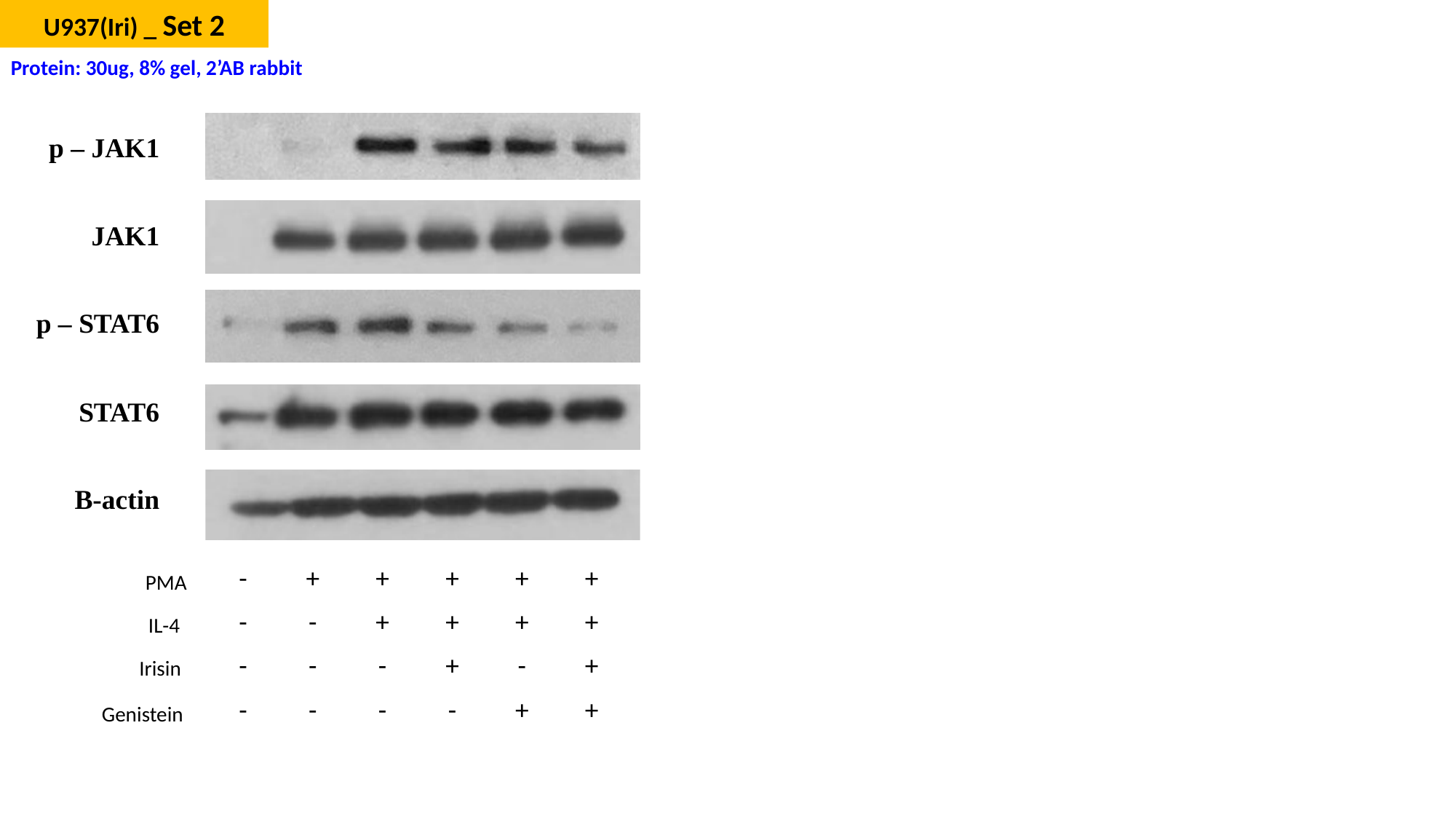

U937(Iri) _ Set 2
Protein: 30ug, 8% gel, 2’AB rabbit
p – JAK1
JAK1
p – STAT6
STAT6
B-actin
| - | + | + | + | + | + |
| --- | --- | --- | --- | --- | --- |
| - | - | + | + | + | + |
| - | - | - | + | - | + |
| - | - | - | - | + | + |
PMA
IL-4
Irisin
Genistein

## Slide 6
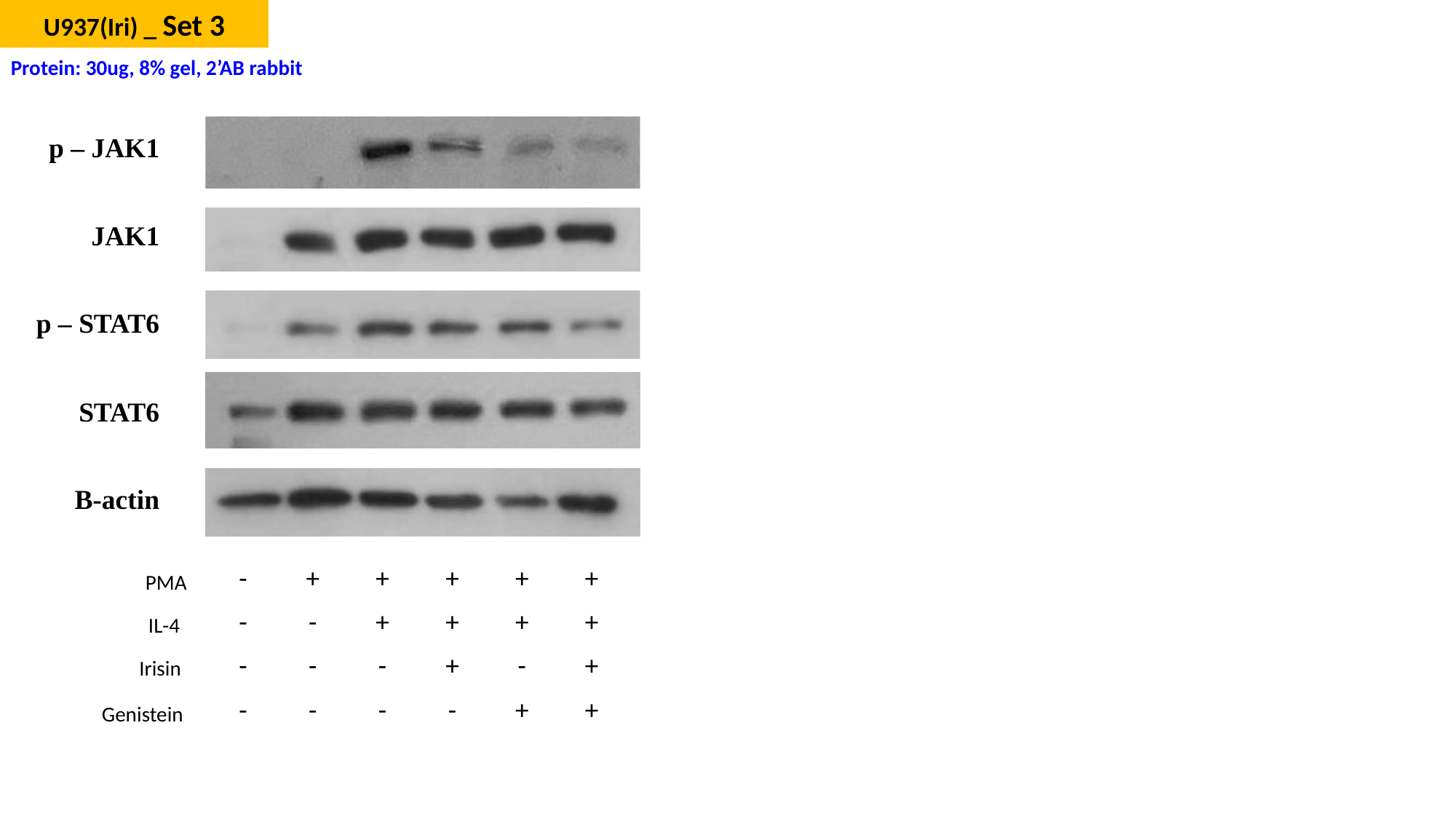

U937(Iri) _ Set 3
Protein: 30ug, 8% gel, 2’AB rabbit
p – JAK1
JAK1
p – STAT6
STAT6
B-actin
| - | + | + | + | + | + |
| --- | --- | --- | --- | --- | --- |
| - | - | + | + | + | + |
| - | - | - | + | - | + |
| - | - | - | - | + | + |
PMA
IL-4
Irisin
Genistein

## Slide 7
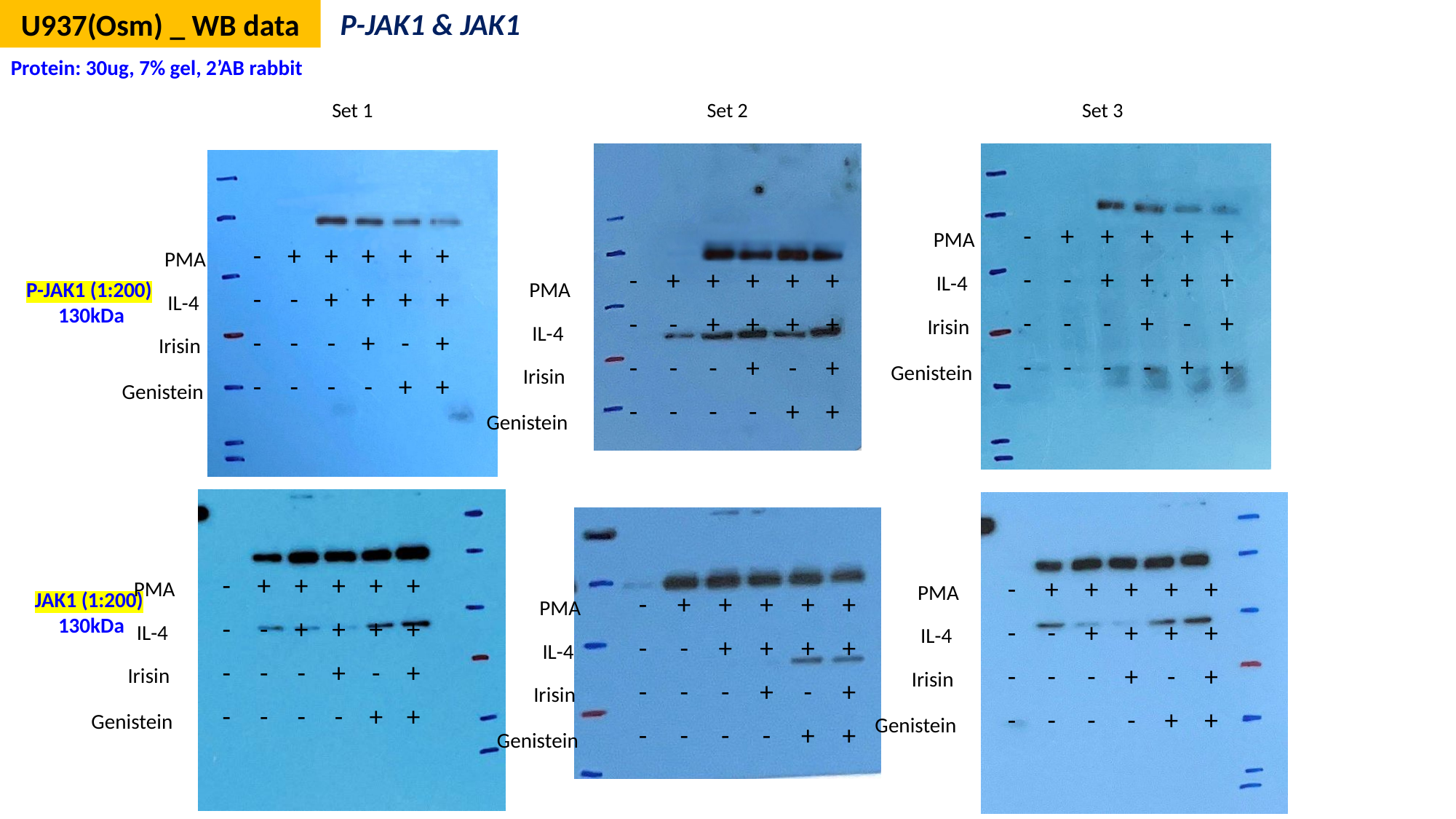

P-JAK1 & JAK1
U937(Osm) _ WB data
Protein: 30ug, 7% gel, 2’AB rabbit
Set 1
Set 2
Set 3
| - | + | + | + | + | + |
| --- | --- | --- | --- | --- | --- |
| - | - | + | + | + | + |
| - | - | - | + | - | + |
| - | - | - | - | + | + |
PMA
IL-4
Irisin
Genistein
| - | + | + | + | + | + |
| --- | --- | --- | --- | --- | --- |
| - | - | + | + | + | + |
| - | - | - | + | - | + |
| - | - | - | - | + | + |
PMA
IL-4
Irisin
Genistein
| - | + | + | + | + | + |
| --- | --- | --- | --- | --- | --- |
| - | - | + | + | + | + |
| - | - | - | + | - | + |
| - | - | - | - | + | + |
P-JAK1 (1:200)
130kDa
PMA
IL-4
Irisin
Genistein
| - | + | + | + | + | + |
| --- | --- | --- | --- | --- | --- |
| - | - | + | + | + | + |
| - | - | - | + | - | + |
| - | - | - | - | + | + |
PMA
IL-4
Irisin
Genistein
| - | + | + | + | + | + |
| --- | --- | --- | --- | --- | --- |
| - | - | + | + | + | + |
| - | - | - | + | - | + |
| - | - | - | - | + | + |
PMA
IL-4
Irisin
Genistein
JAK1 (1:200)
130kDa
| - | + | + | + | + | + |
| --- | --- | --- | --- | --- | --- |
| - | - | + | + | + | + |
| - | - | - | + | - | + |
| - | - | - | - | + | + |
PMA
IL-4
Irisin
Genistein

## Slide 8
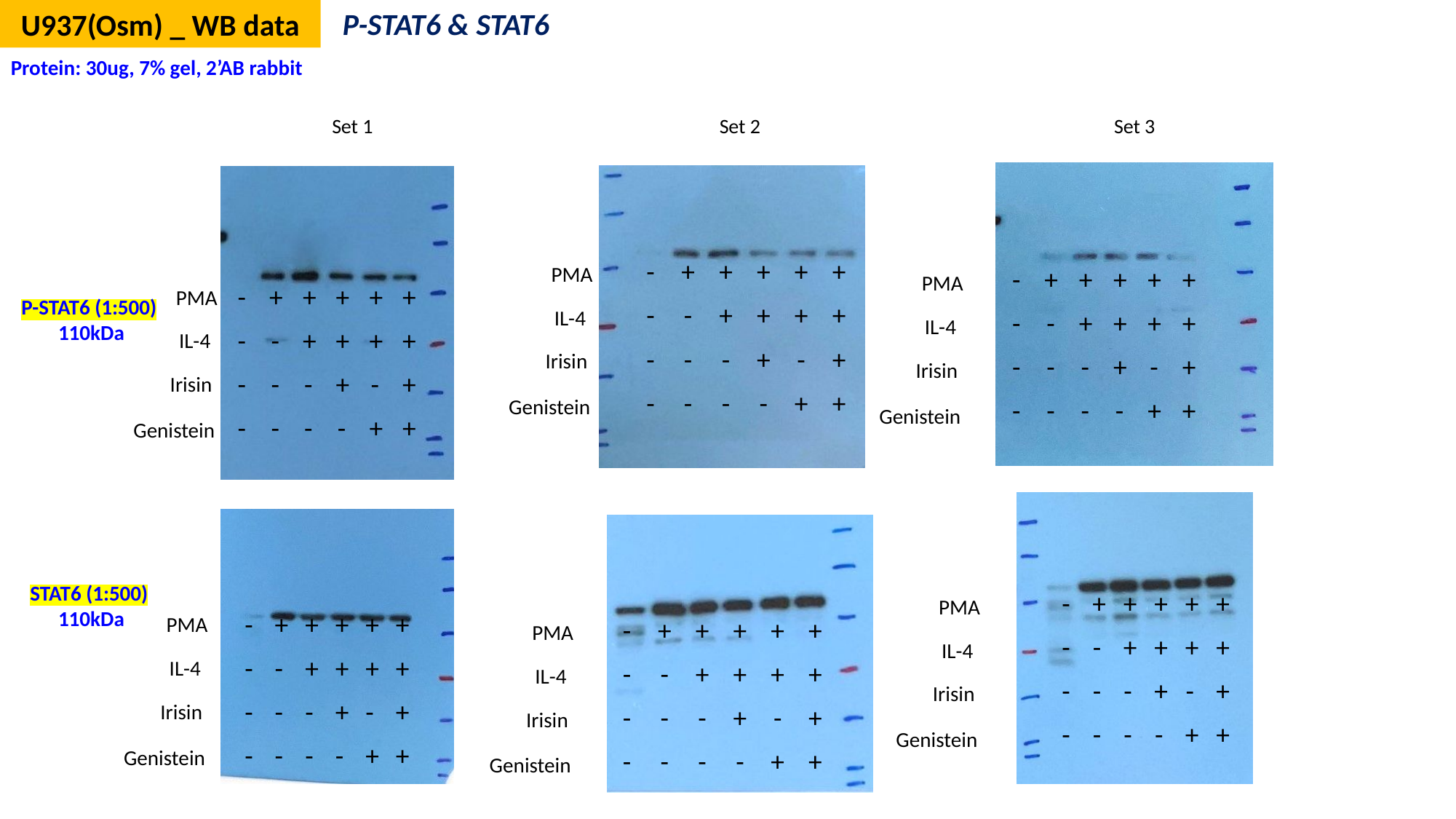

P-STAT6 & STAT6
U937(Osm) _ WB data
Protein: 30ug, 7% gel, 2’AB rabbit
Set 1
Set 2
Set 3
PMA
IL-4
Irisin
Genistein
| - | + | + | + | + | + |
| --- | --- | --- | --- | --- | --- |
| - | - | + | + | + | + |
| - | - | - | + | - | + |
| - | - | - | - | + | + |
| - | + | + | + | + | + |
| --- | --- | --- | --- | --- | --- |
| - | - | + | + | + | + |
| - | - | - | + | - | + |
| - | - | - | - | + | + |
PMA
IL-4
Irisin
Genistein
PMA
IL-4
Irisin
Genistein
| - | + | + | + | + | + |
| --- | --- | --- | --- | --- | --- |
| - | - | + | + | + | + |
| - | - | - | + | - | + |
| - | - | - | - | + | + |
P-STAT6 (1:500)
110kDa
STAT6 (1:500)
110kDa
| - | + | + | + | + | + |
| --- | --- | --- | --- | --- | --- |
| - | - | + | + | + | + |
| - | - | - | + | - | + |
| - | - | - | - | + | + |
PMA
IL-4
Irisin
Genistein
PMA
IL-4
Irisin
Genistein
| - | + | + | + | + | + |
| --- | --- | --- | --- | --- | --- |
| - | - | + | + | + | + |
| - | - | - | + | - | + |
| - | - | - | - | + | + |
PMA
IL-4
Irisin
Genistein
| - | + | + | + | + | + |
| --- | --- | --- | --- | --- | --- |
| - | - | + | + | + | + |
| - | - | - | + | - | + |
| - | - | - | - | + | + |

## Slide 9
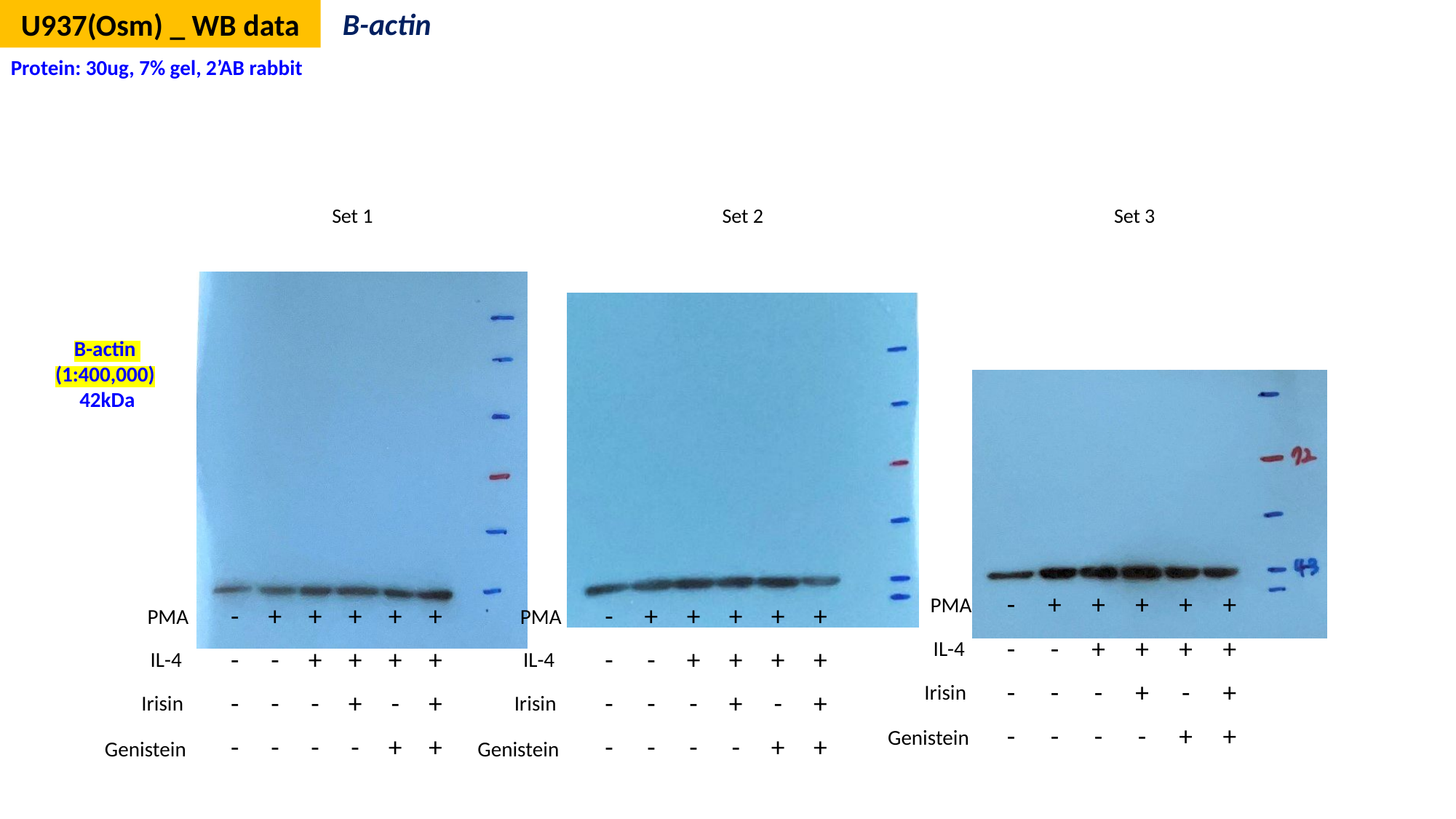

B-actin
U937(Osm) _ WB data
Protein: 30ug, 7% gel, 2’AB rabbit
Set 1
Set 2
Set 3
B-actin
(1:400,000)
42kDa
PMA
IL-4
Irisin
Genistein
| - | + | + | + | + | + |
| --- | --- | --- | --- | --- | --- |
| - | - | + | + | + | + |
| - | - | - | + | - | + |
| - | - | - | - | + | + |
PMA
IL-4
Irisin
Genistein
PMA
IL-4
Irisin
Genistein
| - | + | + | + | + | + |
| --- | --- | --- | --- | --- | --- |
| - | - | + | + | + | + |
| - | - | - | + | - | + |
| - | - | - | - | + | + |
| - | + | + | + | + | + |
| --- | --- | --- | --- | --- | --- |
| - | - | + | + | + | + |
| - | - | - | + | - | + |
| - | - | - | - | + | + |

## Slide 10
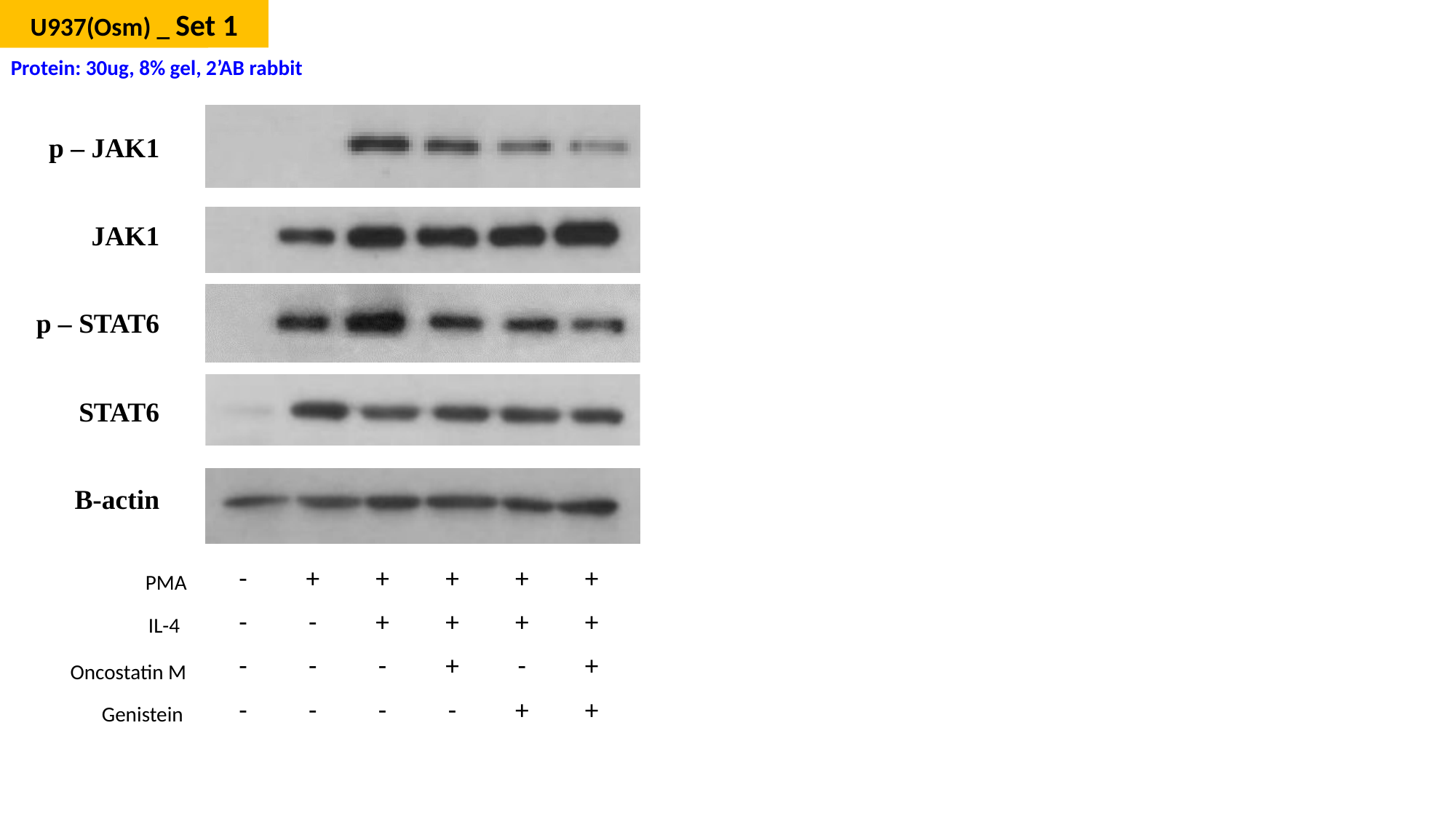

Set 1
U937(Osm) _ Set 1
Protein: 30ug, 8% gel, 2’AB rabbit
p – JAK1
JAK1
p – STAT6
STAT6
B-actin
| - | + | + | + | + | + |
| --- | --- | --- | --- | --- | --- |
| - | - | + | + | + | + |
| - | - | - | + | - | + |
| - | - | - | - | + | + |
PMA
IL-4
Oncostatin M
Genistein

## Slide 11
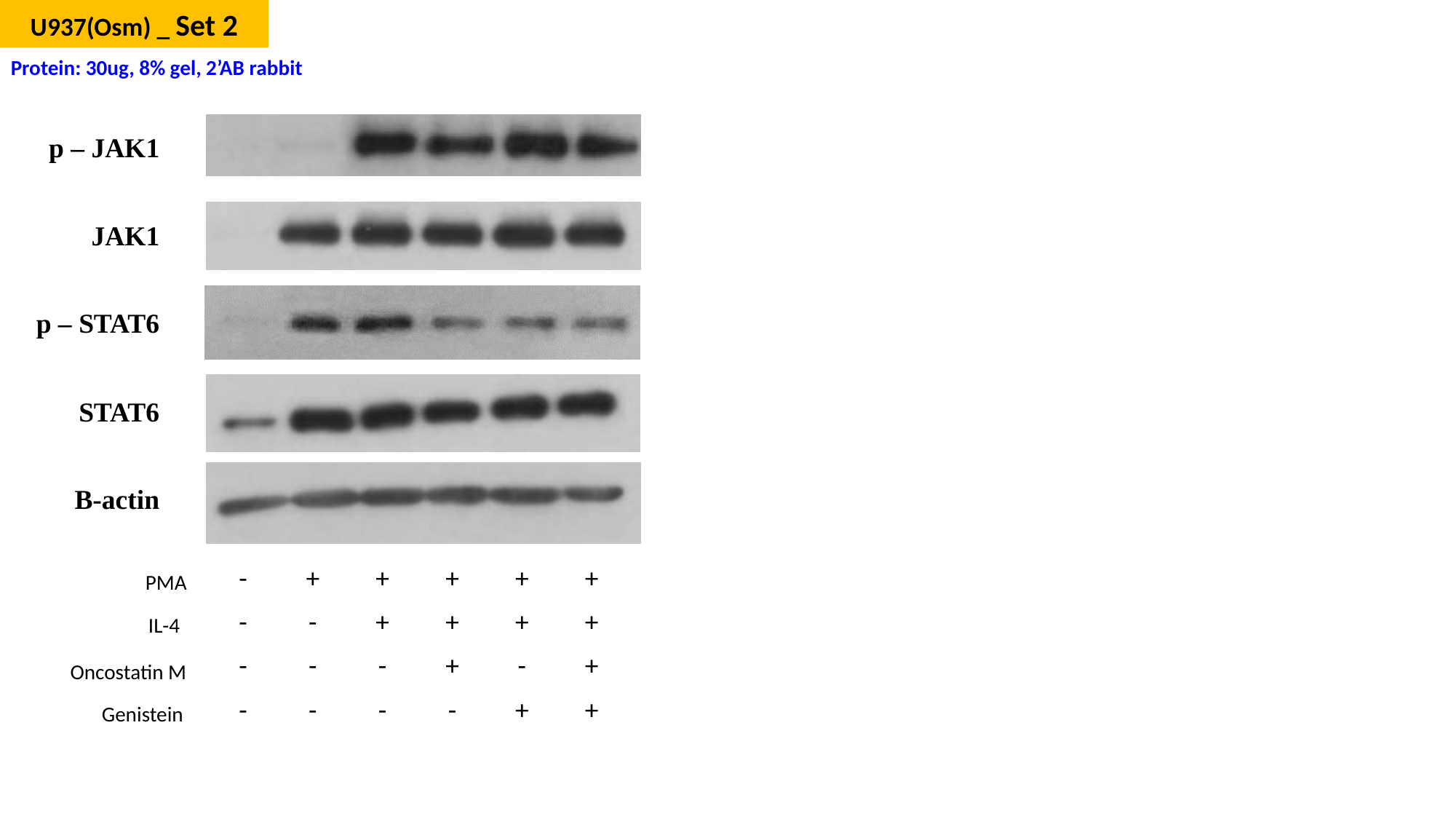

U937(Osm) _ Set 2
Protein: 30ug, 8% gel, 2’AB rabbit
p – JAK1
JAK1
p – STAT6
STAT6
B-actin
| - | + | + | + | + | + |
| --- | --- | --- | --- | --- | --- |
| - | - | + | + | + | + |
| - | - | - | + | - | + |
| - | - | - | - | + | + |
PMA
IL-4
Oncostatin M
Genistein

## Slide 12
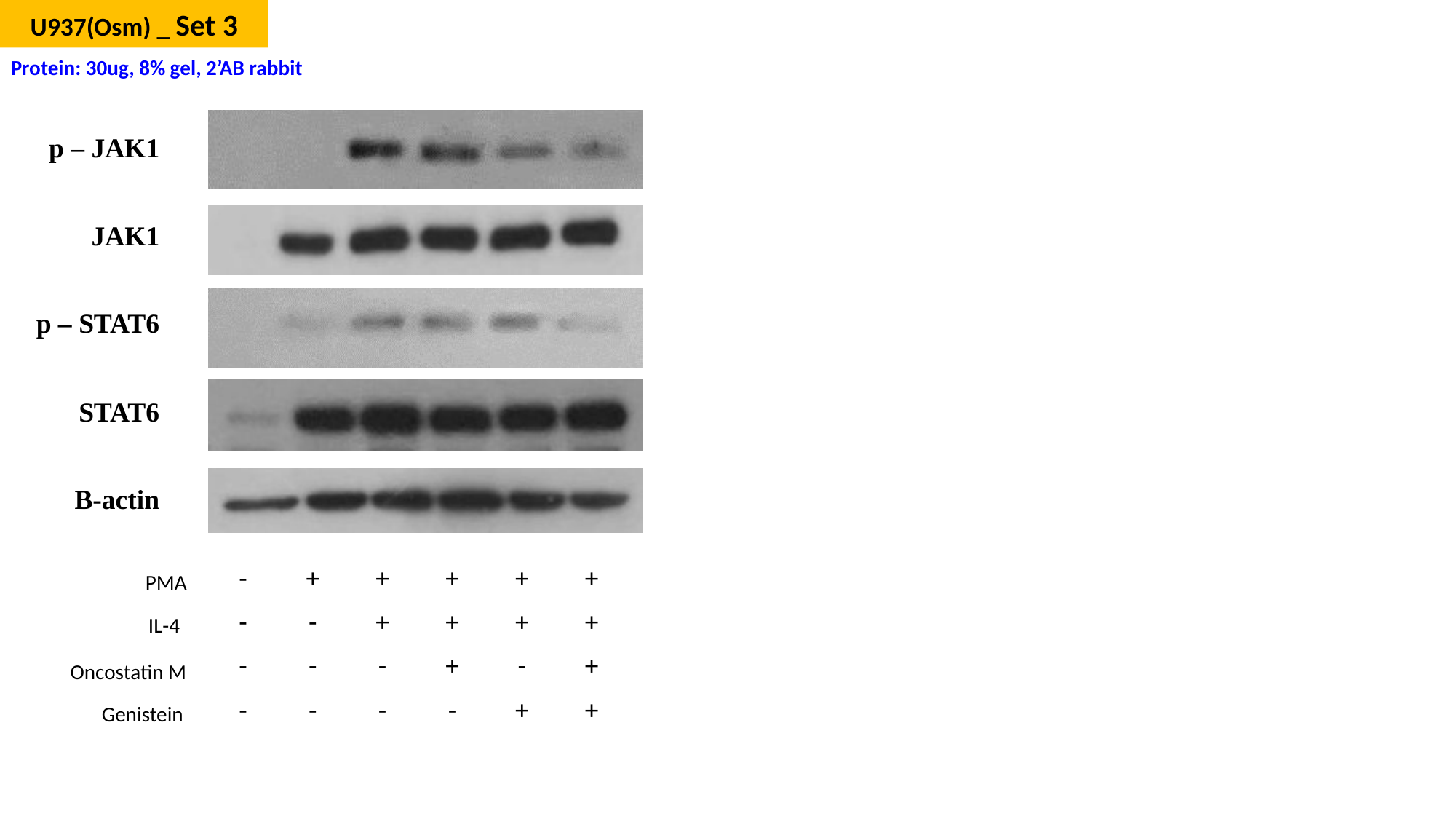

U937(Osm) _ Set 3
Protein: 30ug, 8% gel, 2’AB rabbit
p – JAK1
JAK1
p – STAT6
STAT6
B-actin
| - | + | + | + | + | + |
| --- | --- | --- | --- | --- | --- |
| - | - | + | + | + | + |
| - | - | - | + | - | + |
| - | - | - | - | + | + |
PMA
IL-4
Oncostatin M
Genistein

## Slide 13
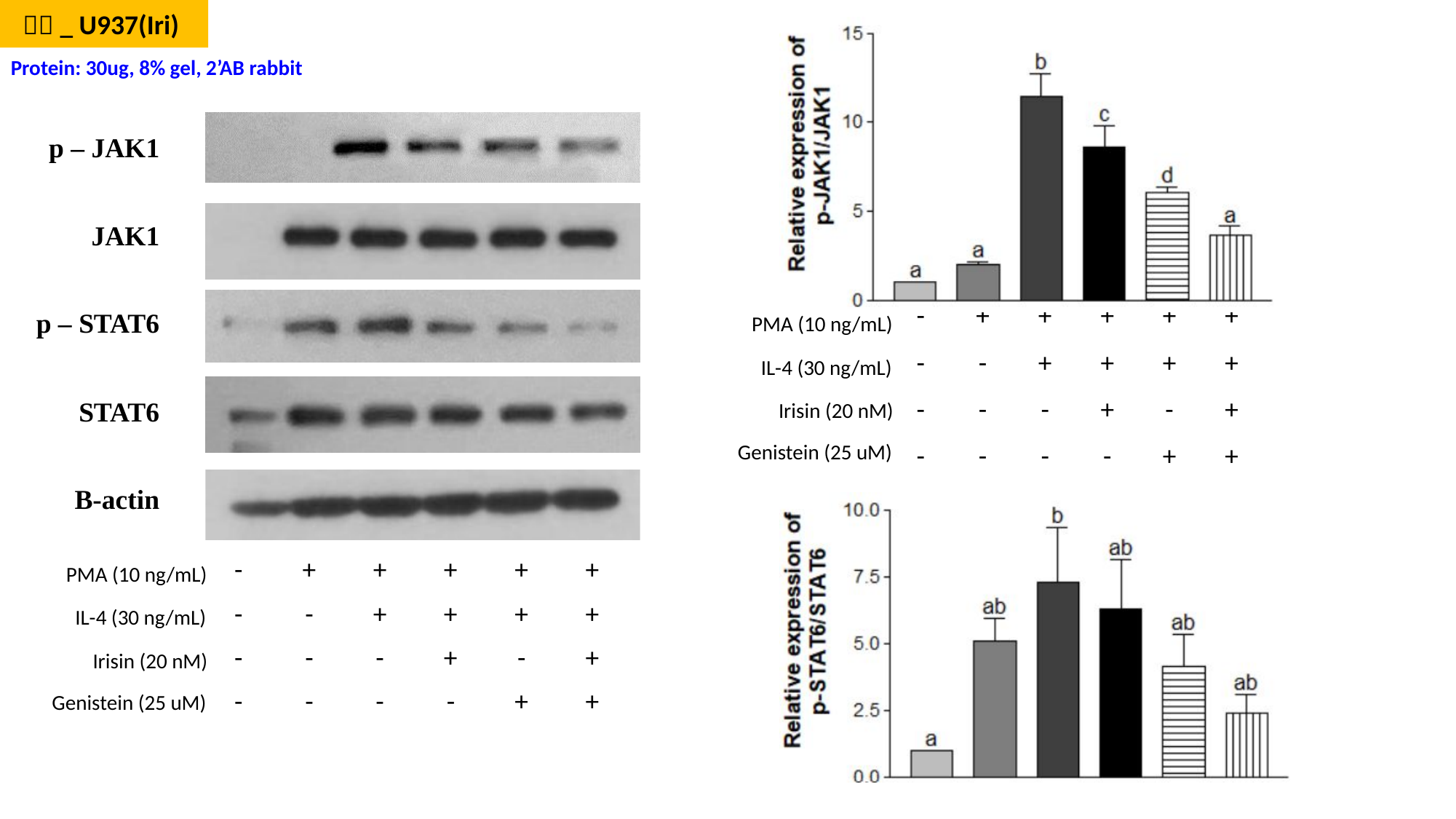

대표_ U937(Iri)
Protein: 30ug, 8% gel, 2’AB rabbit
p – JAK1
JAK1
| - | + | + | + | + | + |
| --- | --- | --- | --- | --- | --- |
| - | - | + | + | + | + |
| - | - | - | + | - | + |
| - | - | - | - | + | + |
p – STAT6
PMA (10 ng/mL)
IL-4 (30 ng/mL)
STAT6
Irisin (20 nM)
Genistein (25 uM)
B-actin
| - | + | + | + | + | + |
| --- | --- | --- | --- | --- | --- |
| - | - | + | + | + | + |
| - | - | - | + | - | + |
| - | - | - | - | + | + |
PMA (10 ng/mL)
IL-4 (30 ng/mL)
Irisin (20 nM)
Genistein (25 uM)

## Slide 14
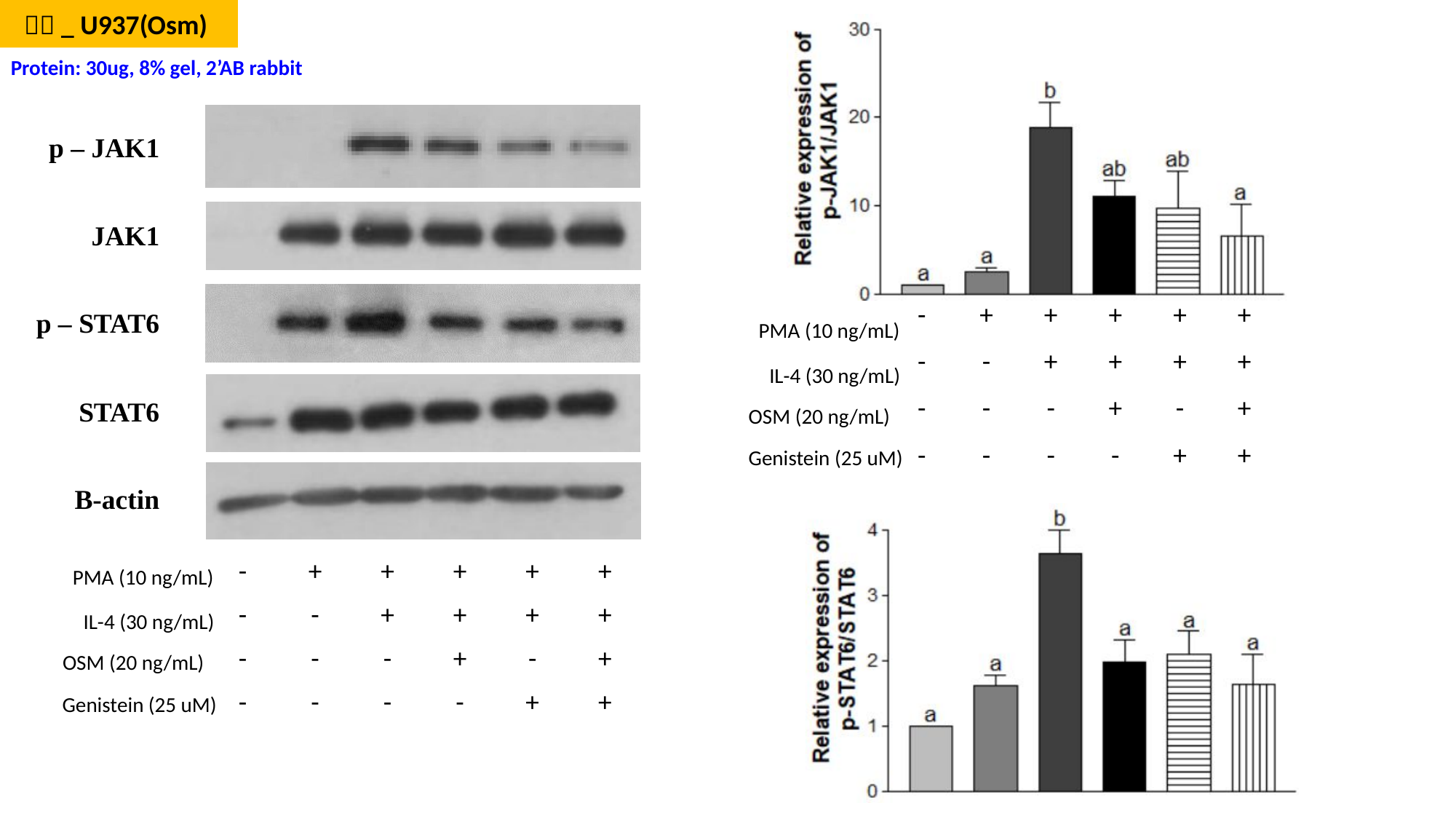

대표_ U937(Osm)
Protein: 30ug, 8% gel, 2’AB rabbit
p – JAK1
JAK1
| - | + | + | + | + | + |
| --- | --- | --- | --- | --- | --- |
| - | - | + | + | + | + |
| - | - | - | + | - | + |
| - | - | - | - | + | + |
p – STAT6
PMA (10 ng/mL)
IL-4 (30 ng/mL)
STAT6
OSM (20 ng/mL)
Genistein (25 uM)
B-actin
| - | + | + | + | + | + |
| --- | --- | --- | --- | --- | --- |
| - | - | + | + | + | + |
| - | - | - | + | - | + |
| - | - | - | - | + | + |
PMA (10 ng/mL)
IL-4 (30 ng/mL)
OSM (20 ng/mL)
Genistein (25 uM)
