# Supplementary material for: Anti-cancer effects of genistein supplementation and moderate-intensity exercise in high-fat diet-induced breast cancer via regulation of inflammation and adipose tissue metabolism in vivo and in vitro
Source: BMC Complement Med Ther. 2025 Jul 2;25:223. doi: 10.1186/s12906-025-04968-x (PMC12225189; doi:10.1186/s12906-025-04968-x)

Full-length blots of Figure 1. B.

PCNA Tumor

Protein: 30ug, 15% gel, 2'AB rabbit

PCNA (1:200)
36kDa

H, HG #1,2,3
HE, HGE #1

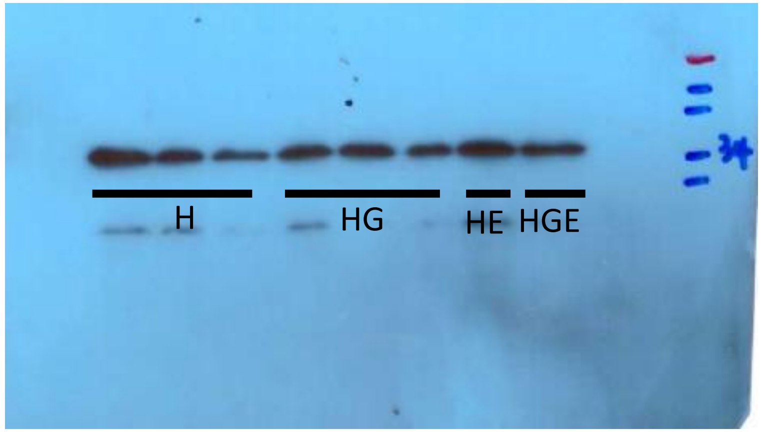

H, HG #4,5,6
HE, HGE #2

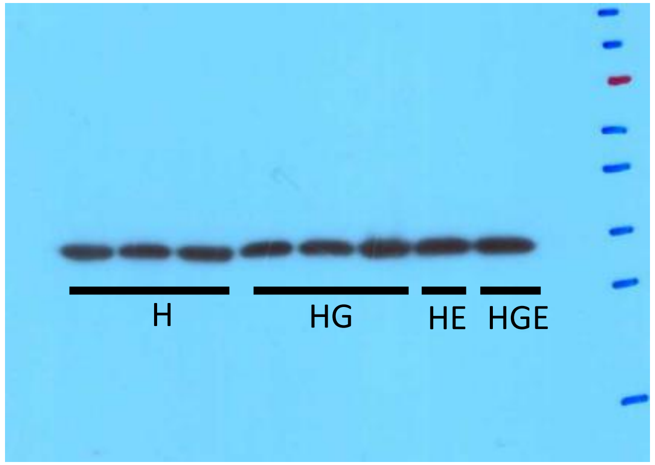

H #7,8,9,10
HG #7,8,9
HE, HGE #3

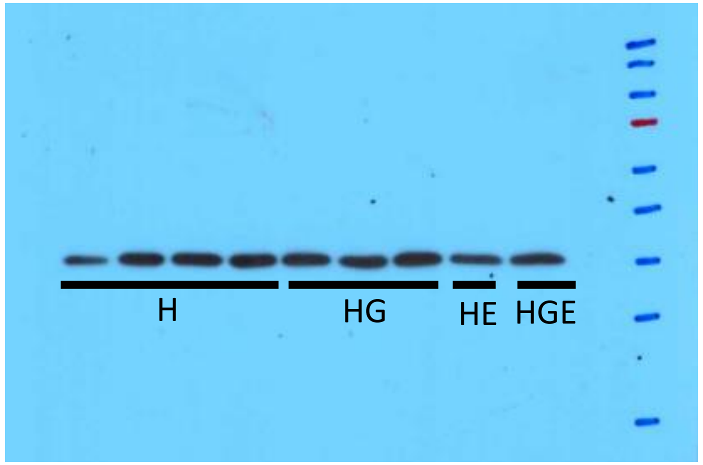

PCNA (1:200)
Representative image

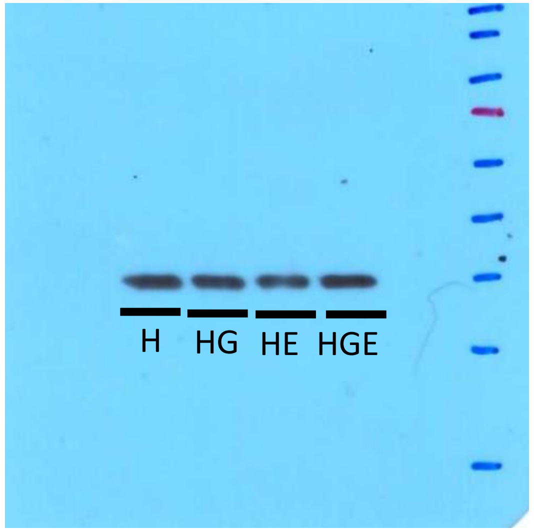

Full-length blots of **Figure 1. B.**

Bcl-2 Tumor

Protein: 30ug, 15% & 12% gel, 2'AB mouse

Bcl-2 (1:500)
26kDa

H, HG #1,2,3
HE, HGE #1

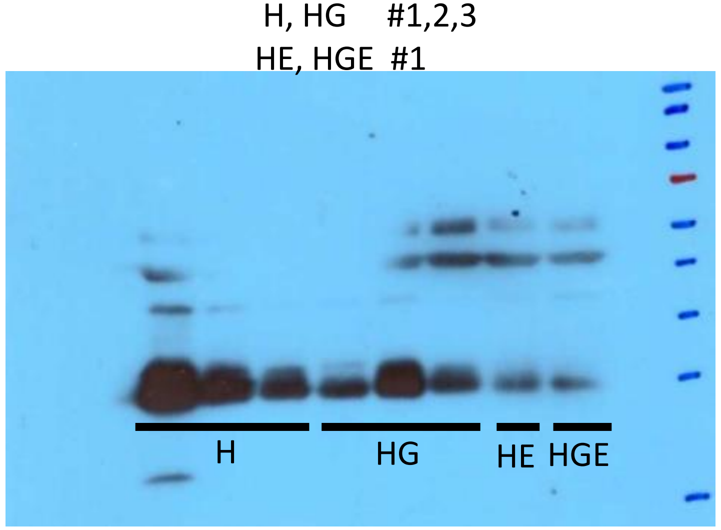

H, HG #4,5,6
HE, HGE #2

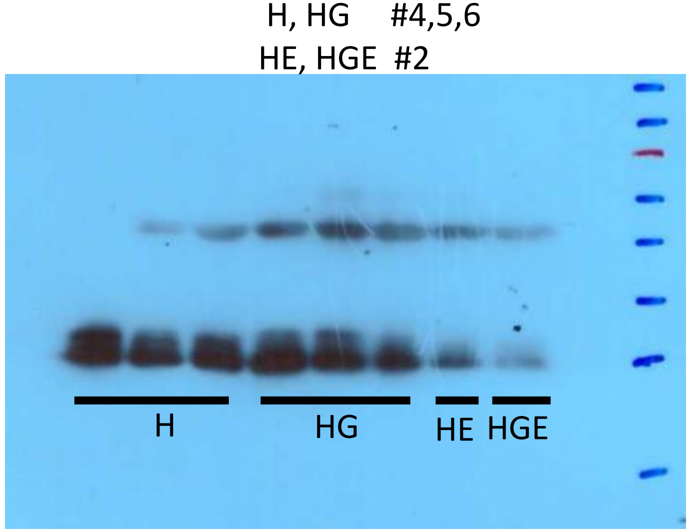

H #7,8,9,10
HG #7,8,9
HE, HGE #3

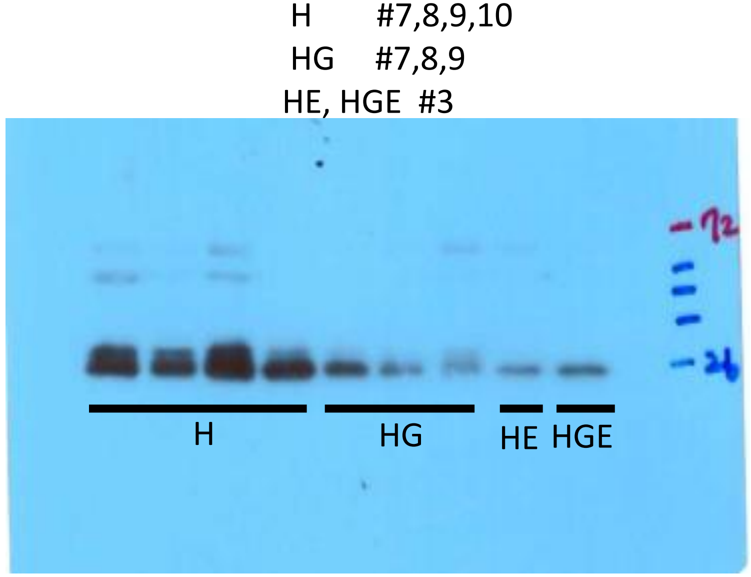

Bcl-2 (1:500)
Representative image

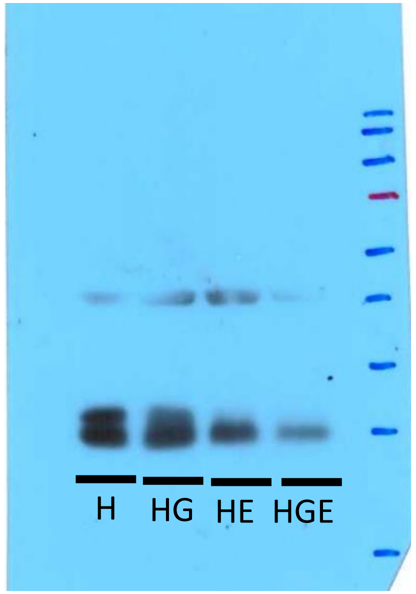

Full-length blots of **Figure 1. B.**

Bax Tumor

Protein: 30ug, 15% gel, 2'AB rabbit

Bax (1:2000)
21kDa

H, HG #1,2,3
HE, HGE #1

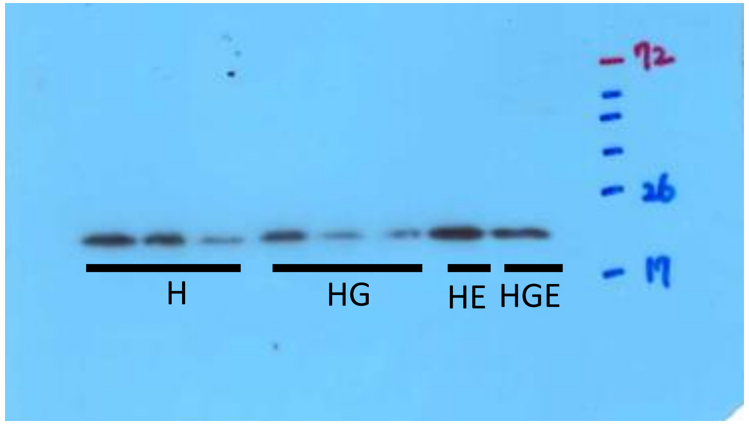

H, HG #4,5,6
HE, HGE #2

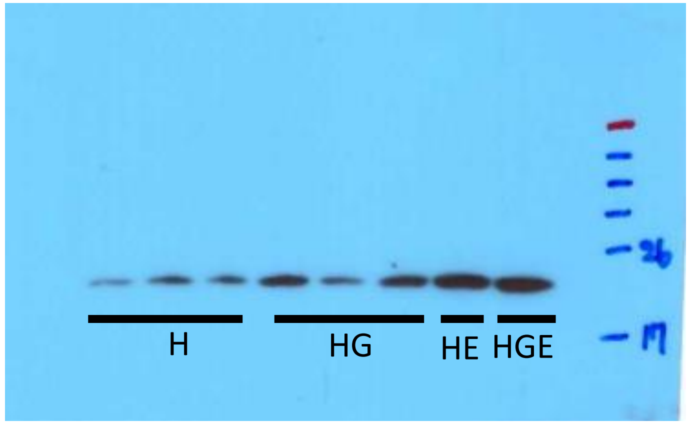

H #7,8,9,10
HG #7,8,9
HE, HGE #3

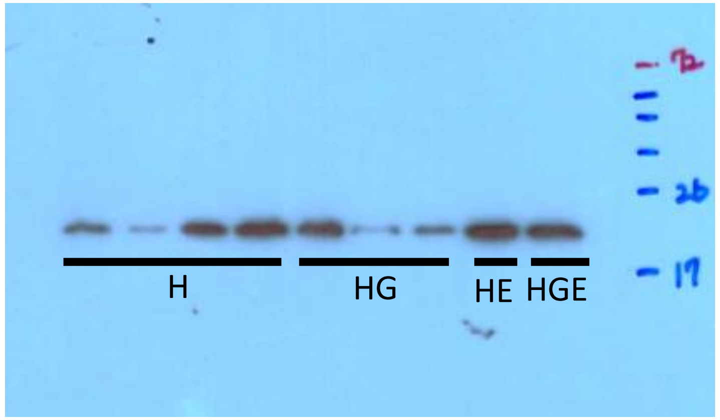

Bax (1:2000)
Representative image

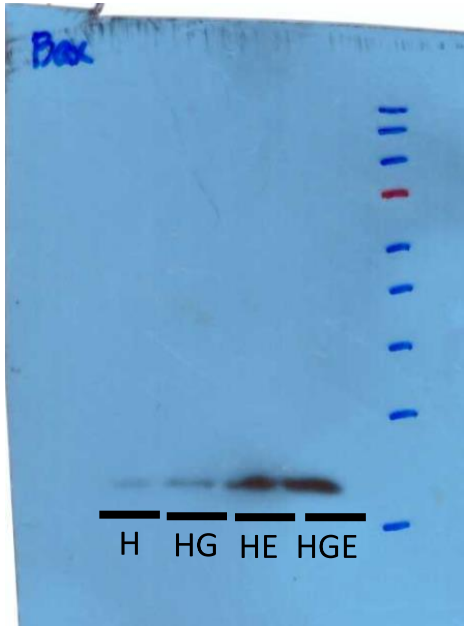

Full-length blots of Figure 1. B.

Cleaved caspase-3

Tumor

Protein: 30ug, 15% gel, 2'AB rabbit

Cleaved caspase-3 (1:1000)
17,19kDa

H, HG #1,2,3
HE, HGE #1

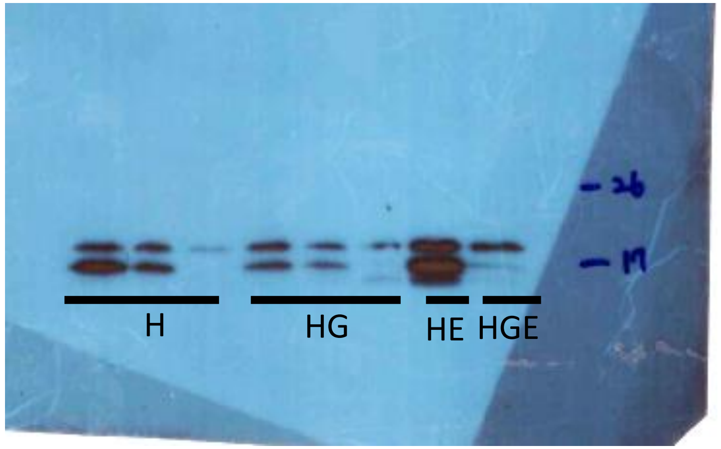

H, HG #4,5,6
HE, HGE #2

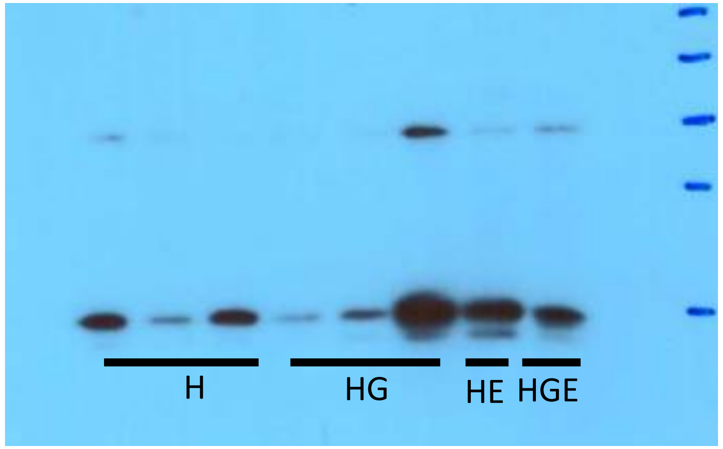

H #7,8,9,10
HG #7,8,9
HE, HGE #3

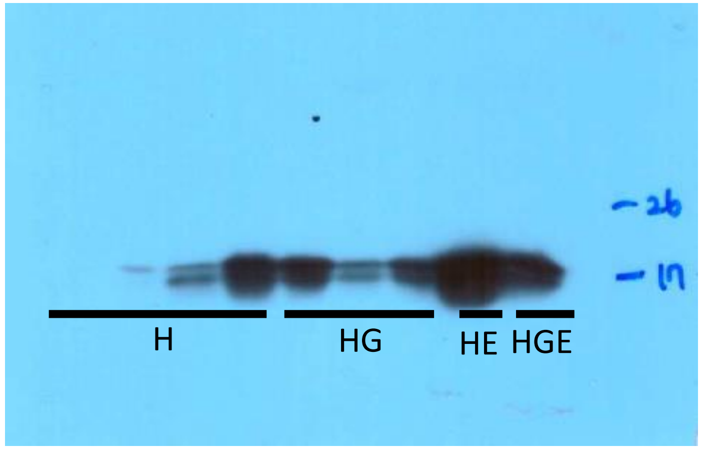

Cleaved caspase-3 (1:1000)
Representative image

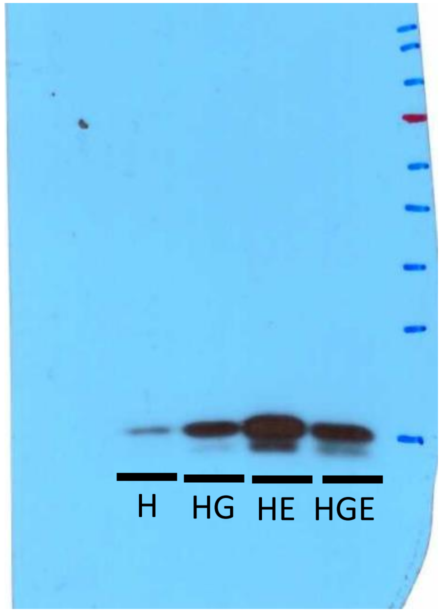

Full-length blots of Figure 1. B.

B-actin Tumor

Protein: 30ug, 15% & 12% gel, 2'AB mouse

H, HG #1,2,3
HE, HGE #1

H, HG #4,5,6
HE, HGE #2

H #7,8,9,10
HG #7,8,9
HE, HGE #3

B-actin (1:200,000)
42kDa

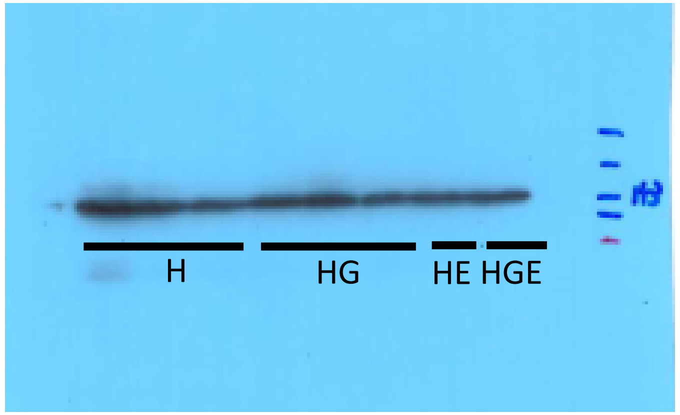

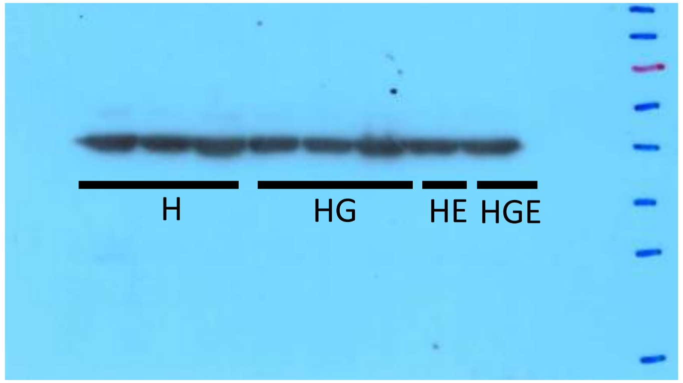

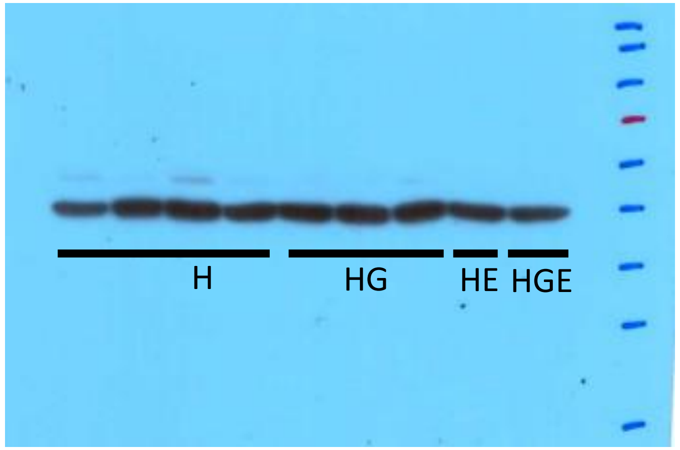

B-actin (1:200,000)
Representative image

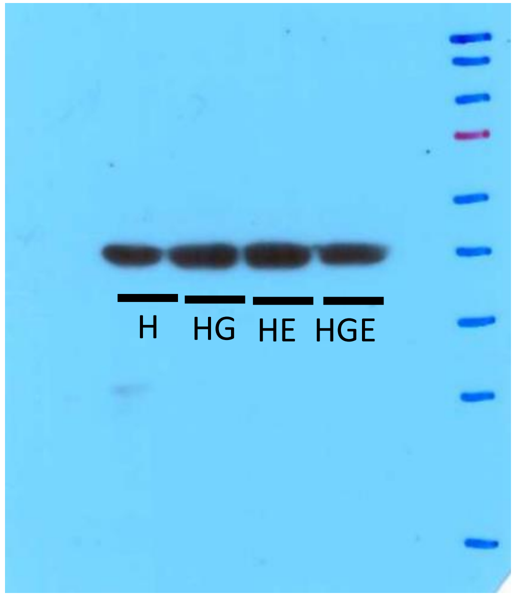

Full-length gels of Figure 2. B.

Cd68

Cd68
(28cycle)

H, HG #1,2,3
HE, HGE #1

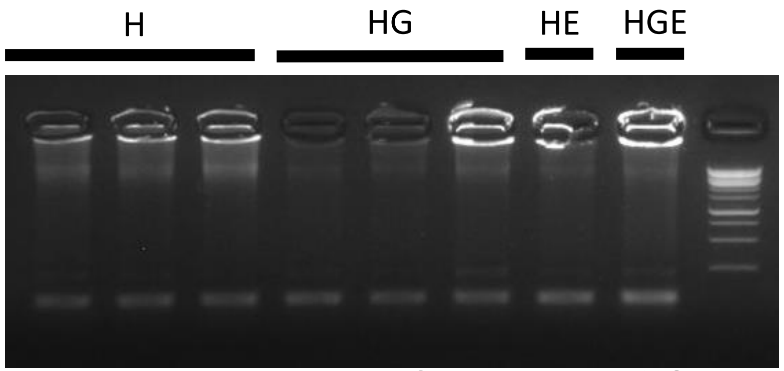

H, HG #4,5,6
HE, HGE #2

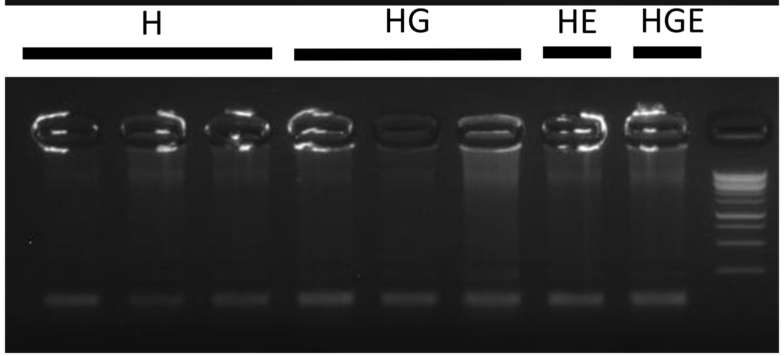

H #7,8,9,10
HG #7,8,9
HE, HGE #3

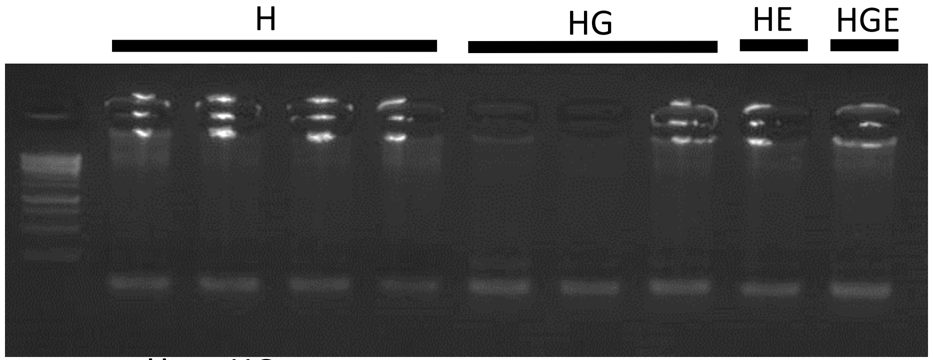

Representative image

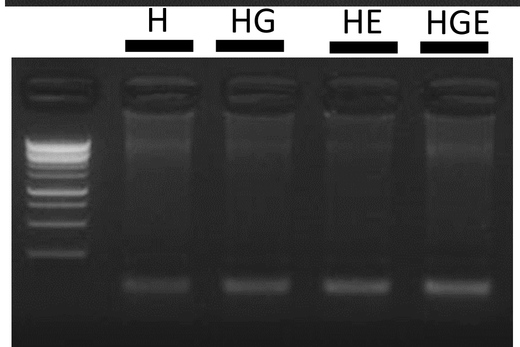

Full-length gels of Figure 2. B.

Cd163

Cd163
(28cycle)

H, HG #1,2,3
HE, HGE #1

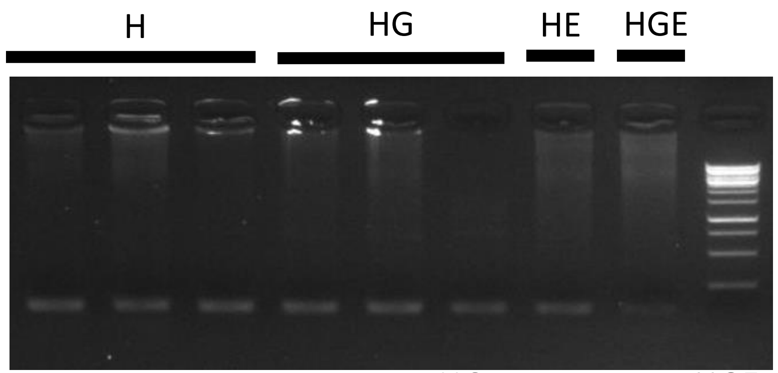

H, HG #4,5,6
HE, HGE #2

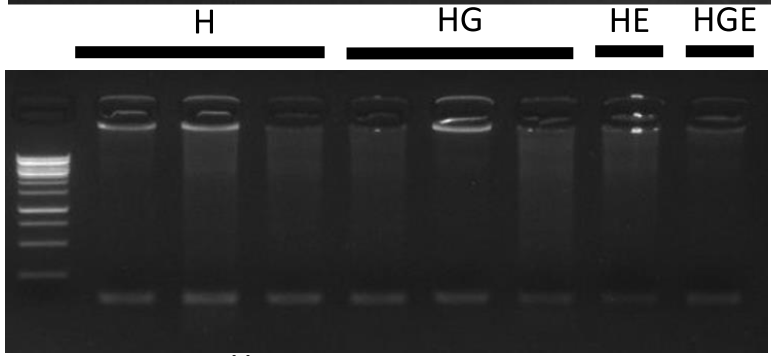

H #7,8,9,10
HG #7,8,9
HE, HGE #3

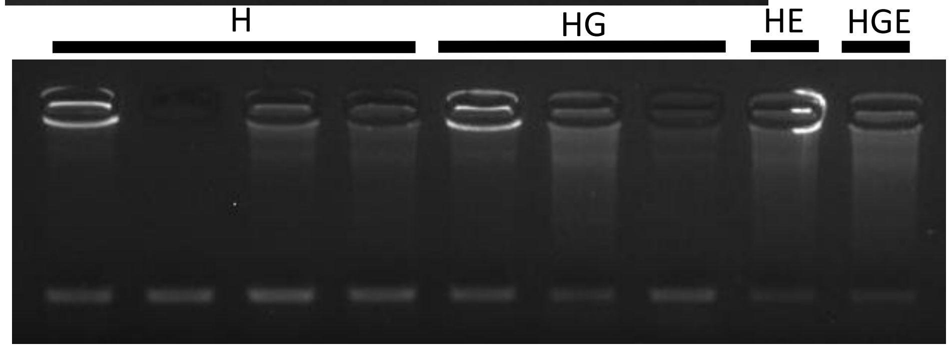

Representative image

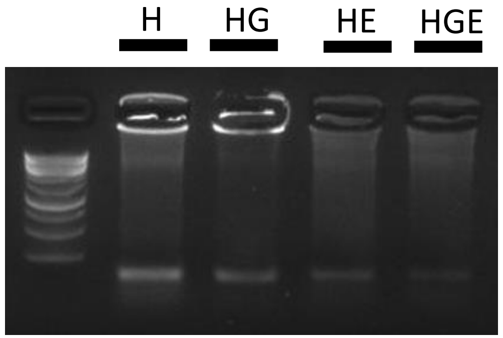

Full-length gels of Figure 2. B.

Arg1

H, HG #1,2,3
HE, HGE #1

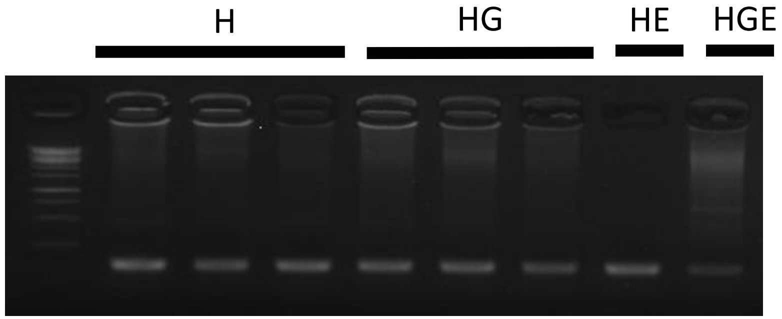

Arg1
(28cycle)

H, HG #4,5,6
HE, HGE #2

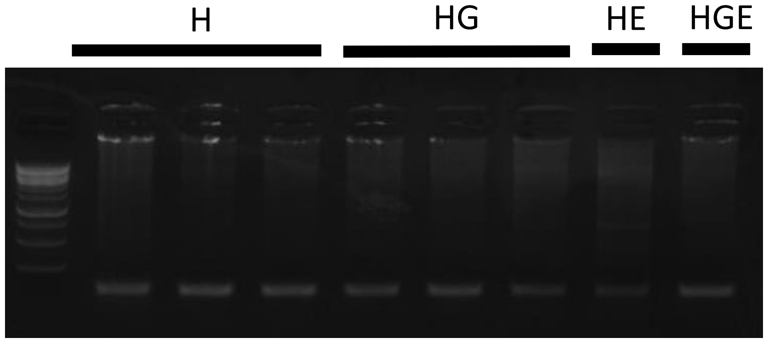

H #7,8,9,10
HG #7,8,9
HE, HGE #3

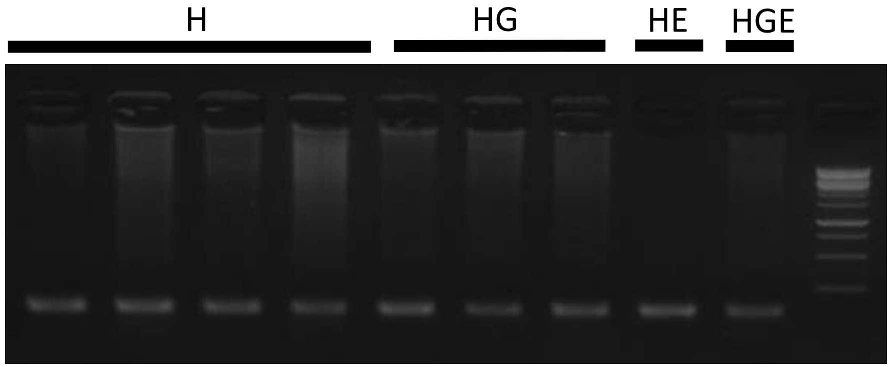

Representative image

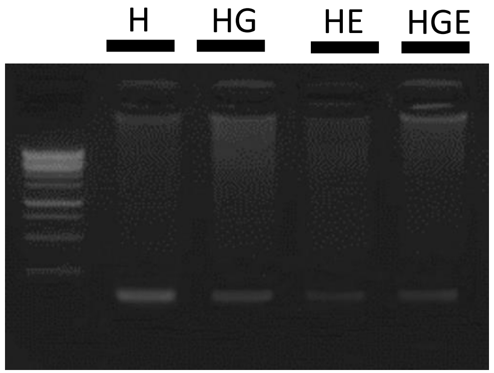

Full-length gels of Figure 2. B.

Gapdh

Gapdh
(27cycle)

H, HG #1,2,3
HE, HGE #1

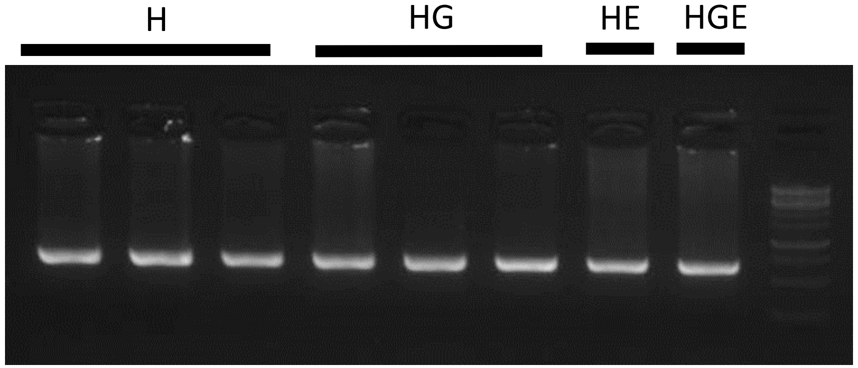

H, HG #4,5,6
HE, HGE #2

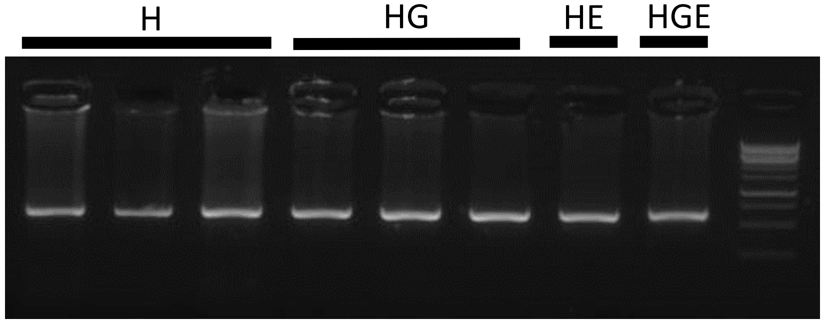

H #7,8,9,10
HG #7,8,9
HE, HGE #3

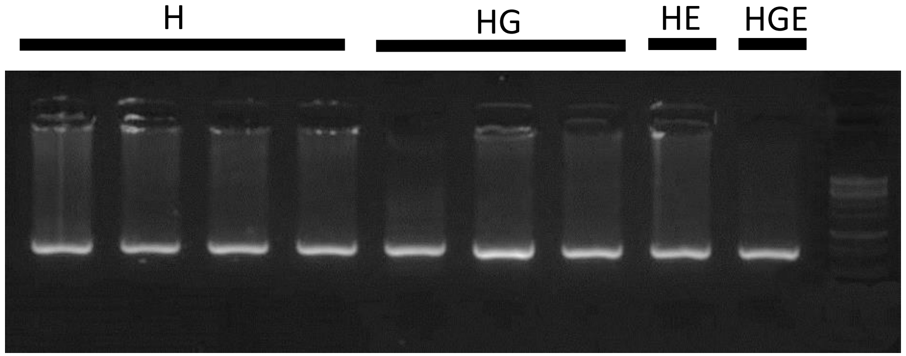

Representative image

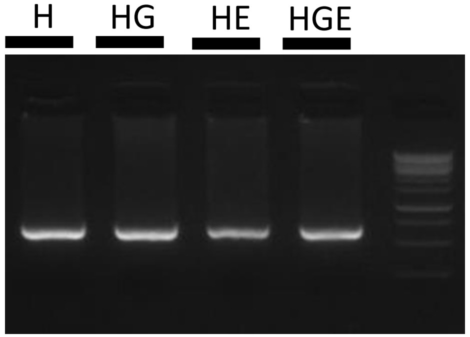

Full-length blots of Figure 2. C.

CD68 Tumor

Protein: 30ug, 15% gel, 2'AB rabbit

CD68 (1:10,000)
35kDa

H, HG #1,2,3
HE, HGE #1

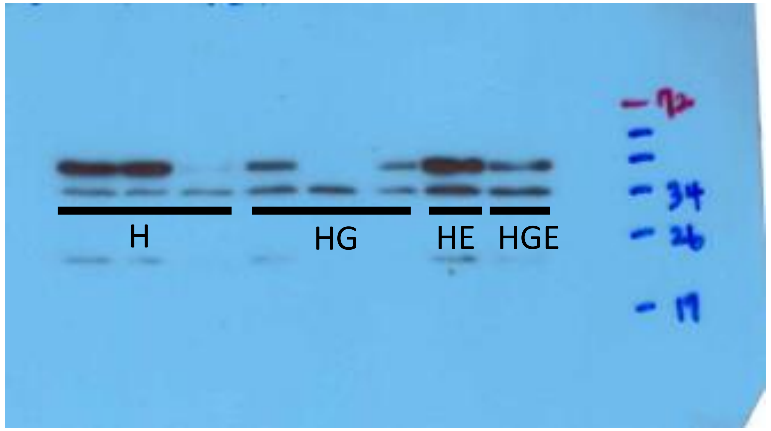

H, HG #4,5,6
HE, HGE #2

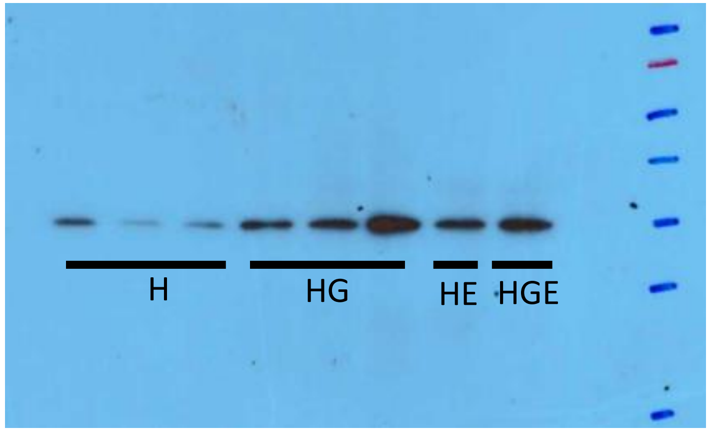

H #7,8,9,10
HG #7,8,9
HE, HGE #3

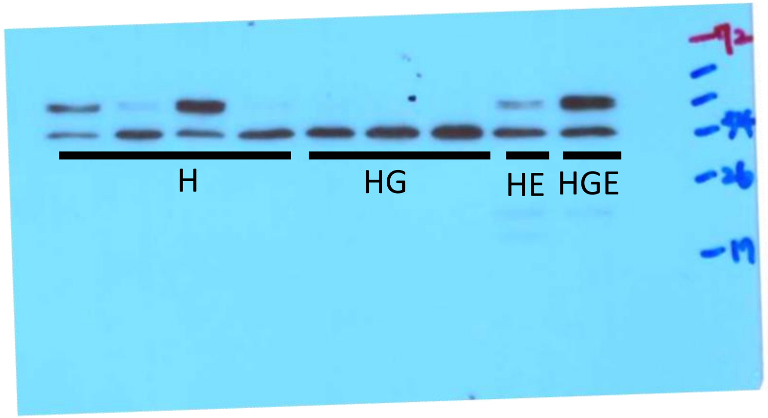

CD68 (1:10,000)
Representative image

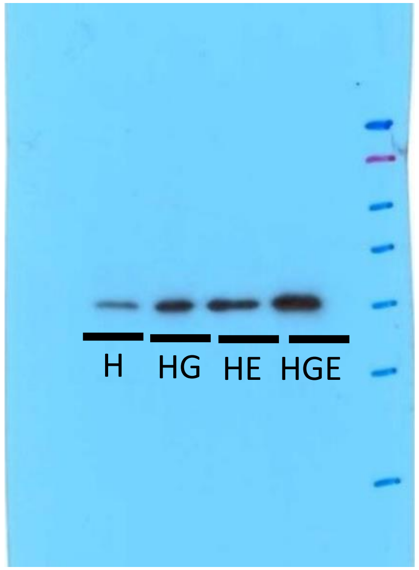

Full-length blots of Figure 2. C.

CD163 Tumor

Protein: 30ug, 7% gel, 2'AB rabbit

CD163 (1:1000)
150kDa

H, HG #1,2,3
HE, HGE #1

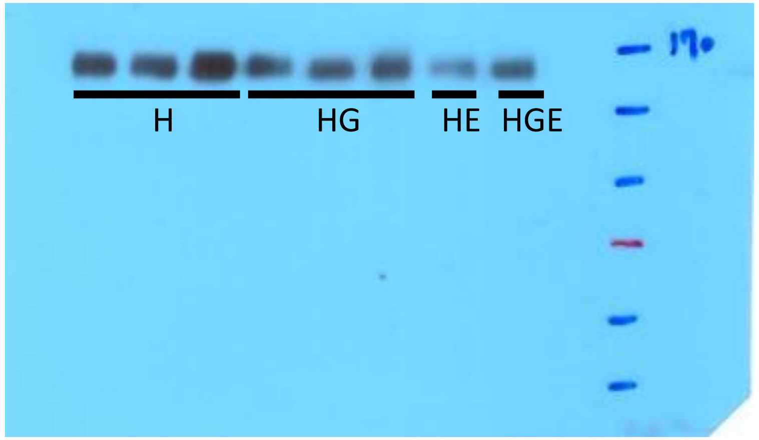

H, HG #4,5,6
HE, HGE #2

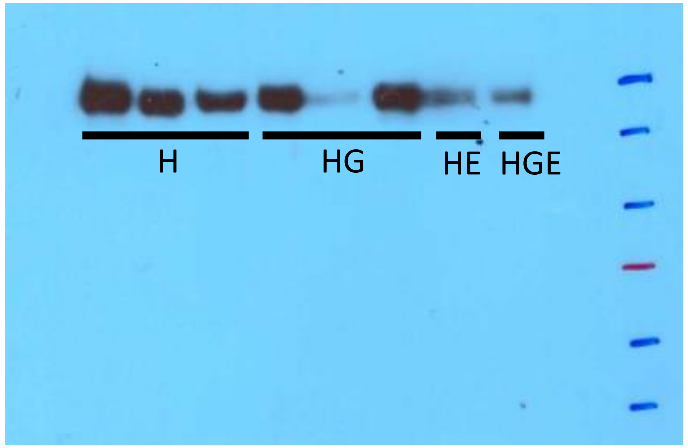

H #7,8,9,10
HG #7,8,9
HE, HGE #3

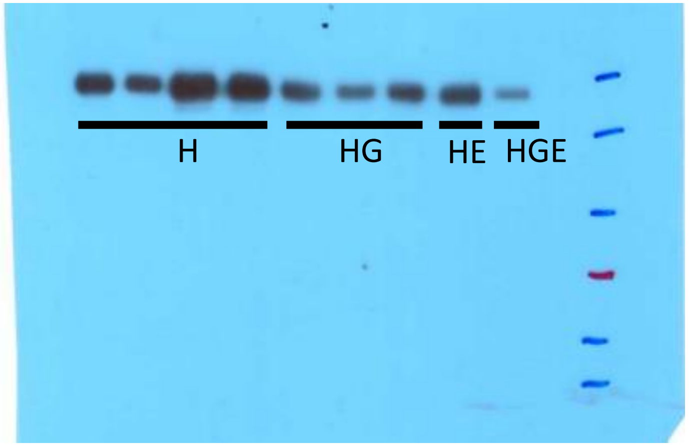

CD163 (1:1000)
Representative image

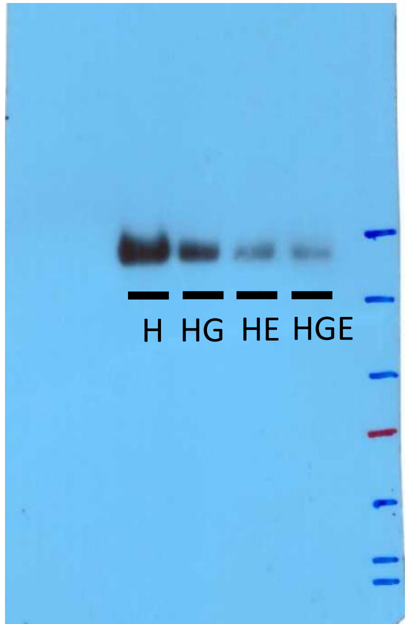

Full-length blots of Figure 2. C.

ARG1 Tumor

Protein: 30ug, 12% gel, 2'AB rabbit

H, HG #1,2,3
HE, HGE #1

H, HG #4,5,6
HE, HGE #2

H #7,8,9,10
HG #7,8,9
HE, HGE #3

Arg1 (1:5000)
35-36kDa

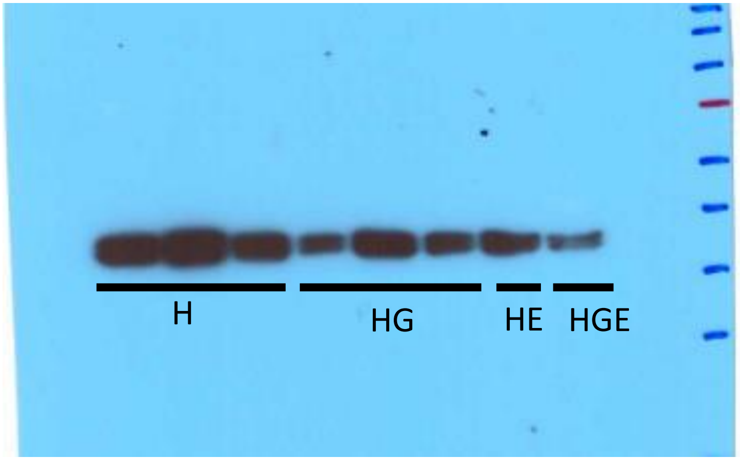

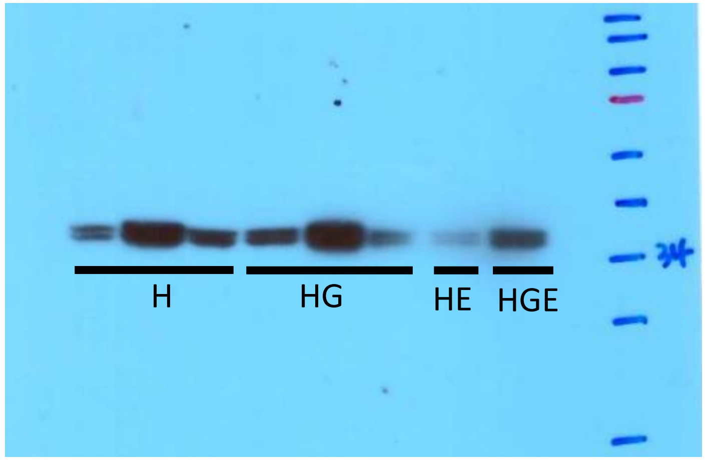

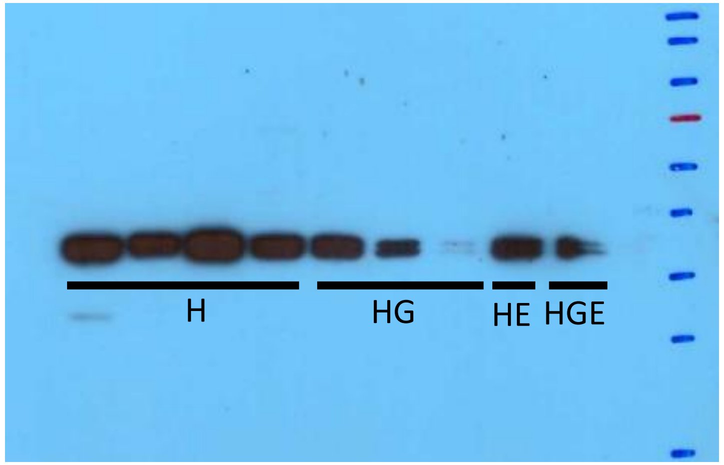

Arg1 (1:5000)
Representative image

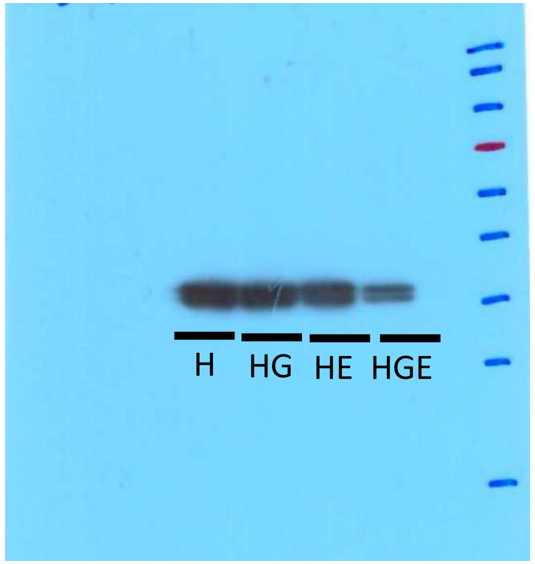

Full-length blots of Figure 2. C.

B-actin Tumor

Protein: 30ug, 12% gel, 2'AB rabbit

B-actin (1:400,000)
42kDa

H, HG #1,2,3
HE, HGE #1

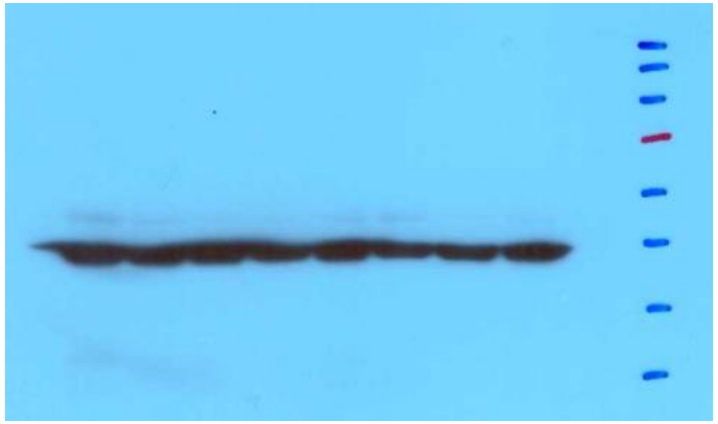

H, HG #4,5,6
HE, HGE #2

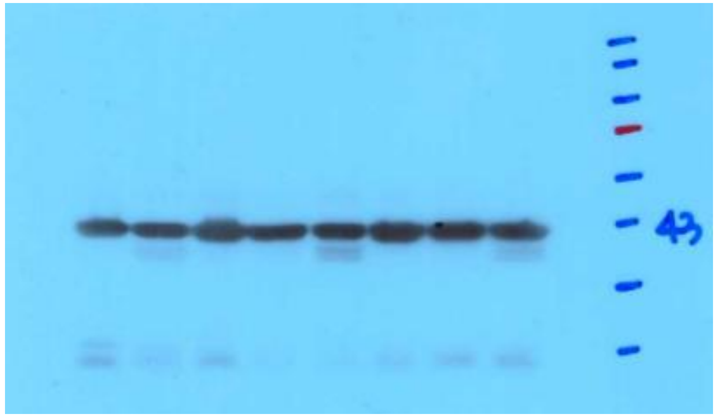

H #7,8,9,10
HG #7,8,9
HE, HGE #3

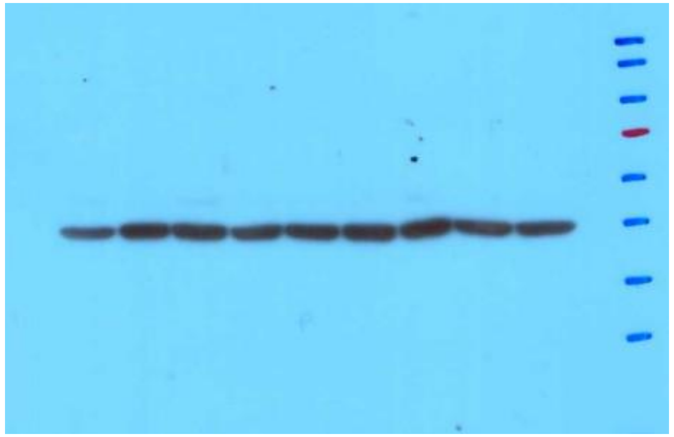

B-actin (1:400,000)
Representative image

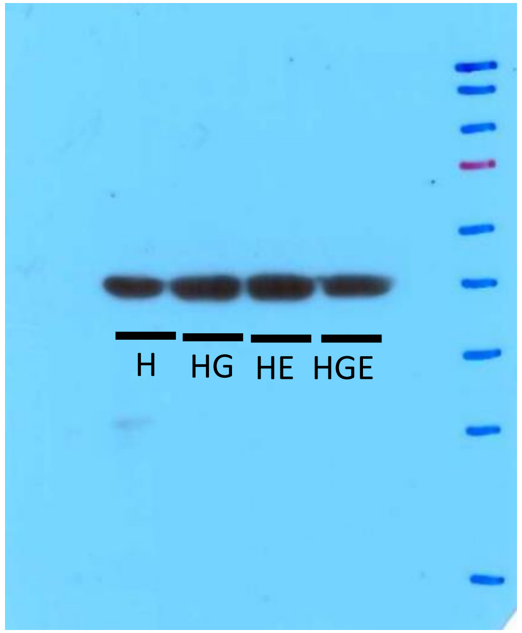

Full-length gels of Figure 3. B.

CD163 (Irisin)

CD163
(28cycle)

Set 1

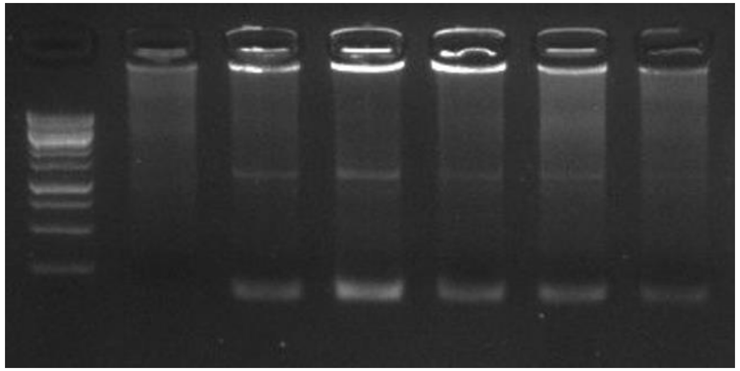

Set 2

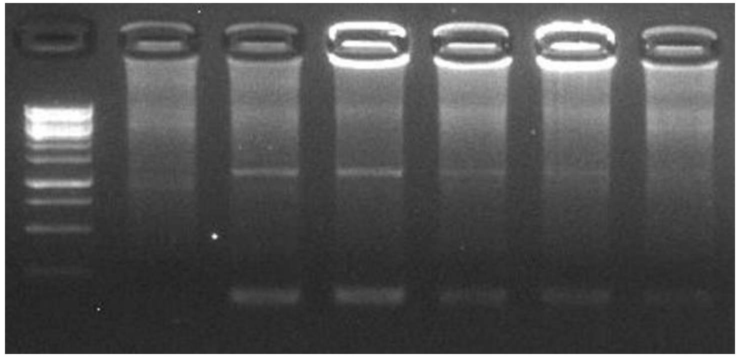

Set 3
(**Representative image**)

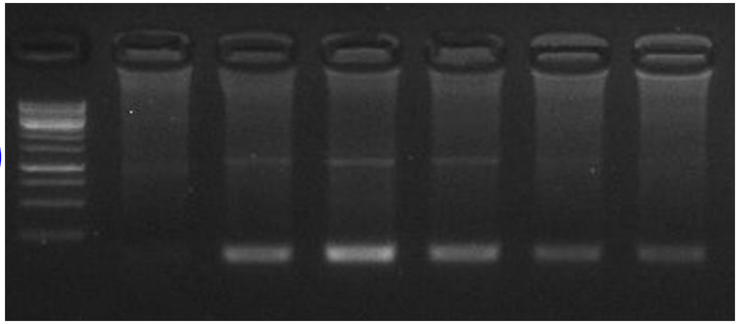

PMA (10 ng/mL)	-	+	+	+	+	+
IL-4 (30 ng/mL)	-	-	+	+	+	+
Irisin (20 nM)	-	-	-	+	-	+
Genistein (25 uM)	-	-	-	-	+	+

Full-length gels of Figure 3. B.

ARG1 (Irisin)

ARG1
(28cycle)

Set 1
(**Representative image**)

Set 2

Set 3

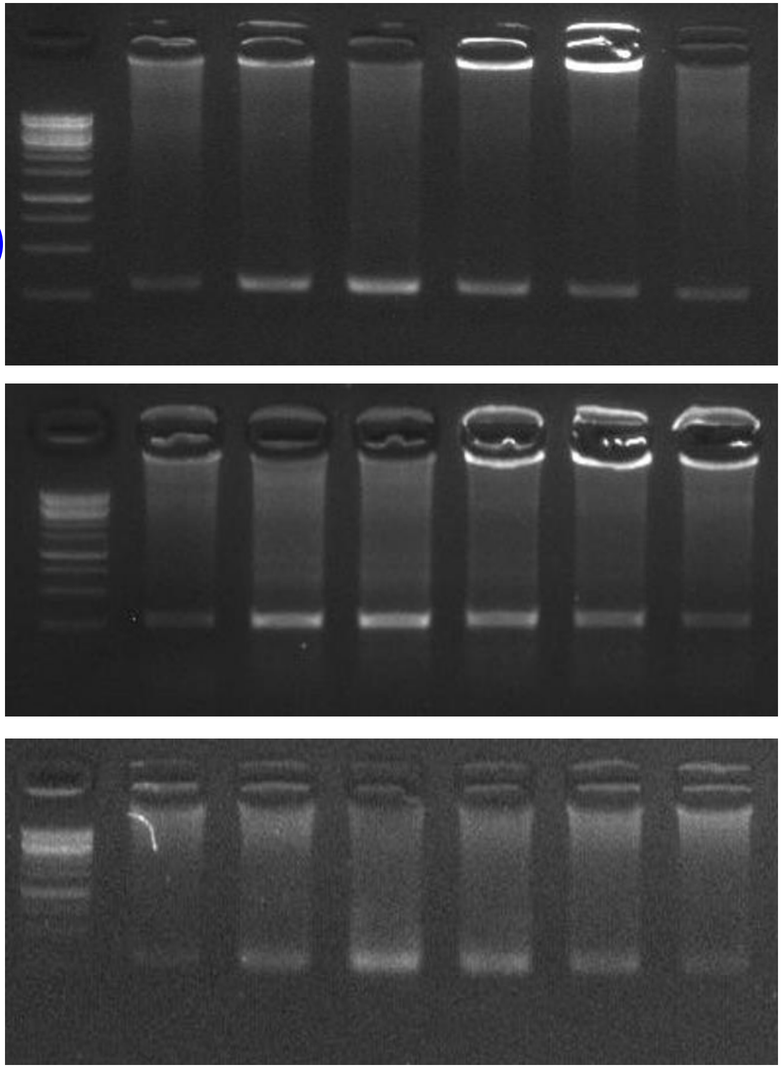

PMA (10 ng/mL)	-	+	+	+	+	+
IL-4 (30 ng/mL)	-	-	+	+	+	+
Irisin (20 nM)	-	-	-	+	-	+
Genistein (25 uM)	-	-	-	-	+	+

Full-length gels of Figure 3. B.

GAPDH (Irisin)

GAPDH
(28cycle)

Set 1
(Representative image)

Set 2

Set 3

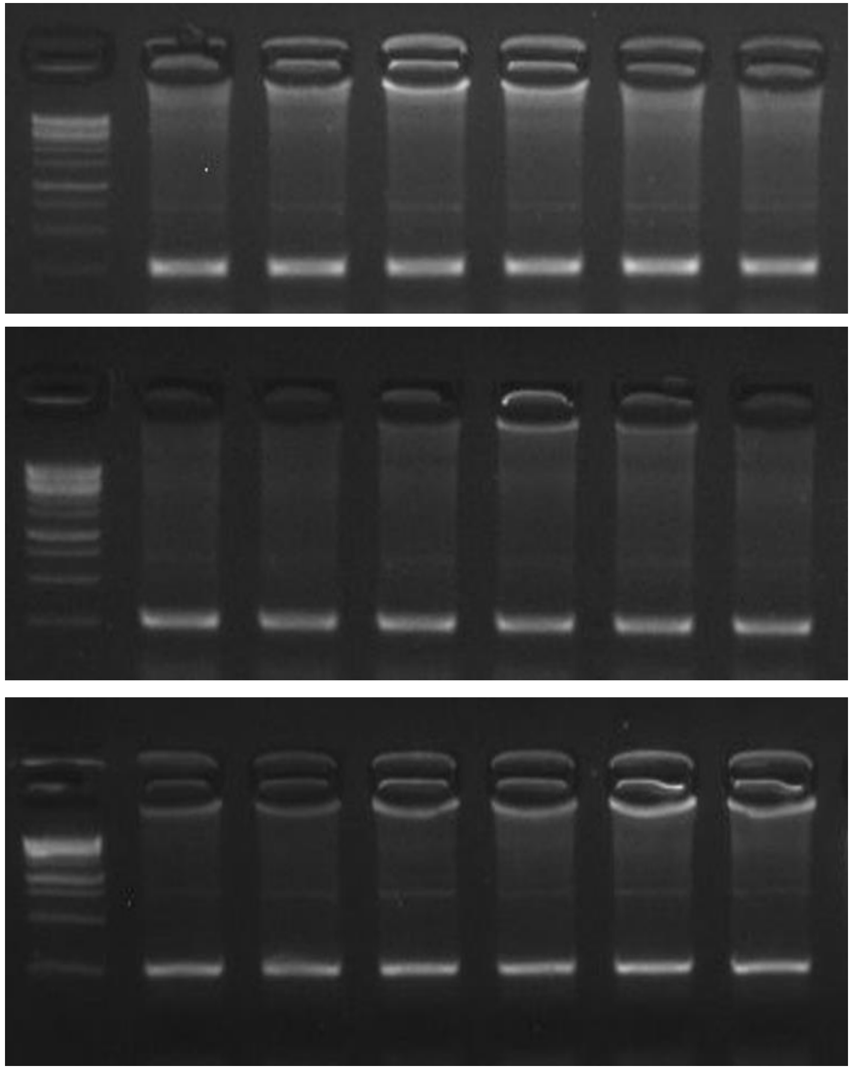

PMA (10 ng/mL)	-	+	+	+	+	+
IL-4 (30 ng/mL)	-	-	+	+	+	+
Irisin (20 nM)	-	-	-	+	-	+
Genistein (25 uM)	-	-	-	-	+	+

Full-length gels of Figure 3. C.

CD163 (OSM)

CD163
(28cycle)

Set 1

Set 2

Set 3
(**Representative image**)

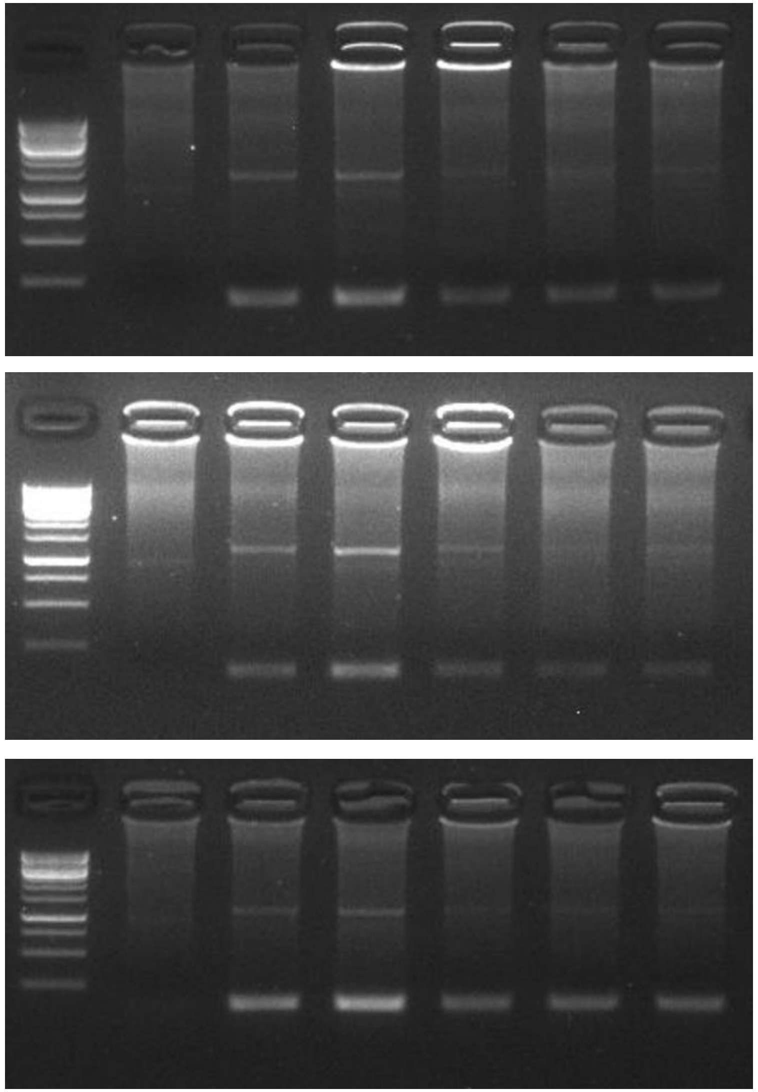

PMA (10 ng/mL)	-	+	+	+	+	+
IL-4 (30 ng/mL)	-	-	+	+	+	+
OSM (20 ng/mL)	-	-	-	+	-	+
Genistein (25 uM)	-	-	-	-	+	+

Full-length gels of Figure 3. C.

ARG1 (OSM)

ARG1
(28cycle)

Set 1
(**Representative image**)

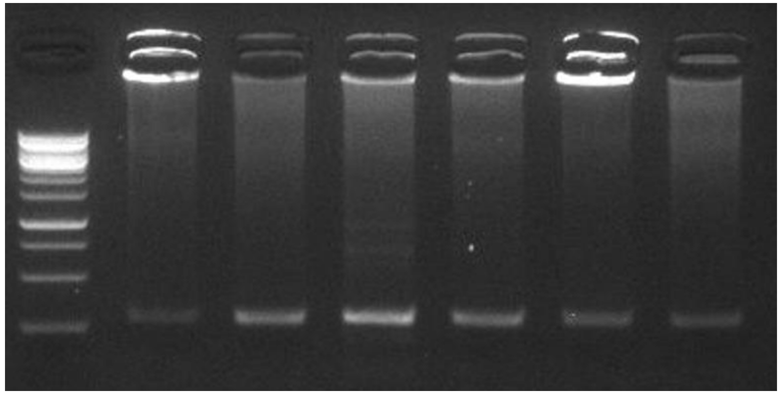

Set 2

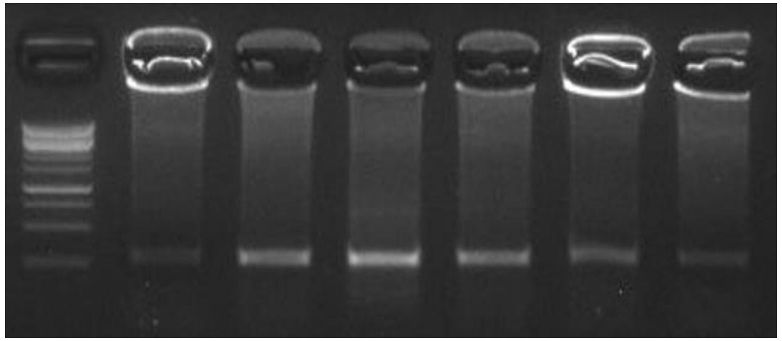

Set 3

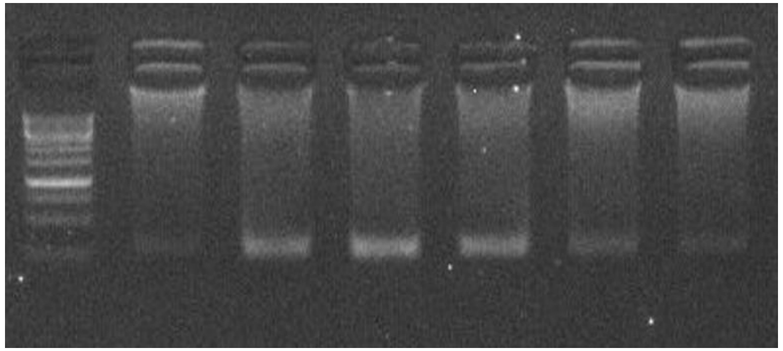

PMA (10 ng/mL)	-	+	+	+	+	+
IL-4 (30 ng/mL)	-	-	+	+	+	+
OSM (20 ng/mL)	-	-	-	+	-	+
Genistein (25 uM)	-	-	-	-	+	+

Full-length gels of Figure 3. C.

GAPDH (OSM)

GAPDH
(28cycle)

Set 1
(**Representative image**)

Set 2

Set 3

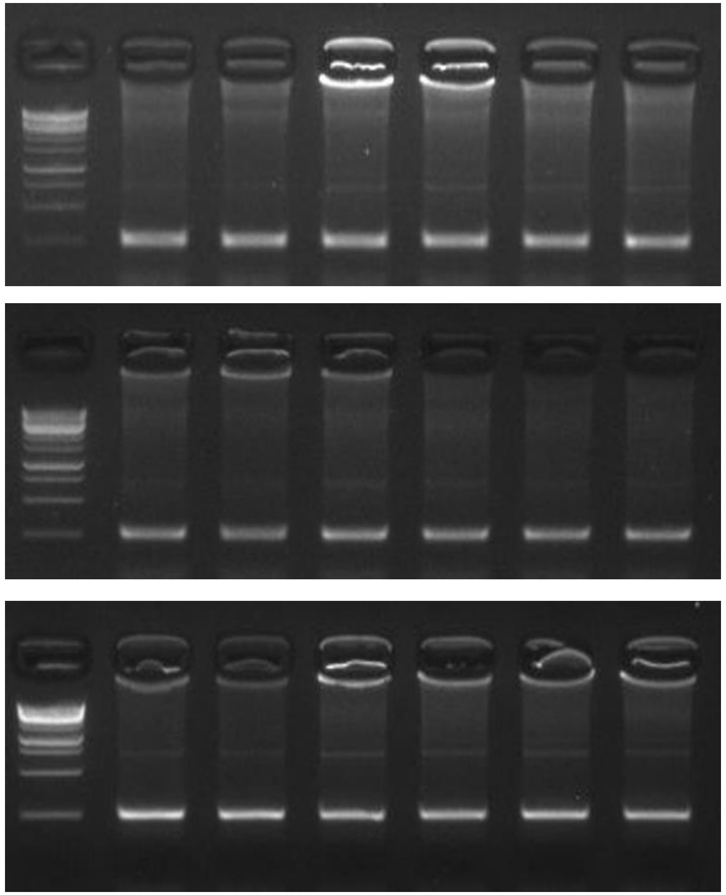

PMA (10 ng/mL)	-	+	+	+	+	+
IL-4 (30 ng/mL)	-	-	+	+	+	+
OSM (20 ng/mL)	-	-	-	+	-	+
Genistein (25 uM)	-	-	-	-	+	+

Full-length blots of Figure 4. A.

p-JAK1 (Irisin)

U937 cell

Protein: 30ug, 7% gel, 2'AB rabbit

p-JAK1 (1:200)
130kDa

Set 1
(**Representative image**)

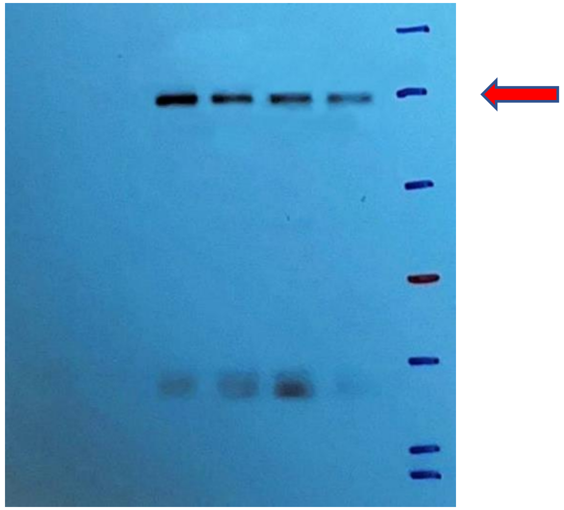

PMA (10 ng/mL)	-	+	+	+	+	+
IL-4 (30 ng/mL)	-	-	+	+	+	+
Irisin (20 nM)	-	-	-	+	-	+
Genistein (25 uM)	-	-	-	-	+	+

Set 2

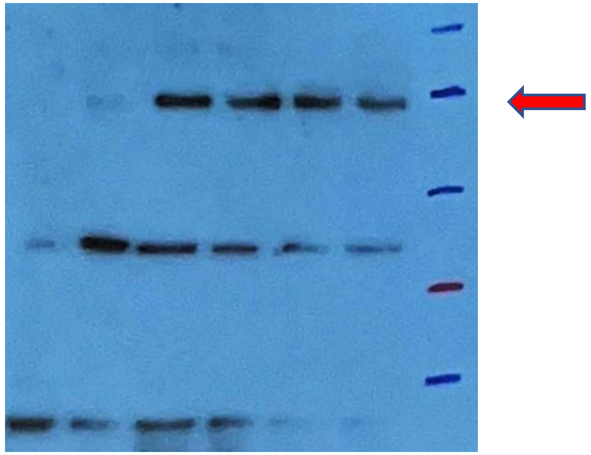

PMA (10 ng/mL)	-	+	+	+	+	+
IL-4 (30 ng/mL)	-	-	+	+	+	+
Irisin (20 nM)	-	-	-	+	-	+
Genistein (25 uM)	-	-	-	-	+	+

Set 3

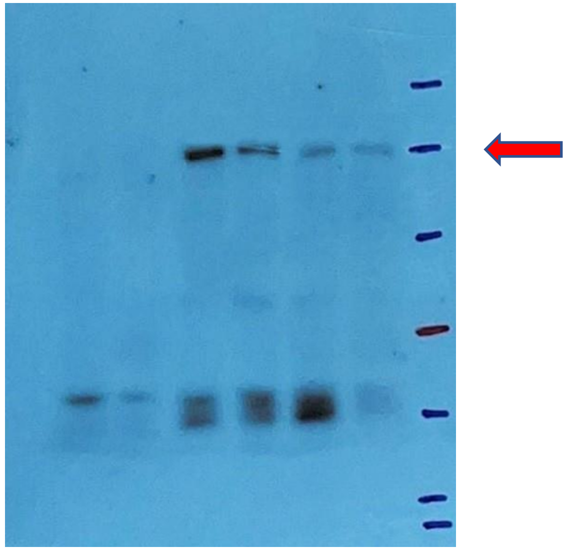

PMA (10 ng/mL)	-	+	+	+	+	+
IL-4 (30 ng/mL)	-	-	+	+	+	+
Irisin (20 nM)	-	-	-	+	-	+
Genistein (25 uM)	-	-	-	-	+	+

Full-length blots of <u>Figure 4. A.</u>

JAK1 (Irisin)

U937 cell

Protein: 30ug, 7% gel, 2'AB rabbit

JAK1 (1:200)
130kDa

Set 1
(**Representative image**)

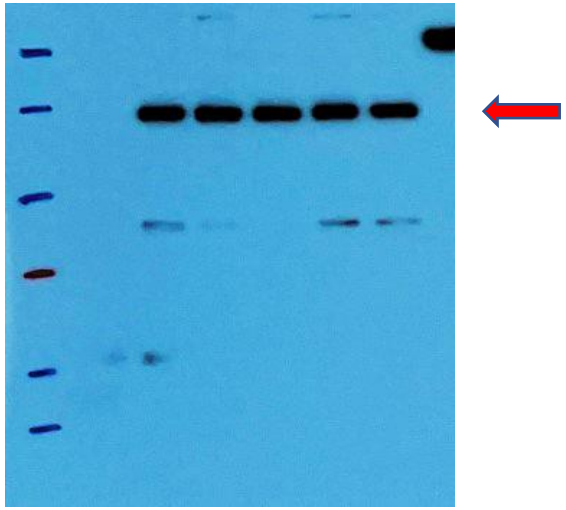

PMA (10 ng/mL)	-	+	+	+	+	+
IL-4 (30 ng/mL)	-	-	+	+	+	+
Irisin (20 nM)	-	-	-	+	-	+
Genistein (25 uM)	-	-	-	-	+	+

Set 2

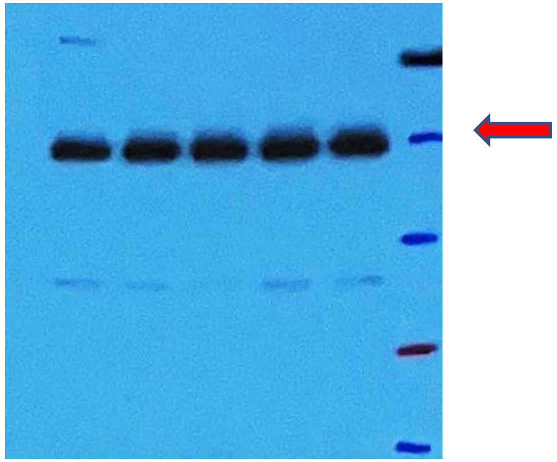

PMA (10 ng/mL)	-	+	+	+	+	+
IL-4 (30 ng/mL)	-	-	+	+	+	+
Irisin (20 nM)	-	-	-	+	-	+
Genistein (25 uM)	-	-	-	-	+	+

Set 3

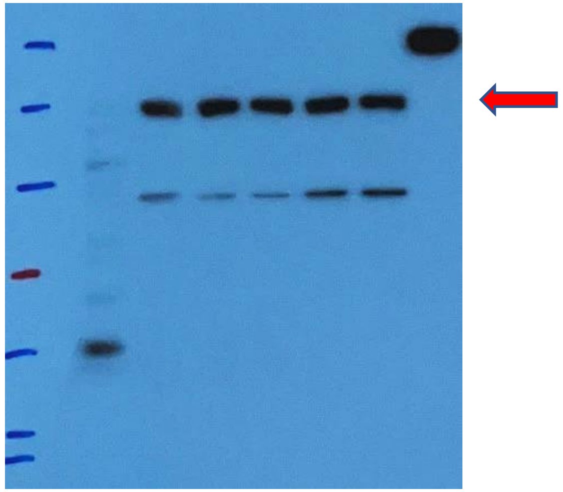

PMA (10 ng/mL)	-	+	+	+	+	+
IL-4 (30 ng/mL)	-	-	+	+	+	+
Irisin (20 nM)	-	-	-	+	-	+
Genistein (25 uM)	-	-	-	-	+	+

Full-length blots of Figure 4. A.

p-STAT6 (Irisin) U937 cell

Protein: 30ug, 7% gel, 2’AB rabbit

p-STAT6 (1:500)
110kDa

Set 1

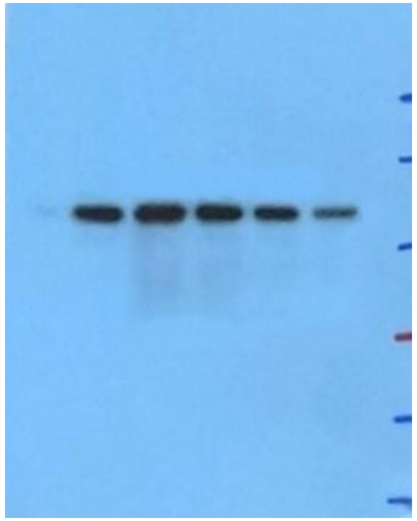

PMA (10 ng/mL)	-	+	+	+	+	+
IL-4 (30 ng/mL)	-	-	+	+	+	+
Irisin (20 nM)	-	-	-	+	-	+
Genistein (25 uM)	-	-	-	-	+	+

Set 2
(**Representative image**)

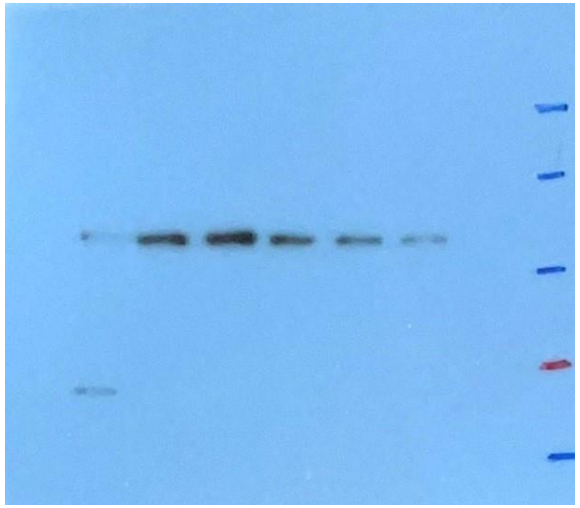

PMA (10 ng/mL)	-	+	+	+	+	+
IL-4 (30 ng/mL)	-	-	+	+	+	+
Irisin (20 nM)	-	-	-	+	-	+
Genistein (25 uM)	-	-	-	-	+	+

Set 3

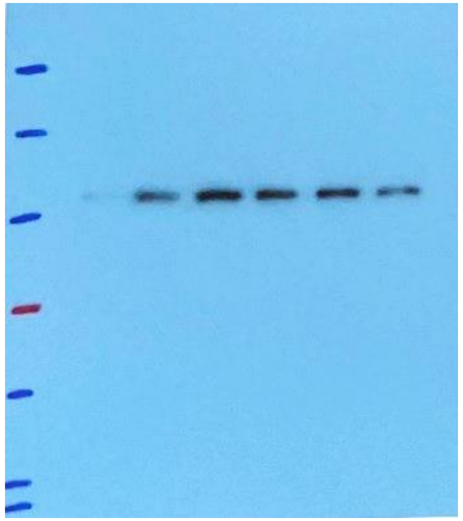

PMA (10 ng/mL)	-	+	+	+	+	+
IL-4 (30 ng/mL)	-	-	+	+	+	+
Irisin (20 nM)	-	-	-	+	-	+
Genistein (25 uM)	-	-	-	-	+	+

Full-length blots of Figure 4. A.

STAT6 (Irisin)

U937 cell

Protein: 30ug, 7% gel, 2'AB rabbit

STAT6 (1:500)
110kDa

Set 1

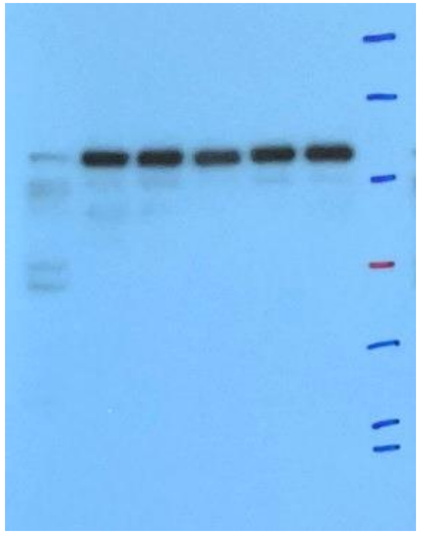

PMA (10 ng/mL)	-	+	+	+	+	+
IL-4 (30 ng/mL)	-	-	+	+	+	+
Irisin (20 nM)	-	-	-	+	-	+
Genistein (25 uM)	-	-	-	-	+	+

Set 2

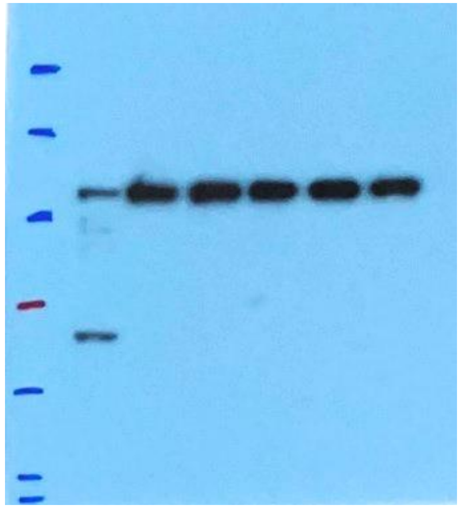

PMA (10 ng/mL)	-	+	+	+	+	+
IL-4 (30 ng/mL)	-	-	+	+	+	+
Irisin (20 nM)	-	-	-	+	-	+
Genistein (25 uM)	-	-	-	-	+	+

Set 3
(**Representative image**)

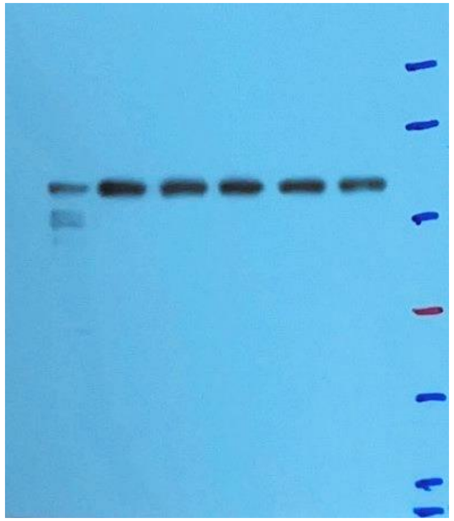

PMA (10 ng/mL)	-	+	+	+	+	+
IL-4 (30 ng/mL)	-	-	+	+	+	+
Irisin (20 nM)	-	-	-	+	-	+
Genistein (25 uM)	-	-	-	-	+	+

Full-length blots of Figure 4. A.

B-actin (Irisin)

U937 cell

Protein: 30ug, 7% gel, 2'AB rabbit

B-actin (1:400,000) 42kDa

Set 1

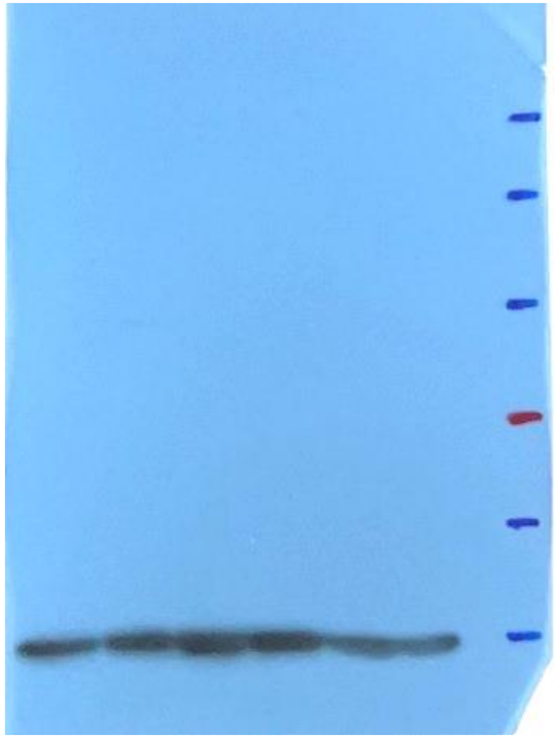

PMA (10 ng/mL)	-	+	+	+	+	+
IL-4 (30 ng/mL)	-	-	+	+	+	+
Irisin (20 nM)	-	-	-	+	-	+
Genistein (25 uM)	-	-	-	-	+	+

Set 2
(**Representative image**)

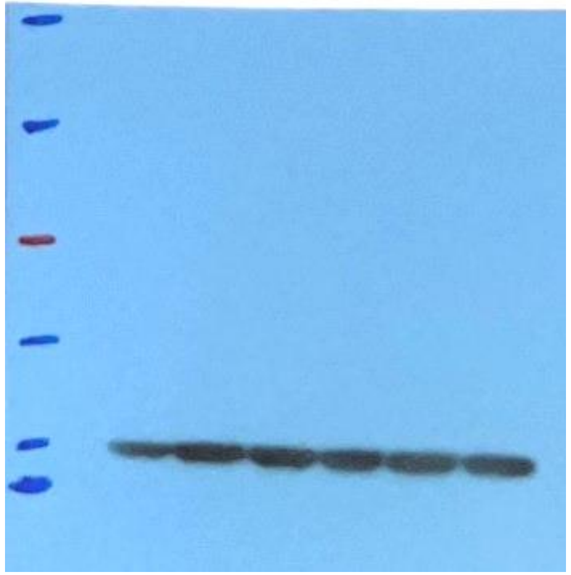

PMA (10 ng/mL)	-	+	+	+	+	+
IL-4 (30 ng/mL)	-	-	+	+	+	+
Irisin (20 nM)	-	-	-	+	-	+
Genistein (25 uM)	-	-	-	-	+	+

Set 3

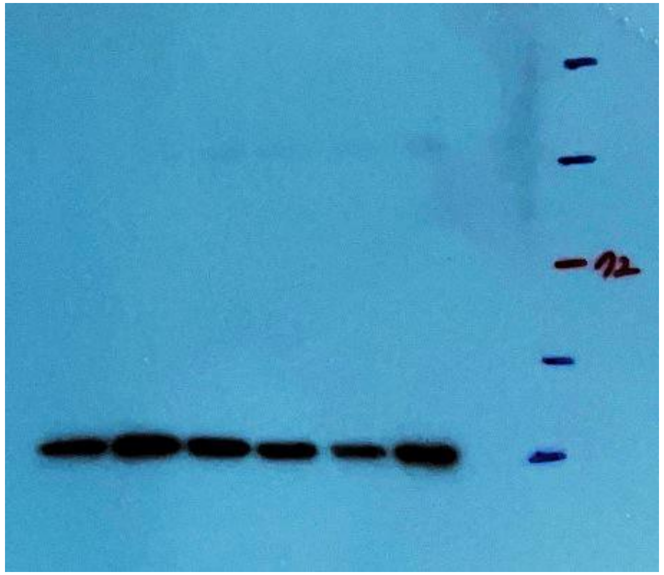

PMA (10 ng/mL)	-	+	+	+	+	+
IL-4 (30 ng/mL)	-	-	+	+	+	+
Irisin (20 nM)	-	-	-	+	-	+
Genistein (25 uM)	-	-	-	-	+	+

Full-length blots of Figure 4. B.

p-JAK1 (OSM)

U937 cell

Protein: 30ug, 7% gel, 2'AB rabbit

p-JAK1 (1:200)
130kDa

Set 1
(**Representative image**)

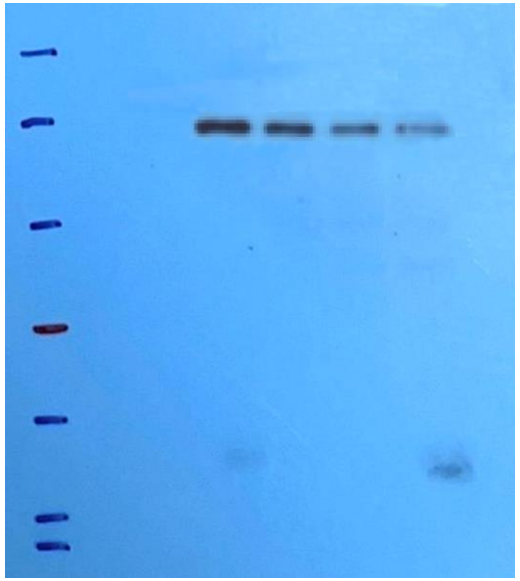

PMA (10 ng/mL)	-	+	+	+	+	+
IL-4 (30 ng/mL)	-	-	+	+	+	+
OSM (20 ng/mL)	-	-	-	+	-	+
Genistein (25 uM)	-	-	-	-	+	+

Set 2

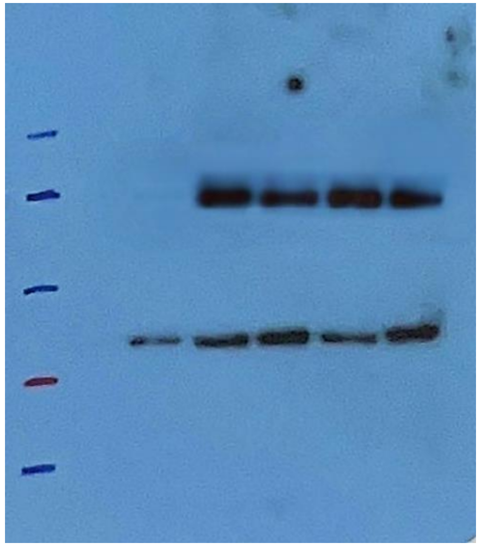

PMA (10 ng/mL)	-	+	+	+	+	+
IL-4 (30 ng/mL)	-	-	+	+	+	+
OSM (20 ng/mL)	-	-	-	+	-	+
Genistein (25 uM)	-	-	-	-	+	+

Set 3

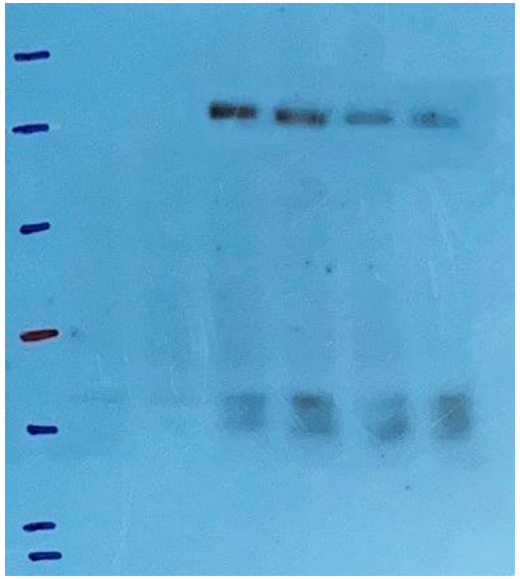

PMA (10 ng/mL)	-	+	+	+	+	+
IL-4 (30 ng/mL)	-	-	+	+	+	+
OSM (20 ng/mL)	-	-	-	+	-	+
Genistein (25 uM)	-	-	-	-	+	+

Full-length blots of <u>Figure 4. B.</u>

JAK1 (OSM)

U937 cell

Protein: 30ug, 7% gel, 2'AB rabbit

JAK1 (1:200)
130kDa

Set 1

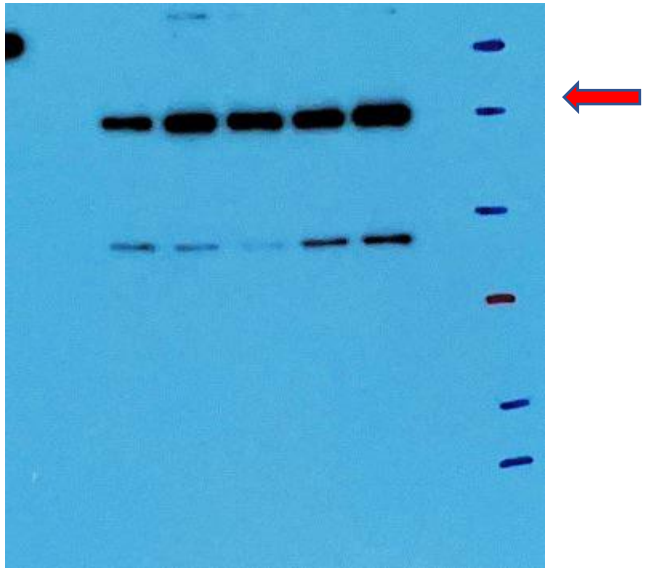

PMA (10 ng/mL)	-	+	+	+	+	+
IL-4 (30 ng/mL)	-	-	+	+	+	+
OSM (20 ng/mL)	-	-	-	+	-	+
Genistein (25 uM)	-	-	-	-	+	+

Set 2
(**Representative image**)

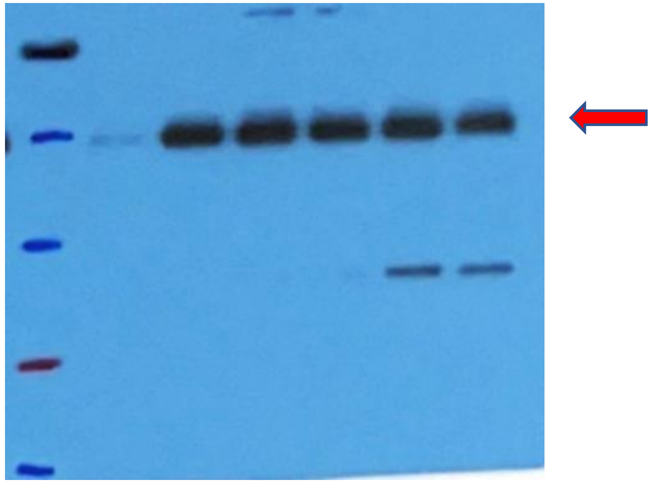

PMA (10 ng/mL)	-	+	+	+	+	+
IL-4 (30 ng/mL)	-	-	+	+	+	+
OSM (20 ng/mL)	-	-	-	+	-	+
Genistein (25 uM)	-	-	-	-	+	+

Set 3

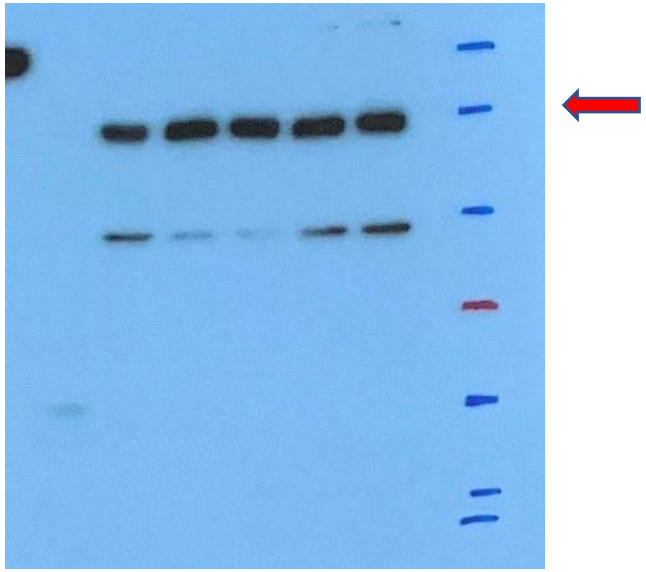

PMA (10 ng/mL)	-	+	+	+	+	+
IL-4 (30 ng/mL)	-	-	+	+	+	+
OSM (20 ng/mL)	-	-	-	+	-	+
Genistein (25 uM)	-	-	-	-	+	+

Full-length blots of Figure 4. B.

p-STAT6 (OSM) U937 cell

Protein: 30ug, 7% gel, 2'AB rabbit

p-STAT6 (1:500)
110kDa

Set 1
(**Representative image**)

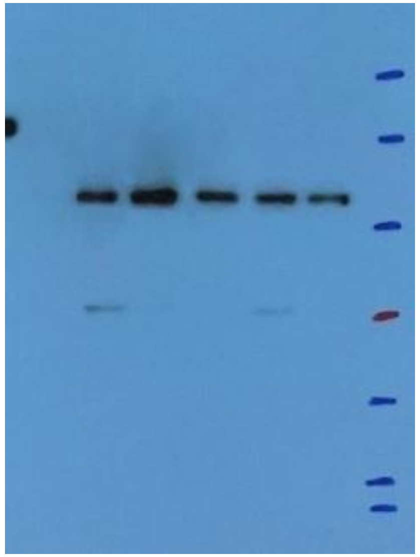

PMA (10 ng/mL)	-	+	+	+	+	+
IL-4 (30 ng/mL)	-	-	+	+	+	+
OSM (20 ng/mL)	-	-	-	+	-	+
Genistein (25 uM)	-	-	-	-	+	+

Set 2

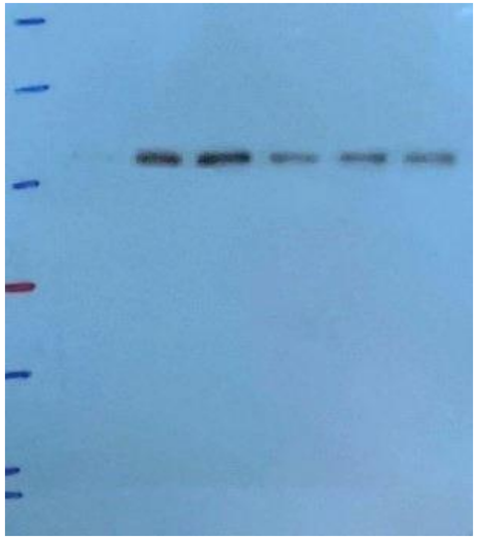

PMA (10 ng/mL)	-	+	+	+	+	+
IL-4 (30 ng/mL)	-	-	+	+	+	+
OSM (20 ng/mL)	-	-	-	+	-	+
Genistein (25 uM)	-	-	-	-	+	+

Set 3

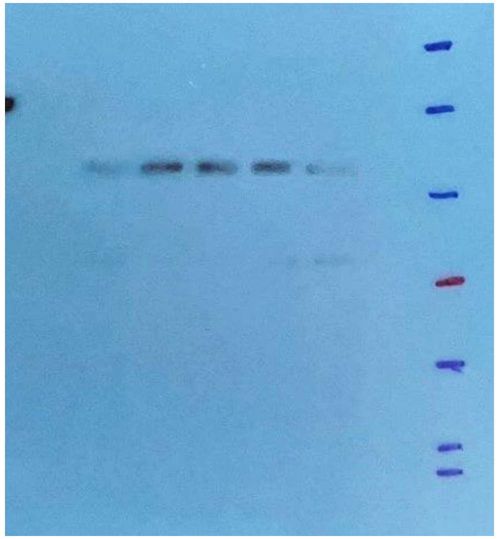

PMA (10 ng/mL)	-	+	+	+	+	+
IL-4 (30 ng/mL)	-	-	+	+	+	+
OSM (20 ng/mL)	-	-	-	+	-	+
Genistein (25 uM)	-	-	-	-	+	+

Full-length blots of Figure 4. B.

STAT6 (OSM)

U937 cell

Protein: 30ug, 7% gel, 2'AB rabbit

STAT6 (1:500)
110kDa

Set 1

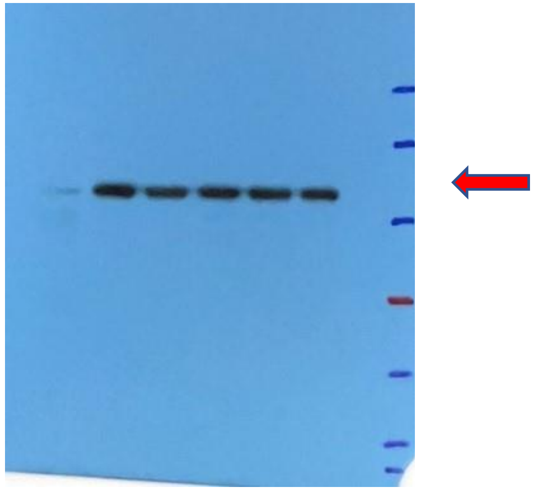

PMA (10 ng/mL)	-	+	+	+	+	+
IL-4 (30 ng/mL)	-	-	+	+	+	+
OSM (20 ng/mL)	-	-	-	+	-	+
Genistein (25 uM)	-	-	-	-	+	+

Set 2
(**Representative image**)

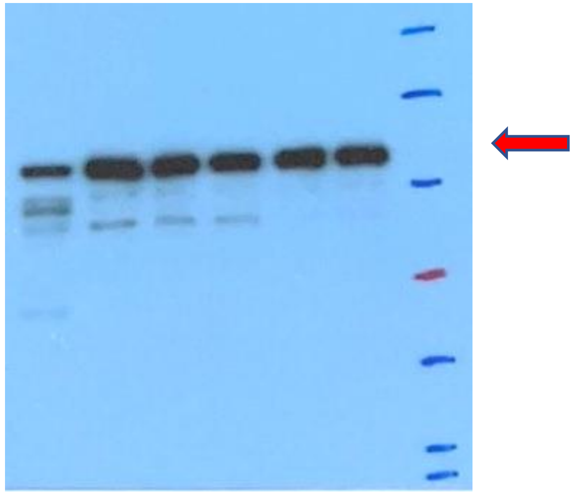

PMA (10 ng/mL)	-	+	+	+	+	+
IL-4 (30 ng/mL)	-	-	+	+	+	+
OSM (20 ng/mL)	-	-	-	+	-	+
Genistein (25 uM)	-	-	-	-	+	+

Set 3

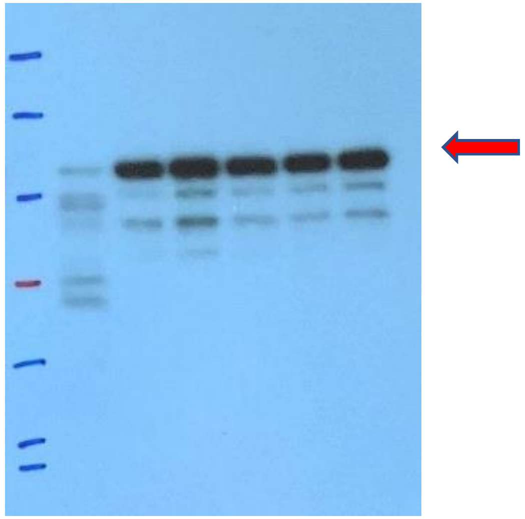

PMA (10 ng/mL)	-	+	+	+	+	+
IL-4 (30 ng/mL)	-	-	+	+	+	+
OSM (20 ng/mL)	-	-	-	+	-	+
Genistein (25 uM)	-	-	-	-	+	+

Full-length blots of <u>Figure 4. B.</u>

B-actin (OSM)

U937 cell

Protein: 30ug, 7% gel, 2'AB rabbit

B-actin
(1:400,000)
42kDa

Set 1

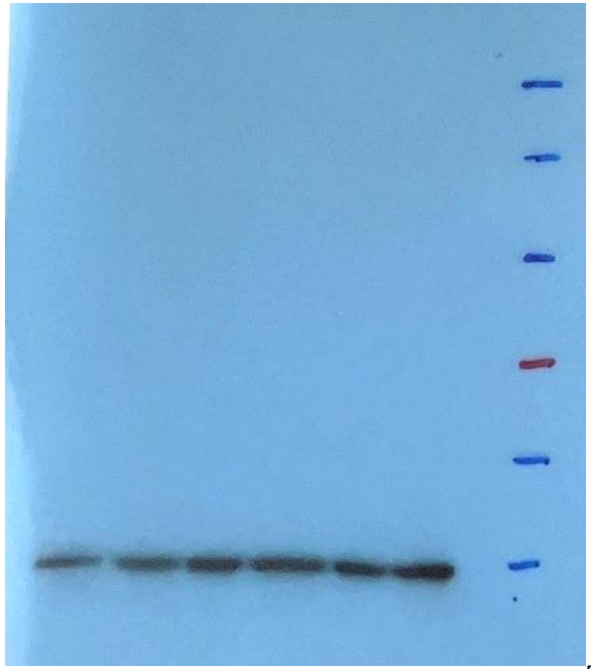

PMA (10 ng/mL)	-	+	+	+	+	+
IL-4 (30 ng/mL)	-	-	+	+	+	+
OSM (20 ng/mL)	-	-	-	+	-	+
Genistein (25 uM)	-	-	-	-	+	+

Set 2
(**Representative image**)

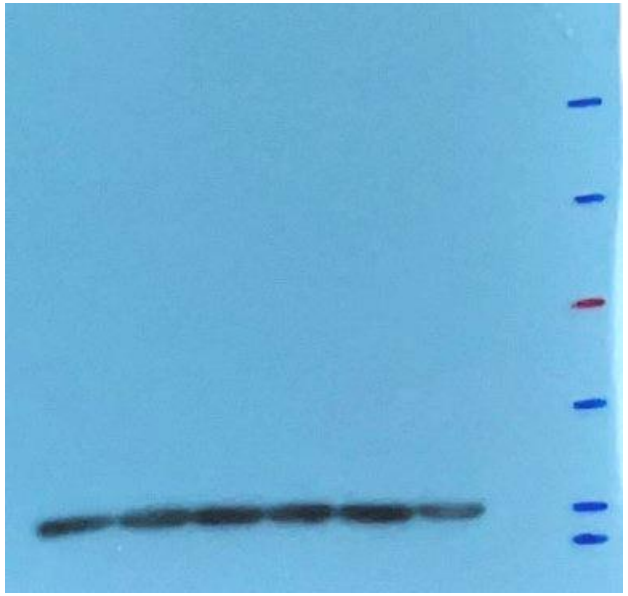

PMA (10 ng/mL)	-	+	+	+	+	+
IL-4 (30 ng/mL)	-	-	+	+	+	+
OSM (20 ng/mL)	-	-	-	+	-	+
Genistein (25 uM)	-	-	-	-	+	+

Set 3

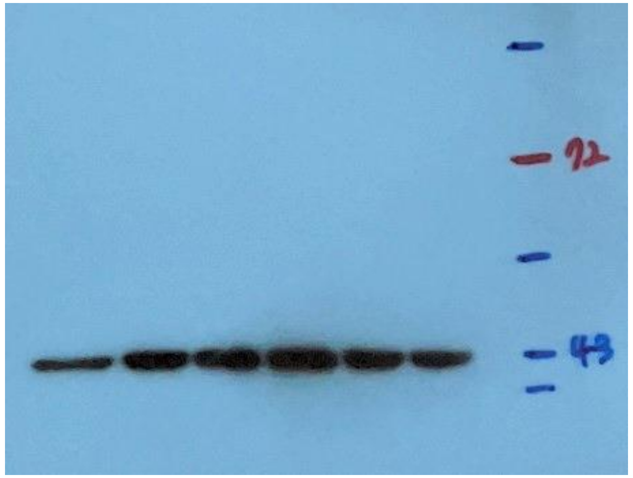

PMA (10 ng/mL)	-	+	+	+	+	+
IL-4 (30 ng/mL)	-	-	+	+	+	+
OSM (20 ng/mL)	-	-	-	+	-	+
Genistein (25 uM)	-	-	-	-	+	+

Full-length gels of Figure 5. A.

Pparg **(29cycle)**

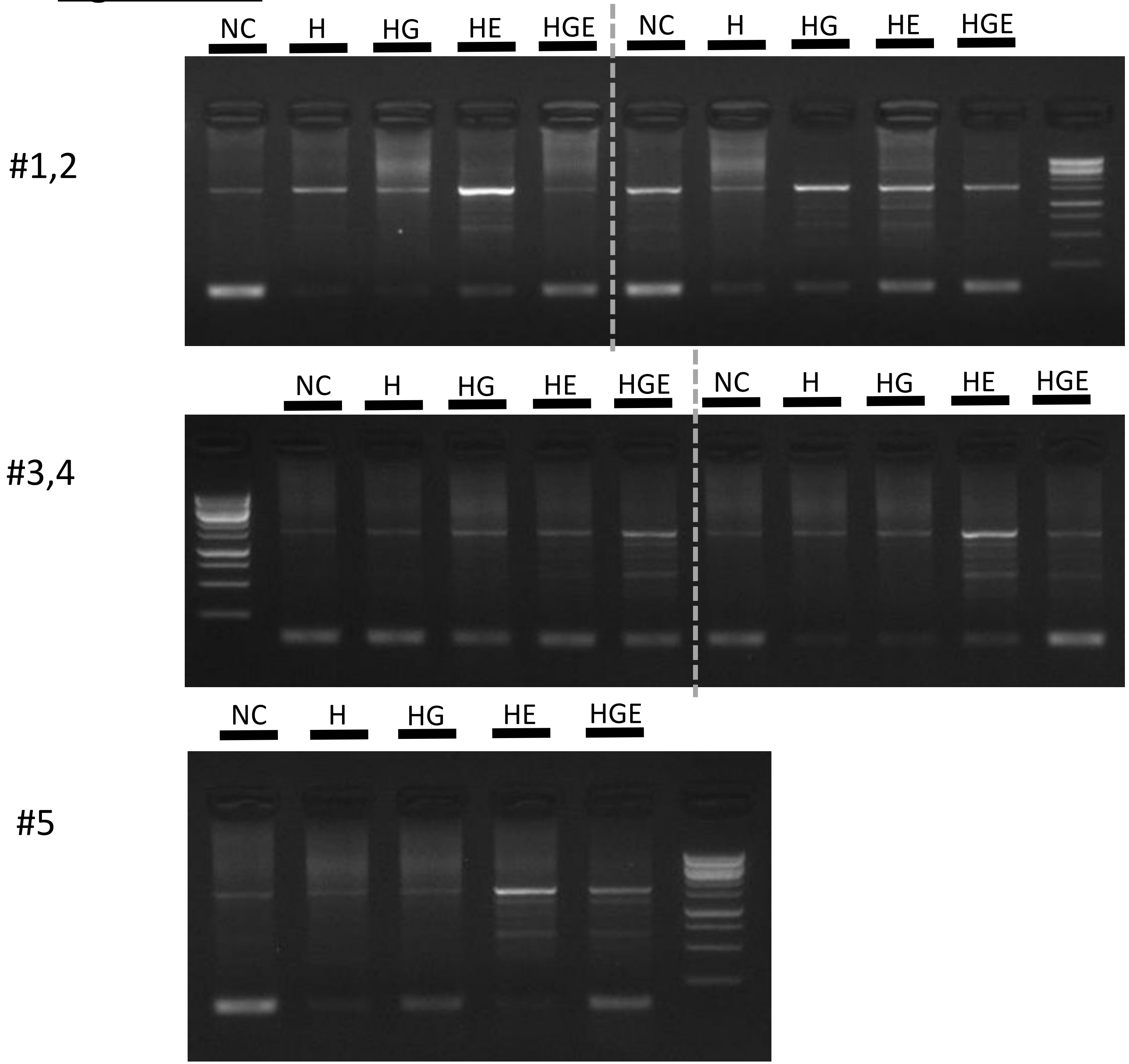

Full-length gels of Figure 5. A.

Pparg **(29cycle)**

#6

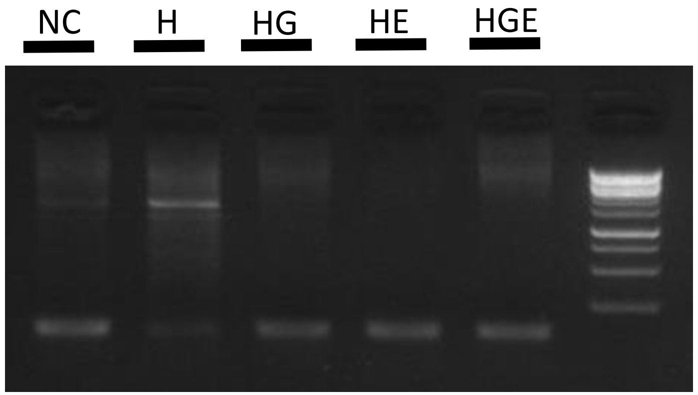

#8(**Representative image**),9

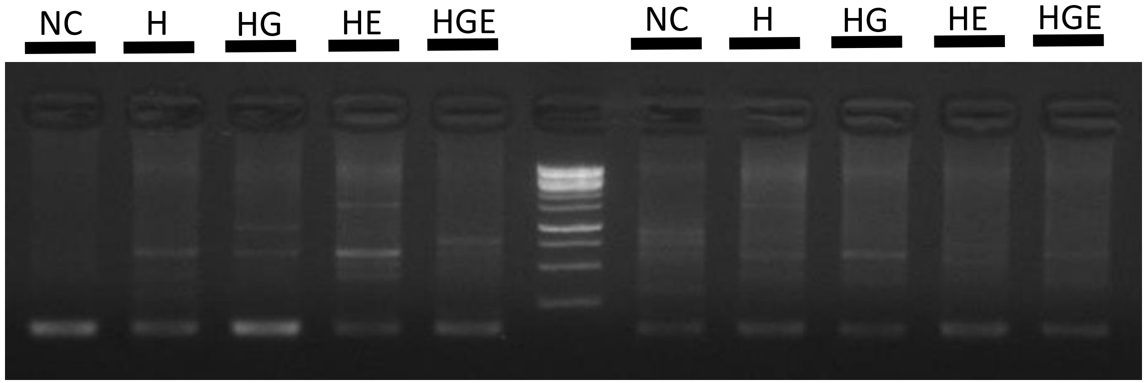

#7,10

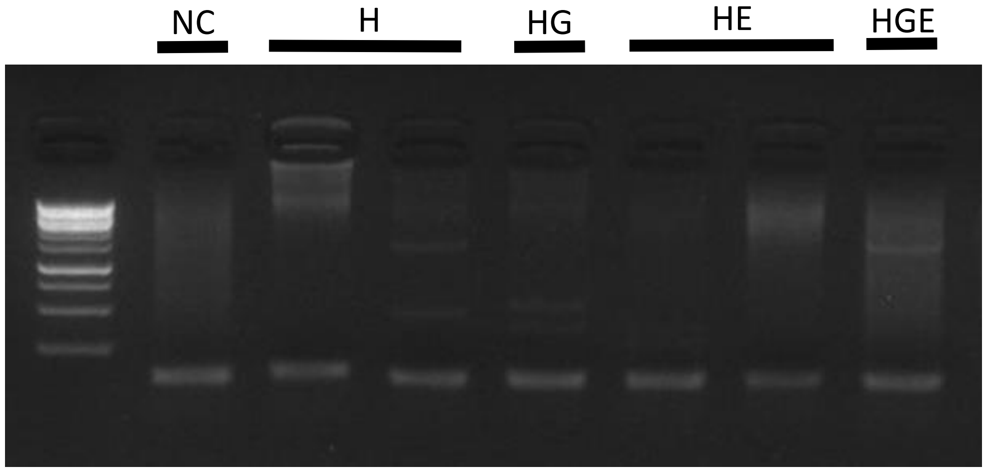

Full-length gels of Figure 5. A.

Cebpa **(31cycle)**

#1,2

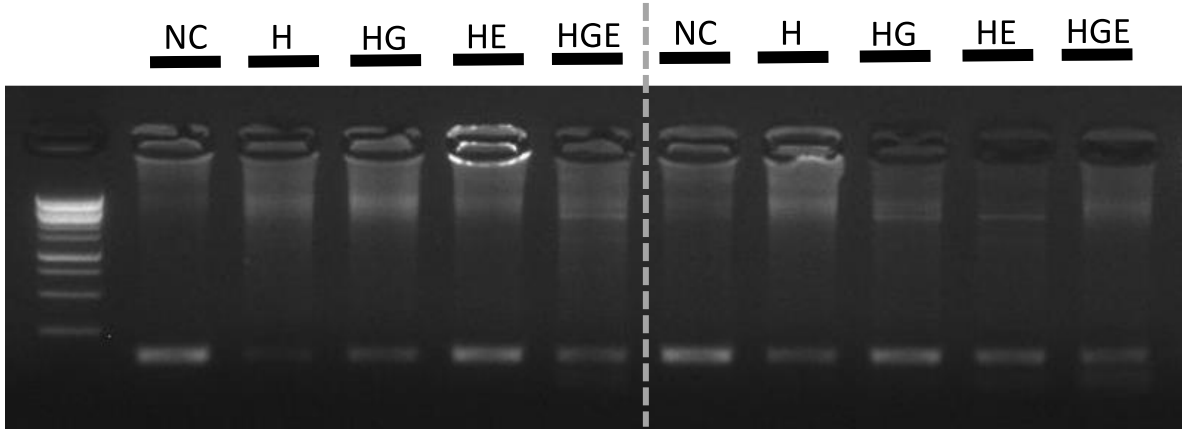

#3

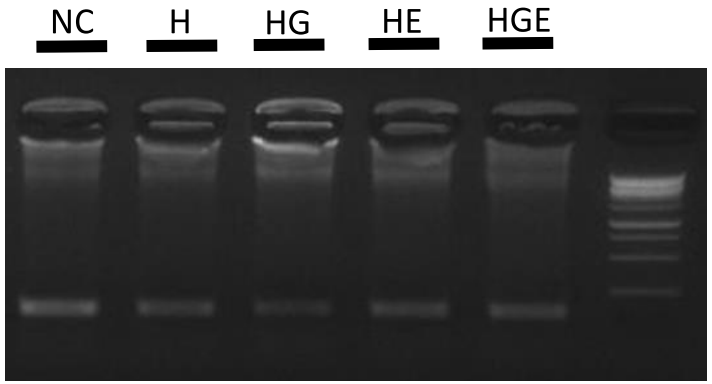

#4 (**Representative image)**,5

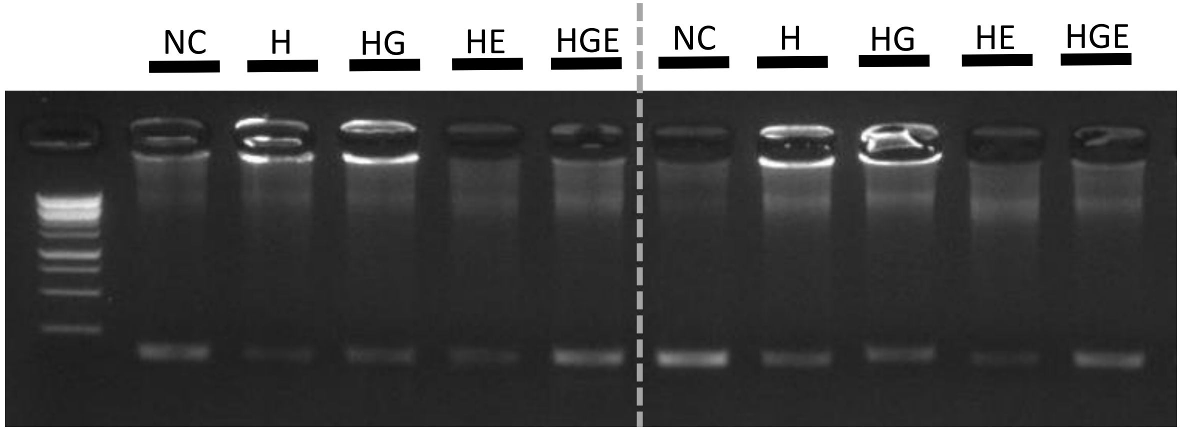

Full-length gels of Figure 5. A.

Cebpa (31cycle)

NC H HG HE HGE

#6

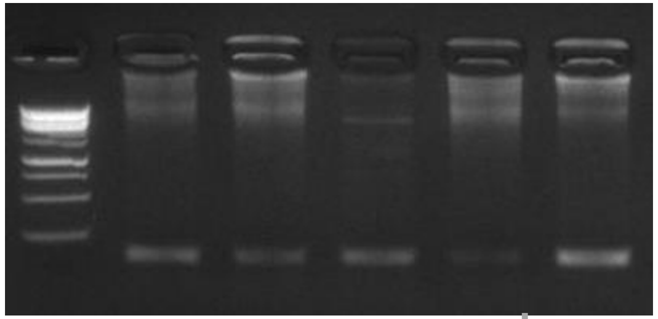

NC H HG HE HGE NC H HG HE HGE

#8,9

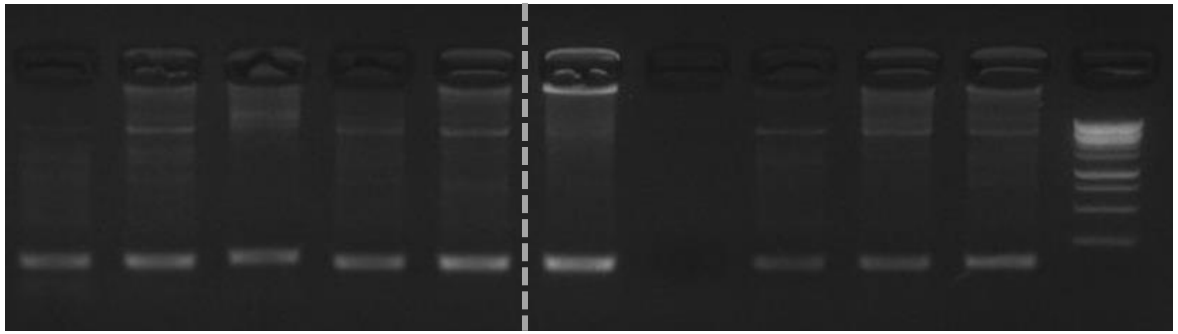

NC H HG HE HGE

#7,10

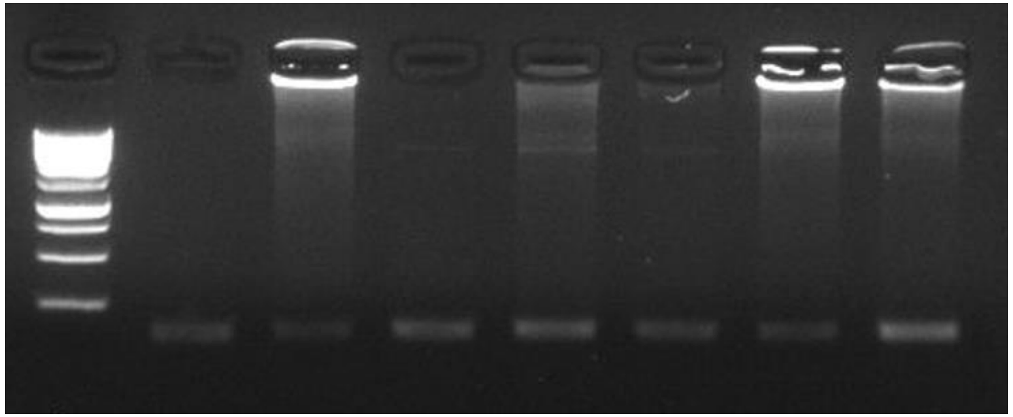

Full-length gels of Figure 5. A.

Srebf1 (30cycle)

#1, 2(Representative image)

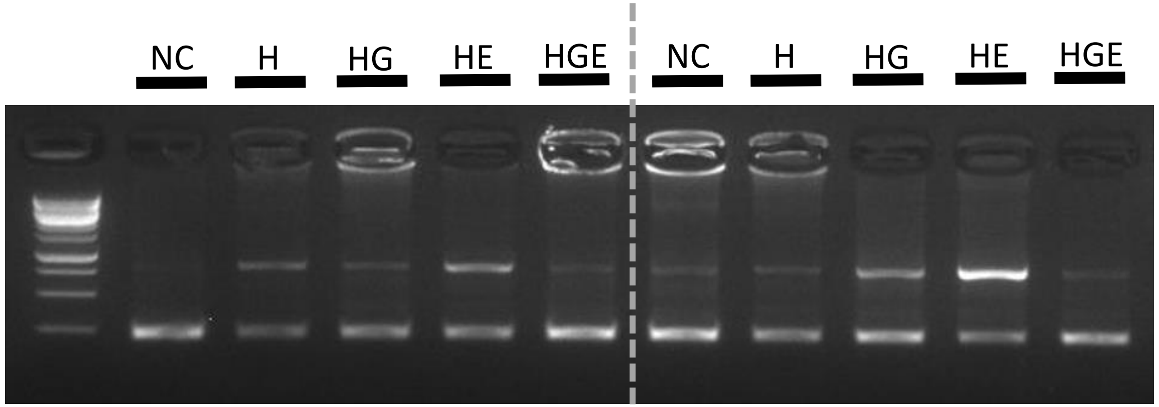

#3,4

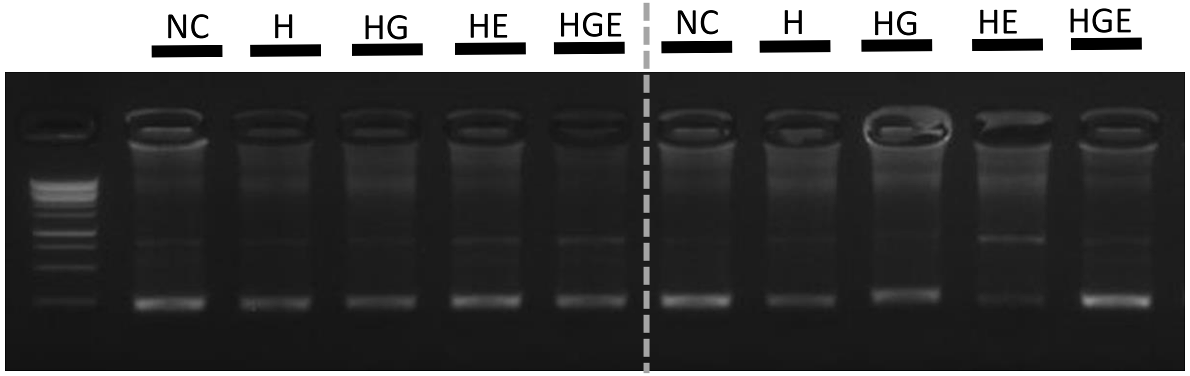

#5

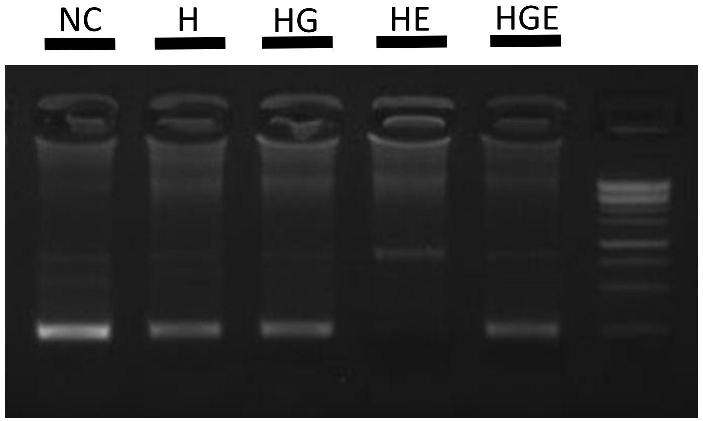

Full-length gels of <u>Figure 5. A.</u>

Srebf1 **(30cycle)**

#6,8

NC H HG HE HGE NC H HG HE HGE

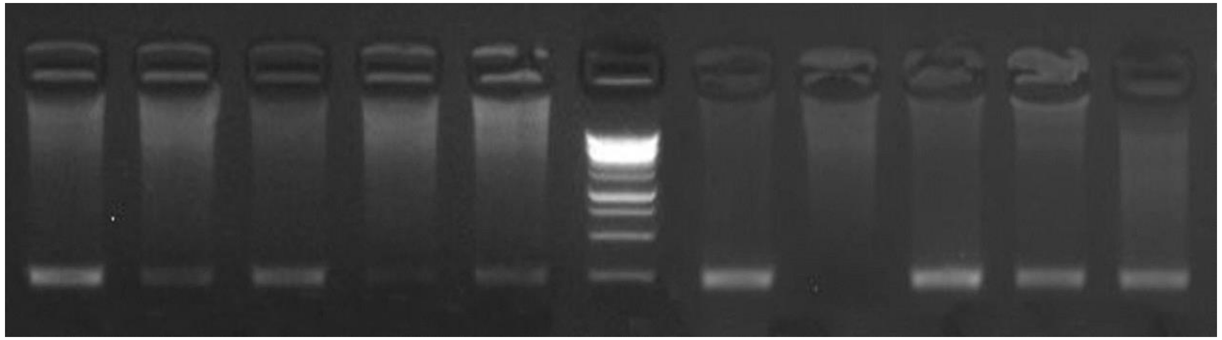

#9

NC H HG HE HGE

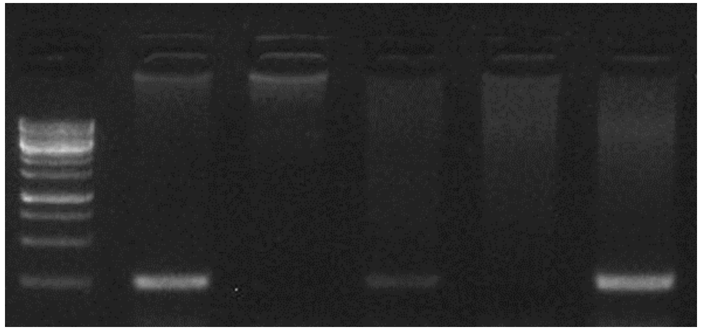

#7,10

NC H HG HE HGE

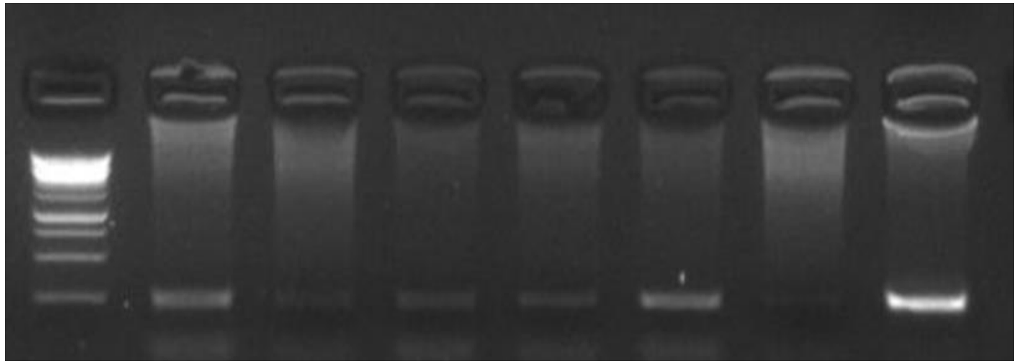

Full-length gels of Figure 5. A.

Acaca (31cycle)

#1,2

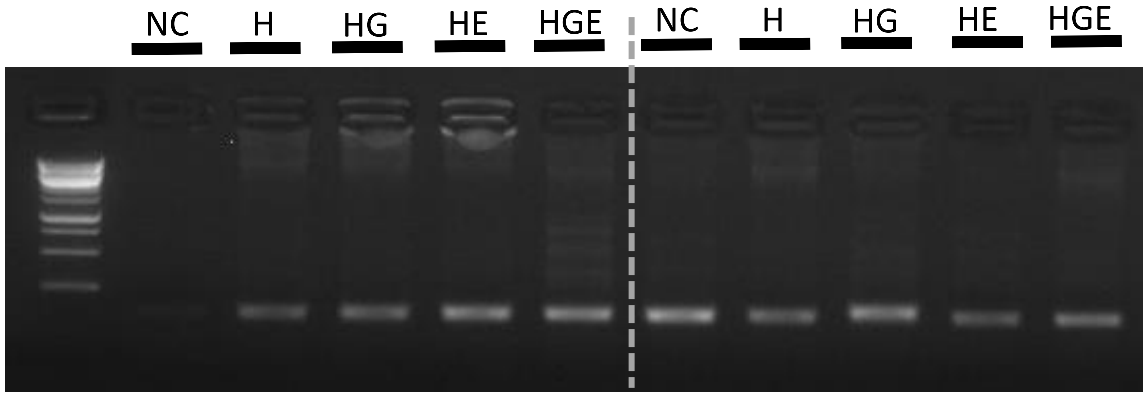

#3

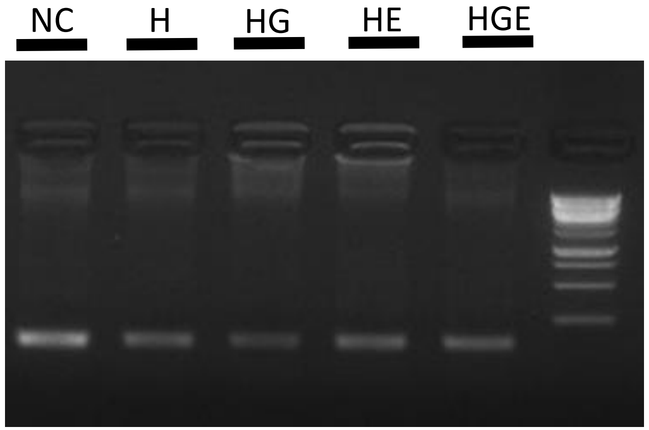

#4, 5(Representative image)

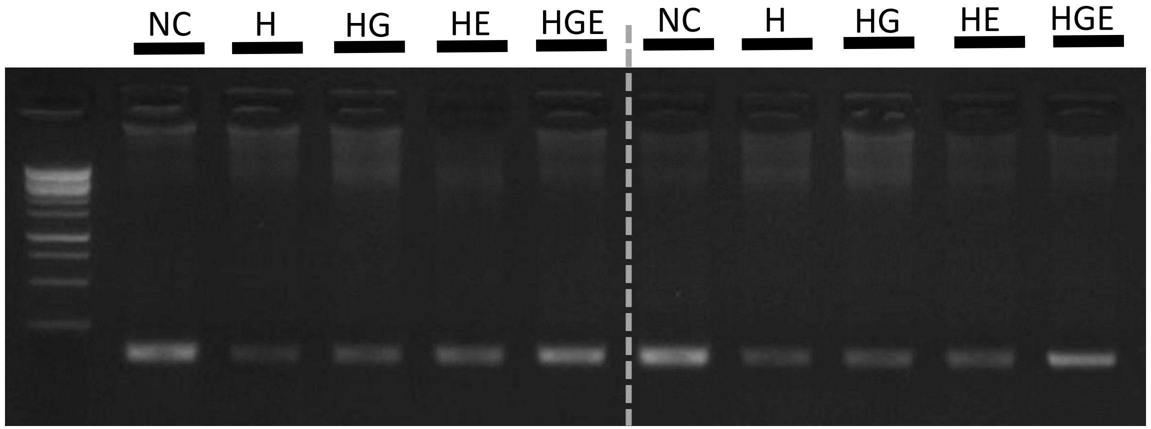

Full-length gels of Figure 5. A.

Acaca **(31cycle)**

#6

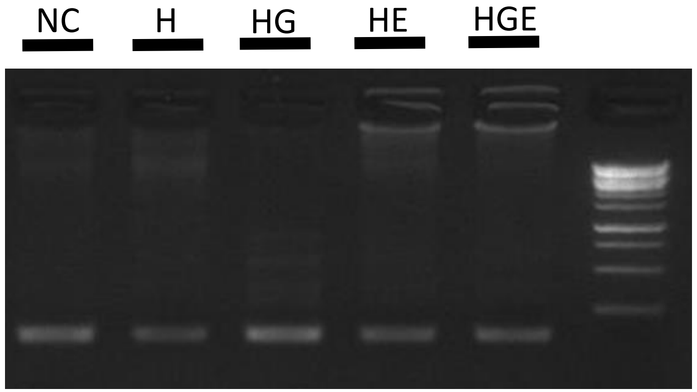

#8,9

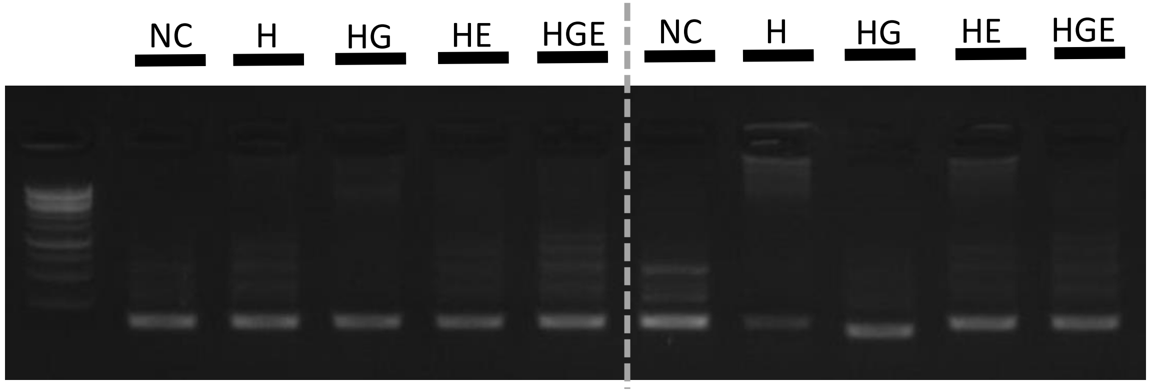

#7,10

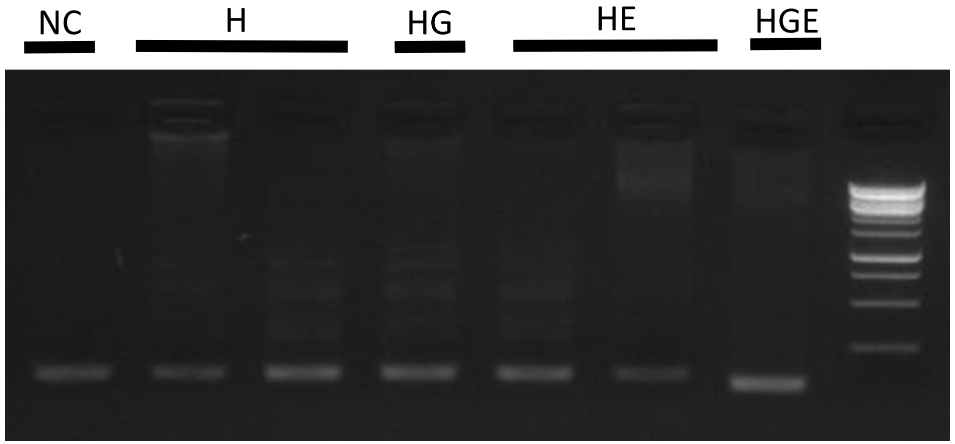

Full-length gels of Figure 5. A.

Fasn (30cycle)

#1

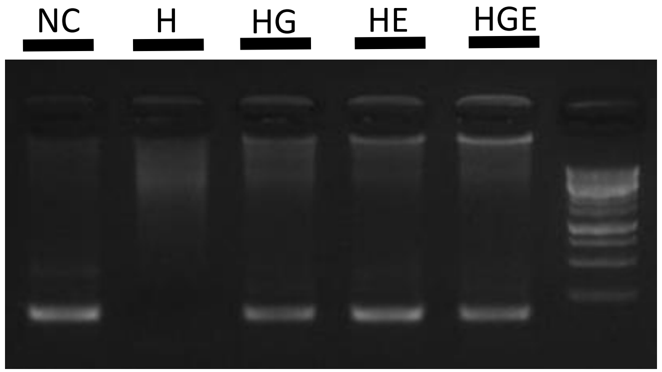

#2
(Representative image)

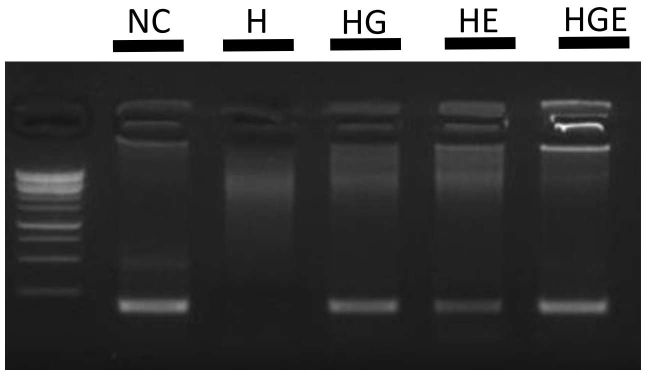

#3

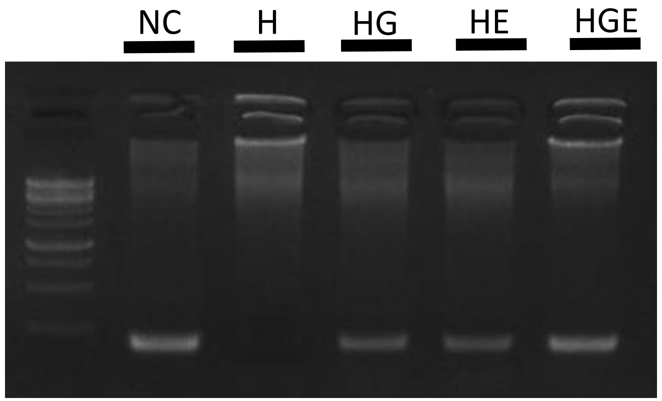

#4

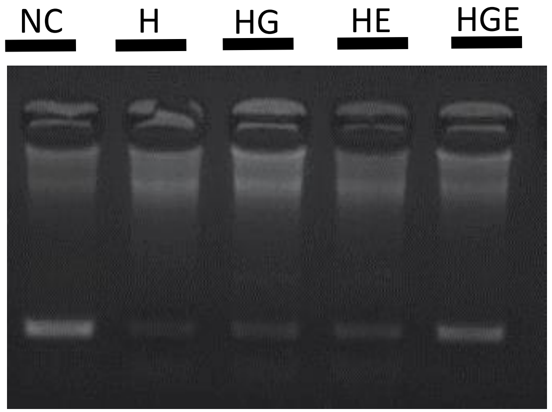

#5,6

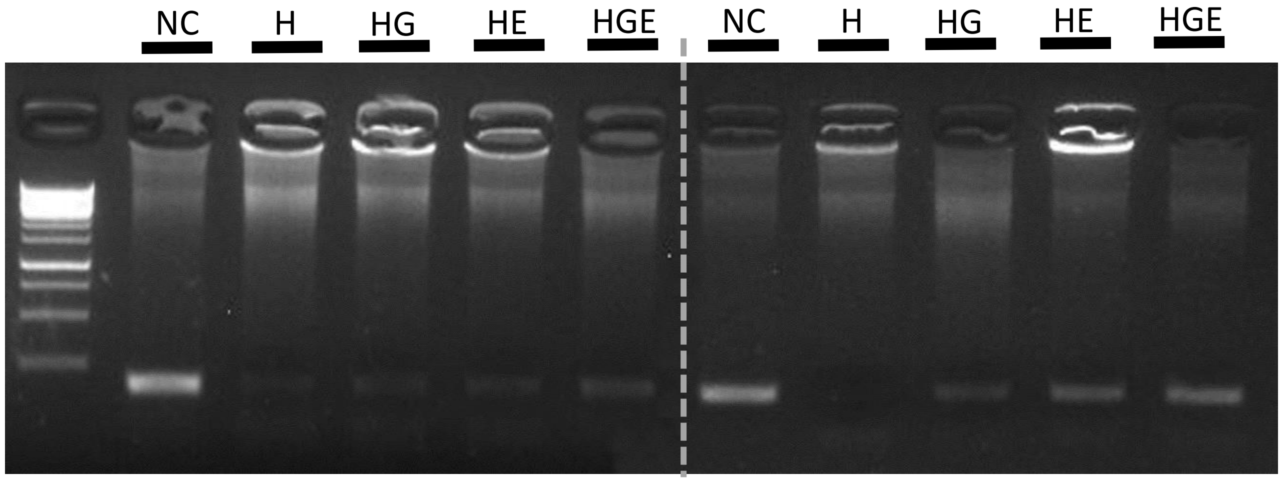

Full-length gels of <u>Figure 5. A.</u>

Fasn (30cycle)

#8

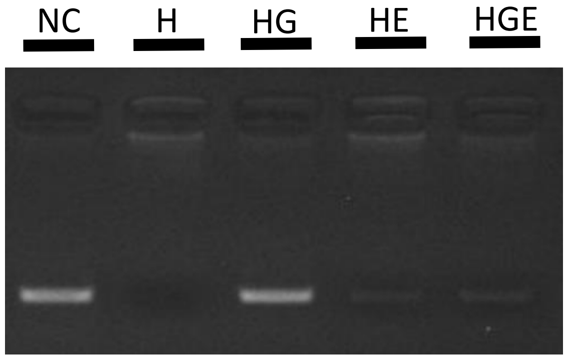

#9

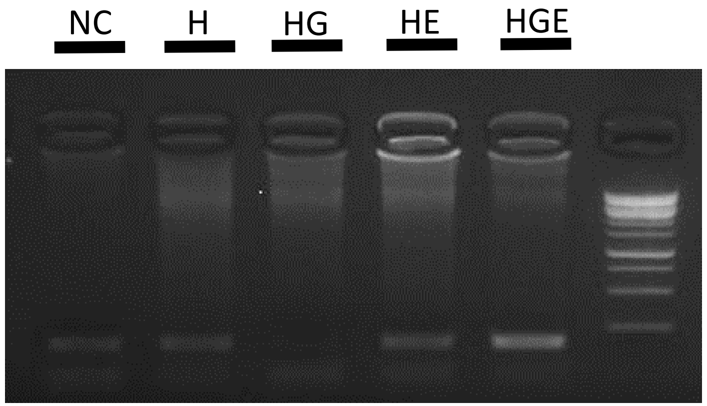

#7,10

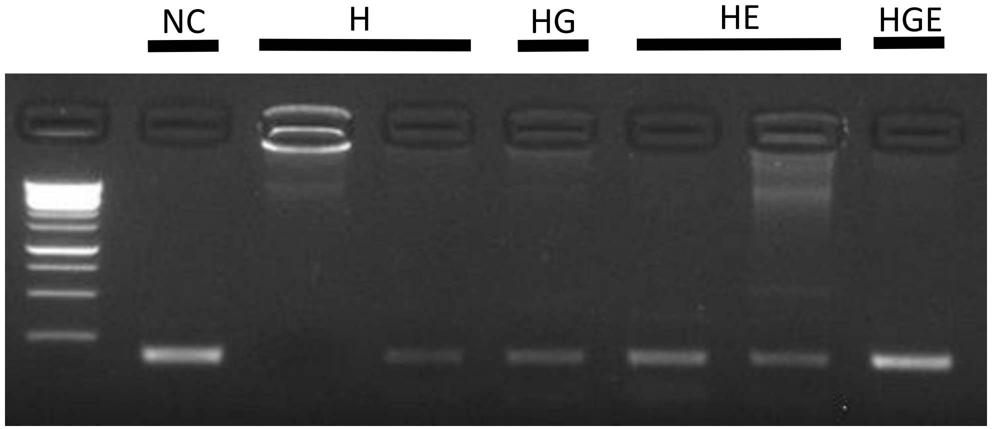

Full-length gels of Figure 5. A.

Gapdh (27cycle)

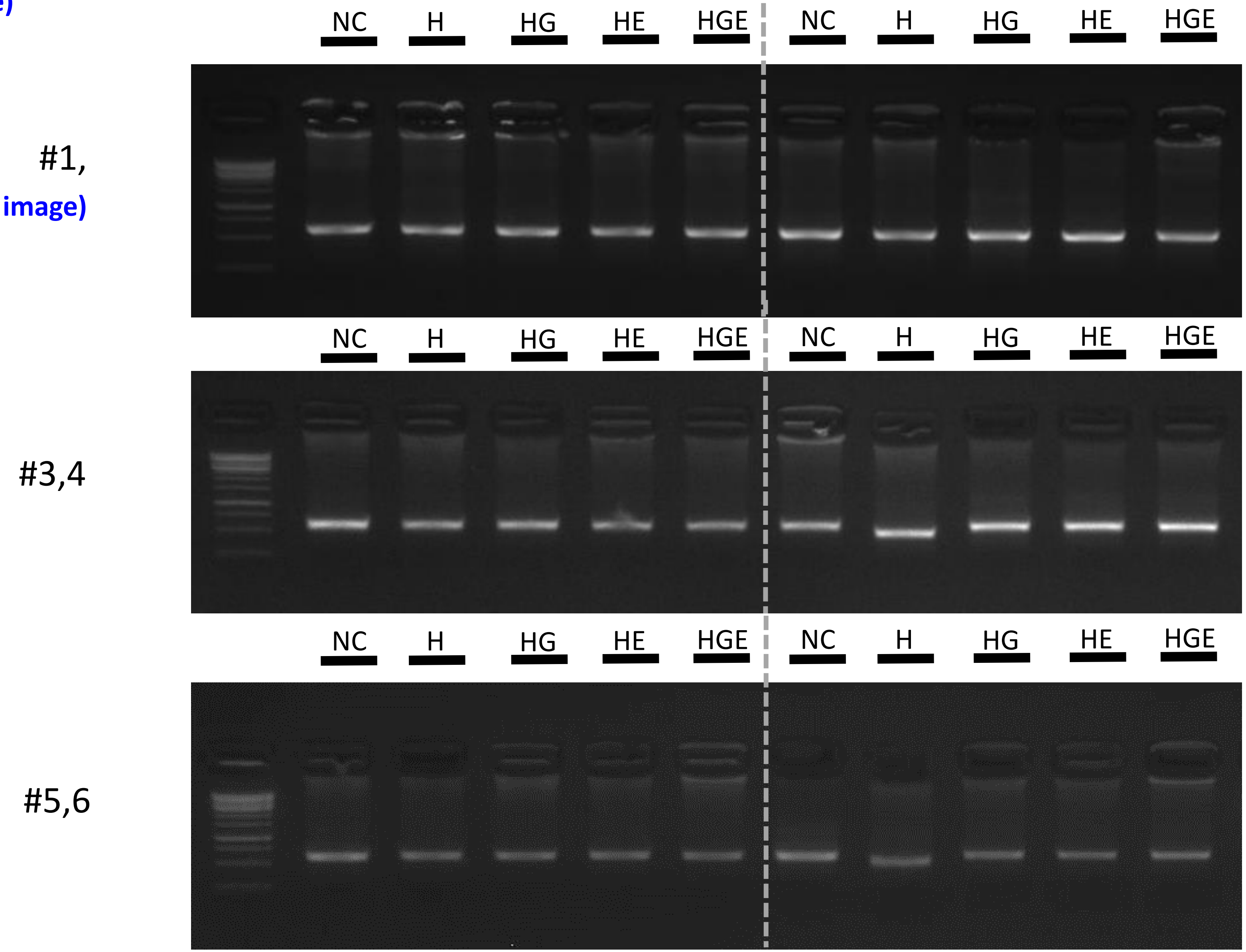

Full-length gels of <u>Figure 5. A.</u>

Gapdh **(27cycle)**

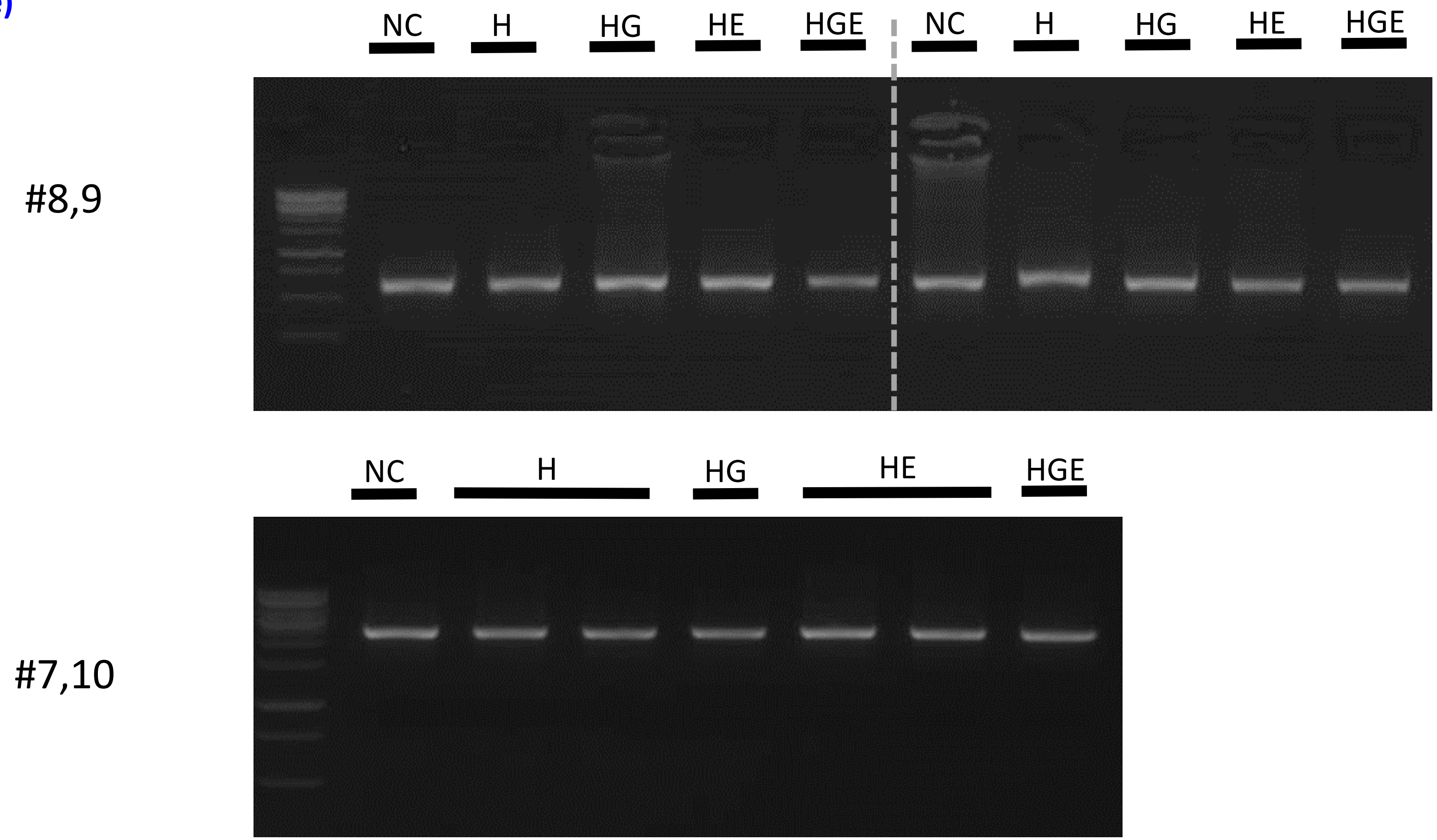

Full-length gels of <u>Figure 5. B.</u>

Atgl (29cycle)

#1,2

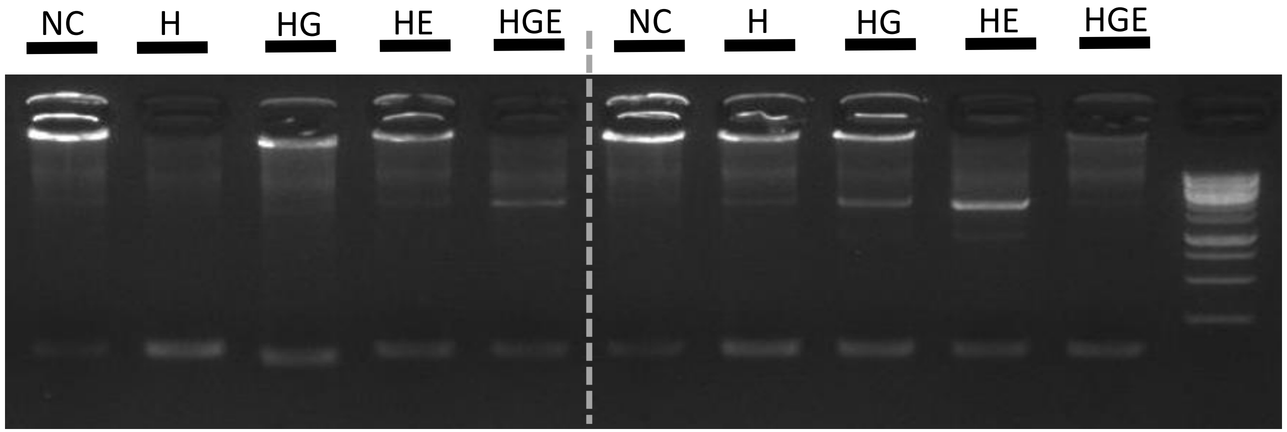

#3,4

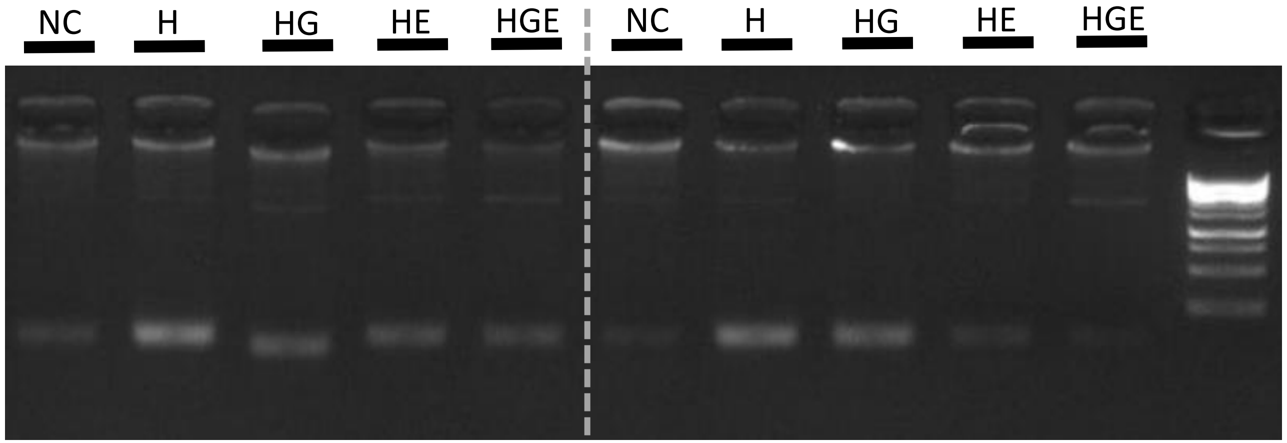

#5

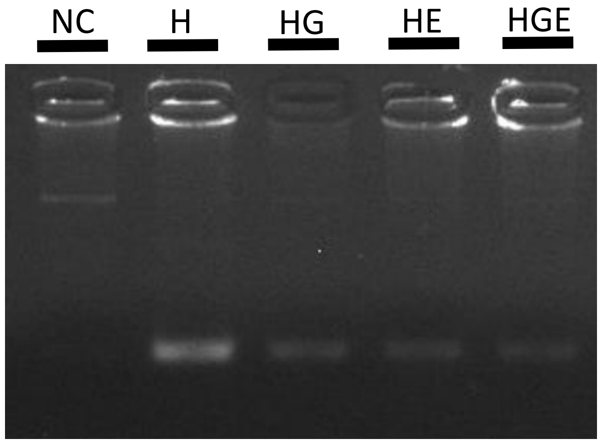

Full-length gels of Figure 5. B.

Atgl **(29cycle)**

#6

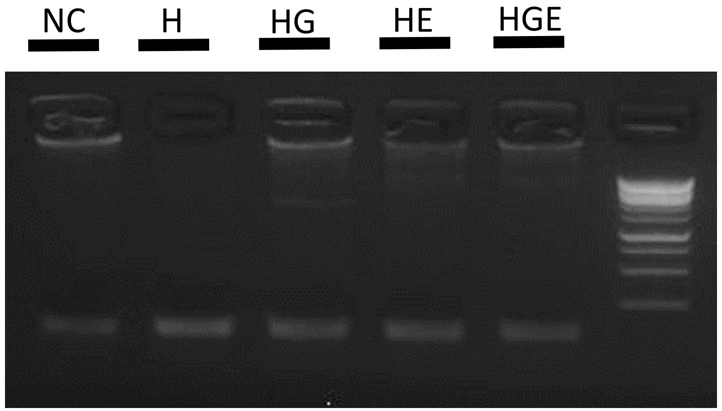

#8,
9(**Representative image)**

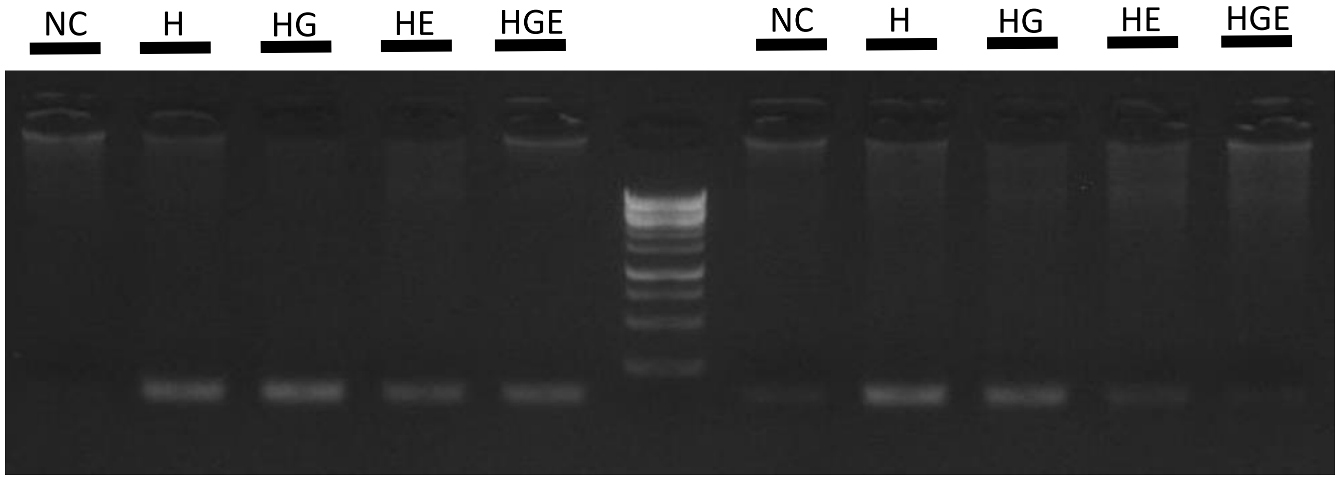

#7,10

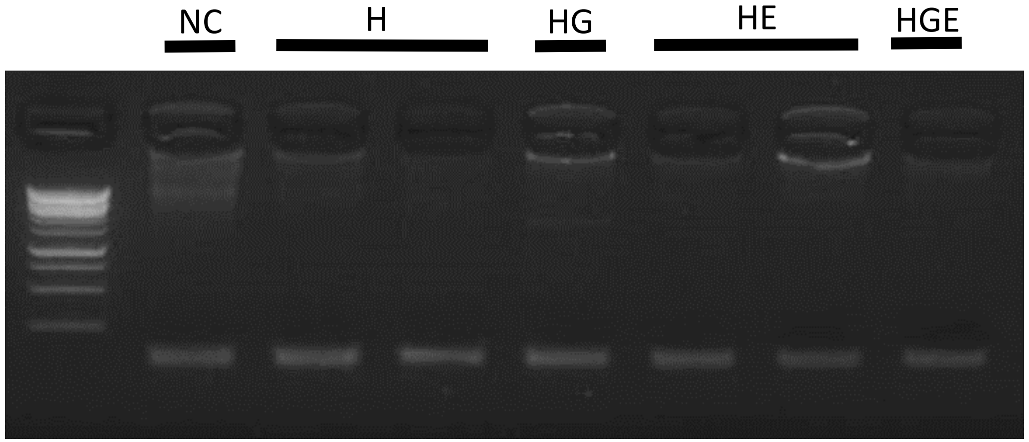

Full-length gels of <u>Figure 5. B.</u>

Hsl **(29cycle)**

#1

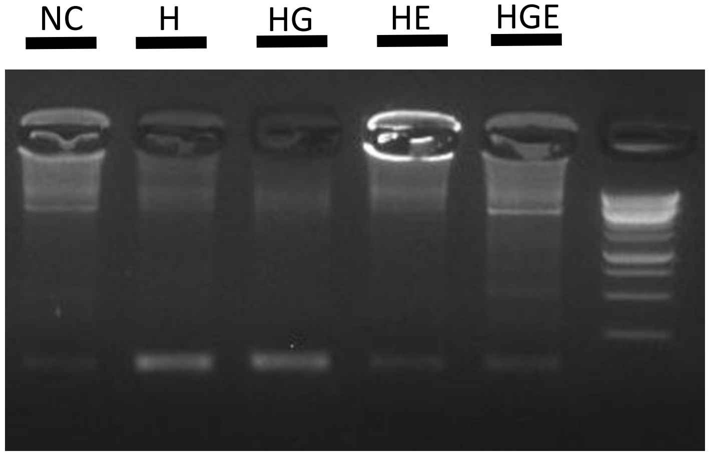

#2

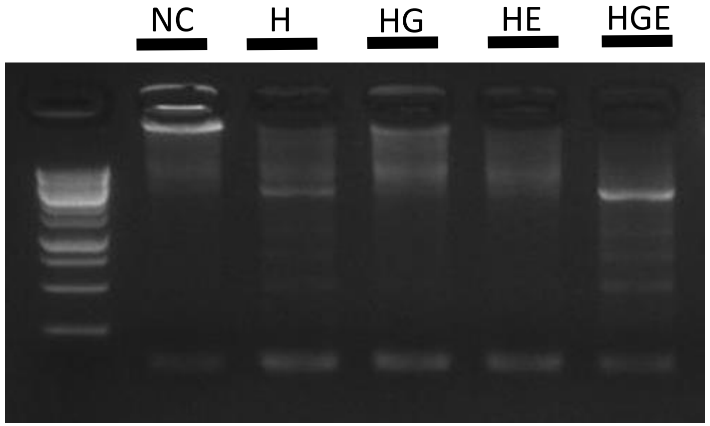

#3, 4 **(Representative image)**

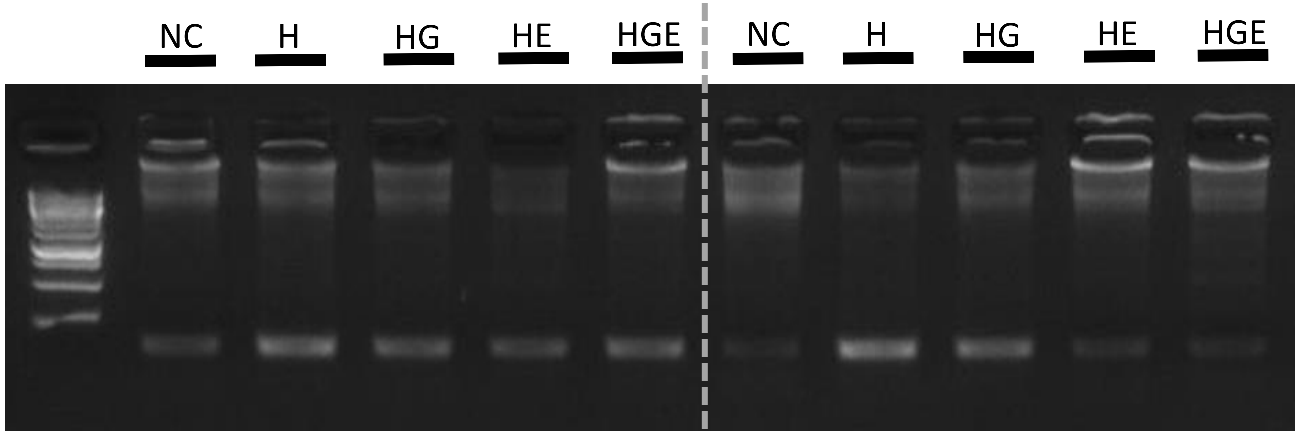

#5

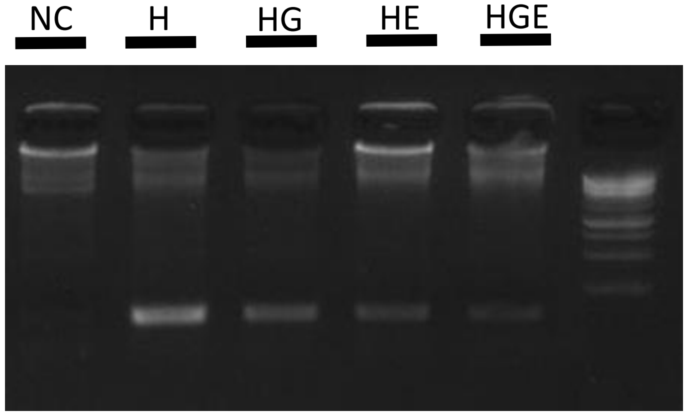

Full-length gels of Figure 5. B.

Hsl (29cycle)

#6

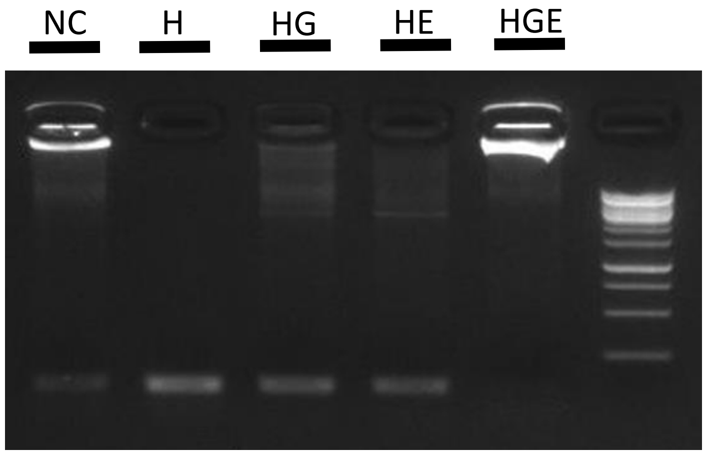

#8,9

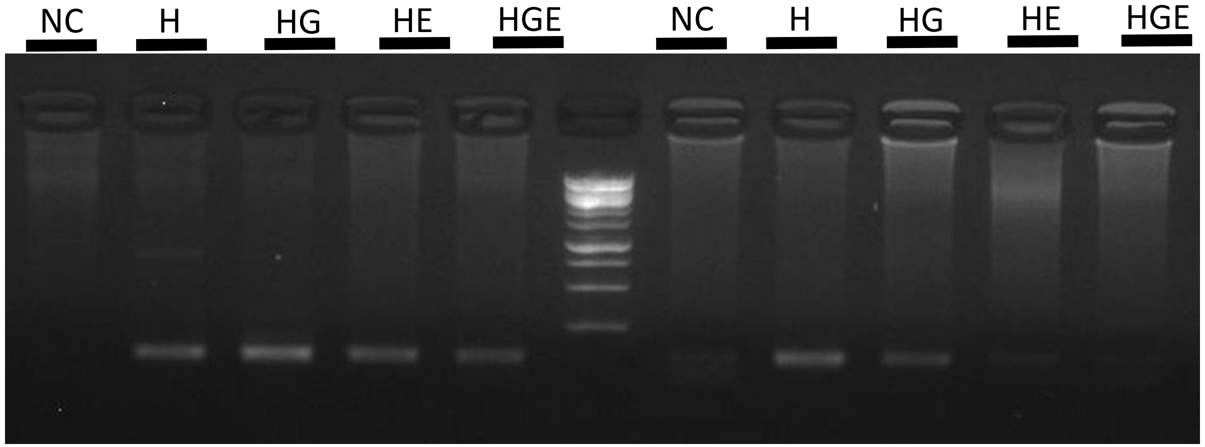

#7,10

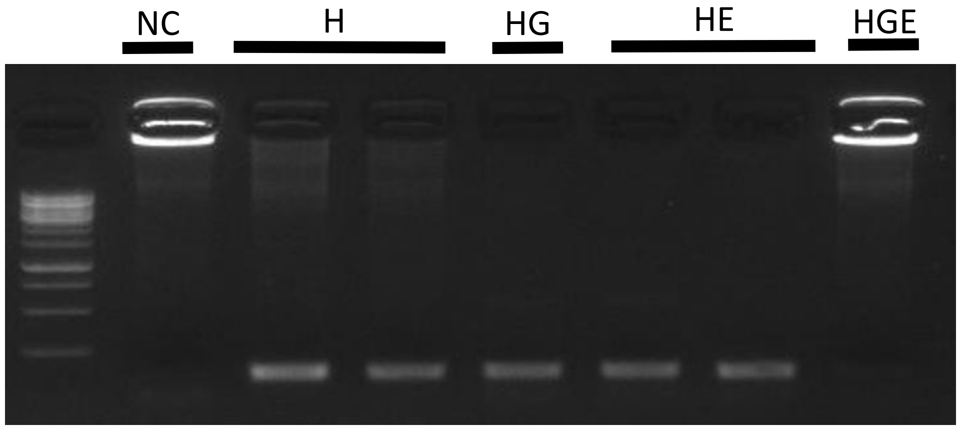

Full-length gels of Figure 5. B.

Gapdh **(27cycle)**

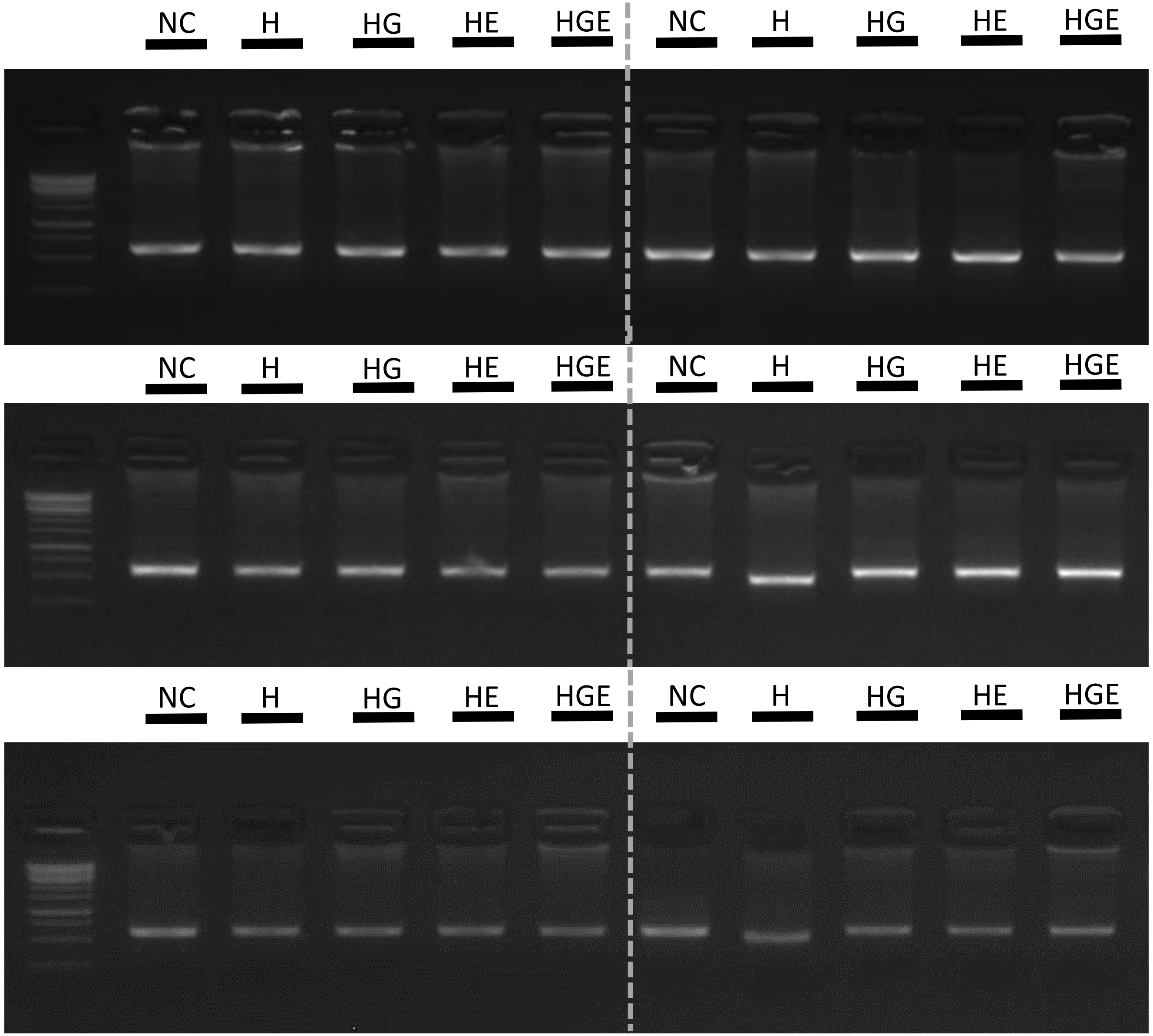

Full-length gels of <u>Figure 5. B.</u>

Gapdh **(27cycle)**

#8,9

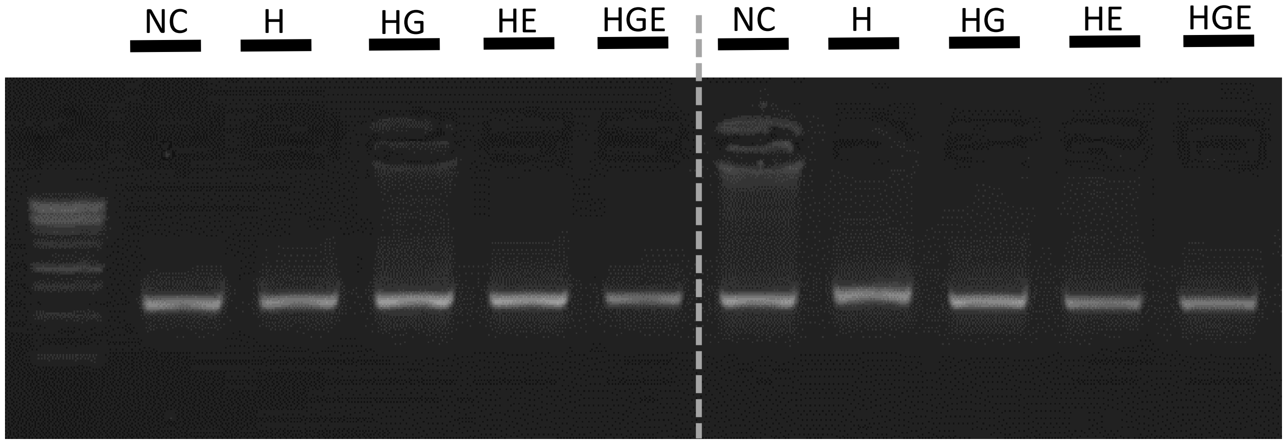

#7,10

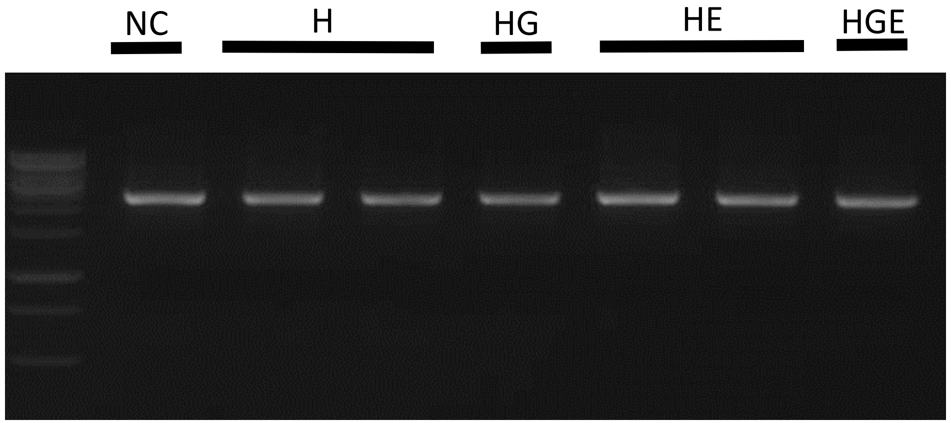

Full-length gels of Figure 5. C.

Tnf (42cycle)

#1,2

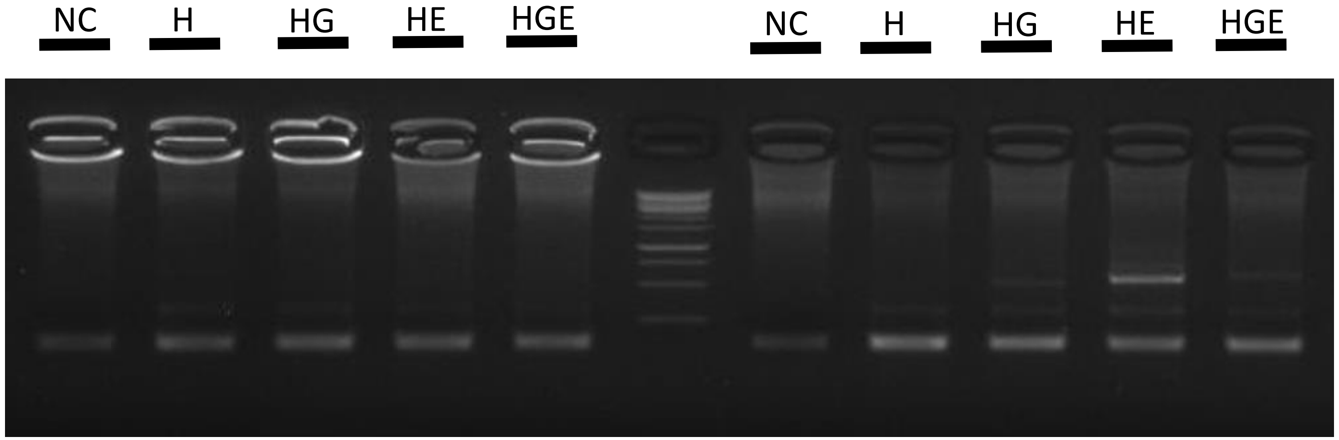

#3,4

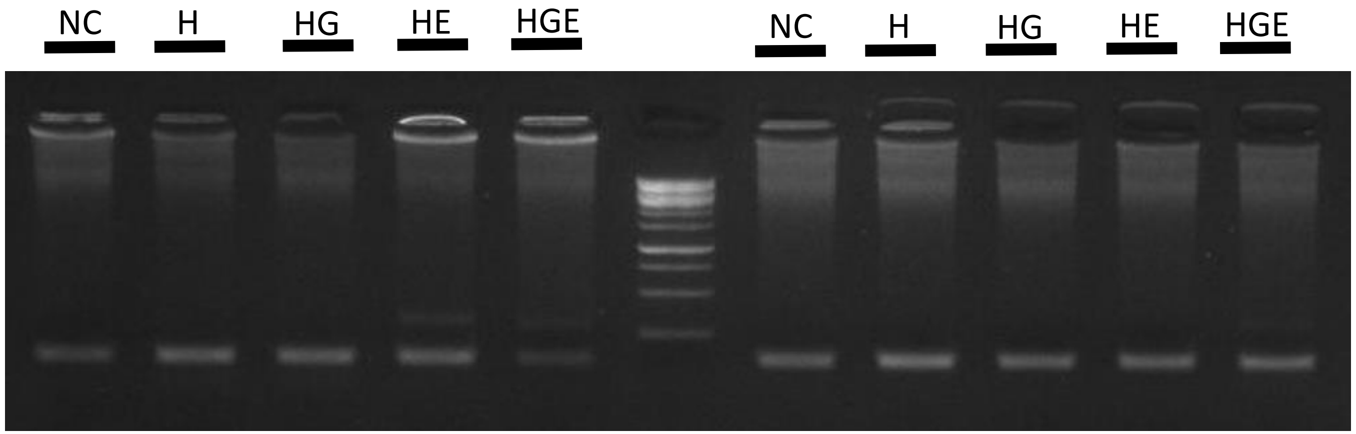

#5

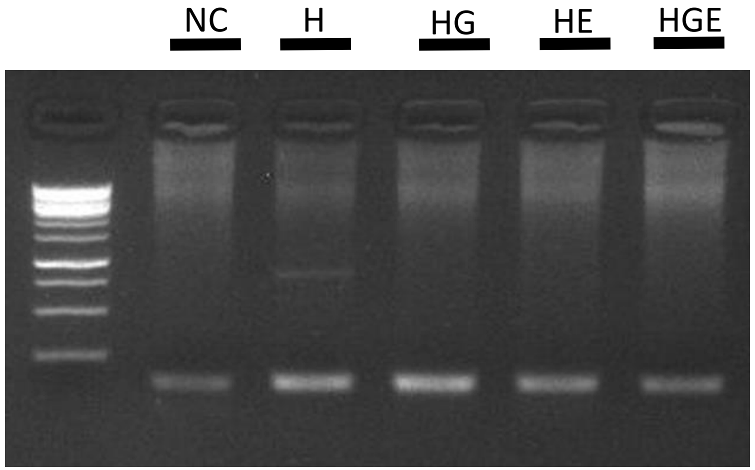

Full-length gels of Figure 5. C.

Tnf (42cycle)

#6(Representative image)

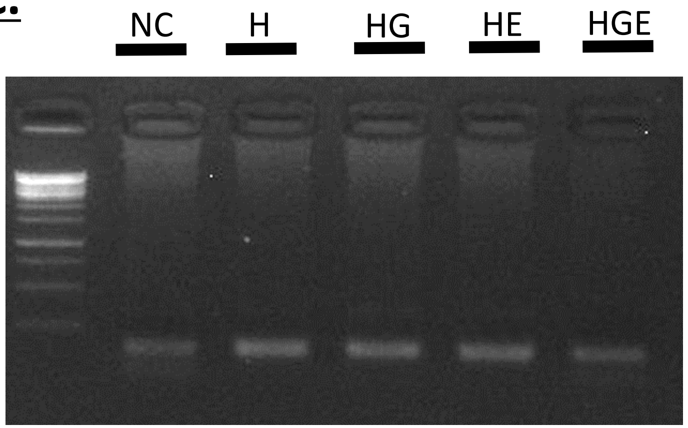

#8

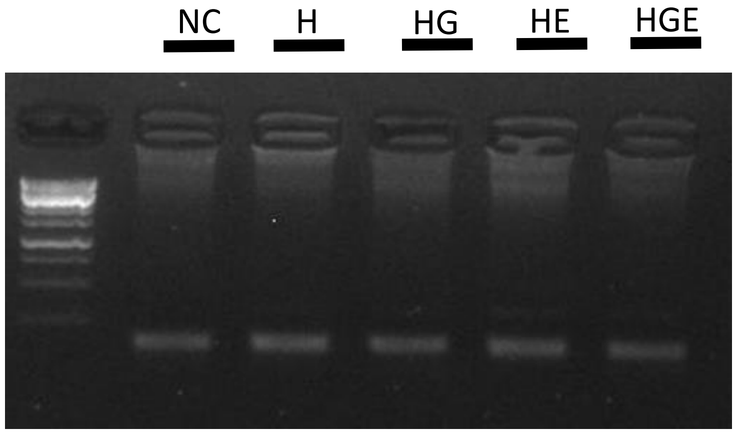

#9

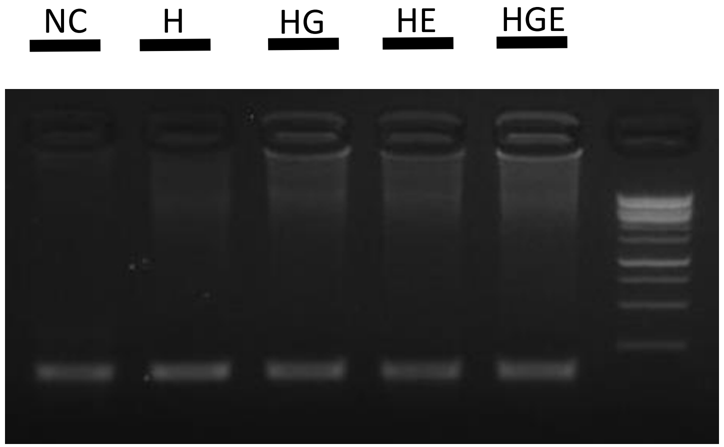

#7,10

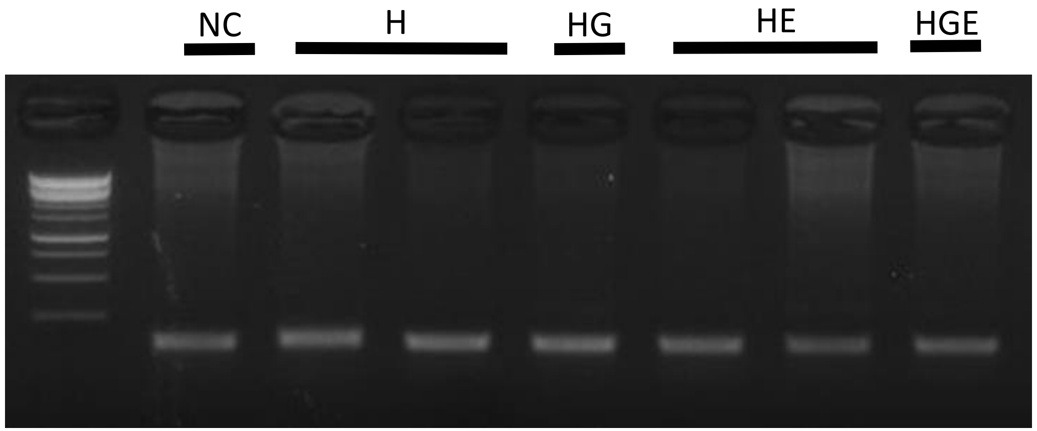

Full-length gels of Figure 5. C.

Il1b (42cycle)

#1,2

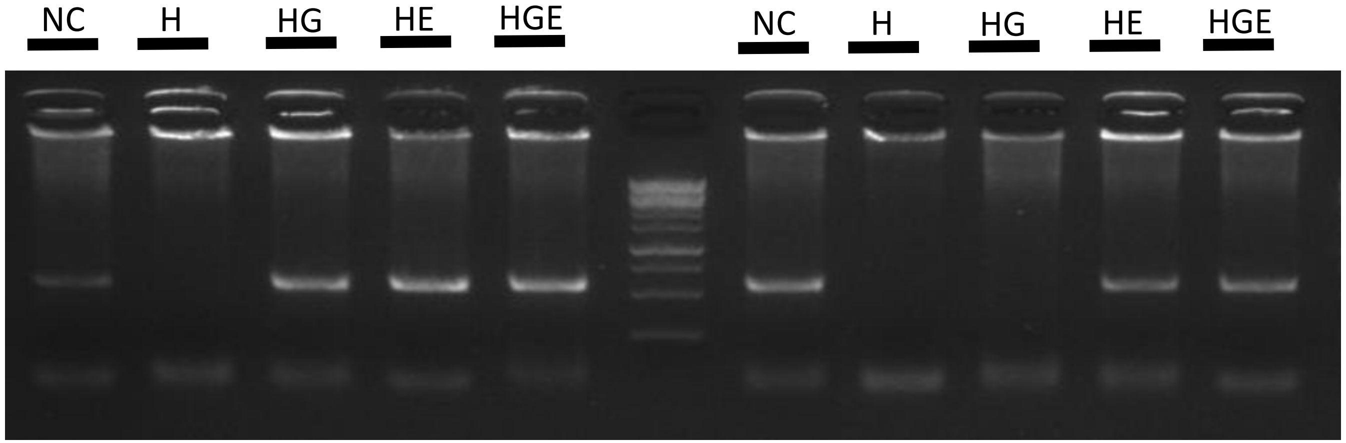

#3

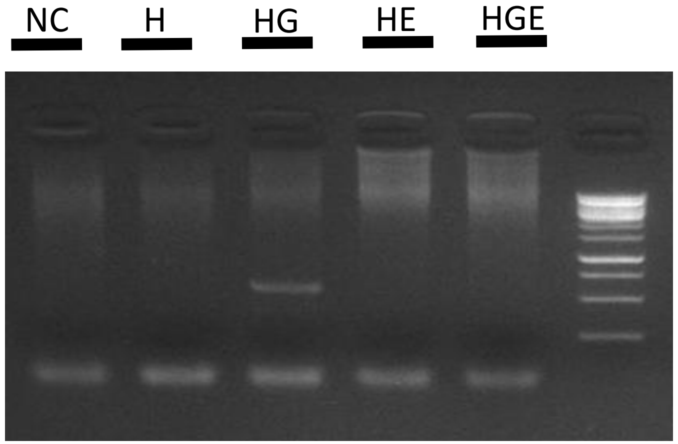

#4(Representative image)

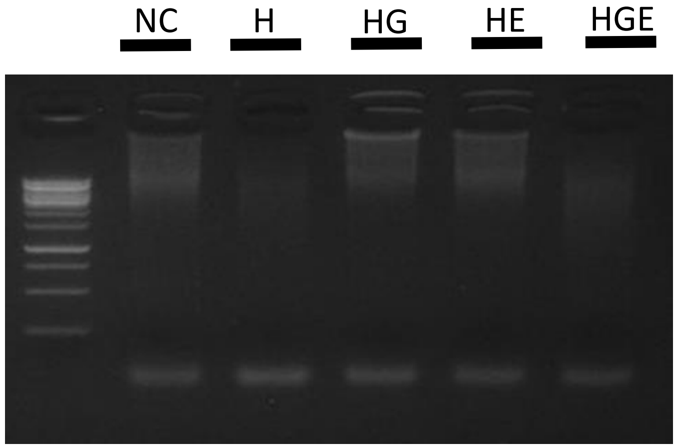

#5

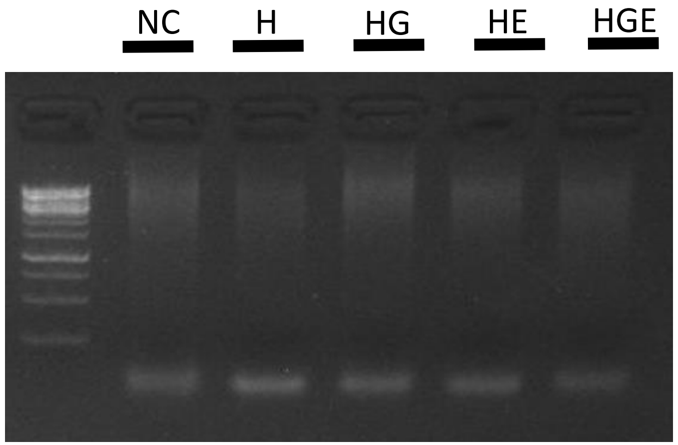

Full-length gels of Figure 5. C.

Il1b (42cycle)

#6

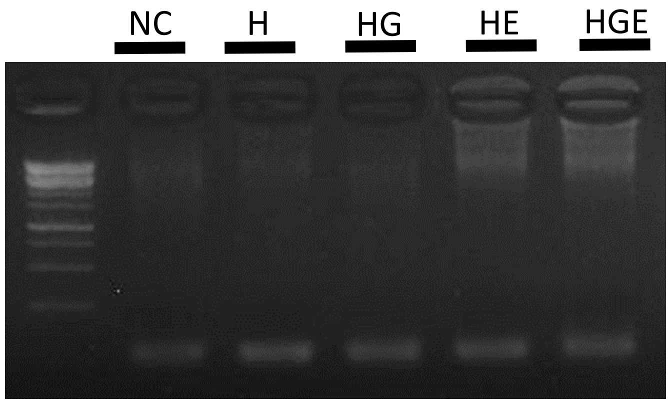

#8

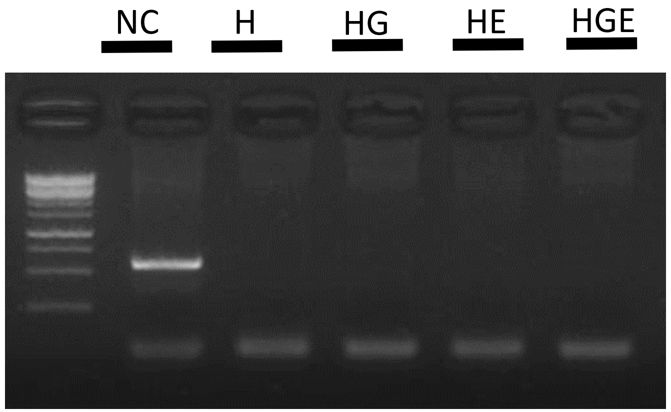

#9

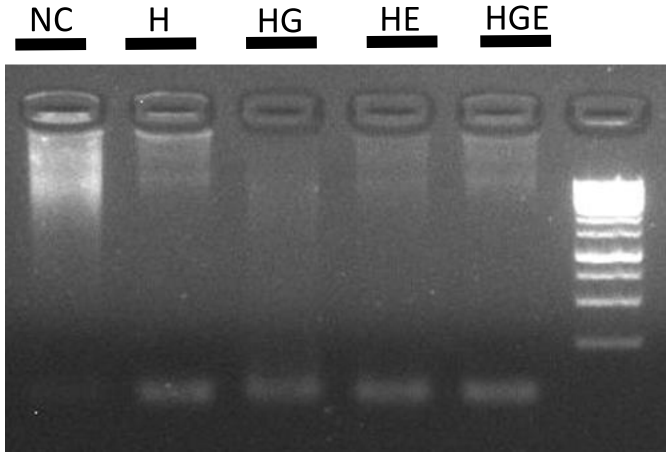

#7,10

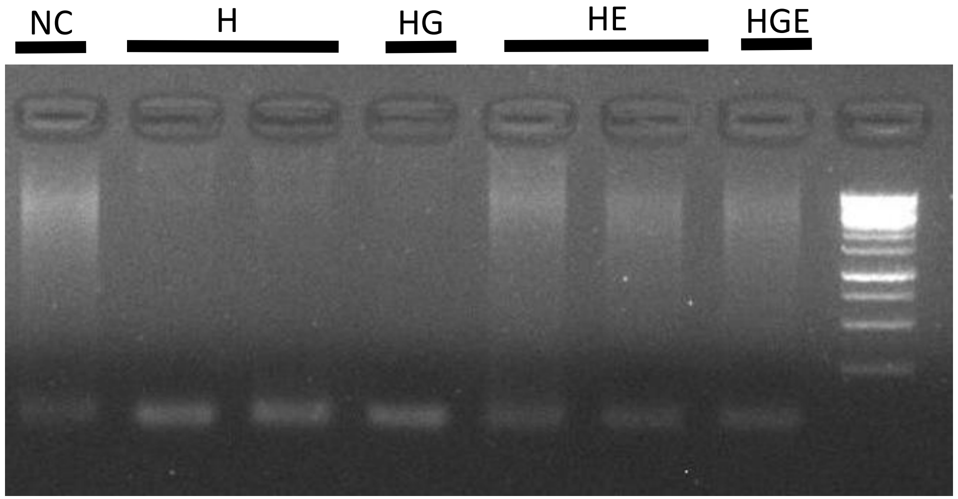

Full-length gels of Figure 5. C.

Gapdh (27cycle)

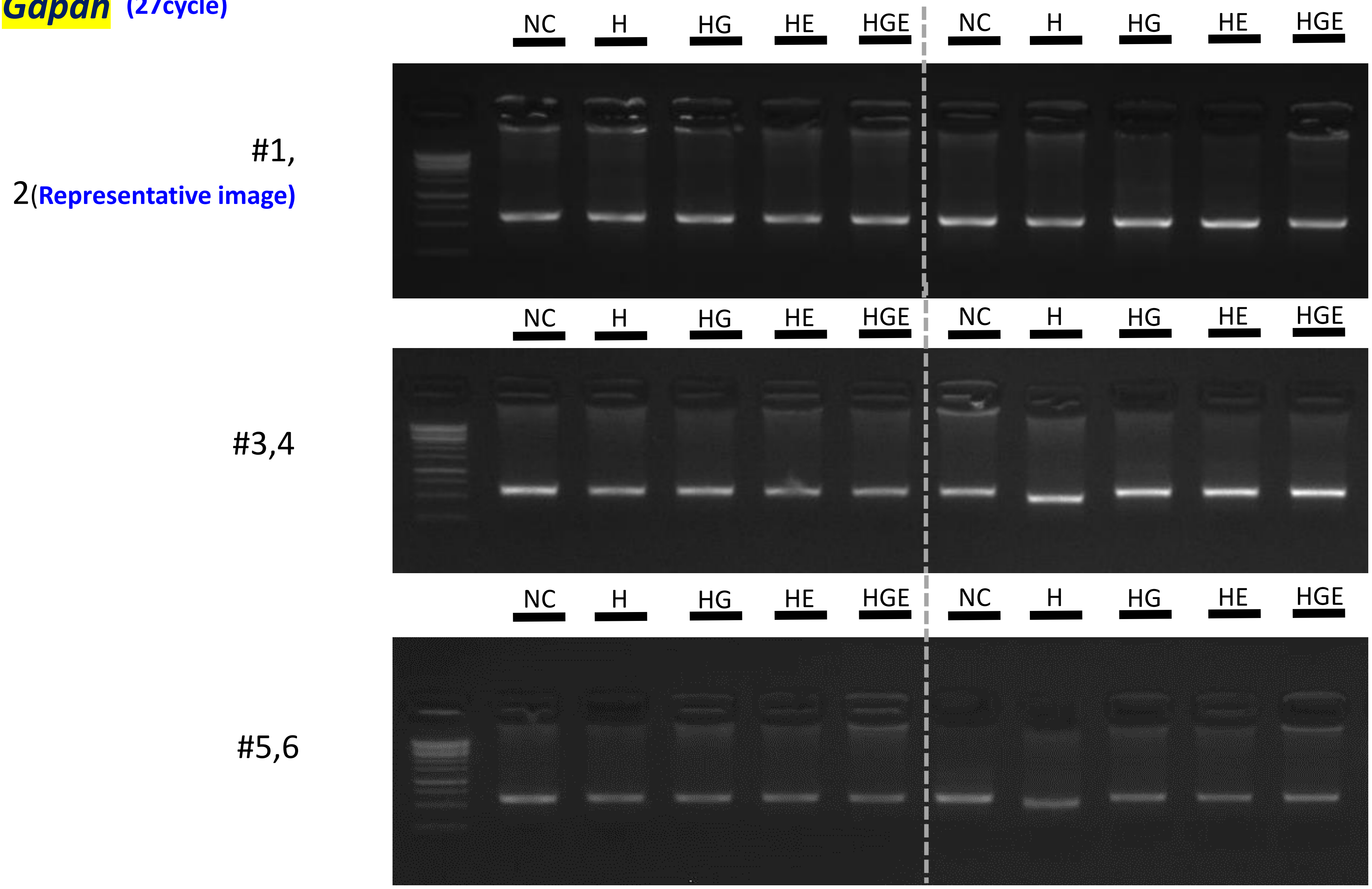

Full-length gels of <u>Figure 5. C.</u>

Gapdh (27cycle)

#8,9

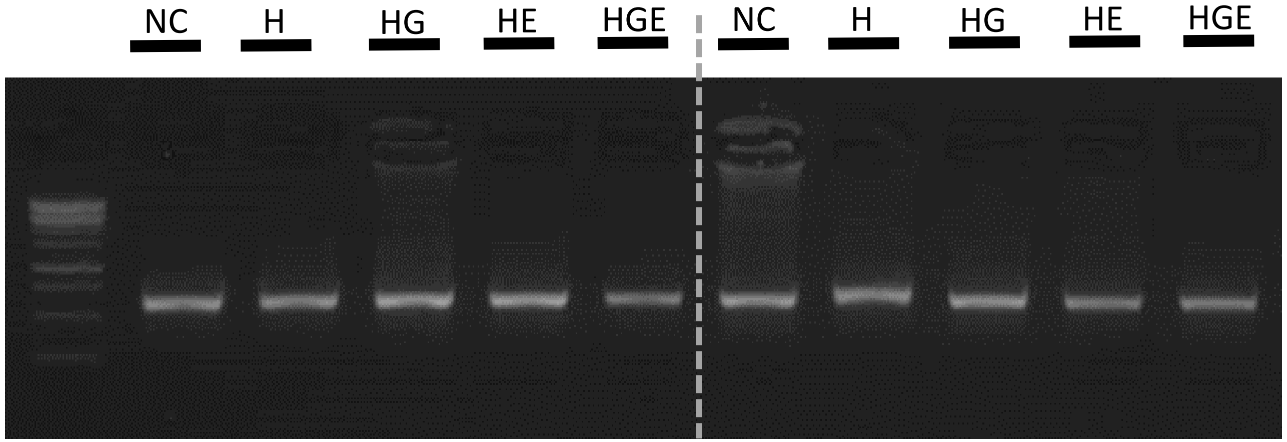

#7,10

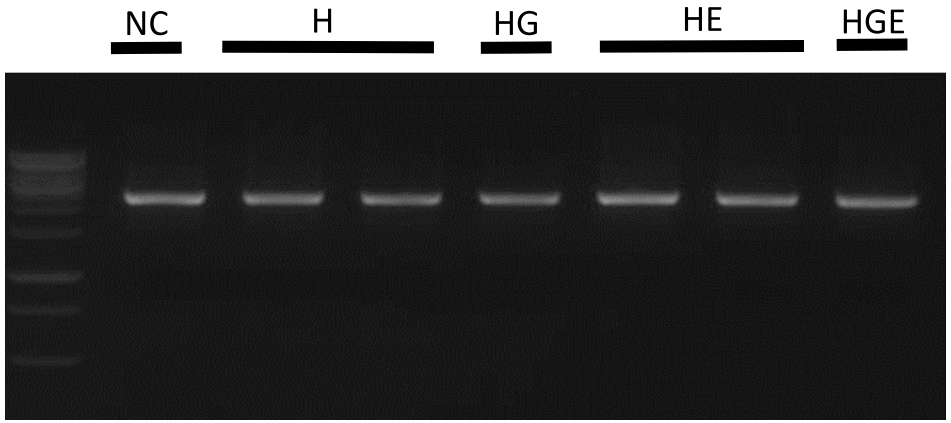

Supplement: Supplementary file 9 — Supplementary Material 9 [file 12906_2025_4968_MOESM9_ESM.pdf]
